# Antiviral Rotenoids and Isoflavones Isolated from *Millettiaoblata* ssp. *teitensis*

**DOI:** 10.1021/acs.jnatprod.3c01288

**Published:** 2024-04-05

**Authors:** Ivan Kiganda, Jonathan Bogaerts, Lianne H. E. Wieske, Tsegaye Deyou, Yoseph Atilaw, Colores Uwamariya, Masum Miah, Joanna Said, Albert Ndakala, Hoseah M. Akala, Wouter Herrebout, Edward Trybala, Tomas Bergström, Abiy Yenesew, Mate Erdelyi

**Affiliations:** † Department of Chemistry, University of Nairobi, P.O. Box 30197, 00100 Nairobi, Kenya; ‡ Department of Chemistry − BMC, Uppsala University, SE-751 23 Uppsala, Sweden; ⊥ Department of Chemistry, Salale University, P.O. Box 245, QPVQ+6C7, Fitche, Ethiopia; ∥ Department of Chemistry, University of Antwerp, 2020 Antwerp, Belgium; § Department of Infectious Diseases/Virology, Institute of Biomedicine, Sahlgrenska Academy, University of Gothenburg, S-413 46 Gothenburg, Sweden; Δ Walter Reed Army Institute of Research - Africa (WRAIR-A), Kenya Medical Research Institute (KEMRI), P.O. Box 54, 40100 Kisumu, Kenya

## Abstract

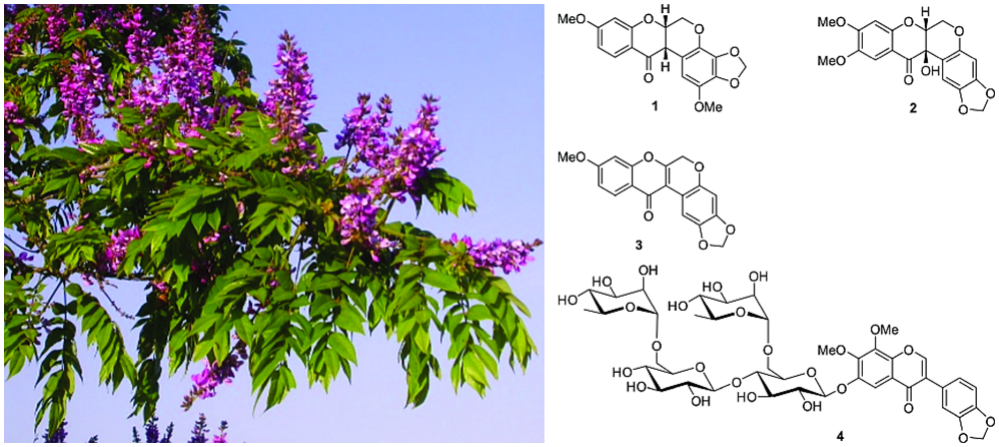

Three new (**1**–**3**) and six known rotenoids (**5**–**10**), along with three known isoflavones (**11**–**13**), were isolated from the leaves of *Millettia oblata* ssp. *teitensis*. A new glycosylated isoflavone (**4**), four known isoflavones (**14**–**18**), and one known chalcone (**19**) were isolated from the root wood extract of the same plant. The structures were elucidated by NMR and mass spectrometric analyses. The absolute configuration of the chiral compounds was established by a comparison of experimental ECD and VCD data with those calculated for the possible stereoisomers. This is the first report on the use of VCD to assign the absolute configuration of rotenoids. The crude leaves and root wood extracts displayed anti-RSV (human respiratory syncytial virus) activity with IC_50_ values of 0.7 and 3.4 μg/mL, respectively. Compounds **6**, **8**, **10**, **11**, and **14** showed anti-RSV activity with IC_50_ values of 0.4–10 μM, while compound **3** exhibited anti-HRV-2 (human rhinovirus 2) activity with an IC_50_ of 4.2 μM. Most of the compounds showed low cytotoxicity for laryngeal carcinoma (HEp-2) cells; however compounds **3**, **11**, and **14** exhibited low cytotoxicity also in primary lung fibroblasts. This is the first report on rotenoids showing antiviral activity against RSV and HRV viruses.

In contrast to the large variety of antimicrobials accessible for the treatment of bacterial and fungal infections, antiviral drugs are so far only available for the treatment of ten human viruses, namely, the human immunodeficiency virus (HIV), the hepatitis B and C viruses, three herpesviruses, the respiratory syncytial virus (RSV), the influenza viruses, the human papillomavirus,^1,2^ and recently for SARS coronavirus-2. The epidemiological control of most viral infections focuses on the isolation of cases, quarantine of contacts, personal protection, and mass vaccination since specific treatments are not available against most viral infections.^3,4^ A variety of viral diseases have evolved to endemics and pandemics and lead to significantly increased mortality rates and social, economic, and political changes.^1,5,6^ Antiviral vaccines and new drugs are thus required. Natural products have been sources of inspiration for the development of new antimicrobial drugs.^7^ Some flavonoids^8^ and specifically flavones,^9^ for instance, exhibit antiviral properties by inhibiting enzymes and preventing virus penetration.^10,11^

Out of the over 260 *Millettia* species (Fabaceae/Leguminosae) distributed over the tropics of Asia, Australia, and Africa, 139 are found in Africa.^12−14^ Traditionally, some of these species have been applied in the treatment of diseases including paralysis, insect bites, snake bites, and dysmenorrhea.^15,16^ The genus *Millettia* is known to be a rich source of various subclasses of flavonoids, especially isoflavones, chalcones, and rotenoids.^17−19^ Some of these compounds presented cytotoxic, antiplasmodial, anti-inflammatory, antileishmanial, and antibacterial activities.^12,15,20^ Isoflavones specifically affect various stages of the viral life cycle by targeting cellular components of importance for viral replication and, accordingly, have an effect on many viruses.^21^ The isolation and biological applications of some rotenoids have recently been reviewed.^22^ We previously isolated rotenoids and isoflavones from the leaves and stem bark of *Millettia oblata* ssp. *teitensis*.^18,19^ Here, we report the investigation of the secondary metabolites of root wood and the reinvestigation of its leaves. We have characterized three new rotenoids from the leaves (**1**–**3**), a new isoflavone glycoside (**4**) from the root wood, and 14 additional known compounds from this plant. The antiviral activity against RSV and human rhinovirus 2 (HRV-2) and the cytotoxicity of the crude extracts and a selection of the isolated compounds were explored in human laryngeal epidermoid carcinoma (HEp-2) and cervical cancer (HeLa) cells and in human embryonic lung fibroblasts.

## Results and Discussion

The CH_2_Cl_2_/MeOH (1:1) extract of the leaves of *M. oblata* ssp. *teitensis* was subjected to a silica gel column chromatography, followed by purification by Sephadex LH-20 and preparative thin layer chromatography (TLC) to afford the three new rotenoids oblarotenoid E (**1**), oblarotenoid F (**2**), and oblarotenoid G (**3**). Similar treatment of the root wood afforded the new isoflavone glycoside obloneside (**4**), the six known rotenoids oblarotenoid C (**5**),^18^ oblarotenoid A (**6**),^18^ oblarotenoid D (**7**),^18^ 12a-hydroxymunduserone (**8**),^18^ tephrosin (**9**),^23^ and deguelin (**10**),^17^ the seven known isoflavones ichthynone (**11**),^24^ 7,2′,5′-trimethoxy-3′,4′-methylenedioxyisoflavone (**12**),^18^ isoerythrin-A-4′-prenyl ether (**13**),^25^ 4′-prenyloxyderrone (**14**),^26^ aglycuneatin methyl ether (**15**),^27^ calopogonium isoflavone B (**16**),^28^ maximaisoflavone G (**17**),^17^ and milldurone (**18**),^27^ and the known chalcone isobavachromene (**19**).^29^ The known compounds were identified by comparison of the experimental spectroscopic data to the literature data (Supporting Information). The ^13^C NMR assignment of compounds **1**–**4** was confirmed by CSEARCH.^30^
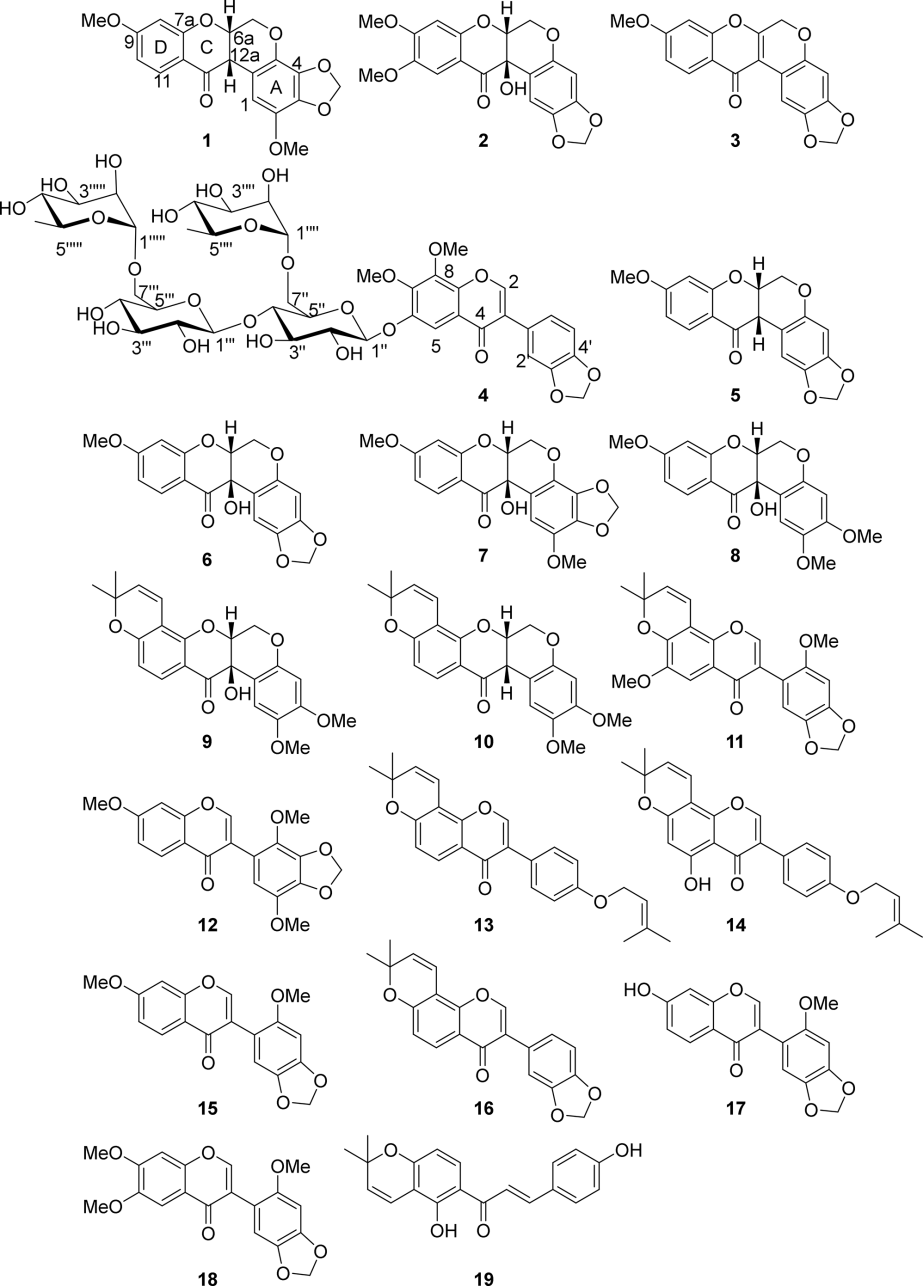


**Figure 1:**
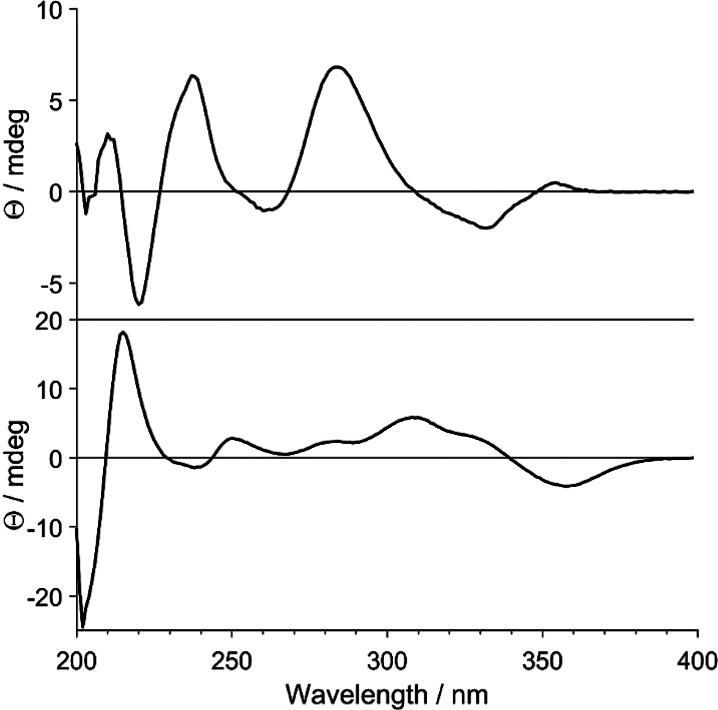
Experimental ECD spectra of compounds **1** (top) and **2** (bottom).

**Figure 2:**
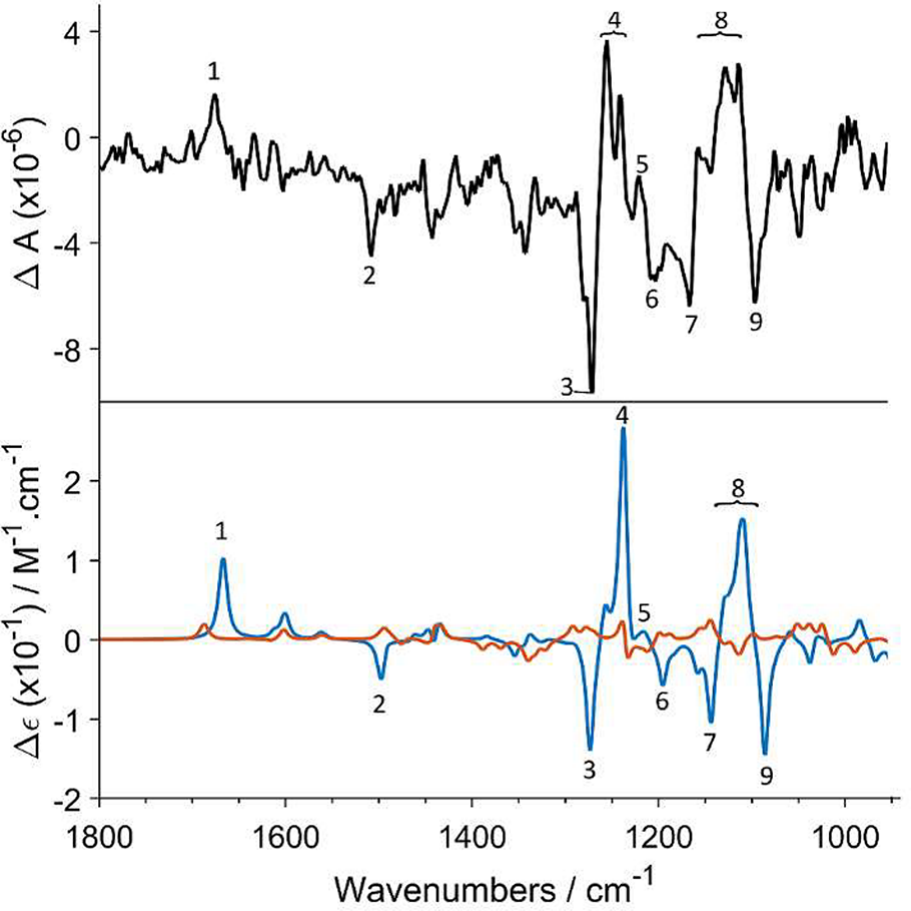
VCD spectra observed for **1** (black) and those predicted for its (6a*S*,12a*S*)-**1** (blue) and (6a*S*,12a*R*)-**1** (red) stereoisomers. The numbers 1–9 indicate band assignment of key importance for AC assignment and are given to facilitate comparison of the experimental and simulated spectra.

Compound **1** was obtained as a white, amorphous solid that showed UV absorption maxima at 280 and 305 nm. Its molecular formula was established as C_19_H_16_O_7_ based on HRESIMS ([M + H]^+^ at *m*/*z* 357.0976, calcd for C_19_H_17_O_7_ 357.0974, Figures S1–S8, Supporting Information) and NMR data (Table 1). The ^1^H NMR spectrum displayed four sets of coupled aliphatic protons at δ_H_ 4.21 (br d, *J* = 12.1 Hz, H-6), 4.65 (dd, *J* = 12.1, 3.0 Hz, H-6), 4.94 (m, H-6a), and δ_H_ 3.81 (H-12a, overlapping with a OMe signal), which are consistent with the NMR signals expected for the B ring of a rotenoid skeleton.^31^ The ^13^C NMR spectrum displayed the corresponding signals at δ_C_ values of 66.8 (C-6), 72.7 (C-6a), 189.0 (C-12), and 45.0 (C-12a). Two methoxy groups (δ_H_ 3.71 and 3.81; δ_C_ 57.2 and 56.2) and a methylenedioxy group (δ_H_ 5.91 and δ_H_ 5.97; δ_C_ 102.7) were also identified based on their characteristic chemical shifts. The three mutually coupled aromatic protons of ring D showed an AMX spin system at δ_H_ 7.85 (d, *J* = 8.9 Hz, H-11), δ_H_ 6.59 (dd, *J* = 8.9, 2.3 Hz, H-10), and δ_H_ 6.44 (d, *J* = 2.3 Hz, H-8) (Table 2 and Figures S1–S8, Supporting Information). The placement of the methoxy group at C-9 (δ_C_ 167.0) was supported by the HMBC cross peaks from H-8 (δ_H_ 6.44), H-10 (δ_H_ 6.59), and H-11 (δ_H_ 7.85) to C-9 (δ_C_ 167.0) and from OMe-9 (δ_H_ 3.85) to C-9. The OMe-9 group further showed a NOE correlation to H-8 (δ_H_ 6.44). This substitution pattern is consistent with the biogenetically expected oxygenation (OMe) at C-9.^32^ The remaining aromatic proton (δ_H_ 6.39) was assigned to H-1, based on its HMBC correlations with C-1a (δ_C_ 109.4), C-4a (δ_C_ 133.2), and C-12a (δ_C_ 45.0). This places the second methoxy group and the methylenedioxy group at C-2, C-3, and C-4, giving rise to two alternative structures that have the methoxy group at C-2 or at C-4. The observed ^13^C NMR chemical shift of the methoxy (δ_C_ 57.2) better fits to oxygenation at C-2, as it would be expected to have a chemical shift above 59 ppm if it was at C-4, due to di-*ortho* substitution.^33^ The placement of the methoxy group at C-2 (δ_C_ 138.9) was further supported by the NOE correlation between OMe-2 (δ_H_ 3.73) and H-1 (δ_H_ 6.31) and by the HMBC correlations of H-1 (δ_H_ 6.31) with C-1a (δ_C_ 109.4), C-2 (δ_C_ 138.9), C-3 (δ_C_ 136.3), C-4a (δ_C_ 133.2), and C-12a (δ_C_ 45.0) as well as between OMe-2 (δ_H_ 3.73) and C-2 (δ_C_ 138.9) (Table 1). Based on the spectroscopic data, the gross structure of compound **1** was identified as 2,9-dimethoxy-3,4-methylenedioxyrotenoid. It has an uncommon trisubstitution with a methoxy and a methylenedioxy group at its ring A, which may indicate it to be biosynthesized from 7,2′,5′-trimethoxy-3′,4′-methylenedioxyisoflavone (**12**), a co-metabolite that has the same oxygenation pattern in its B ring as the A ring of **1**. The chemical shift of H-1 (δ_H_ 6.39) along with the small coupling constant (*J* = 2.9 Hz) between H-6 (δ_H_ 4.65 and 4.21) and H-6a (δ_H_ 4.94) suggested that H-6a is equatorially oriented, and compound **1** hence has a *cis* configuration at its B/C ring junction, which has been reported as the thermodynamically most favorable configuration.^18^ The NOE correlation between H-6a (δ_H_ 4.94) and H-12a (δ_H_ 3.81) corroborates the *cis* configuration of the B/C ring junction. To determine the absolute configuration, the electronic circular dichroism (ECD) spectrum of **1** was recorded (Figure 1). According to Slade et al. (2005), dextrorotatory rotenoids with a *cis*-B/C ring junction, showing a positive Cotton effect (CE) at 300–330 nm (π to π* transition) and negative CE at 348–360 nm (for n to π* transition), are associated with the (6a*R*,12a*R*) configuration. On the other hand, levorotatory rotenoids with a *cis*-B/C ring junction, showing a negative CE at 300–330 nm and a positive CE at 348–360 nm, are associated with a (6a*S*,12a*S*) configuration. The n to π* transition at 348–360 nm is generally weak, and for many rotenoids, it has not been observed and thus may not be a reliable indicator for determination of rotenoids’ absolute configuration.^18^ Compound **1** is dextrorotatory with a [α] = ^20^_D_ +44.0 (*c* 0.2 CH_3_OH) and showed a negative CE at 330 nm for its π to π* transition in its ECD spectrum. This contradicts the above literature proposal.^34^ A similar contradiction has previously been reported for some rotenoids, which had either low positive or low negative specific rotation.^18^ In contrast to prenylated rotenoids with a *cis*-B/C ring junction that show specific rotations of [α]^20^_D_ < −100 or [α]^20^_D_ > +100, with the sign of the rotation reliably indicating the absolute configuration,^35,36^ nonprenylated rotenoids with a *cis*-B/C ring junction typically show specific rotations of −50 < [α]^20^_D_ < +50, and the sign of the rotation is not reliable for determining their absolute configuration.^18^ For compound **1**, which has a positive specific rotation of [α]^20^_D_ +44.0 (*c* 0.2 CH_3_OH), we relied on the ECD spectrum and assigned its absolute configuration as (6a*S*,12a*S*)-**1** upon comparison of its ECD spectrum with that reported for (6a*S*,12a*S*)-9-methoxy-2,3-methylenedioxyrotenoid, a related rotenoid whose absolute configuration was established by single-crystal X-ray crystallography.^34^ The absolute configuration of compound **1** was confirmed by acquiring a vibrational circular dichroism (VCD) spectrum (Figure 2), which showed a good match with that simulated for the (6a*S*,12a*S*) stereoisomer of **1**, whereas it showed no match for the predicted spectrum of the (6a*S*,12a*R*) stereoisomer. The vibrational IR pattern of **1** at 1300–1200 cm^–1^ (Figure S135, Supporting Information) was further consistent with a *cis* configuration at the B/C ring junction.^37,38^ This is the first report of the use of VCD spectroscopy to determine the relative and absolute configuration of rotenoids. Based on the spectroscopic data, the new compound (**1**) was identified as (6a*S*,12a*S*)-2,9-dimethoxy-3,4-methylenedioxyrotenoid and was given the trivial name oblarotenoid E. It is structurally closely related to the previously reported oblarotenoid C,^18^ differing in the substitution of its ring A.

**Table 1:** NMR Spectroscopic Data (^1^H 500 and ^13^C 125 MHz) of Oblarotenoids E (**1**), F (**2**) (CD_2_Cl_2_^a^), and G (CDCl_3_)

	**1**			**2**			**3**		
position	δ_C_, type	δ_H_ (*J* in Hz)	HMBC^b^	δ_C_, type	δ_H_ (*J* in Hz)	HMBC	δ_C_, type	δ_H_ (*J* in Hz)	HMBC
1	106.6, CH	6.31 s	C-1a, C-2, C-3, C-4a, C-12a	105.9, CH	6.54 s	C-1a, C-2, C-3, C-4a, C-12a	107.1, CH	8.32 s	C-2, C-3, C-4a, C-12a
1a	109.4, C			109.9, C			111.8, C		
2	138.9, C			149.5, C			142.9, C		
3	136.3, C			142.4, C			147.4, C		
4	136.9, C			99.3, CH	6.46 s	C-1a, C-2, C-3	98.7, CH	6.53 s	C-1a, C-2, C-3
4a	133.2, C			149.5, C			147.5, C		
6	66.8, CH_2_	4.65 dd (12.5, 2.6)	C-4a, C-6a, C-12a	64.0, CH_2_	4.58 dd (13.5, 2.6)	C-4a, C-6a, C-12a	65.0, CH_2_	4.96 s	C-4a, C-6a, C-12a
		4.21 br d (12.5)	C-4a,C-6a		4.46 d (13.5, 2.6)	C-4a,C-6a			
6a	72.7, CH	4.94 dd (2.6)	C-1a, C-4a, C-6, C-12, C-12a	76.4, CH	4.56 br d (3.6)	C-1a, C-4a, C-6, C-12, C-12a	156.8, C		
7a	163.0, C			157.5, C			156.7, C		
8	100.9, CH	6.44 d (2.3)	C-7a, C-9, C-10, C-11a	100.3, CH	6.40 s	C-7a, C-9, C-10, C-11a	100.3, CH	6.83 d (2.2)	C-7a, C-9, C-10, C-11a
9	167.0, C			157.3, C			164.0, C		
10	110.9, CH	6.59 dd (8.9, 2.3)	C-8, C-9, C-11a	145.3, C		C-8, C-9, C-11a	114.7, CH	7.00 dd (8.9, 2.2)	C-8, C-11a
11	129.6, CH	7.85 d (8.9)	C-7a, C-9, C-12	106.9, CH	7.25 s	C-7a, C-9, C-12	127.9, CH	8.20 d (8.9)	C-7a, C-9, C-12
11a	113.1, C			109.5, C			118.7, C		
12	189.0, CO			191.3, CO			174.2, CO		
12a	45.0, CH	3.81^c^	C-1a, C-4, C-6	67.9, C			112.3, C		
OCH_2_O	102.7, CH_2_	5.97 d (1.4); 5.91 d (1.4)	C-3, C-4	101.5, CH_2_	5.83 d (1.4); 5.86 d (1.4)	C-2, C-3	101.5, CH_2_	5.96 s	C-2, C-3
OMe-2	57.2, CH_3_	3.73 s	C-2						
OMe-9	56.2, CH_3_	3.81 s	C-9	56.4, CH_3_	3.86 s	C-9	56.0, CH_3_	3.92 s	C-9
OMe-10				56.5, CH_3_	3.88 s	C-10			

a CD_2_Cl_2_ was used due to the gradual color change observed in the slightly acidic CDCl_3_.

b HMBC correlations, optimized for 6 Hz, are from the stated proton(s) to the indicated carbon.

c Signal overlapping with that of OMe-9.

**Table 2:** NMR Spectroscopic Data (^1^H 800 and ^13^C 200 MHz, CD_3_OD) for Obloneside (**4**)

position	δ_C_, type	δ_H_ m (*J* in Hz)	HMBC
2	154.2, CH	8.17 s	C-1′, C-3, C-4, C-8a
3	126.9, C		
4	177.1, CO		
4a	115.2, C		
5	101.8, CH	7.12 s	C-4, C-4a, C-6, C-7, C-8a
6	157.0, C		
7	142.1, C		
8	153.9, C		
8a	155.5, C		
1′	126.0, C		
2′-	110.8, CH	7.03 d (1.7)	C-3, C-3′, C-4′, C-6′
3′	148.9, C		
4′	148.9, C		
5′	109.0, C	6.84 d (8.0)	C-1′, C-3′, C-4′
6′	123.8, CH	6.97 dd (8.0, 1.7)	C-2′, C-3, C-4′
OCH_2_O	102.5, CH_2_	5.98 d (1.2)	C-3′, C-4′
OMe-7	62.8, OMe	3.92 s	C-7
OMe-8	62.3, OMe	3.91 s	C-8
β-glycoside moiety 1			
1″	101.6, CH	5.05 d (7.9)	C-3″, C-5″, C-6
2″	73.6, CH	3.78 dd (8.7, 7.9)	C-1″, C-4″
3″	76.7, CH	3.70 dd (9.5, 7.9)	C-4″, C-5″, C-7″
4″	89.4, CH	3.61 dd (9.5, 9.1)	C-1‴, C-2″, C-3‴
5″	70.2, CH	3.46 dd (9.3, 9.1, 7.3)	C-1″, C-3″, C-4″, C-7″
7″	67.8, OCH_2_	4.08 d (9.3)	C-1‴′, C-2″, C-4″, C-5″
		3.58 (7.3)	
β-glycoside moiety 2			
1‴	105.4, CH	4.53 d (7.8)	C-3″, C-4″, C-5‴
2‴	75.2, CH	3.34 dd (8.9, 7.8)	C-1‴, C-5‴
3‴	76.6, CH	3.55 dd (9.7, 8.9)	C-2‴, C-4‴
4‴	71.7, CH	3.32 dd (9.7, 9.7)	C-3‴, C-5‴, C-7‴
5‴	77.6, CH	3.43 ddd (9.7, 9.2, 7.2)	C-1‴, C-3‴, C-4‴, C-7‴
7‴	68.5, OCH_2_	4.04 d (9.2)	C-1‴″, C-2‴″, C-4‴
		3.58 d (7.2)	
α-rhamnoside moiety 3			
1‴′	102.1, CH	4.71 d (1.2)	C-2‴′, C-3‴′, C-5‴′, C-7″
2‴′	71.9, CH	3.90 dd (3.3, 1.2)	C-1‴′, C-3‴′, C-4‴′
3‴′	72.2, CH	3.70 dd (9.4, 3.3)	C-4‴′, C-5‴′
4‴′	74.1, CH	3.37 dd (9.5, 9.4)	C-2‴′, C-3‴′, C-5‴′
5‴′	70.0, CH	3.67 dd (9.5, 6.2)	C-1‴′, C-3‴′, C-4‴′
7‴′	18.1, CH_3_	1.28 d (6.2)	C-4‴′, C-5‴′
α-rhamnoside moiety 4			
1‴″	102.4, CH	4.74 d (1.4)	C-2‴″, C-3‴″, C-5‴″, C-7‴
2‴″	72.0, CH	3.92 dd (3.4, 1.4)	C-1‴″, C-4‴″
3‴″	72.4, CH	3.80 dd (9.4, 3.4)	C-1‴″, C-2‴″, C-4‴″
4‴″	74.0, CH	3.39 dd (9.5, 9.4)	C-2‴″, C-3‴″, C-5‴″
5‴″	69.9, CH	3.63 dd (9.5, 6.2)	C-1‴″, C-3‴″, C-4‴″
7‴″	18.0, CH_3_	1.21 d (6.2)	C-4‴″, C-5‴″

**Figure 3:**
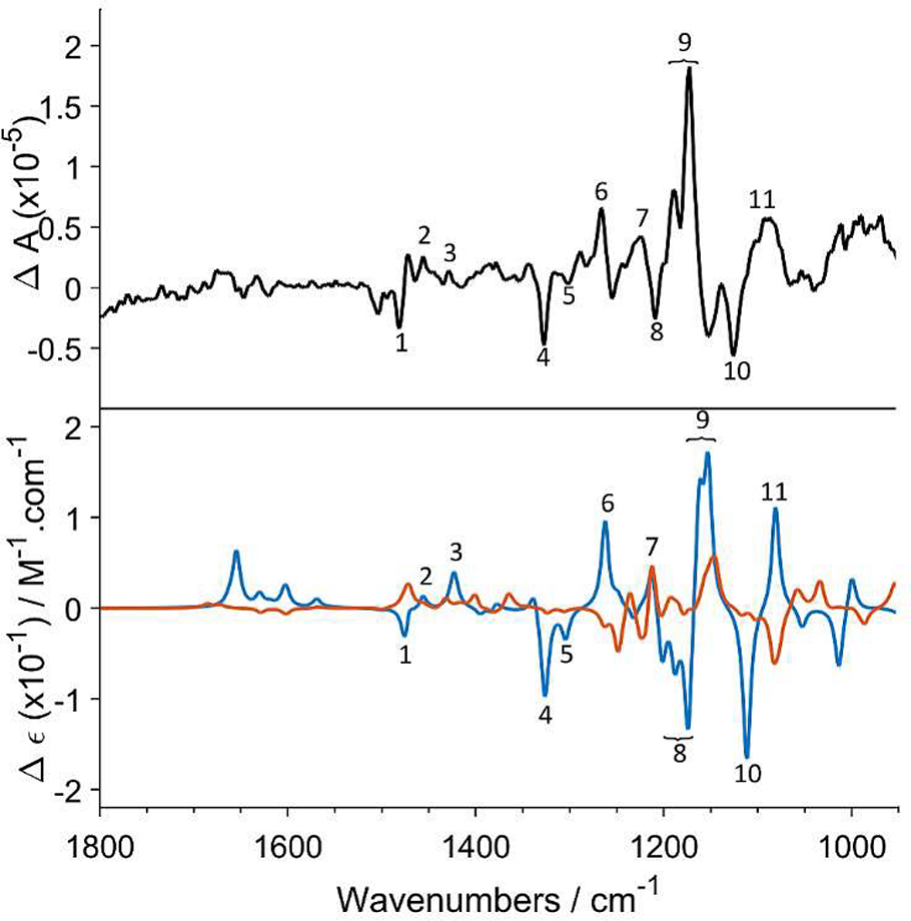
Experimental VCD spectrum of **2** (black) and the spectra predicted for (6a*R*,12a*R*)-**2** (blue) and (6a*R*,12a*S*)-**2** (red). The numbers 1–11 indicate band assignment of key importance for AC assignment and are given to facilitate comparison of the experimental and simulated spectra.

Compound **2** was isolated as a white, amorphous solid. Its molecular formula was established as C_19_H_16_O_8_ based on MS ([M + H]^+^ at *m*/*z* 373.2) and HRESIMS data ([M + H – H_2_O]^+^ at *m*/*z* 355.0354), consistent with the formula C_19_H_15_O_7_ (calcd 355.0818) and NMR data (Table 1). The UV absorbance at λ_max_ 285 and 305 nm as well as the ^1^H NMR [δ_H_ 4.58 dd, *J* = 13.5, 2.6 Hz (H-6), 4.46, dd *J* = 13.5, 2.6 Hz (H-6), and 4.56, d, *J* = 3.6 Hz (H-6a)] and ^13^C NMR [(δ_C_ 64.6 (C-6), 76.4 (C-6a), 191.3 (C-12), and 67.9 (C-12a)] data (Table 1, Figures S10–S17, Supporting Information) were consistent with a 12a-hydroxyrotenoid skeleton.^18^ The NMR spectra indicated a methylenedioxy (δ_H_ 5.83 and 5.86; δ_C_ 101.5) and two methoxy (δ_H_ 3.86 and 3.88; δ_C_ 56.4 and 56.5) functionalities. The four singlet aromatic protons H-1 (δ_H_ 6.54), H-4 (δ_H_ 6.46), H-8 (δ_H_ 6.40), and H-11 (δ_H_ 7.25) were consistent with C-2, C-3, C-9, and C-10 oxygenation of the 12a-hydroxyrotenoid skeleton. The NOE correlation of H-11 (δ_H_ 7.25) with OMe-10 (δ_H_ 3.88) and the NOE of OMe-9 (δ_H_ 3.86) with H-8 (δ_H_ 6.40) along with the HMBC of H-8 (δ_H_ 6.40) to C-7a (δ_C_ 157.5), C-9 (δ_C_ 157.3), C-10 (δ_C_ 149.5), and C-11a (δ_C_ 109.5) as well as of H-10 (δ_H_ 6.59) to C-8 (δ_C_ 100.3), C-9 (δ_C_ 157.3), and C-11a (δ_C_ 109.5) placed the two OMe groups of ring D at C-9 (δ_C_ 145.3) and C-10 (δ_C_ 157.3), respectively. The methylenedioxy moiety was placed at C-2/C-3 based on the HMBC correlations of H-1 (δ_H_ 6.54) to C-1a (δ_C_ 109.9), C-2 (δ_C_ 109.9), C-3 (δ_C_ 109.9), C-4a (δ_C_ 149.5), and C-12a (δ_C_ 67.9), as well as of H-4 (δ_H_ 6.46) to C-1a (δ_C_ 109.9), C-2 (δ_C_ 109.9), and C-3 (δ_C_ 109.9), leading to the planar structure 9,10-dimethoxy-2,3-methylenedioxy-12a-hydroxyrotenoid for compound **2**. The chemical shift of H-1 (δ_H_ 6.54) along with the small coupling constants, *J* ≤ 3.3 Hz, between H-6a (δ_H_ 4.59) and the CH_2_-6 protons (δ_H_ 4.58 and 4.46) indicated a *cis* B/C ring junction,^34^ whereas the negative Cotton effect at ca. 340 nm suggested a (6a*R*,12a*R*)-**2** absolute configuration.^18^ Similar to compound **1**, the positive specific rotation [α]^20^_D_ +42 (*c* 0.3 CH_3_OH) was not found to be reliable in assigning the configuration of **2**. Instead, its (6a*R*,12a*R*)-**2** absolute configuration was determined by the comparison of its experimental VCD (Figure 3) with calculated spectra. This configuration was corroborated by the IR band pattern between 1350 and 1000 cm^–1^ (Figure S136, Supporting Information), which indicated a *cis* configuration.^37,38^ Based on the spectroscopic data, the new compound **2** was identified as (6a*R*,12a*R*)-9,10-dimethoxy-2,3-methylenedioxy-12a-hydroxyrotenoid and was given the trivial name oblarotenoid F. It is structurally closely related to the previously reported oblarotenoid A,^18^ differing in the substitution of its ring D. It should be emphasized that despite structural similarities, the specific rotation of oblarotenoid F, [α]^20^_D_ +42, has an opposite sign as compared to those of oblarotenoid A, [α]^20^_D_ −38.3, and of (−)-(6a*R*,12a*R*)-12a-hydroxy-α-toxicarol, [α]^20^_D_ −108, recently reported from *Millettia brandisiana*.^39^ The ECD and the specific rotation data of rotenoids may not be complementary, which has been previously pointed out.^18,39^

Compound **3** was isolated as a light yellow solid. Its molecular formula, C_18_H_12_O_6_, was established based on HRESIMS ([M + H]^+^*m*/*z* 325.0714, calcd 325.0712) and NMR data (Table 1, Figures S18–S24 Supporting Information). UV absorptions at λ_max_ 237, 278, and 310 nm along with the NMR data, especially the chemical shifts of the oxygenated methylene protons H-6 (δ_H_ 4.96) and of C-6a (δ_C_ 156.8), suggested **3** to have a 6a,12a-dehydrorotenoid skeleton. Furthermore, typical for 6a,12a-dehydrorotenoids, the deshielded chemical shift of the singlet signal at δ_H_ 8.32 was assigned to H-1 of ring A.^40^ This is due to the magnetic anisotropy of the nearby carbonyl C-12 (δ_C_ 174.2) and the flat geometry of the molecule [(sp^2^-hybridized C-6a (δ_C_ 156.8) and C-12a (δ_C_ 112.3)]. The NMR data further indicated the presence of methoxy (δ_H_ 3.92, δ_C_ 56.0) and methylenedioxy (δ_H_ 5.96, δ_C_ 101.5) moieties. Here, the appearance of the two singlets H-1 (δ_H_ 8.32) and H-4 (δ_H_ 6.53) was indicative of the placement of the methylenedioxy moiety at C-2/C-3 of ring A, which was further supported by the HMBCs between the methylenedioxy protons (δ_H_ 5.96) and C-2 (δ_C_ 142.9) and C-3 (δ_C_ 147.4). The placement of the methoxy group at C-9 was established upon the presence of three mutually coupled protons, H-11 (δ_H_ 8.20, d, *J* = 8.9 Hz), H-10 (δ_H_ 7.0, dd, *J* = 2.2, 8.9 Hz), and H-8 (δ_H_ 6.83, d, *J* = 2.2 Hz), in ring D. This was further supported by the HMBCs of the OMe-9 (δ_H_ 3.92) to C-9 (δ_C_ 164.0), of H-11 (δ_H_ 8.20) to C-9 (δ_C_ 164.1) and C-12 (δ_C_174.2), and of H-8 (δ_H_ 6.83) to C-7a (δ_C_ 156.7) and C-11a (δ_C_ 118.7) as well as by the NOE between OMe-9 (δ_H_ 3.92) and H-8 (δ_H_ 6.83). Therefore, the new compound **3** was identified as 9-methoxy-2,3-methylenedioxy-6a-2a-dehydrorotenoid and was given the trivial name oblarotenoid G. It has previously been reported as a synthetic product.^41^ Here, we report it as a natural product and provide its complete NMR data for the first time. It is structurally close to the previously reported oblarotenoid A^18^ and may be obtained by its dehydration. Oblarotenoid G has been observed in the crude extract, by TLC profiling, confirming that it is not an isolation artifact.

Compound **4** was isolated as a white amorphous solid and was assigned the molecular formula C_45_H_54_O_25_ based on HRESIMS ([M + H]^+^*m*/*z* 959.3043) and NMR data (Table 2, Figures S25–S32, Supporting Information). The NMR spectra showed signals at δ_H_ 8.17 (s, H-2), δ_C_ 154.2 (C-2), 126.9 (C-3), and 177.1 (C-4) characteristic for an isoflavone^18^ substituted with a methylenedioxy (δ_H_ 5.98, δ_C_ 102.5), two methoxy (δ_H_ 3.91; δ_C_ 62.3 and δ_H_ 3.92; δ_C_ 62.8), and sugar moieties. Based on the HMBC of H-5 (δ_H_ 7.12) to C-4 (δ_C_ 177.1), C-8a (δ_C_ 155.5), and C-7 (δ_C_ 142.1), the single aromatic proton of the trisubstituted ring A was placed at C-5 (δ_C_ 101.8). Its positioning was confirmed by the HMBC of H-5 (δ_H_ 7.1) to C-4 (δ_C_ 177.1). The chemical shift values δ_C_ 62.8 and δ_C_ 62.3 of the two OMe groups indicated that both are di-*ortho-*disubstituted.^33^ They were placed at C-7 and C-8 based on the HMBC of 7-OMe (δ_H_ 3.92) and C-7 (δ_C_ 142.1) as well as between 8-OMe (δ_H_ 3.91) and C-8 (δ_C_ 153.9). The methylenedioxy moiety was placed at C-3′/C-4′ (δ_C_ 148.9) of ring B, which has an AMX-spin system with protons at δ_H_ 7.03 (d, *J* = 1.7 Hz, H-2′), δ_H_ 6.97 (dd, *J* = 1.6, 8.0 Hz, H-6′) and δ_H_ 6.84 (d, *J* = 8.0 Hz, H-5′). The isoflavone is also substituted with four glycosides as indicated by the characteristic signals of four anomeric protons at δ_H_ 5.08, 4.77, 4.73, and 4.55. Two of the sugars that possessed methyl groups at δ_H_/δ_C_ 1.14/17.9 and δ_H_/δ_C_ 1.09/17.8, respectively, were assigned as rhamnosyl, whereas the other two were assigned as glucosyl. The ^1^H and ^13^C NMR signals corresponding to each sugar (Table 2) were identified with the help of COSY, TOCSY, HSQC, HMBC, and NOESY spectra (Figures S26–S30, Supporting Information). Carbon C-6 of the isoflavone is the site of *O*-glycosidation, based on the NOE of H-5 (δ_H_ 7.13) and OMe-7 (δ_H_ 3.80) with the anomeric proton H-1″ (δ_H_ 5.05) of the first glucose as well as on the HMBC of H-1″ and C-6 (δ_C_ 157.0). The large coupling constant, ^3^*J*_H1″H2″_ = 7.9 Hz of the anomeric proton H-1″ (δ_H_ 5.05) to H-2″ (δ_H_ 3.78) suggested the β-linkage of the sugar to the isoflavone.^42^ The large diaxial coupling constants of the protons of this sugar moiety (Table 2) suggested it to be a β-glucose. Both H-1‴ (δ_H_ 4.53), the anomeric proton of the second sugar moiety, and H-2″ (δ_H_ 3.80) showed HMBC with C-4″ (δ_C_ 89.4), which together with the NOE of H-4″ (δ_H_ 3.61) and H-1‴ (δ_H_ 4.53) and with the ^3^*J*_H1‴H2‴_ = 7.8 Hz identified the β-glucopyranosyl-(4″**→**1‴)-β-glucopyranosyl linkage between the two glycoside moieties. The characteristic large diaxial coupling constants (Table 2) and the NOEs of H-1‴ (δ_H_ 4.53) with 1‴″ (δ_H_ 4.74), H-3‴′ (δ_H_ 3.70), and H-3‴″ (δ_H_ 3.80) indicated the second sugar moiety to also be a β-glucopyranosyl. The anomeric protons of the two rhamnosyl moieties, H-1‴′ (δ_H_ 4.71) and H-1‴″ (δ_H_ 4.74), showed HMBC with C-7″ (δ_C_ 67.8) and C-7‴ (δ_C_ 68.6), respectively. Furthermore, H-7″ (δ_H_ 4.08 and 3.58) and H-7‴ (δ_H_ 4.04 and 3.55) showed HMBC to the respective anomeric carbons of the rhamnosyl groups, C-1‴ (δ_C_ 102.1) and C-1‴″ (δ_C_ 102.4). These HMBCs along with the NOEs of H-7″ (δ_H_ 4.11 and 3.60) and H-7‴ (δ_H_ 4.06 and 3.58) to H-1‴′ (δ_H_ 4.71) and H-1‴″ (δ_H_ 4.74), respectively, established the α-rhamnosyl-(1‴′→7″)-β-glucosyl and the α-rhamnosyl-(1‴″→7‴)-β-glucosyl linkages. The equatorial orientation of H-1‴′ (δ_H_ 4.71) and H-1‴″ (δ_H_ 4.74) was assigned upon the observation of characteristic small, diequatorial ^3^*J*_H1‴′H2‴′_ = 1.2 Hz and ^3^*J*_H1‴″H2‴″_ = 1.4 Hz coupling constants and of the absence of NOEs between H-1‴″ (δ_H_ 4.74) and H-3‴″ (δ_H_ 3.80) and H-1‴″ (δ_H_ 4.74) and H-3‴″ (δ_H_ 4.80). The identity of the aglycone was further confirmed by comparison of its NMR data with that of its literature-known 6-methoxy derivative (Figures S33–S39).^43^ The absolute configuration of compound **4** has not been elucidated. However, natural sugars are known to almost always be d-glucose and l-rhamnose.^44,45^ Based on the above spectroscopic data, obloneside (**4**) was characterized as isoplatycarpanetin-6-*O*-β-glucosyl-(7″→1‴′)-α-rhamnosyl-(4″→1‴)-β-glucosyl-(7‴→1‴′)-α-rhamnoside.

The extracts and the isolated compounds were tested for antiviral activity against RSV by the viral plaque reduction assay in cultures of HEp-2 cells (Table 3, Supporting Information). A tetrazolium-based cytotoxicity assay on HEp-2 cells was also performed to exclude that the anti-RSV activity was a result of general cytotoxicity. The cytotoxicity assay was complemented by microscopy observation of the compound- and extract-treated cells to detect possible cytostatic activity, such as poor cell growth and/or altered cell shape that may affect antiviral activity. The leaf extract exhibited anti-RSV activity with an IC_50_ of 0.7 μg/mL and comparably low toxicity, CC_50_, of 50.0 μg/mL, for HEp-2 cells (Table S1 and Figures S129–S130, Supporting Information), whereas the root wood extract showed anti-RSV activity with an IC_50_ of 3.4 μg/mL. Both crude extracts decreased the cell viability also at concentrations less than their CC_50_ values (Figure S130, Supporting Information), suggesting that they are rich in cytotoxic/cytostatic compounds that may interfere with their anti-RSV activities (Figure S129, Supporting Information). Out of the 19 isolated compounds tested, **6** (IC_50_ 1.4 μM), **8** (IC_50_ 1.5 μM), **10** (IC_50_ 0.4 μM), **11** (IC_50_ 8.0 μM), and **14** (IC_50_ 10.0 μM) exhibited substantial anti-RSV activity and showed cytotoxicity (26.5 μM to >100.0 μM) and cytostatic activity only at much higher concentrations than their antiviral IC_50_ values (Table 3 and Figures S131–S132, Supporting Information). Compounds **9** (IC_50_ 0.8 μM) and **13** (IC_50_ 2.1 μM) showed substantial anti-RSV activity with selectivity indices (SI) of 15.6 and >47.6, respectively; however, both exhibited cytostatic activity at concentrations close to their IC_50_ values (Table 3). At this stage, it is difficult to assess whether the cytostatic activity of these specific compounds contributed to the protection of cells against virus infection, and accordingly the specificity of anti-RSV action of **9** and **13** requires further investigation. An attempt was made to address this issue by testing cytotoxic activities of the most promising compounds in human embryonic lung fibroblasts (HELFs), i.e., primary cells derived from lung interstitium. In these cells, the CC_50_ values were >100 μM for compounds **3**, **11**, and **14** and 47.1 μM for compound **15**, thus confirming the low cytotoxic activity of these compounds found in HEp-2 cells (Table 3), an observation that further strengthens their antiviral potential. In contrast the other active compounds showed CC_50_ values at low concentrations, i.e., 1.3 μM for **6**, 4.8 μM for **8**, 0.3 μM for **10**, and 9.0 μM for **13**, indicating that these compounds were more toxic for HELF cells than for HEp-2 cells, and the specificity of their anti-RSV activity requires further studies in HELF cells. The addition of the same volume of pure DMSO as the volume of the solution of the studied compounds was used as a negative control in this study, while ribavirin, the only antiviral drug approved in the form of aerosol formulations for the treatment of RSV disease, was used as a positive control. In our hands, ribavirin inhibited RSV infection of HEp-2 cells with an IC_50_ of 10.6 μM and was not toxic for cells at concentrations up to 100.0 μM (CC_50_ > 100.0 μM), but showed cytostatic activity at 100.0 μM (Table 3 and Figures S131 and S132, Supporting Information).

**Table 3:** Anti-RSV Activity, Cytotoxicity for HEp-2 Cells, and Selectivity Indices (SI) of Compounds Isolated from *Milletia oblata*

	anti-RSV activity	cytotoxicity		SI^b^
compound/extract	IC_50_ (μM)	CC_50_ (μM)	cytostatic activity^a^	(CC_50_/IC_50_)
**1**	>100.0	>100.0		1.0
**2**	>100.0	>100.0		1.0
**3**	22.0	90.0		4.1
**4**	>100.0	>100.0		1.0
**5**	53	>100.0		>1.9
**6**	1.4	50.0	PCS (20.0 μM)	35.7
**7**	1.8	6.7		3.8
**8**	1.5	42.0	PCS (20.0 μM)	28.0
**9**	0.8	12.5	PCS (0.8 and 4.0 μM)	15.6
**10**	0.4	26.5	PCS (4.0 μM)	61.6
**11**	8.0	91.0	PCS (20.0 μM)	11.4
**12**	>100.0	>100		1.0
**13**	2.1	>100.0	PCS (4.0, 20.0, and 100.0 μM)	>47.6
**14**	10.0	>100.0	PCS (100.0 μM)	>10
**15**	10.0	65.0	PCS (20.0 μM)	6.5
**16**	>100.0	>100.0		1.0
**17**	>100.0	>100.0		1.0
**18**	ND^c^	ND^c^	ND^c^	ND^c^
**19**	>0.8	2.0		<2.5
ribavirin	10.6	>100	PCP (100 μM)	>9.4
DMSO	>100	>100		1

a The CC_50_ assay was complemented by microscopic recording of possible cytostatic activity of test compounds manifested as poor cell proliferation (PCP), altered cell shape (ACS), or poor cell staining (PCS).

b SI of 1 indicates lack of antiviral activity, i.e., lack of cell protection against RSV; SI of 2–10 suggests that antiviral activity may at least in some compounds be unspecific due to cytostatic activity (poor cell proliferation/altered cell shape) that occurs at concentrations just below the CC_50_ and may not be detectable by the cell toxicity assay used; SI > 10 suggests specific antiviral activity for most compounds tested.

c ND, not determined

We also tested the crude extracts and the isolated compounds for their capability to inhibit HRV-2 infection of adenocarcinoma cells of the uterine cervix (HeLa). Compound **3** displayed anti-HRV-2 activity by protecting the cells at an IC_50_ of 4.2 μM while showing toxicity for HeLa cells first at a CC_50_ of 48.0 μM (SI 11.4, Figure S133, Supporting Information). Compound **3** also showed anti-RSV activity (IC_50_ 22.0 μM, Table 3 and Figures S131 and S132, Supporting Information). Our observations emphasize the antiviral potential of rotenoids, as some of these compounds were reported to inhibit the Newcastle disease virus and the herpes simplex virus (HSV),^46^ and 12a-hydroxyrotenoids showed modest anti-HSV activity.^47^ Isoflavonoids are believed to be produced by plants for protection against microbes, and they can therefore be expected to show activity against viruses.^48^

In conclusion, 19 natural products, including three new roteinoids (**1**–**3**) and a new glycosylated isoflavone (**4**), were isolated from the CH_2_Cl_2_/MeOH (1:1) extract of the leaves and the root wood of *M. oblata* ssp. *teitensis.* The absolute configuration of rotenoids was for the first time determined by VCD spectroscopy. In addition to the crude extracts (IC_50_ 0.7 and 3.4 μg/mL), the isolated compounds oblarotenoid A (**6**), 12a-hydroxymunduserone (**8**), deguelin (**10**), ichthynone (**11**), and 4′-prenyloxyderrone (**14**) showed anti-RSV activity (IC_50_ 0.4–10.0 μM) with low cytotoxicities in HEp-2 cells (>26.5 μM). Tephrosin (**9**) and isoerythrin-A-4′-prenyl ether (**13**) showed substantial anti-RSV activity (IC_50_ 0.8 and 2.1 μM, respectively), yet also exhibited cytostatic activity (CC_50_ 12.5 and >100.0 μM). Besides its anti-RSV activity (IC_50_ 22.0 μM), 9-methoxy-2,3-methylenedioxy-6a-2a-dehydrorotenoid (**3**) protected cells from HRV-2 infection (IC_50_ of 4.2 μM) whereas showing low cytotoxicity (CC_50_ of 48.0 μM).

## Experimental Section

### General Experimental Procedures

Optical rotations were measured on a PerkinElmer 341-LC instrument. NMR spectra were acquired on an Agilent MR-400-DD2 spectrometer equipped with a 5 mm OneNMR probe and on a Bruker Avance NEO 500 MHz or Bruker Avance NEO 800 MHz instrument equipped with a 5 mm cryogenic TXO probe. The spectra were processed using MestReNova 14.1 software and were referenced to the residual solvent peak. LC-ESIMS data were acquired on a Waters Micromass ZQ multimode ionization electrospray ionization (ESI) instrument connected to an Agilent 1100 series gradient pump system and a C_8_ column (Gemini), using a Milli-Q H_2_O/MeCN (5:95 to 95:5, with 1% HCO_2_H over 4 min) eluent mixture. HRESIMS spectra were acquired with a Q-TOF LC/MS spectrometer with a lock mass-ESI source (Stenhagen Analysis Lab AB, Gothenburg, Sweden), using a 2.1 × 30 mm, 1.1 μm reverse phase^49^-C_18_ column and a H_2_O/MeCN gradient (5:95 to 95:5, with 0.2% HCO_2_H). TLC analyses were carried out on Merck precoated silica gel 60 F_254_ plates. Preparative TLCs were performed on glass plates of 20 × 20 cm dimension, precoated with silica gel 60F_254_ having 0.25 to 1 mm thickness. Column chromatography was performed on silica gel (40–63 μm mesh), and gel filtration was performed on Sephadex LH-20.

### Plant Materials

The root wood and leaves of *Millettia oblata* ssp. *teitensis* were collected in July 2014 from Ngaongao forest, Taita Hill, Taita County, Kenya, and were assigned voucher number TD-04/2014. The plant was identified by Mr. Patrick Chalo Mutiso of the Department of Biology, University of Nairobi, Kenya, where the specimen was deposited.

### Extraction and Isolation

The dried and ground leaves of *M. oblata* (795 g) were extracted with CH_2_Cl_2_/MeOH (1:1) (4 × 1.5 l) for 24 h by cold percolation. The extract was filtered, and the supernatant was concentrated under reduced pressure to obtain the extract (35 g). A portion of this crude extract (27 g) was subjected to column chromatography on silica gel (300 g) using *iso*-hexane (a mixture of isomeric branched chain hexanes) containing increasing amounts of EtOAc to give a total of 100 fractions. The first 30 fractions eluted with 0–3% EtOAc in *iso*-hexane were not further investigated. Fractions 31–40 eluted with 6% EtOAc in *iso*-hexane were combined, based on their TLC profile, and were subjected to column chromatography on Sephadex LH-20 (CH_2_Cl_2_/MeOH, 1:1) to give oblarotenoid C (**5**, 8 mg) and 9-methoxy-2,3-methylendioxy-6a-12a-dehyrorotenoid (**3**, 10 mg). Fractions 41–48 eluted at 7% EtOAc in *iso*-hexane afforded a white precipitate, which was washed with MeOH to afford oblarotenoid A (**6**, 11 mg). Fractions 49–57 eluted with 8% EtOAc in *iso*-hexane showed the presence of five compounds by TLC analysis and were separated by column chromatography on silica gel (eluent: *iso*-hexane/CH_2_Cl_2_, 1:1) to yield an additional amount of oblarotenoid A (**6**, 2 mg) together with 12a-hydroxymunduserone (**8**, 4 mg), tephrosin (**9**, 7 mg), deguelin (**10**, 10 mg), and a mixture, which was further separated by column chromatography over silica gel (eluent; *iso*-hexane/CH_2_Cl_2_/EtOAc, 60:35:10) to afford deguelin (**10**, 2 mg) and (6aS,12aS)-4,9-dimethoxy-2,3-methylenedioxyrotenoid (**1**, 3 mg). The fractions eluted with 10–14% EtOAc gave a mixture of four compounds, which was further purified on Sephadex LH-20 eluting with CH_2_Cl_2_/MeOH (1:1) to yield 7,2′,5′-trimethoxy-3′,4′-methylenedioxyisoflavone (**12**, 10 mg), oblarotenoid D (**7**, 8 mg), and a fraction containing three compounds. These three compounds were separated by preparative TLC developed in a mixture of *iso*-hexane/CH_2_Cl_2_/EtOAc (13:5:2) to yield ichthynone (**11**, 4 mg), oblarotenoid D (**7**, 1 mg), and (6a*R*,12a*R*)-9,10-dimethoxy-2,3-methylenedioxyrotenoid (**2**, 4 mg).

The root wood (800 g) was extracted to give a light brown crude extract (30 g) and was subjected to column chromatography over silica (300 g), eluting it as described above. The fractions eluted with 4–8% EtOAc in *iso*-hexane afforded isoerythrin-A-4′ (**13**, 7 mg), 4′-prenyloxyderrone (**14**, 15 mg), and a mixture of two compounds, which were separated on Sephadex LH-20 (CH_2_Cl_2_/MeOH, 1:1) to give cuneatin methyl ether (**15**, 6 mg) and calopogonium isoflavone B (**16**, 10 mg). The fractions eluted with 10% EtOAc were further purified on Sephadex LH20 to yield isobutyl alcohol (**19**, 11 mg). Fractions eluted with 12–18% EtOAc in *iso*-hexane afforded a mixture of two compounds, which upon purification by column chromatography over silica gel (*iso*-hexane/CH_2_Cl_2_, 1:1) yielded maximaisoflavone G (**17**, 9 mg) and milldurone (**18**, 8 mg). The fractions eluted with 100% EtOAc were further purified by Sephadex LH-20 (100% MeOH) chromatography and yielded obloneside (**4**, 15 mg).

#### (6a*S*,12a*S*)-2,9-Dimethoxy-3,4-methylenedioxyrotenoid (**1**)

White amorphous solid; [α]^20^_D_ +44.0 (*c* 0.2 CH_3_OH); UV λ_max_ (log ϵ) 280 nm (4.2) and 305 nm (4.0); ^1^H and ^13^C NMR, see Table 1 and Figures S1–S9, Supporting Information; HRESIMS *m*/*z* 357.0976 [M + H]^+^ (calcd 357.0974 for C_19_H_17_O_7_).

#### (6a*R*,12a*R*)-9,10-Dimethoxy-2,3-methylenedioxyrotenoid (**2**)

White amorphous solid, [α]^20^_D_ +42.0 (*c* 0.3 CH_3_OH); UV λ_max_ (log ϵ) 285 nm (4.3) and 305 nm (3.9); ^1^H and ^13^C NMR, see Table 1 and Figures S10–S17, Supporting Information; HRESIMS *m*/*z* 355.0354 [M + H – H_2_O]^+^ (calcd for C_19_H_15_O_7_ 355.0815), LC-ESIMS *m*/*z* 373.2 [M + 1]^+^ (calcd 373.1 for C_19_H_18_O_9_).

#### 9-Methoxy-2,3-methylenedioxy-6a,2a-dehydrorotenoid (**3**)

White amorphous solid; UV λ_max_ (log ϵ) 237 nm (4.5), 278 nm (2.3), and 310 nm (4.0); ^1^H and ^13^C NMR, see Table 1 and Figures S18–S24, Supporting Information; HRESIMS *m*/*z* 325.074 [M + H]^+^ (calcd 325.0712 for C_18_H_13_O_6_).

#### Obloneside (**4**)

Light yellow amorphous solid; UV λ_max_ (log ϵ) 290 nm (4.3); ^1^H and ^13^C NMR, see Table 2 and Figures S25–S32, Supporting Information; HRESIMS *m*/*z* 959.3043 [M + H]^+^ (calcd 959.3033 for C_42_H_55_O_25_).

### Cells and Viruses

HEp-2 cells were cultivated in Dulbecco’s modified Eagle’s medium (DMEM) supplemented with 8% fetal calf serum and 292 μg/mL of l-glutamine. Human adenocarcinoma cells of uterine cervix (HeLa) were grown in Eagle’s minimum essential medium supplemented with 5% fetal calf serum and antibiotics. HELFs were grown in Eagle’s minimum essential medium supplemented with 5% fetal calf serum, l-glutamine, and antibiotics. The A2 strain of RSV (ATCC VR-1540) was used. The RSV stock was prepared and stored as described previously by Lundin et al.^50^ In some experiments, strain HGP of HRV-2 (ATCC, VR-482) was used.

### Antiviral Assays

The RSV plaque reduction assay was performed as described previously^27,51^ with some modifications. Briefly, the test extracts and compounds were serially diluted at a range of 1.6–200 μg/mL and 0.16–100 μM, respectively, in DMEM supplemented with 2% heat-inactivated fetal calf serum, 60 μg/mL of penicillin, 100 μg/mL of streptomycin, and 292 μg/mL of l-glutamine (DMEM-M) and then added to one-day-old cultures of HEp-2 cell in 24-well culture plates. Following 15 min of incubation at 37 °C in a humidified atmosphere comprising 5% CO_2_ (incubator), the cells were inoculated with 50–100 plaque-forming units of RSV A2. After incubation of the virus–compound mixture with cells for 2.5 h in the CO_2_ incubator, the inoculum was removed and 750 μL of 1% methylcellulose solution in DMEM-M comprising the same concentration of test compound/extract was added and left for a further 3 days in the CO_2_ incubator. The cells were stained with a 1% solution of crystal violet, and the viral plaques were counted under a microscope. Ribavirin, a drug approved for the treatment of RSV disease, was used as a positive control, whereas DMSO at concentrations corresponding to those present in the test compounds was used as solvent control.

The anti-HRV-2 activity of extracts and compounds was assessed by a tetrazolium-based colorimetric assay^52^ with some modifications. Briefly, serial 5-fold dilutions of test samples (0.16–100 μM) in EMEM supplemented with 2% fetal calf serum, 1% pest stock, 1% l-glutamine stock, 30 mM MgCl_2_, and 20 mM HEPES (pH 7.1) (EMEM-M) were added to one-day-old cultures of HeLa cells seeded in 96-well culture plates. Following the incubation of cells with test samples for 3 h in a 34 °C CO_2_ incubator, 100 tissue culture infectious doses (TCID_50_) in EMEM-M were added. In some wells addition of the test samples and the virus was omitted to serve as the virus control and uninfected cell control, respectively. After incubation of the test plates in the CO_2_ incubator for 3 days, the CellTiter 96 Aqueous One solution reagent (Promega, Madison, WI, USA) was added, and following further incubation of plates in the CO_2_ incubator for 1 h, the absorbance was recorded at 490 nm. The % of the test sample protection of cells against HRV-2 infection was calculated as (absorbance of the test sample – virus control) × 100/absorbance of the cell control – virus control.

### The Cell Toxicity Assay

The tetrazolium-based cytotoxicity assay using a CellTiter 96 Aqueous One solution reagent (Promega) was performed for the test extracts and isolated compounds in HEp-2, HELFs, and HeLa cells as described previously.^50^

### Optical Spectroscopy

UV absorbance and ECD spectra were collected simultaneously on a 0.02 mg/mL sample in MeOH using a path length of 10 mm on a ChiraScan-Plus instrument (Applied Photophysics) with a scan speed of 30 nm/min under a continuous N_2_ flow. The solvent spectra were recorded under identical conditions to remove solvent bands in the UV spectra and to baseline correct the ECD spectra. IR and VCD spectra were recorded simultaneously on a ChiralIR-2X (Biotools) equipped with a dual PEM system running at a resolution of 4 cm^–1^ and the central PEM frequency set to 1400 cm^–1^. Samples were dissolved in CDCl_3_, in a cell equipped with BaF_2_ windows and a path length of 100 μm, and a total of 96 000 scans were recorded (32 h). The final experimental VCD spectra were obtained by subtracting the solvent VCD spectra recorded under identical conditions.

### Calculations

A low-energy conformation library of postulated compounds **1** and **2** in both the *cis* and *trans* configuration was created using PCModel^53^ with the incorporated MMFF94 force field. All conformers within a cutoff of 5 kcal/mol from the lowest energy conformer were retained and subjected to DFT optimization and spectral calculations at the B3LYP/6-311++G(d,p) level of theory. DFT calculations were performed on conformers exhibiting a Boltzmann weight higher than 1% (based on the enthalpy). The solvent was implicitly taken into account using the IEFPCM model, as implemented in the Gaussian suite. All DFT level calculations were performed using the Gaussian 16 software package^54^ with tight convergence criteria and ultrafine integration grids. For each conformer, IR absorbance and VCD spectra were created by applying a Lorentzian broadening with full width at half-maximum of 10 cm^–1^ and subsequent Boltzmann-weighted based on their enthalpies. To compare the calculated spectra with the experimental one, a spectra scaling factor of 0.98 was applied on the calculated frequencies.

## Data Availability

The original FIDs and MestreNova files for all compounds, NMReDATA^55,56^ files, and CSEARCH^30^ results for the new compounds **1**–**4**, original UV and IR spectra, and DFT-computed conformers for compounds **1** and **2** are freely available on Zenodo (DOI: 10.5281/zenodo.10846050).

## Supporting Information Available

NMR, MS, and optical spectroscopy data for the isolated compounds and antiviral activity and cytotoxicity data (PDF)
