# Supplementary material for: Antiviral Rotenoids and Isoflavones Isolated from Millettiaoblata ssp. teitensis
Source: J Nat Prod. 2024 Apr 5;87(4):1003–12. doi: 10.1021/acs.jnatprod.3c01288 (PMC11061832; doi:10.1021/acs.jnatprod.3c01288)
Supplement: Supplementary file 1 — np3c01288_si_001.pdf [file np3c01288_si_001.pdf]

SUPPORTING INFORMATION

Antiviral Rotenoids and Isoflavones Isolated from *Millettia oblata* ssp. *teitensis*

Ivan Kiganda, Jonathan Bogaerts, Lianne, H. E. Wieske, Tsegaye Deyou, Yoseph Atilaw, Colores Uwamariya, Masum Miah, Joanna Said, Albert Ndakala, Hoseah M. Akala, Wouter Herrebout, Edward Trybala, Tomas Bergström, Abiy Yenesew and Mate Erdelyi

Table of Contents

Natural products isolated from the leaves and roots of *Milletia oblata ssp. teitensis* S2

Spectroscopic Data of Oblarotenoid E (**1**).. S3

Spectroscopic Data of Oblarotenoid F (**2**)... S7

Spectroscopic Data of Oblarotenoid G (**3**) S12

Spectroscopic Data of Obloneside (**4**)... S15

Spectroscopic Data of 6-Hydroxy-7,8-dimethoxy-3',4'-methylenedioxyisoflavone (**4a**).................... S19

Spectroscopic Data of Oblarotenoid C (**5**) S23

Spectroscopic Data of Oblarotenoid A (**6**) S26

Spectroscopic Data of Oblarotenoid D (**7**) S29

Spectroscopic Data of 12a-Hydroxymunduserone (**8**) S32

Spectroscopic Data of Tephrosin (**9**)... S35

Spectroscopic Data of Deguelin (**10**) S38

Spectroscopic Data of Ichthynone (**11**) S41

Spectroscopic Data of 7,2',3'-Trimethoxyl-3',4'-Methoxylenedioxyisoflavone (**12**).......................... S44

Spectroscopic Data of Isoerythrin-A-4'-prenylether (**13**)... S47

Spectroscopic Data of 4'-Prenyloxyderone (**14**).. S50

Spectroscopic Data of Cuneatin Methyl Ether (**15**)... S53

Spectroscopic Data of Calopogonium Isoflavone B (**16**)... S56

Spectroscopic Data of Maximaisoflavone G (**17**) S59

Spectroscopic Data of Milldurone (**18**) S61

Spectroscopic Data of Isobava Chromene (**19**) S64

Antiviral Activity and Cytotoxicity.. S67

Optical Spectroscopy.. S70

Experimental vs Calculated IR for Oblarotenoid E (1) S70

IR spectrum for Oblarotenoid F (2).. S71

Natural products isolated from the leaves and roots of *Milletia oblata ssp. teitensis*

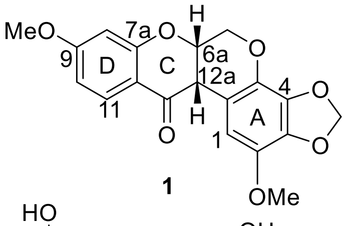

1

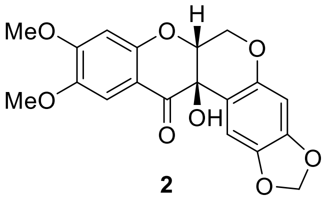

2

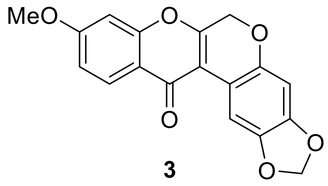

3

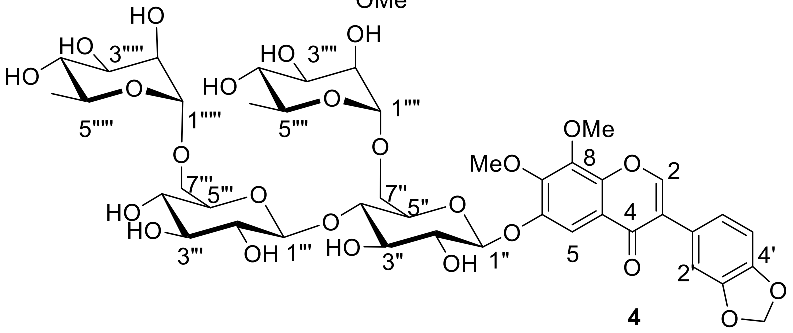

4

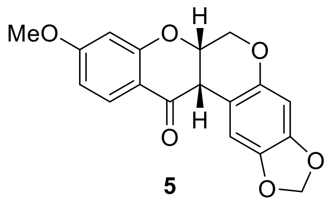

5

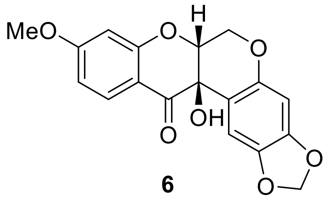

6

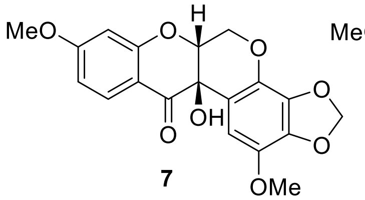

7

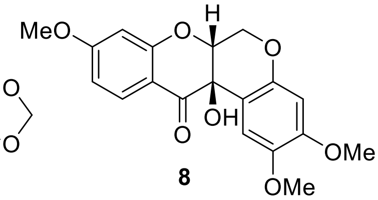

8

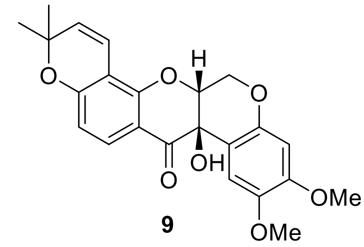

9

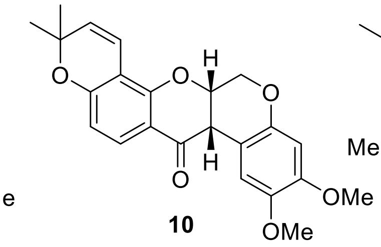

10

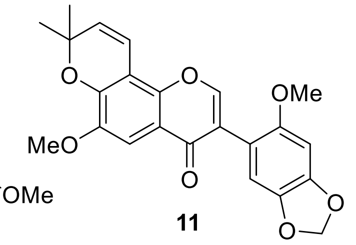

11

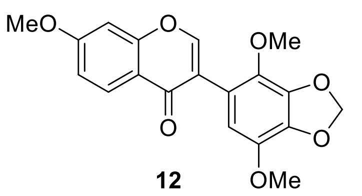

12

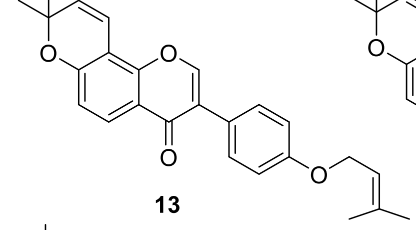

13

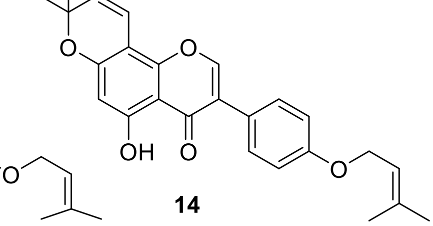

14

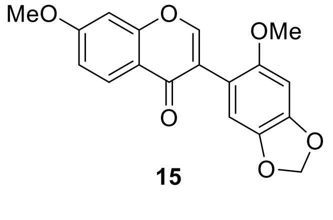

15

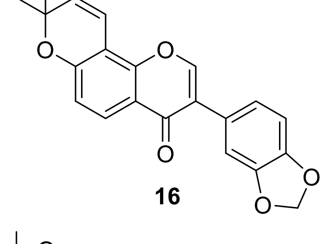

16

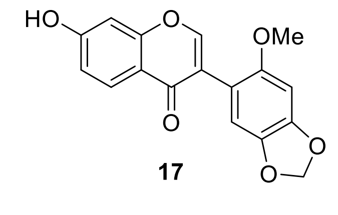

17

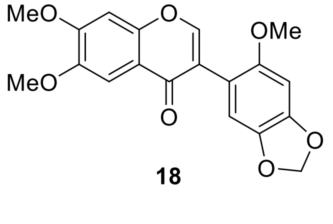

18

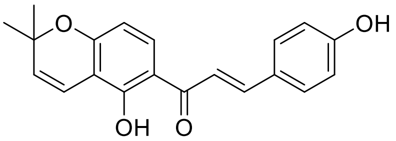

19

Spectroscopic Data of Oblarotenoid E (1)

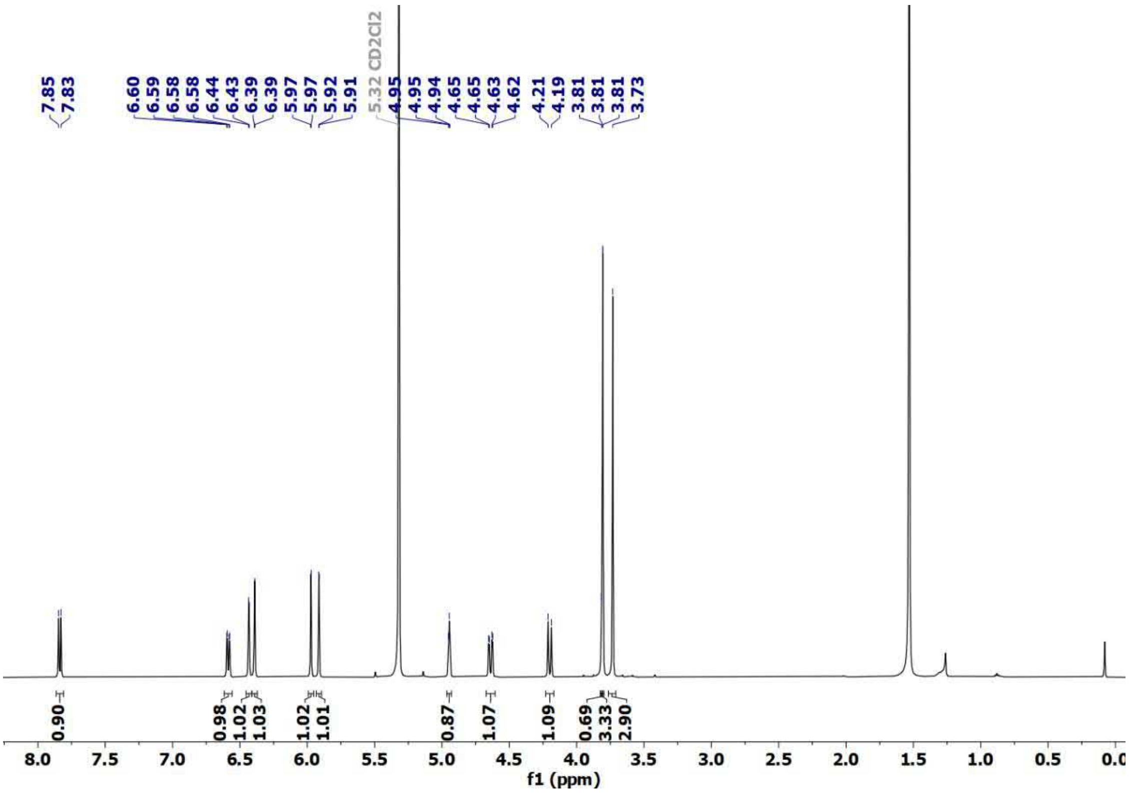

Figure S1. ^{1}H NMR (500 MHz, CD_2Cl_2, 25 ºC) spectrum of oblarotenoid E (**1**).

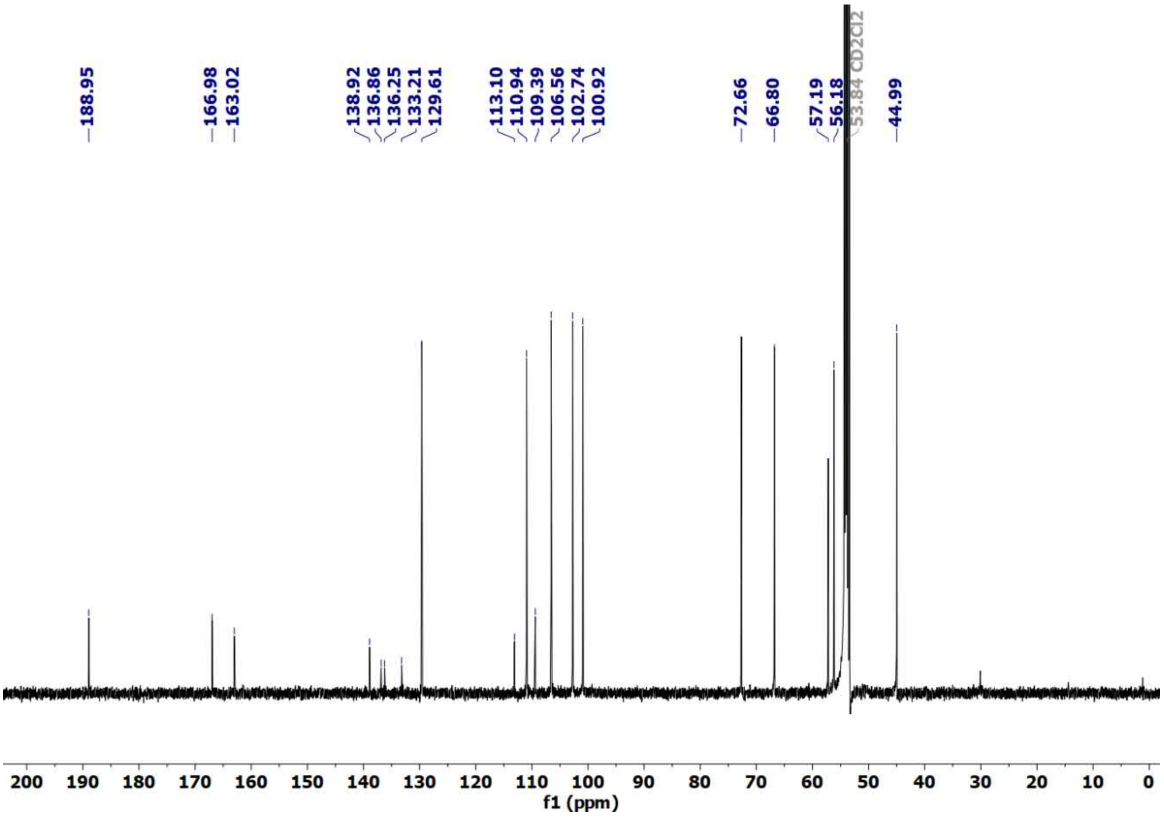

Figure S2. ^{13}C NMR (125 MHz, CD_2Cl_2, 25 ºC) spectrum of oblarotenoid E (**1**).

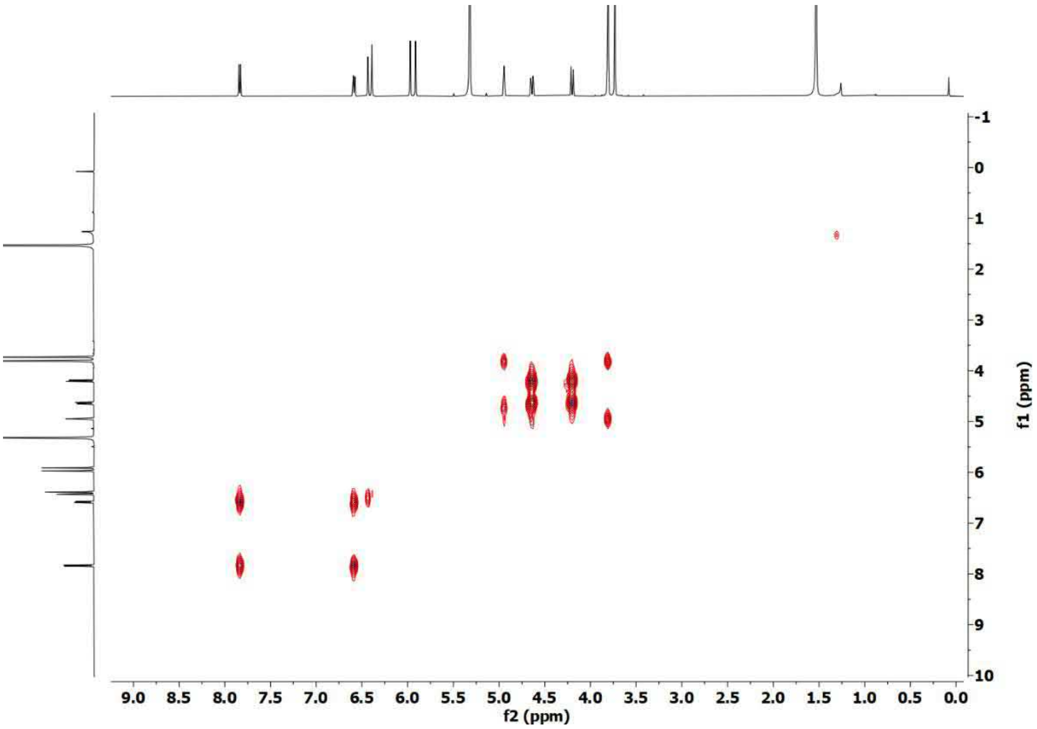

Figure S3. COSY (500 MHz, CD_2Cl_2, 25 ºC) spectrum of oblarotenoid E (**1**).

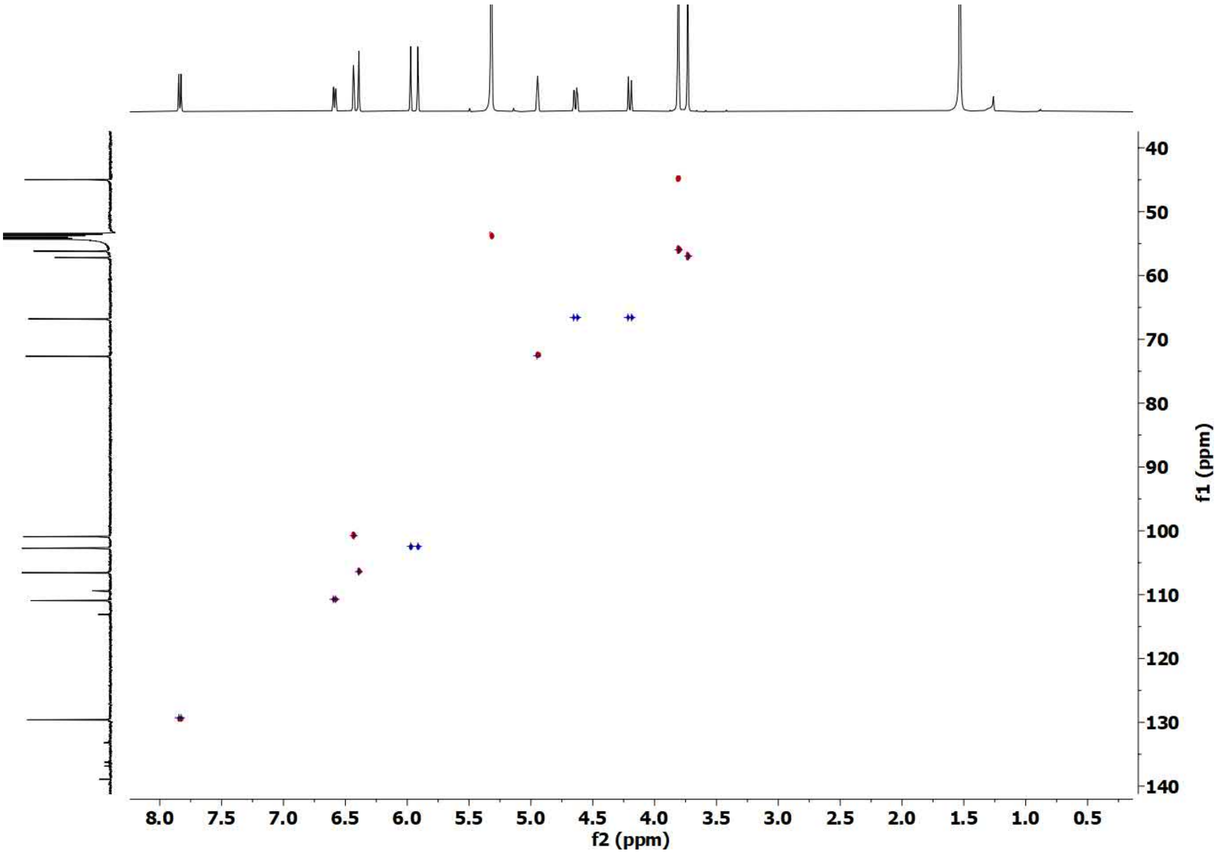

Figure S4. HSQC (500/125 MHz, CD_2Cl_2, 25 ºC) spectrum of oblarotenoid E (**1**).

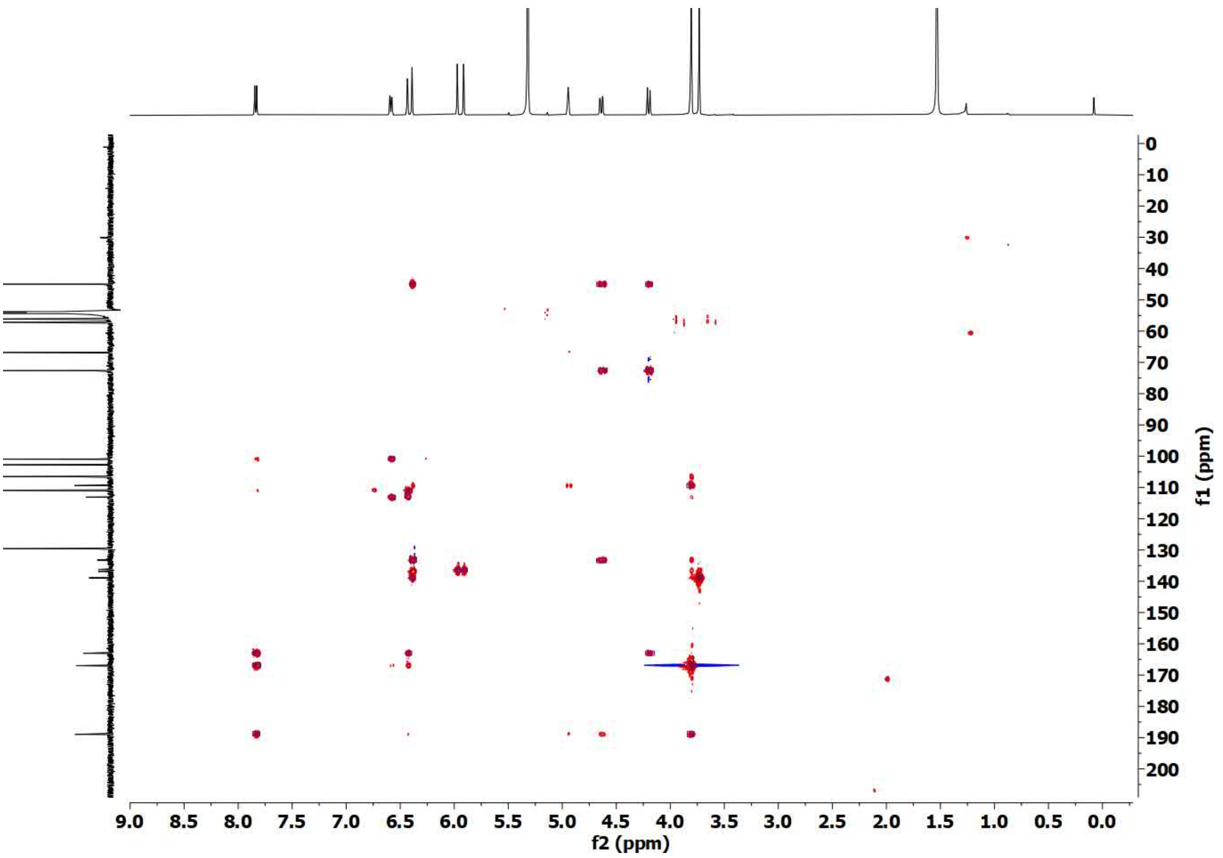

Figure S5. HMBC (500/125 MHz, CD_2Cl_2, 25 ºC) spectrum of oblarotenoid E (**1**).

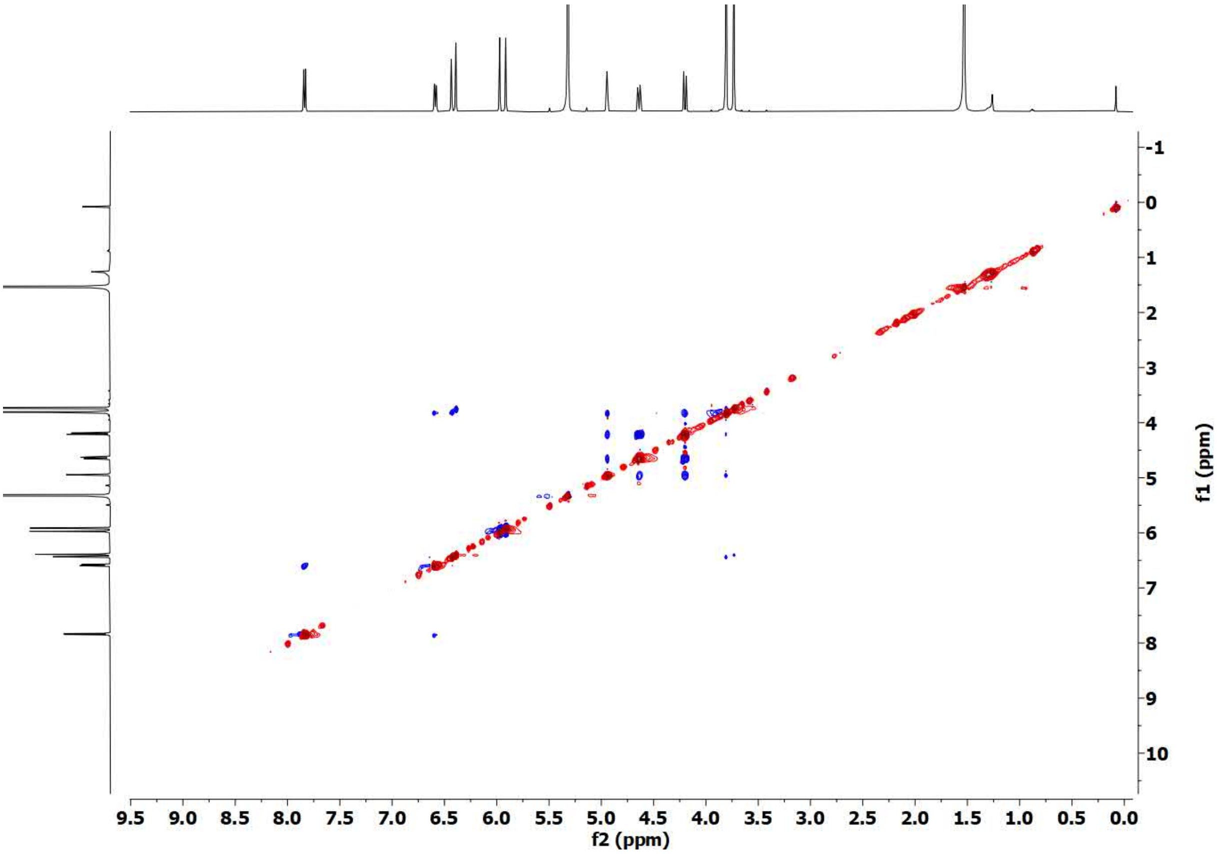

Figure S6. NOESY (500 MHz, CD_2Cl_2, 25 ºC) spectrum of oblarotenoid E (**1**).

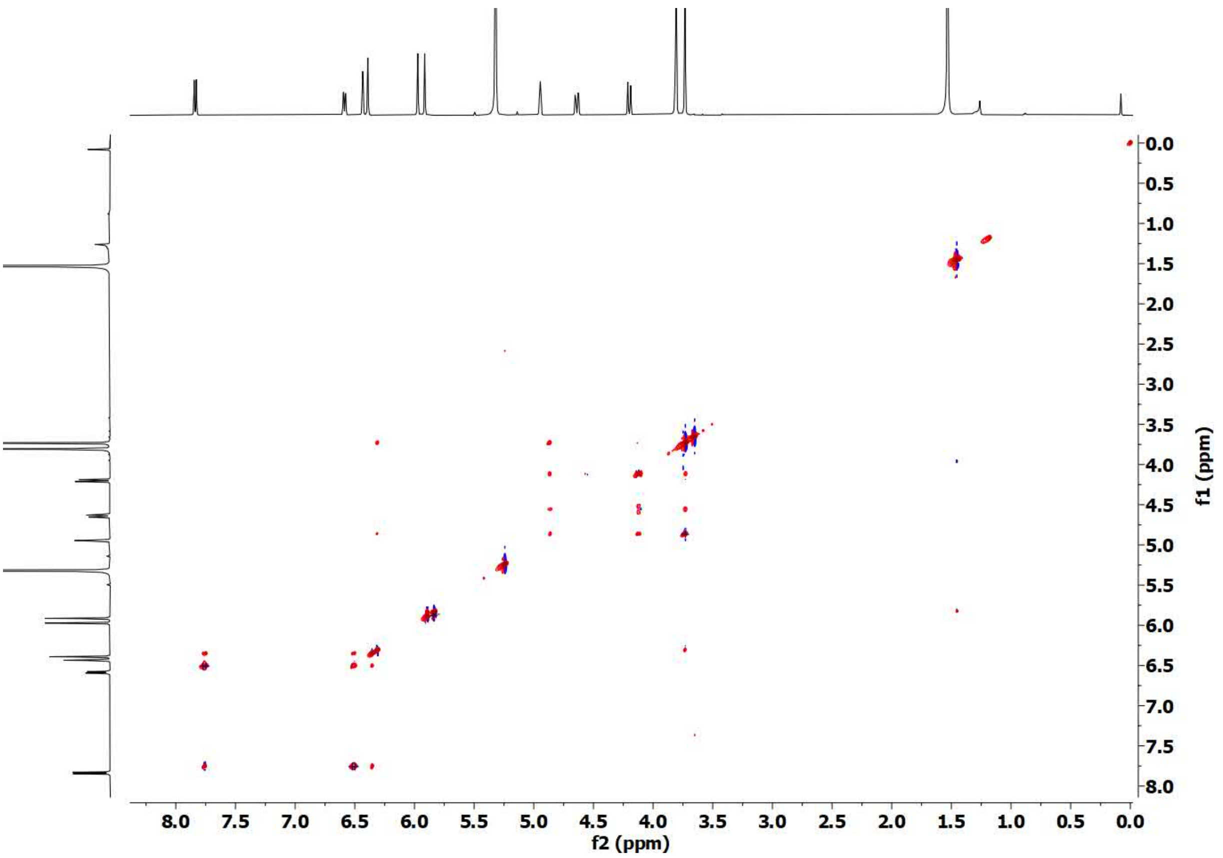

Figure S7. TOCSY (500 MHz, CD_2Cl_2, 25 ºC) spectrum of oblarotenoid E (**1**).

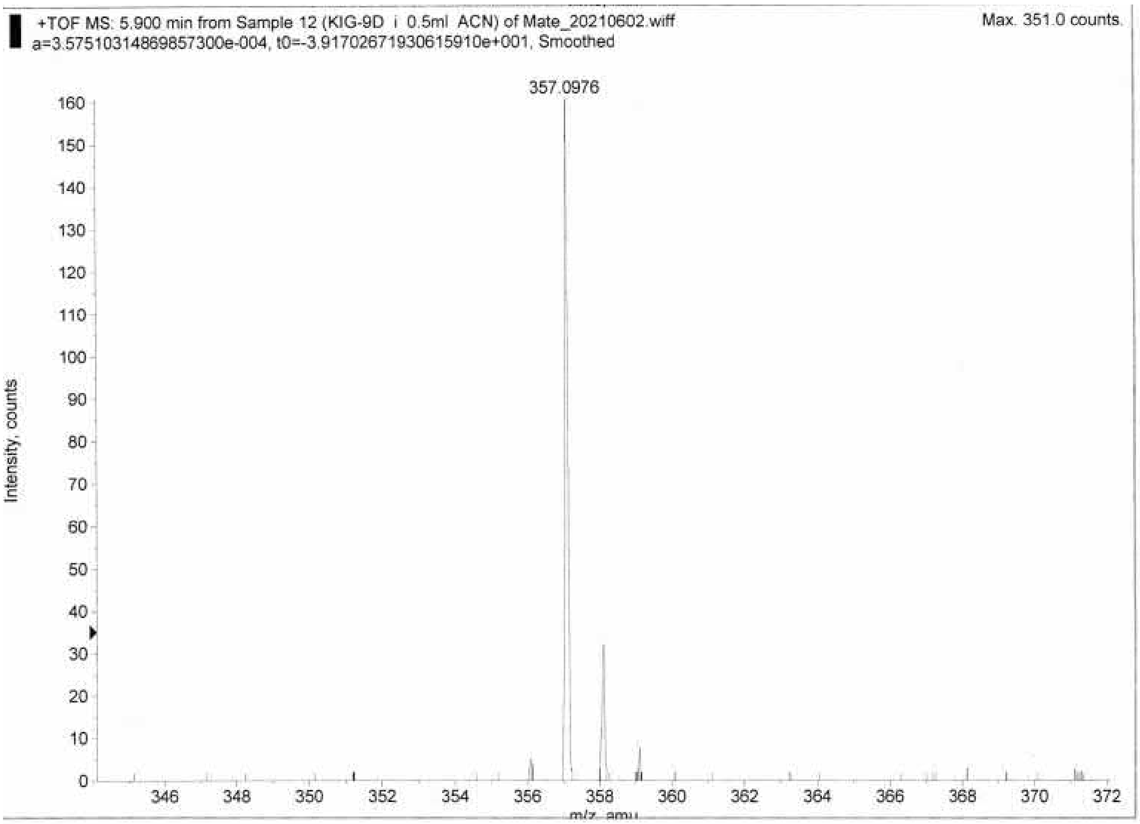

Figure S8. HRESIMS spectrum of oblarotenoid E (**1**).

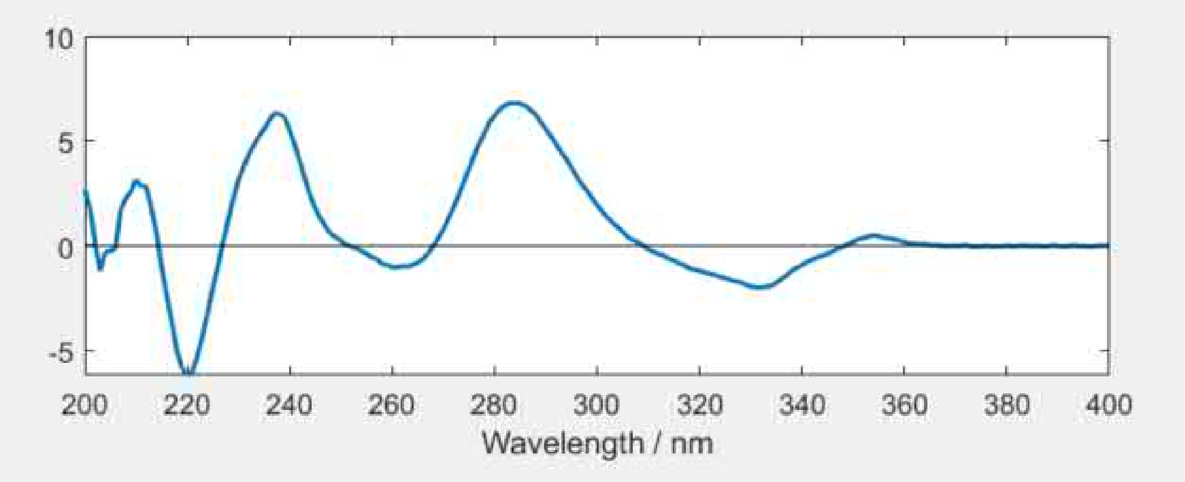

Figure S9. The CD spectrum of oblarotenoid E (**1**)

Spectroscopic Data of Oblarotenoid F (2).

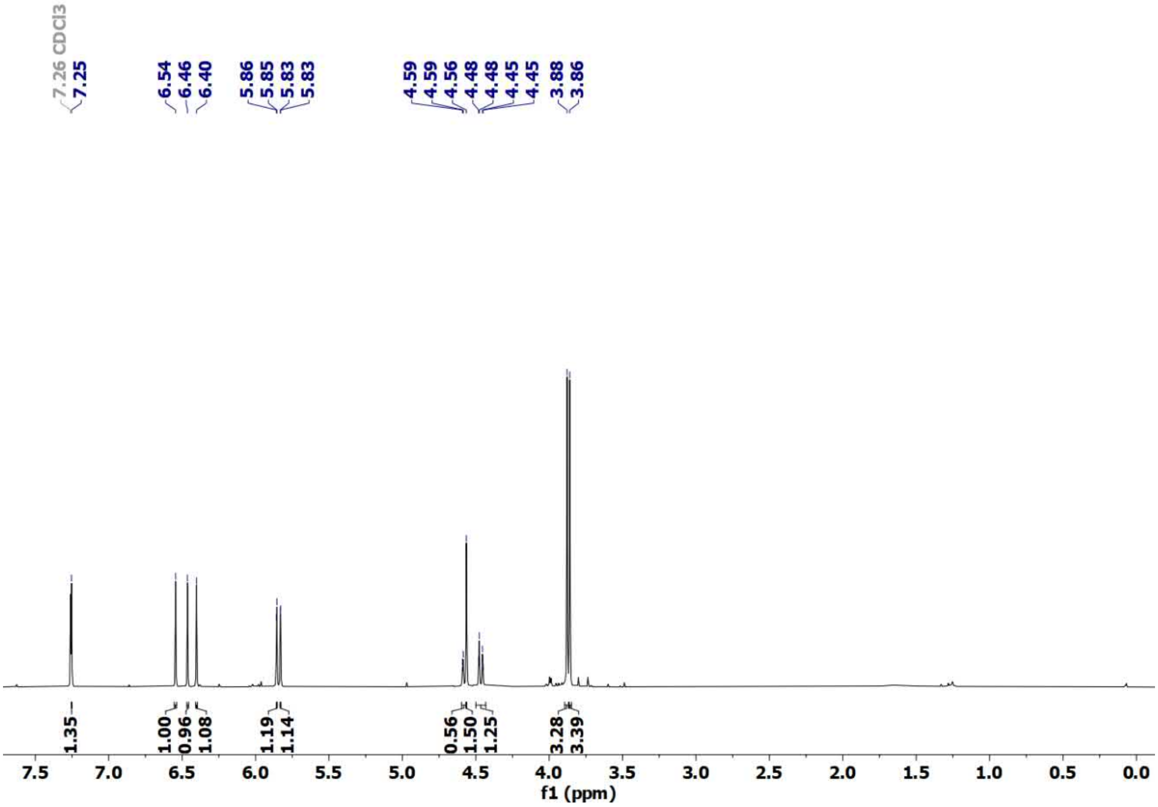

Figure S10. ^{1}H NMR (500 MHz, $CDCl_3$, 25 ºC) spectrum of oblarotenoid F (**2**).

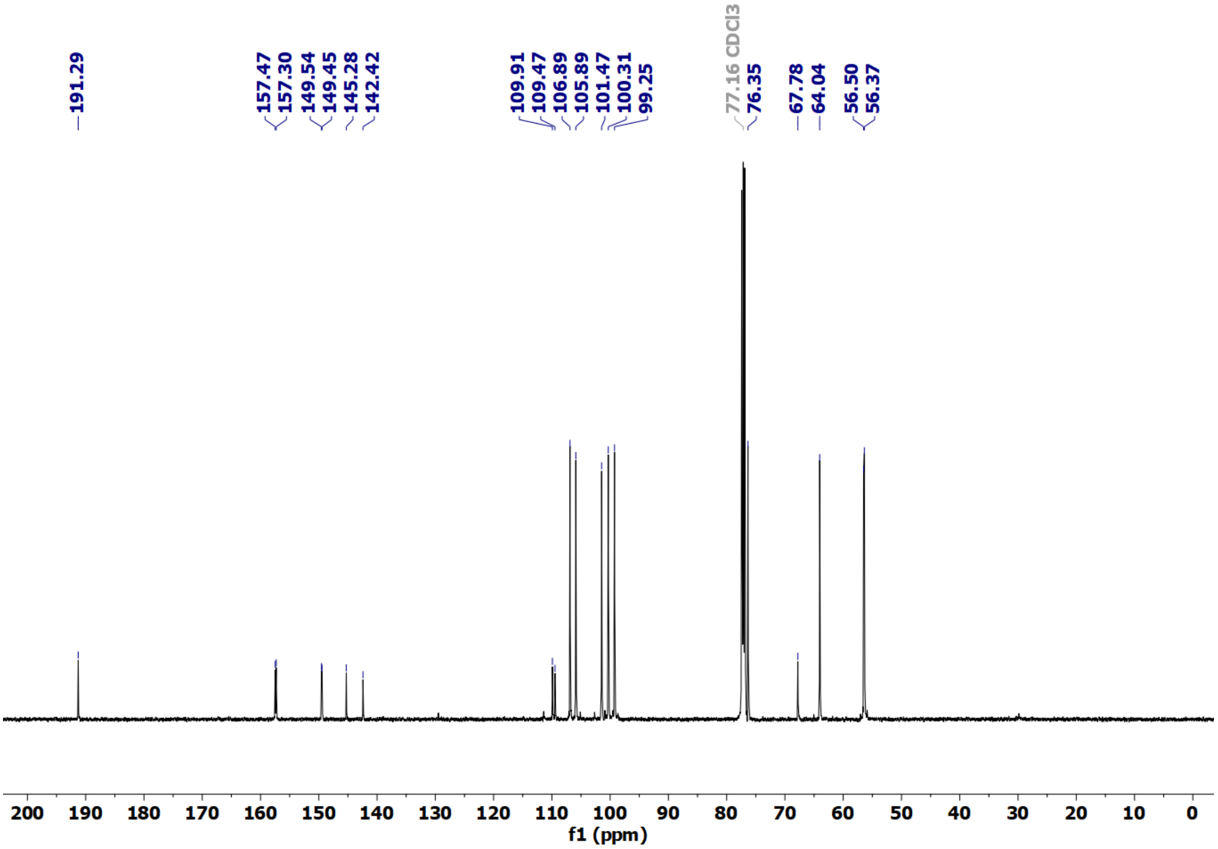

Figure S11. ^{13}C NMR (125 MHz, $CDCl_3$, 25 ºC) spectrum of oblarotenoid F (**2**).

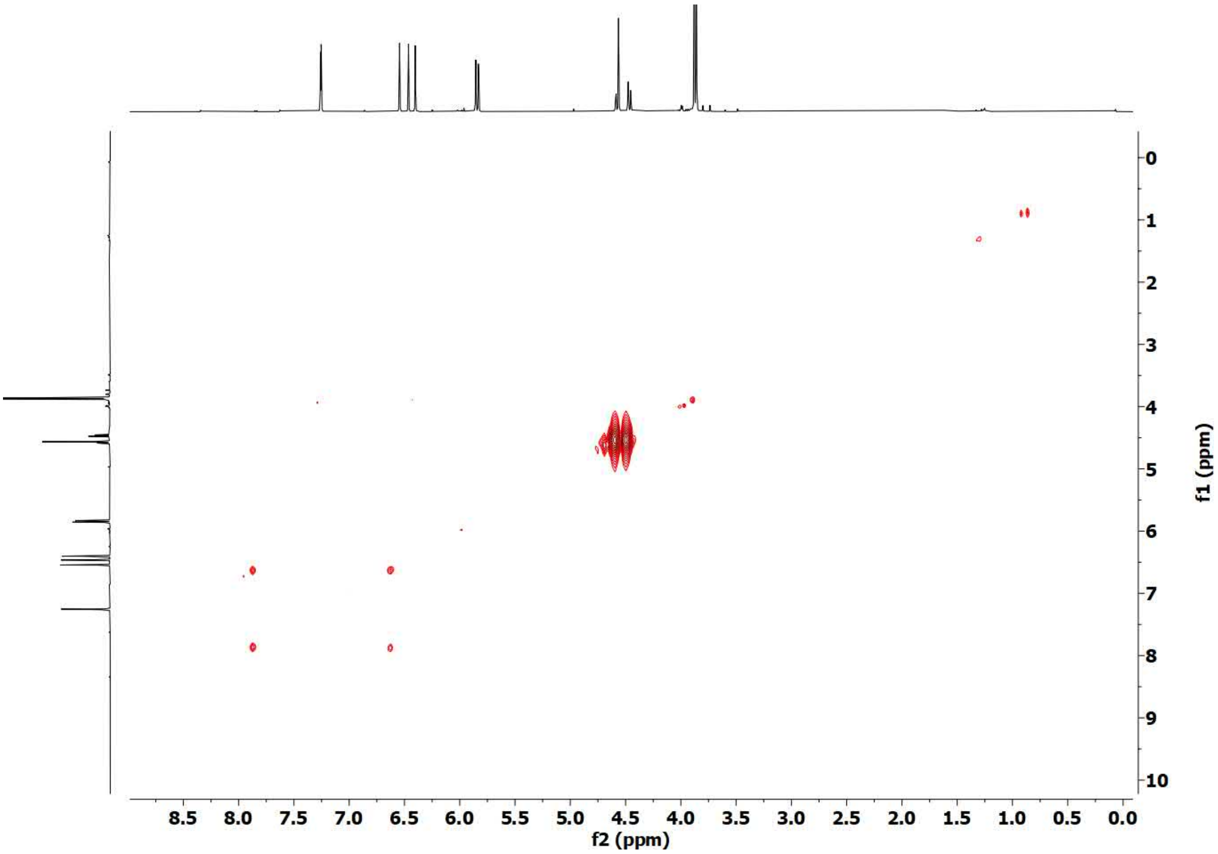

Figure S12. COSY (500 MHz, $CDCl_3$, 25 ºC) spectrum of oblarotenoid F (**2**)

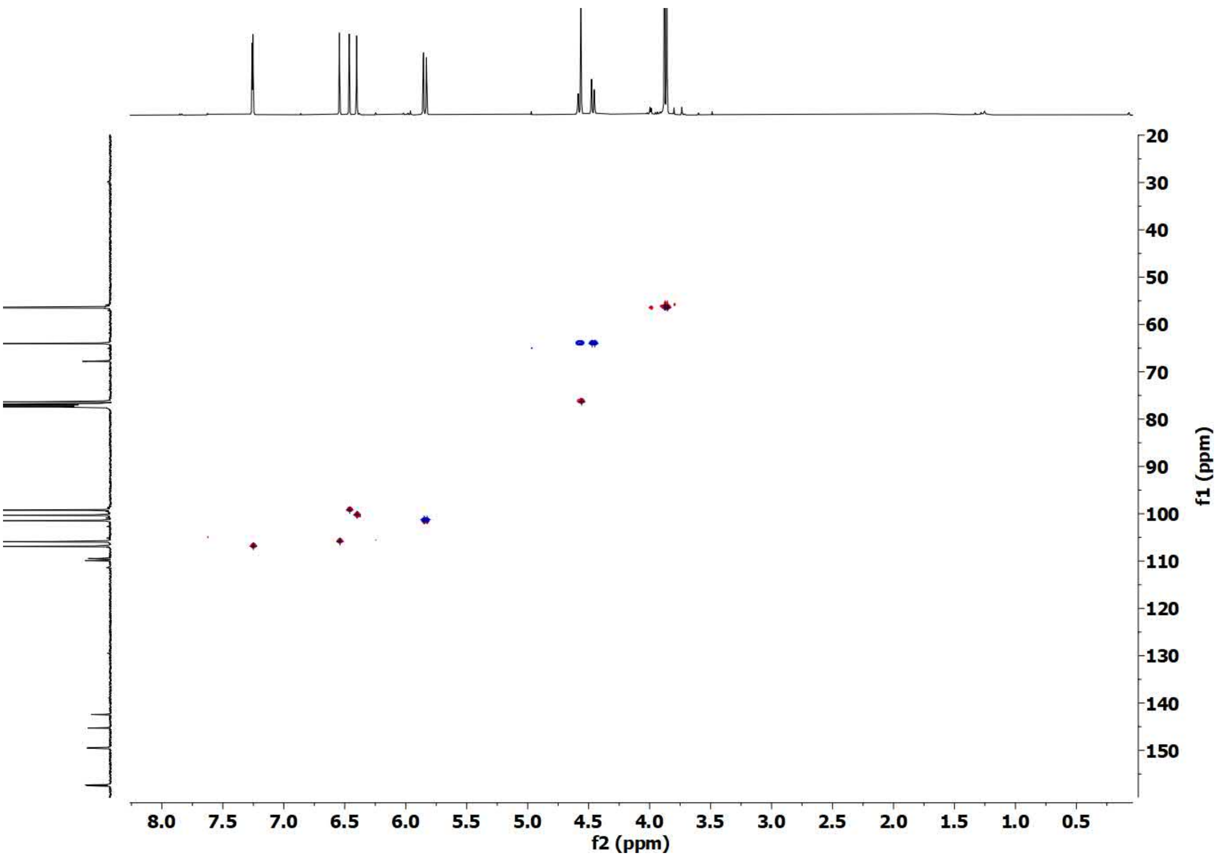

Figure S13. HSQC (500/125 MHz, $CDCl_3$, 25 ºC) spectrum of oblarotenoid F (**2**)

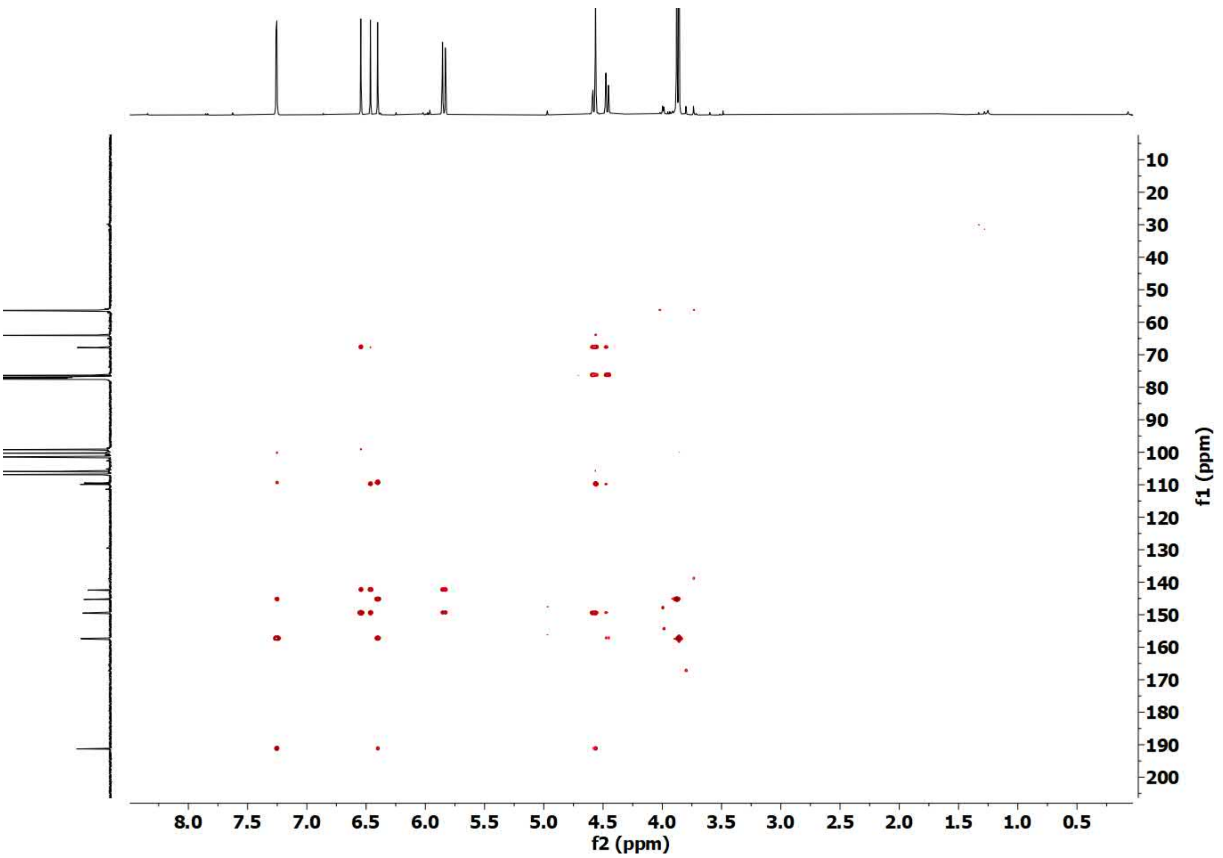

Figure S14. HMBC (500/125 MHz, $CDCl_3$, 25 ºC) spectrum of oblarotenoid F (**2**)

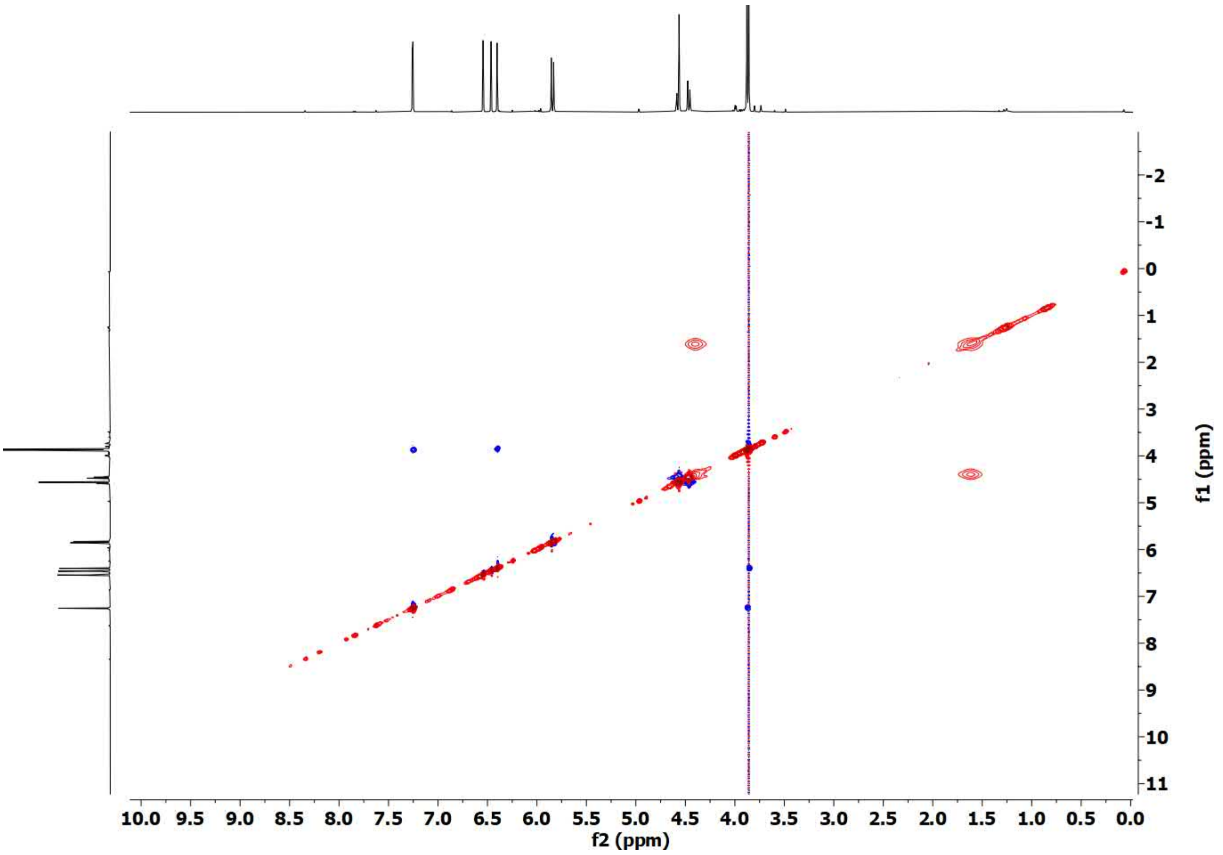

Figure S15. NOESY (500 MHz, $CDCl_3$, 25 °C) spectrum of oblarotenoid F (**2**)

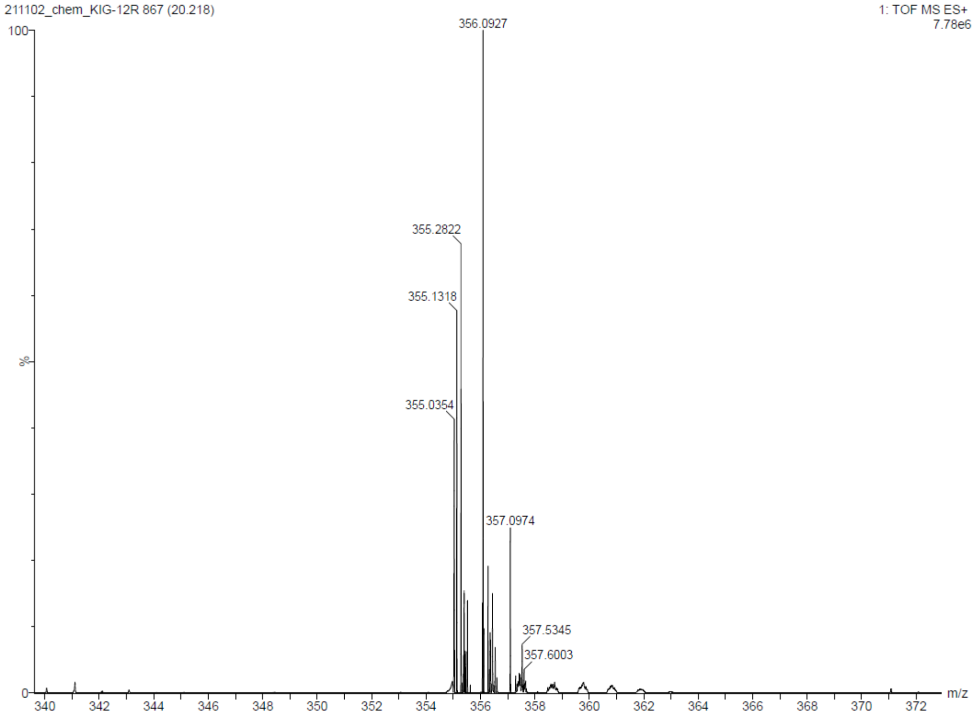

Figure S16. HRESIMS spectrum of oblarotenoid F (**2**)

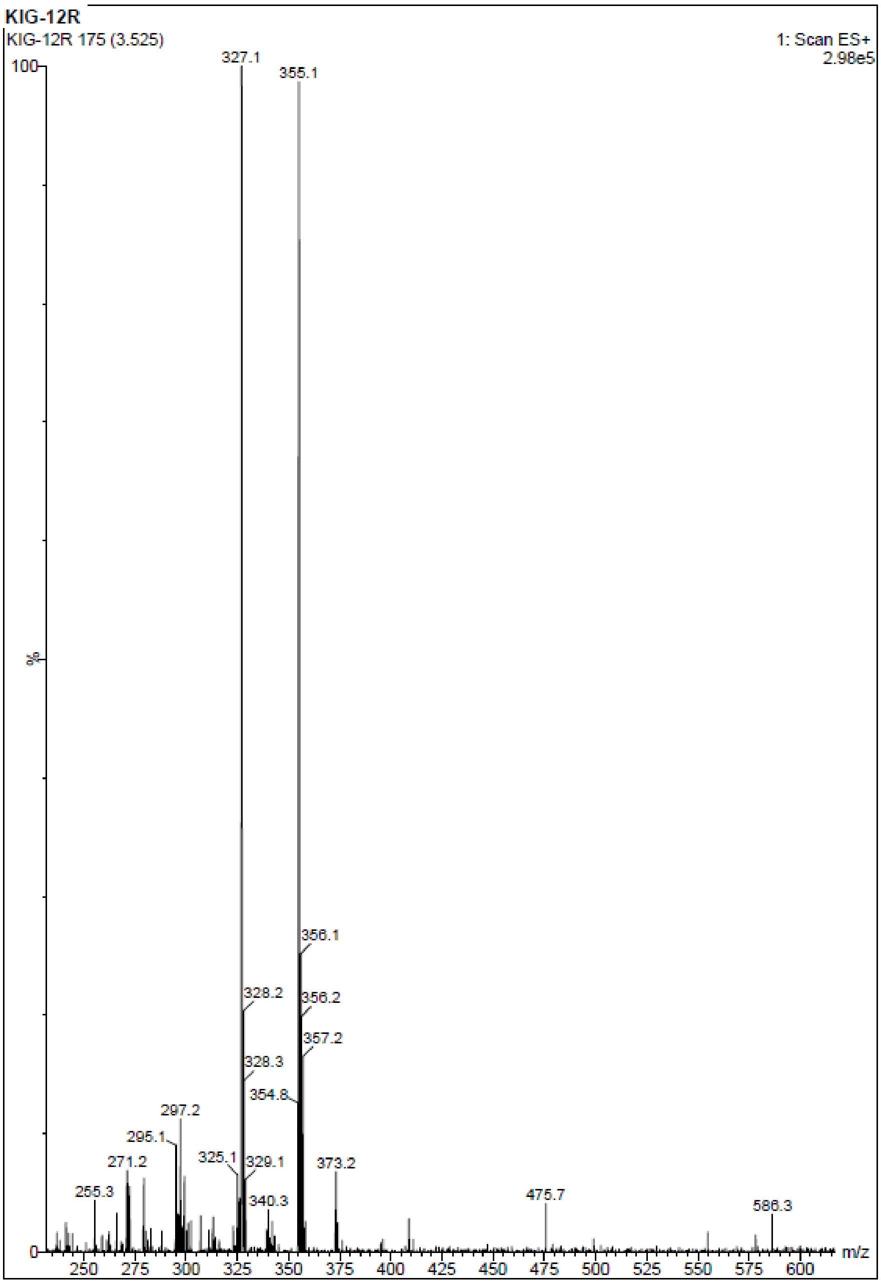

Figure S17. MS spectrum of oblarotenoid F (**2**)

Spectroscopic Data of Oblarotenoid G (3)

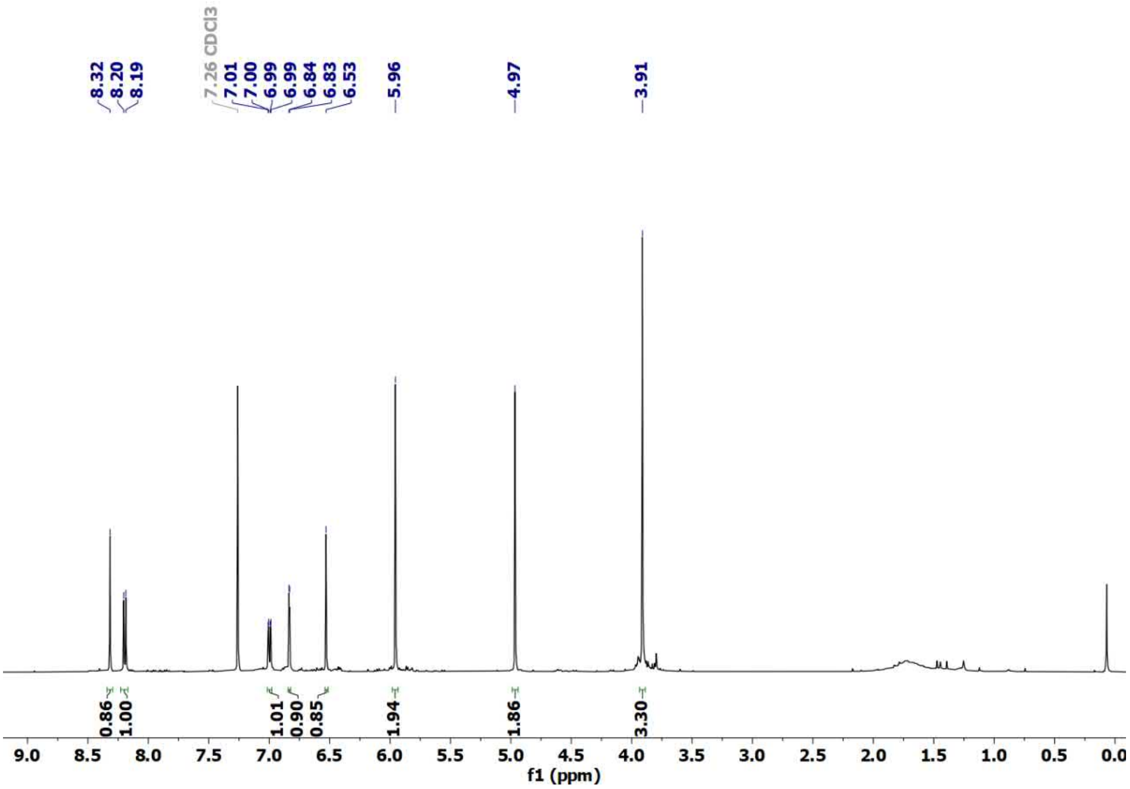

Figure S18. ^{1}H NMR (500 MHz, $CDCl_3$, 25 ºC) spectrum of and was given the trivial name oblarotenoid G (**3**)

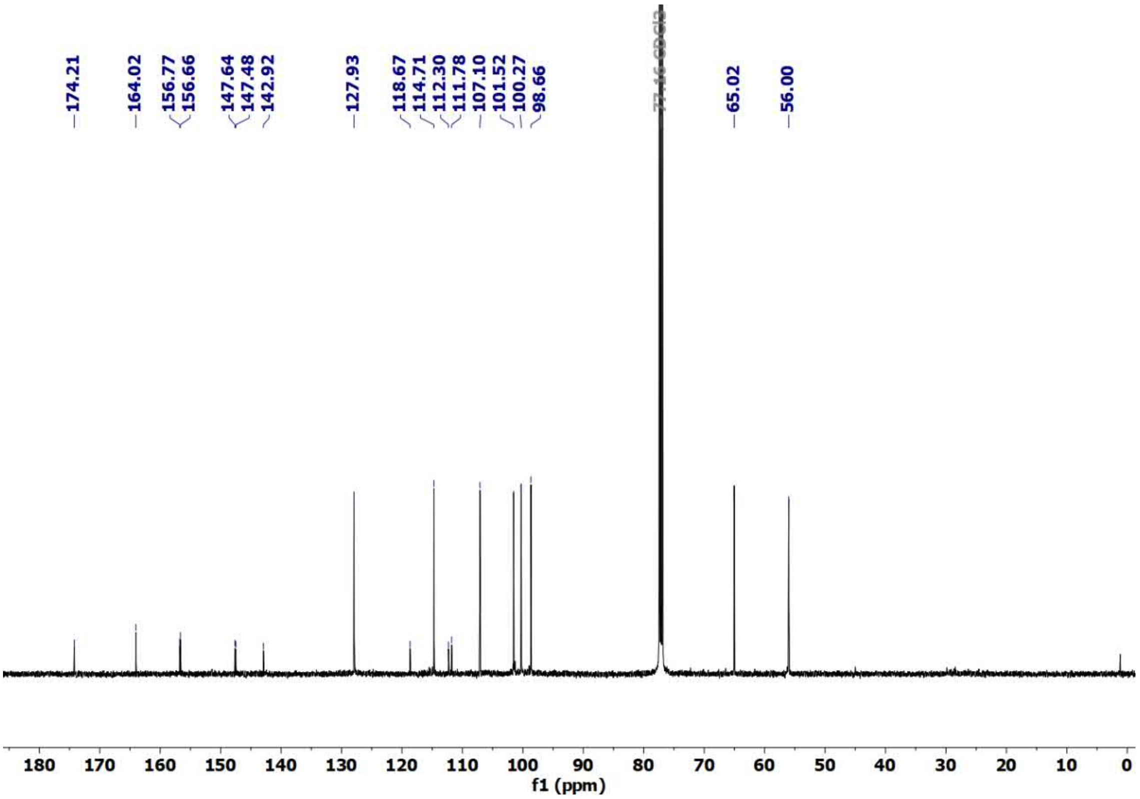

Figure S19. ^{13}C NMR (125 MHz, $CDCl_3$, 25 ºC) spectrum of oblarotenoid G (**3**)

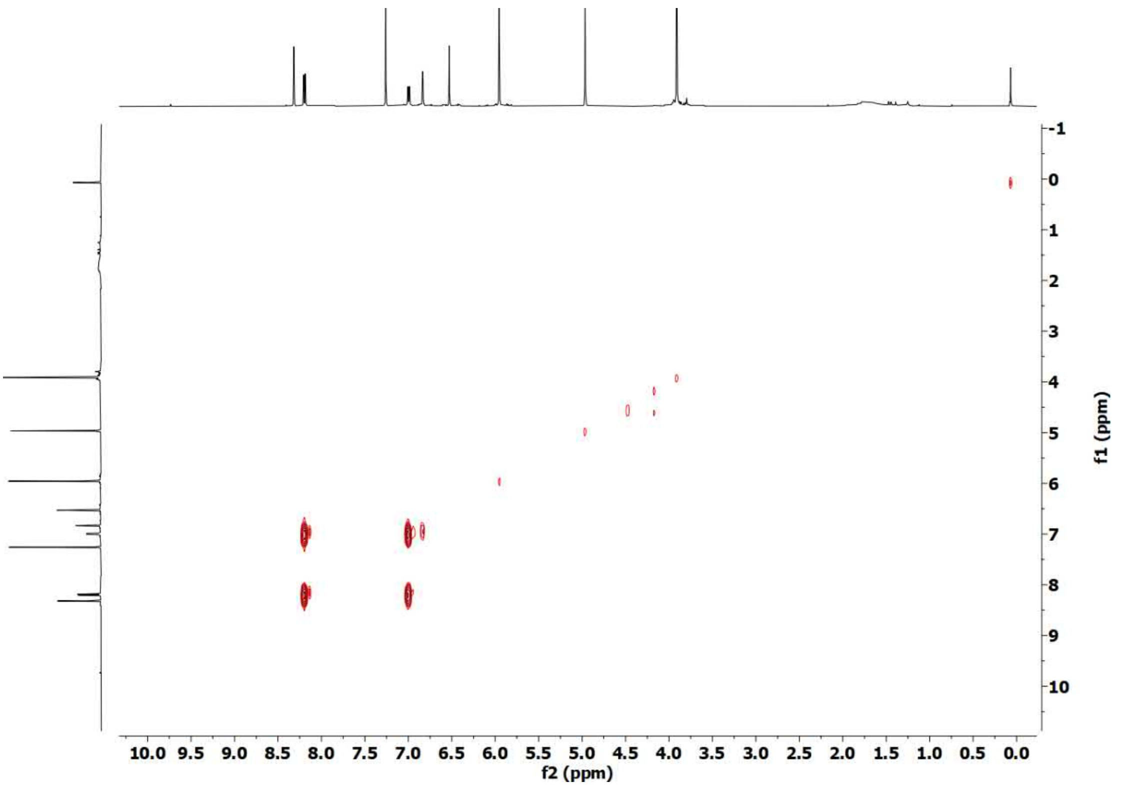

Figure S20. COSY (500 MHz, $CDCl_3$, 25 ºC) spectrum of oblarotenoid G (**3**)

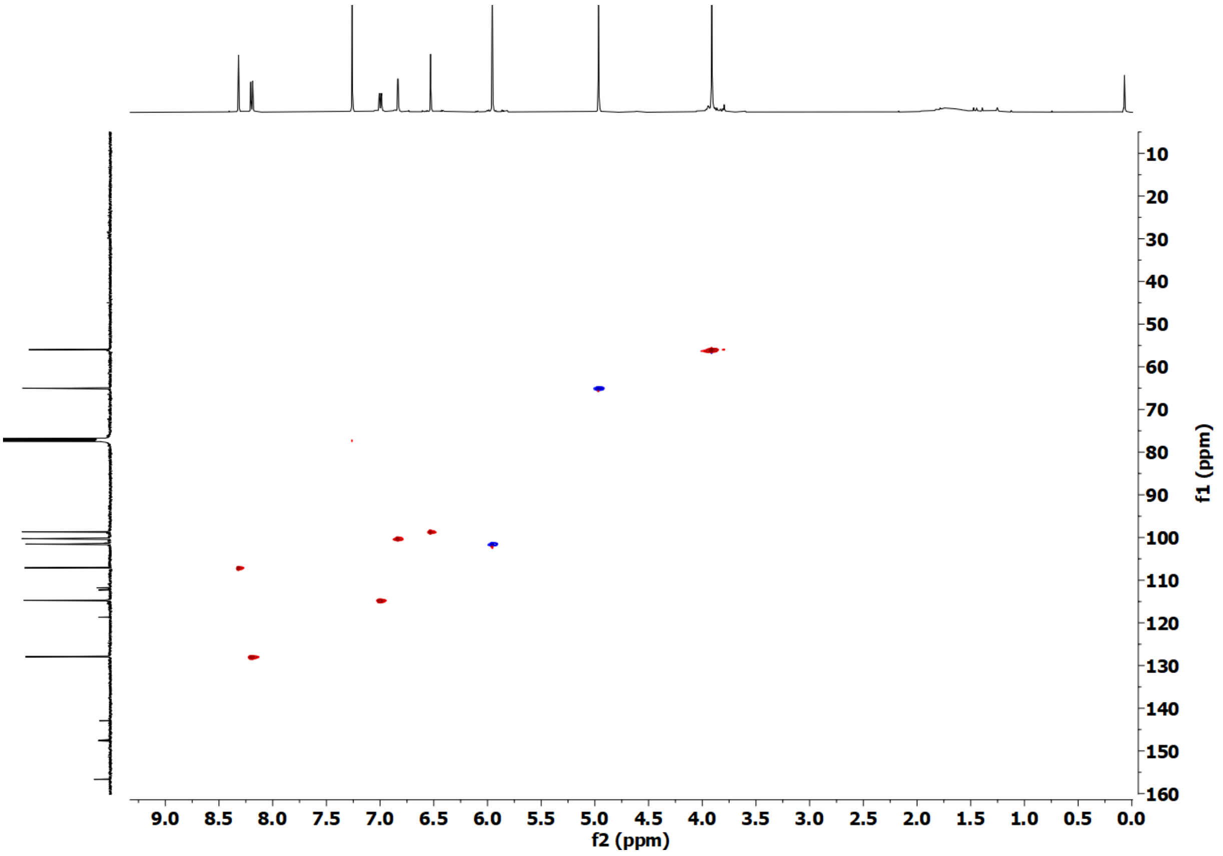

Figure S21. HSQC (500/125 MHz, $CDCl_3$, 25 ºC) spectrum of oblarotenoid G (**3**)

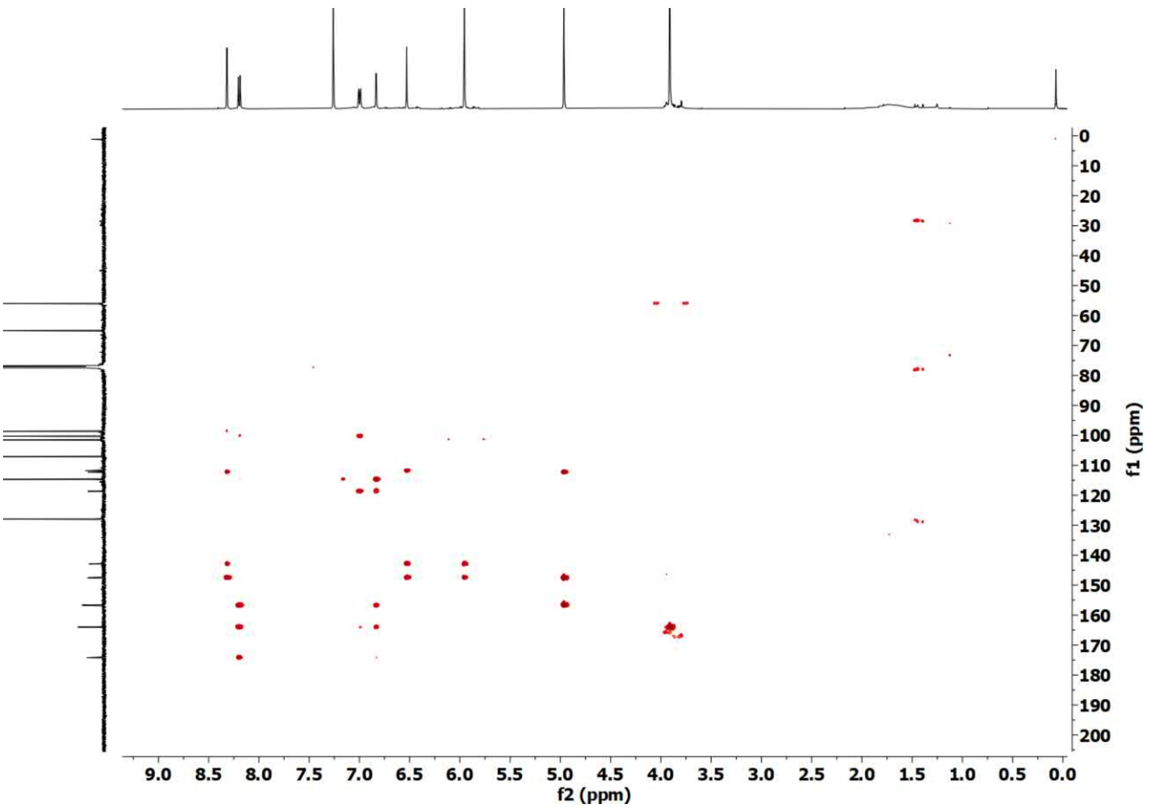

Figure S22. HMBC (500/125 MHz, $CDCl_3$, 25 ºC) spectrum of oblarotenoid G (**3**)

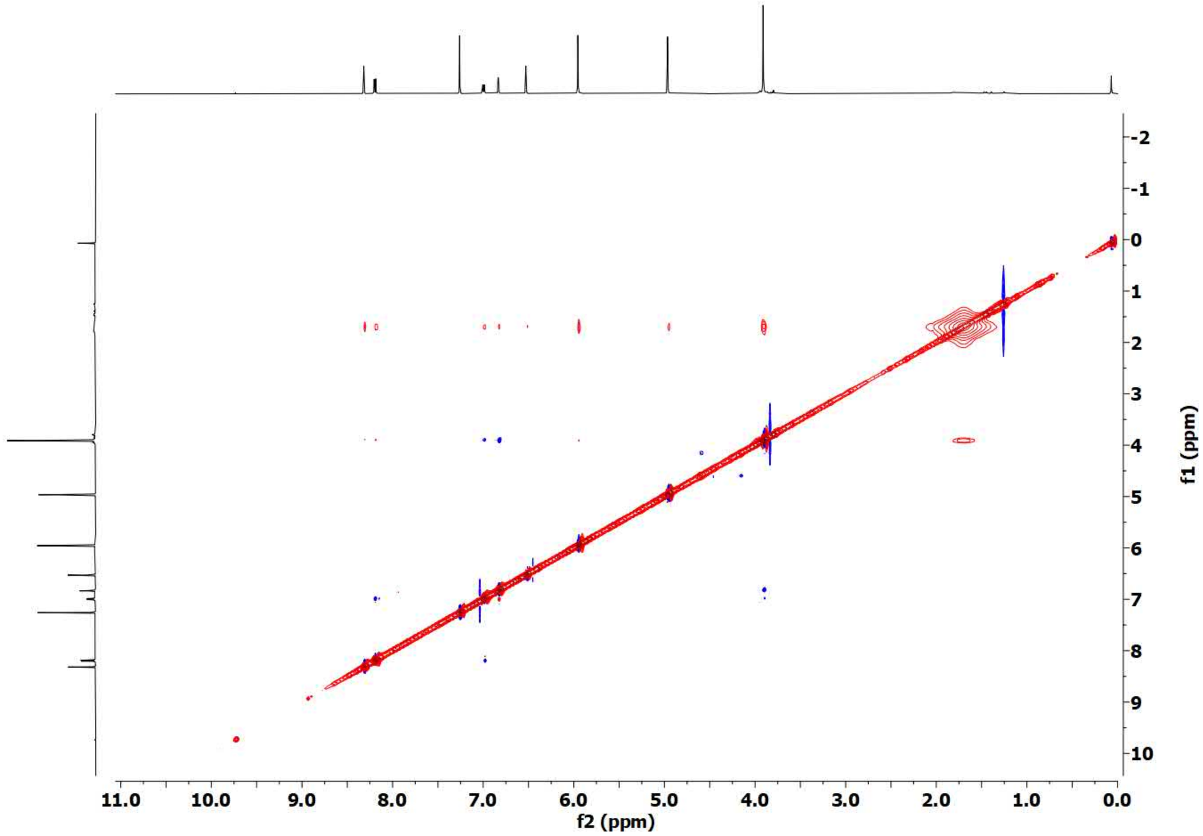

Figure S23. NOESY (500 MHz, $CDCl_3$, 25 ºC) spectrum of oblarotenoid G (**3**)

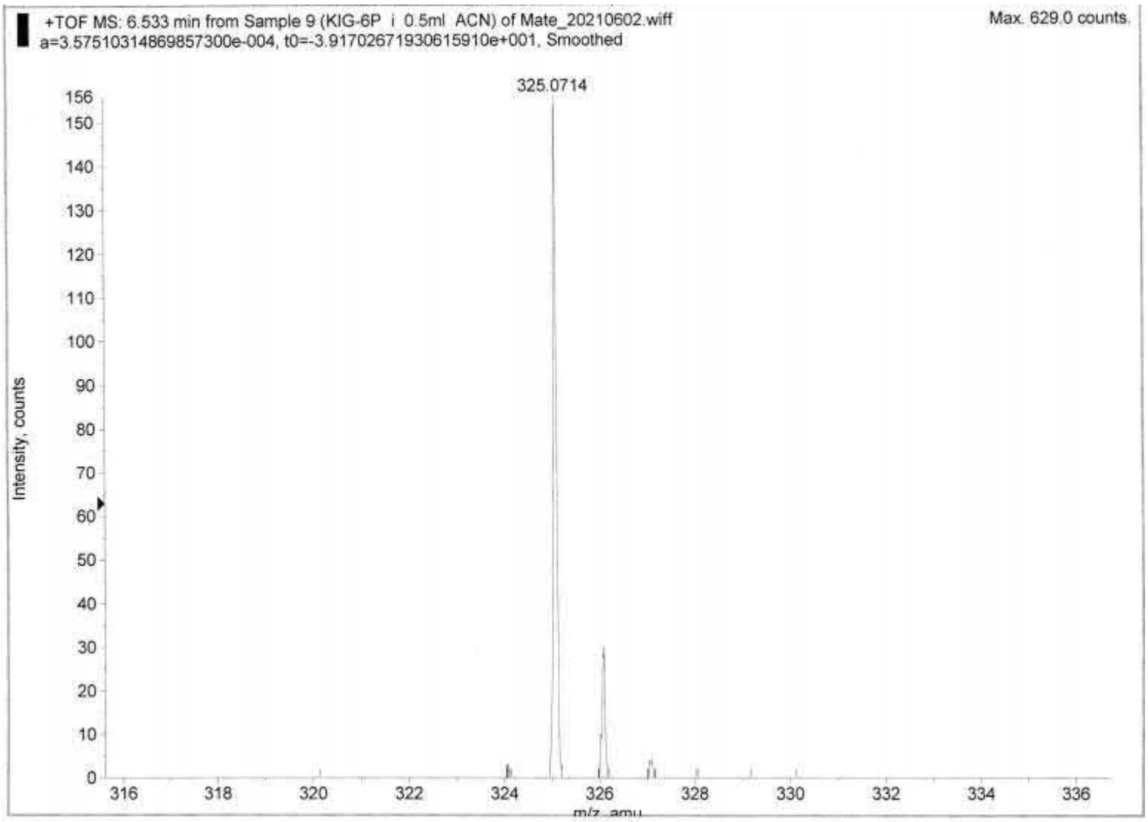

Figure S24. HRESIMS spectrum of oblarotenoid G (**3**)

Spectroscopic Data of Obloneside (4)

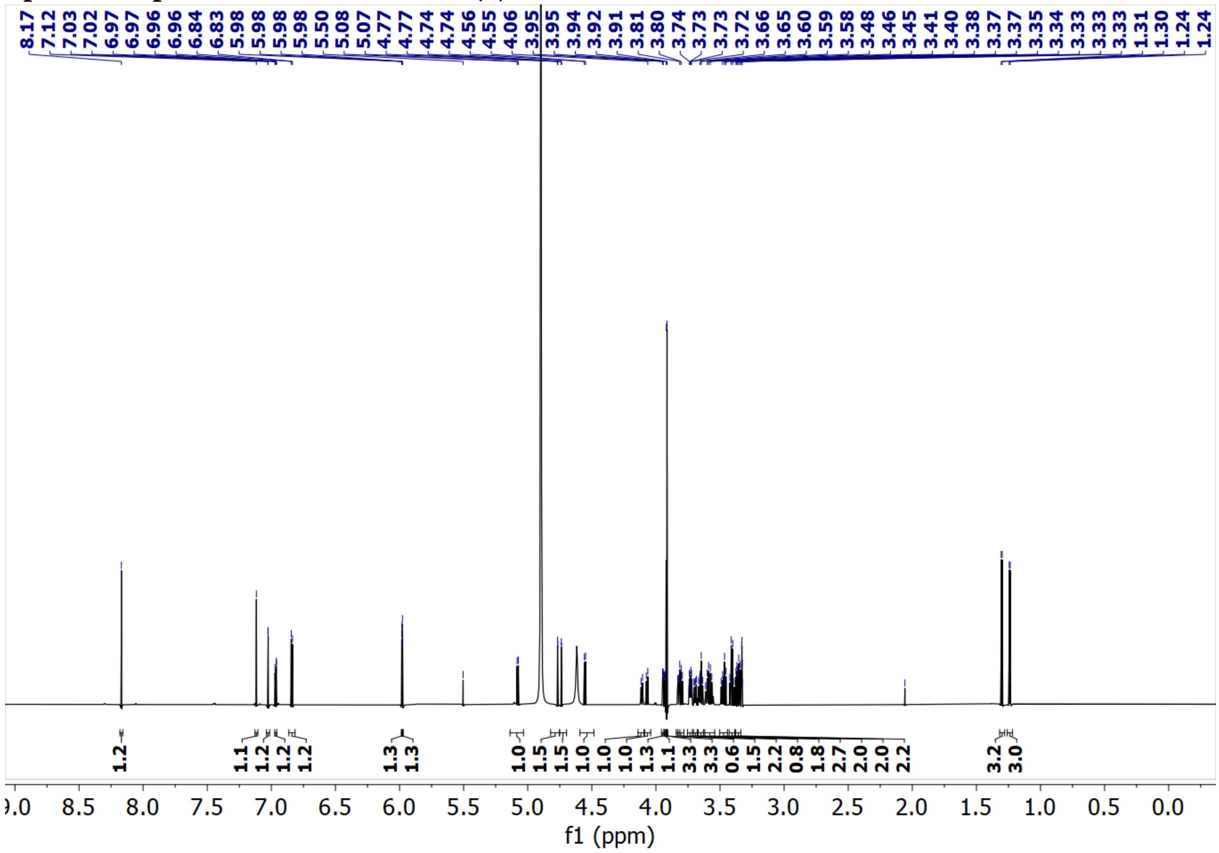

Figure S25. ^{1}H NMR (800 MHz, CD_3OD, 25 ºC) spectrum of oblonesiden (**4**)

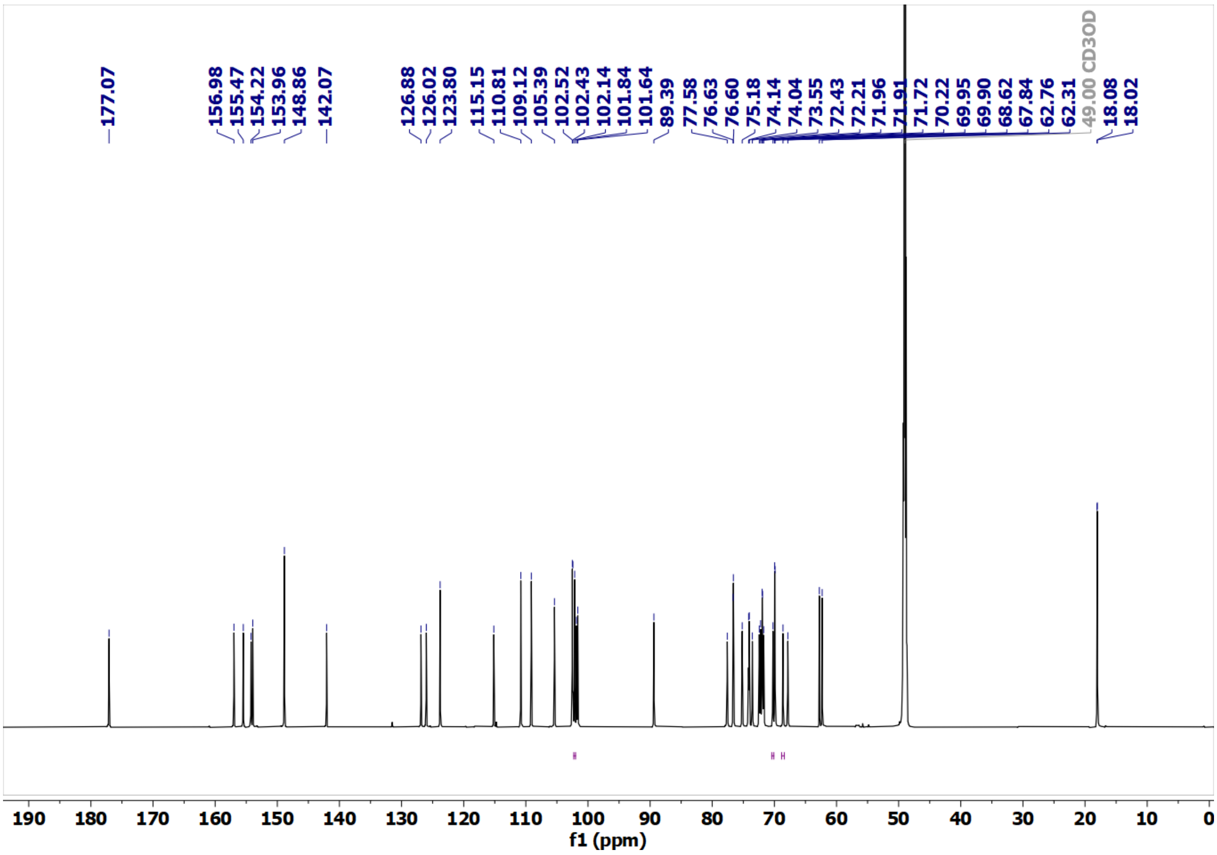

Figure S26. ^{13}C NMR (200 MHz, CD_3OD, 25 ºC) spectrum of obloneside (**4**)

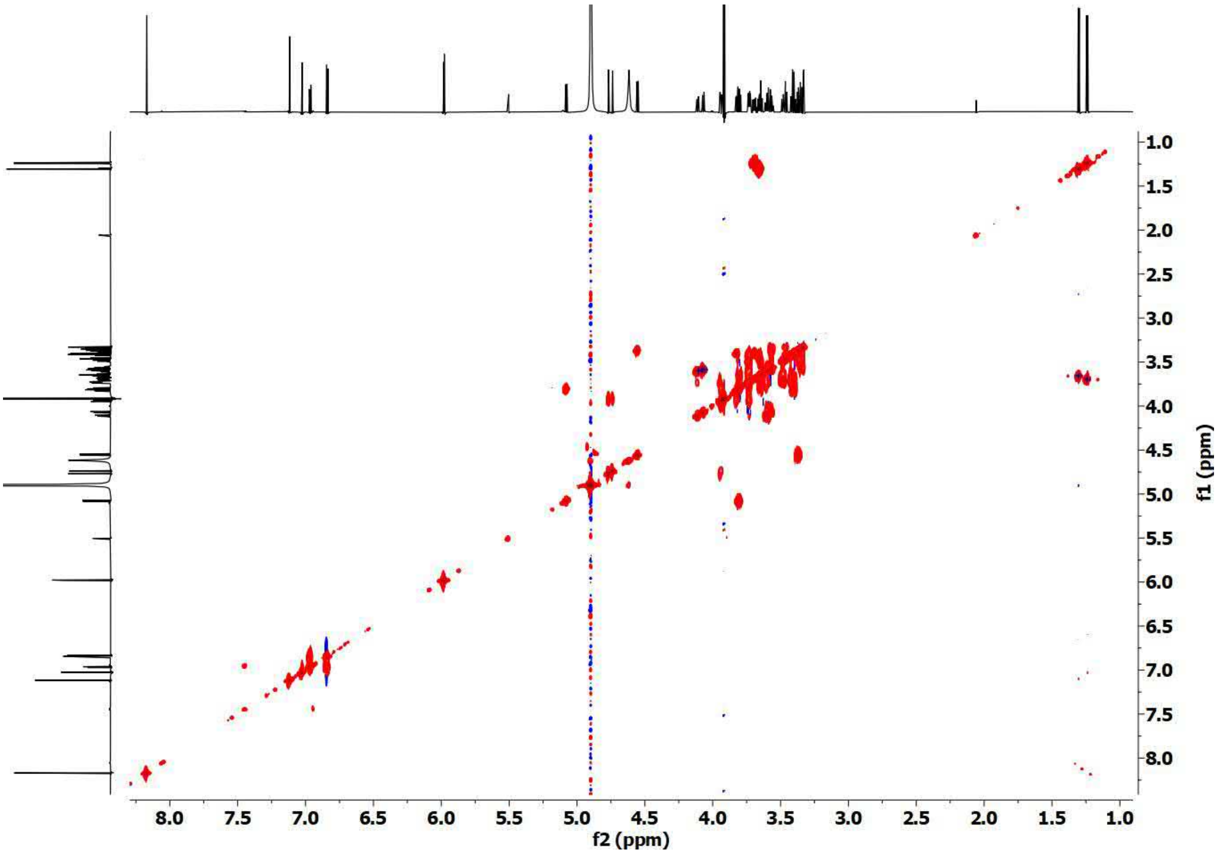

Figure S27. COSY (800 MHz, CD_3OD, 25 ºC) spectrum of obloneside (**4**).

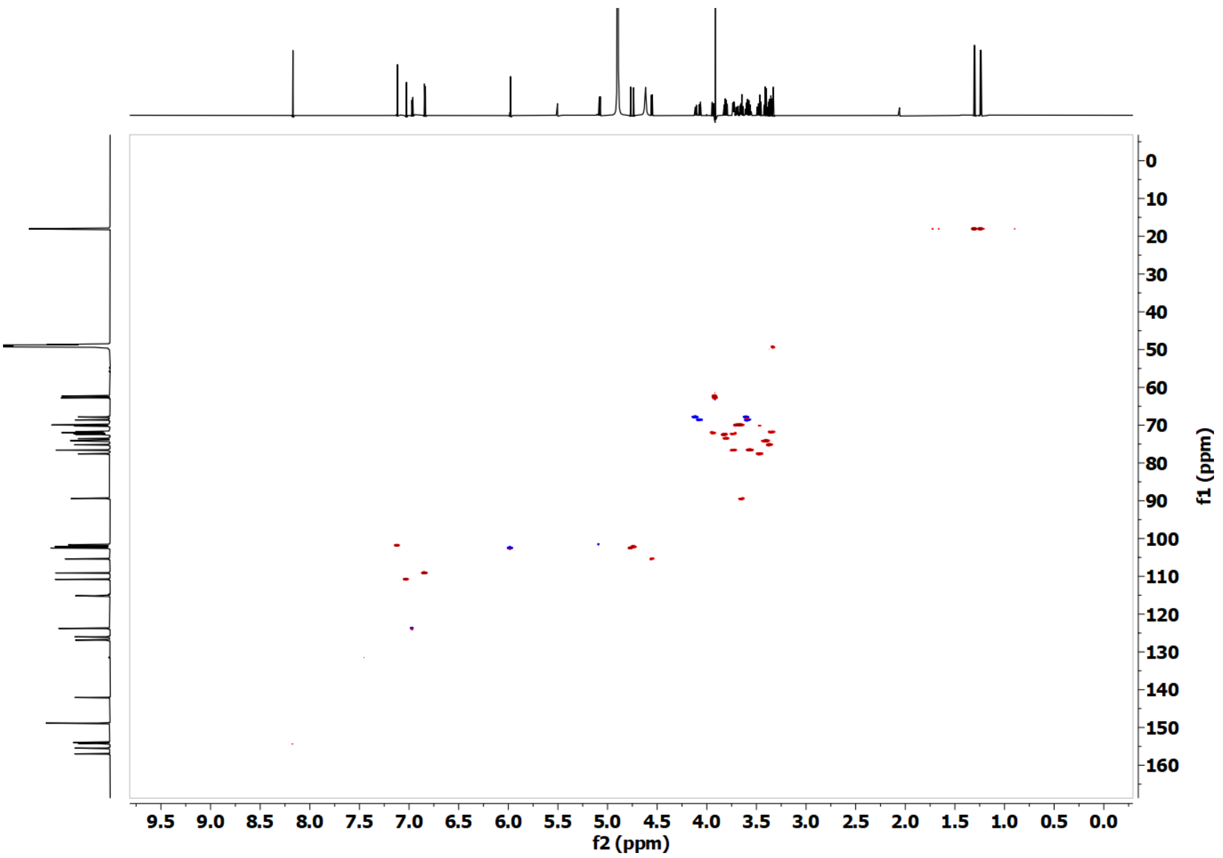

Figure S28. HSQC (800/200 MHz, CD_3OD 25 ºC) spectrum of obloneside (**4**).

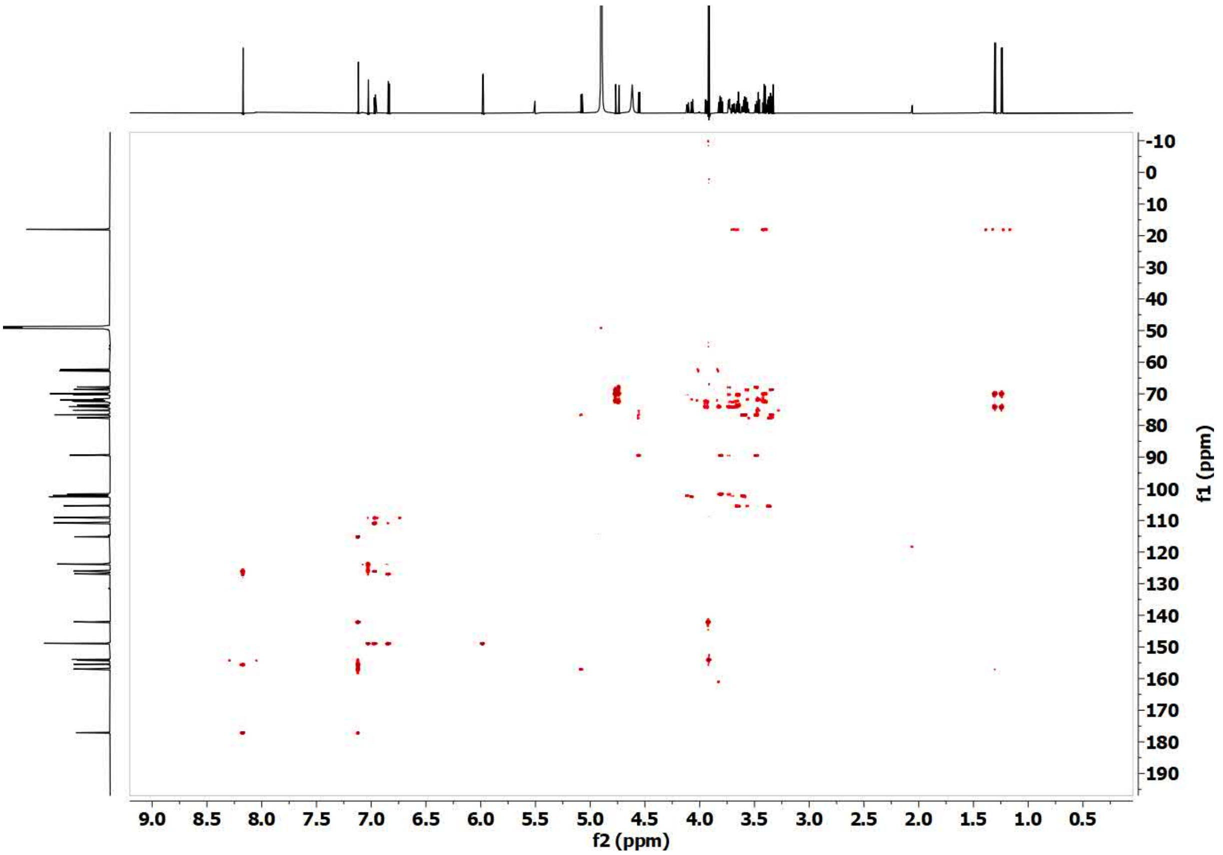

Figure S29. HMBC (800/200 MHz, CD_3OD, 25 ºC) spectrum of obloneside (**4**).

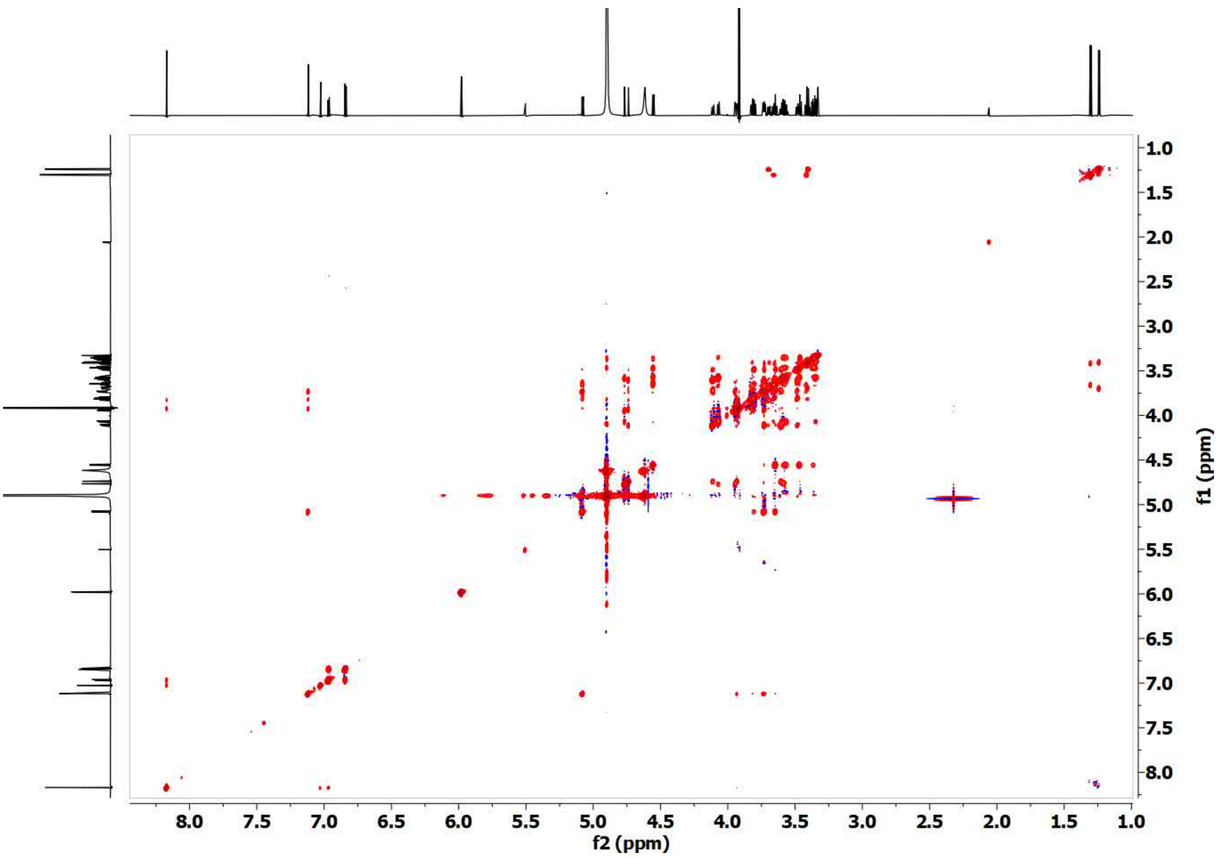

Figure S30. NOESY (800 MHz, CD_3OD, 25 ºC) spectrum of obloneside (**4**).

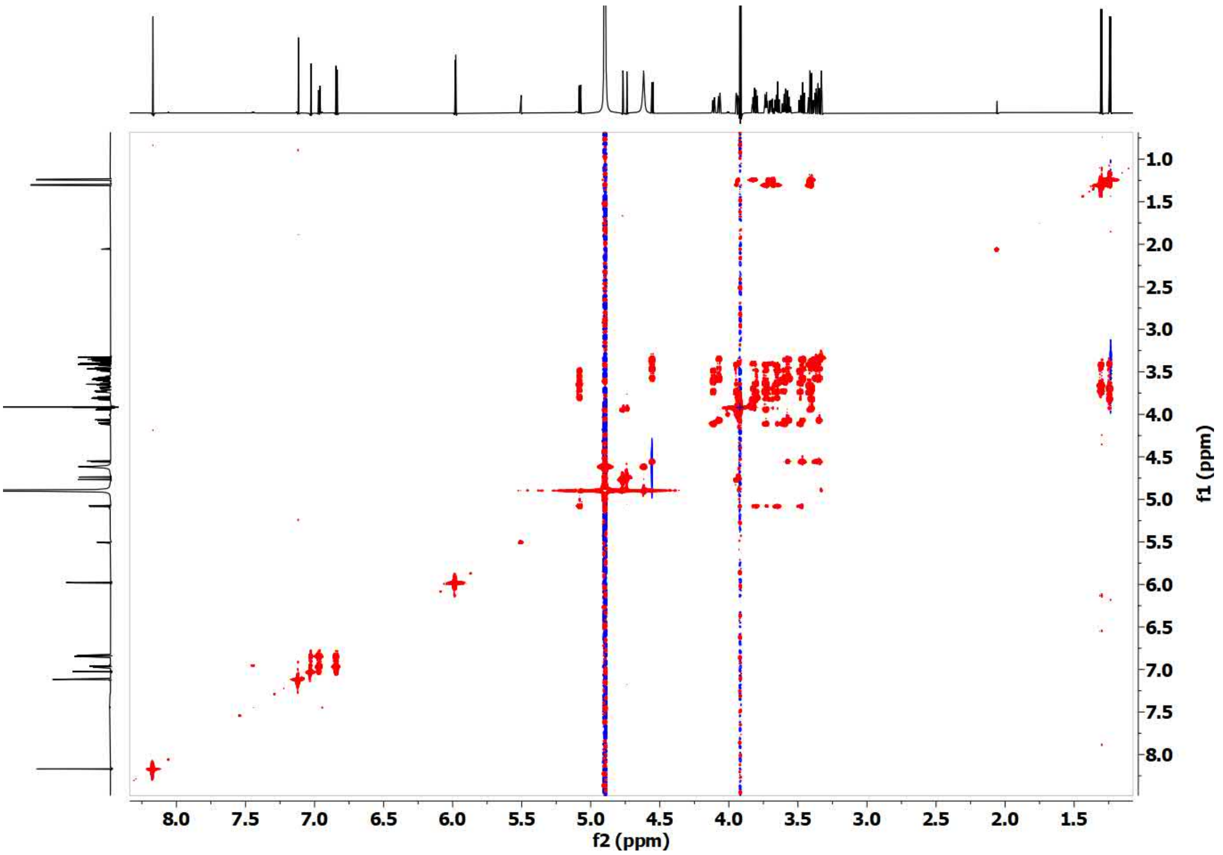

Figure S31. TOCSY (800 MHz, CD_3OD, 25 ºC) spectrum of obloneside (**4**).

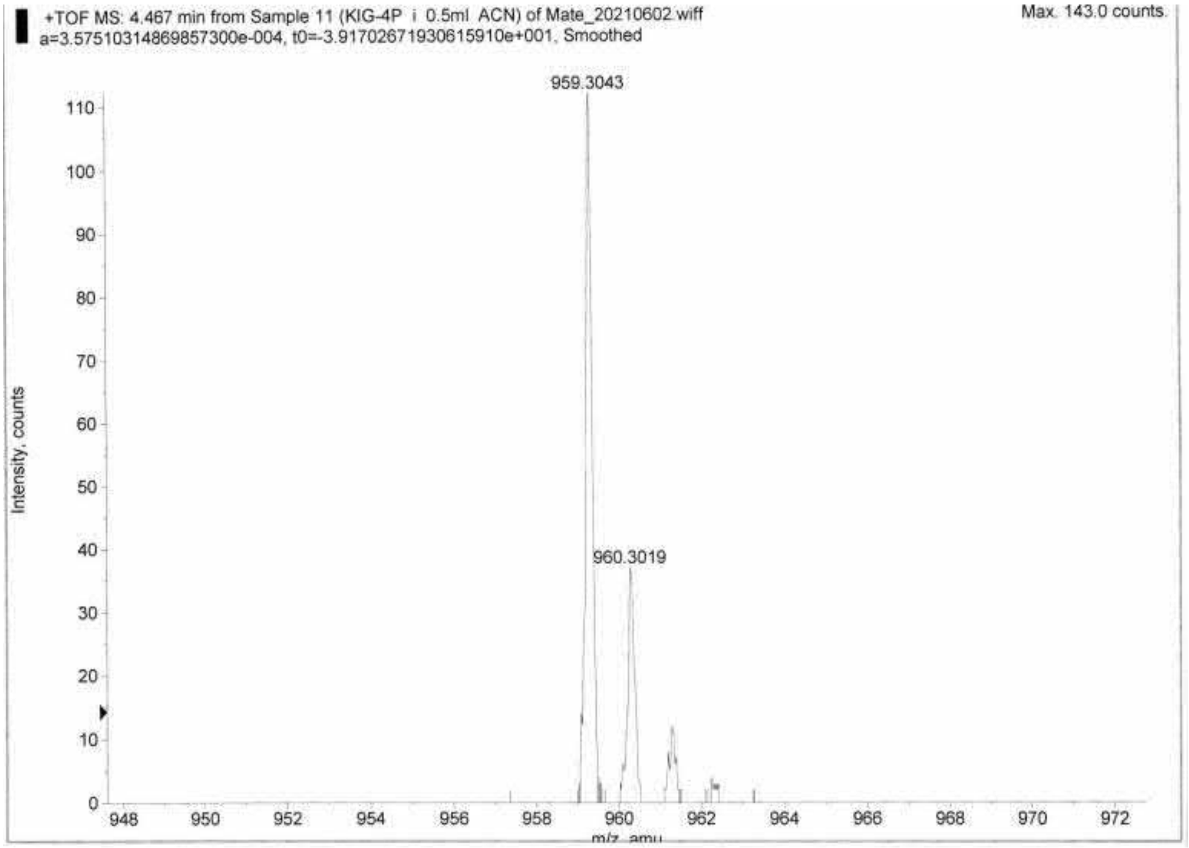

Figure S32. HRESIMS spectrum of obloneside (**4**)

Spectroscopic Data of 6-Hydroxy-7,8-dimethoxy-3',4'-methylenedioxyisoflavone (4a)

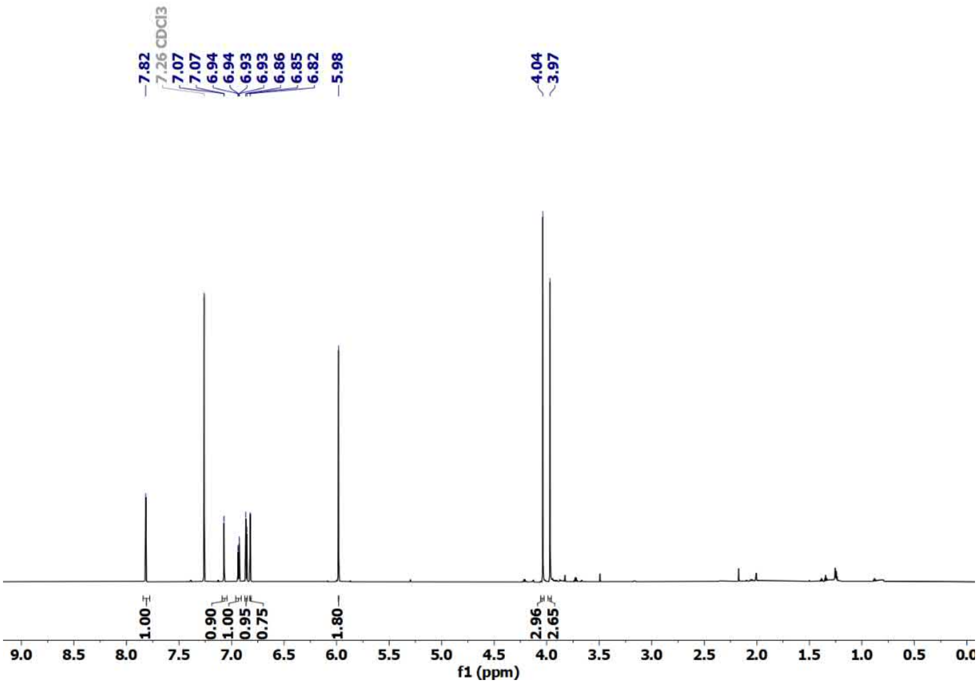

Figure S33. ^{1}H NMR (800 MHz, CD_3OD, 25 ºC) spectrum of 6-hydroxy-7,8-dimethoxy-3',4'-methylenedioxyisoflavone (**4a**)

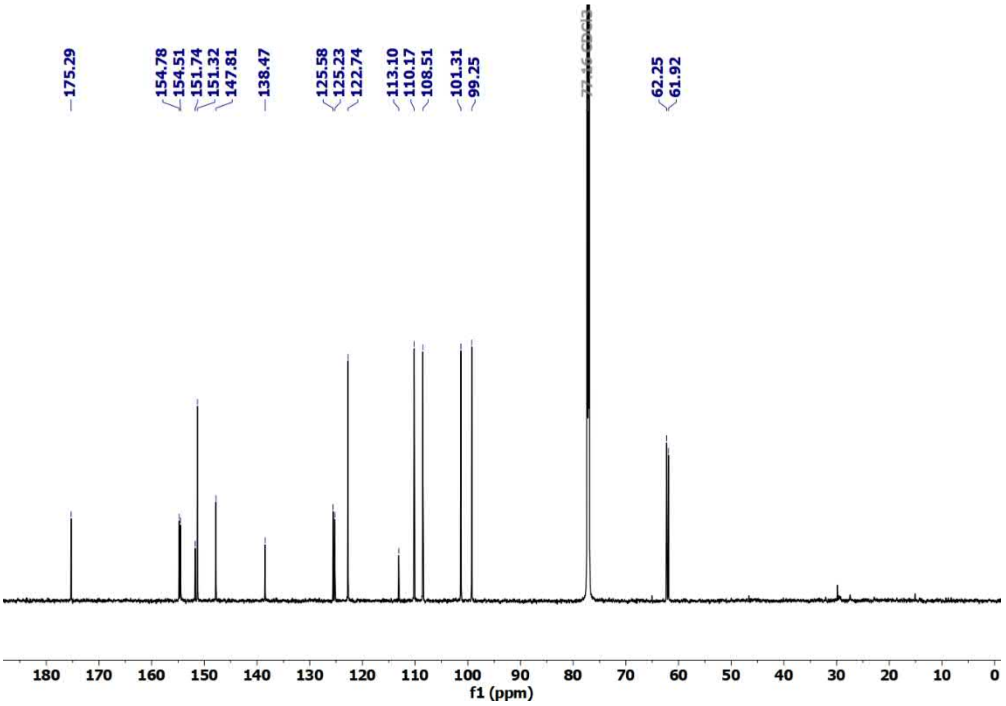

Figure S34. ^{13}C NMR (200 MHz, CD_3OD, 25 ºC) spectrum of 6-hydroxy-7,8-dimethoxy-3',4'-methylenedioxyisoflavone (**4a**)

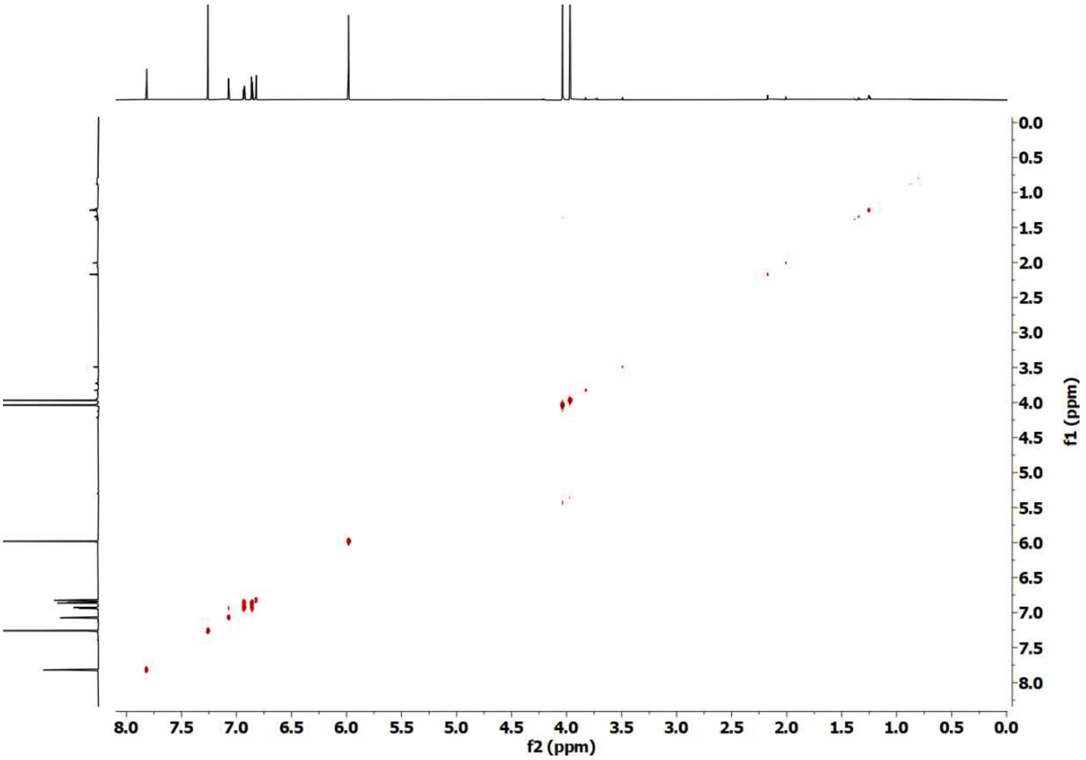

Figure S35. COSY (800 MHz, CD_3OD, 25 ºC) spectrum of 6-hydroxy-7,8-dimethoxy-3',4'-methylenedioxyisoflavone (**4a**)

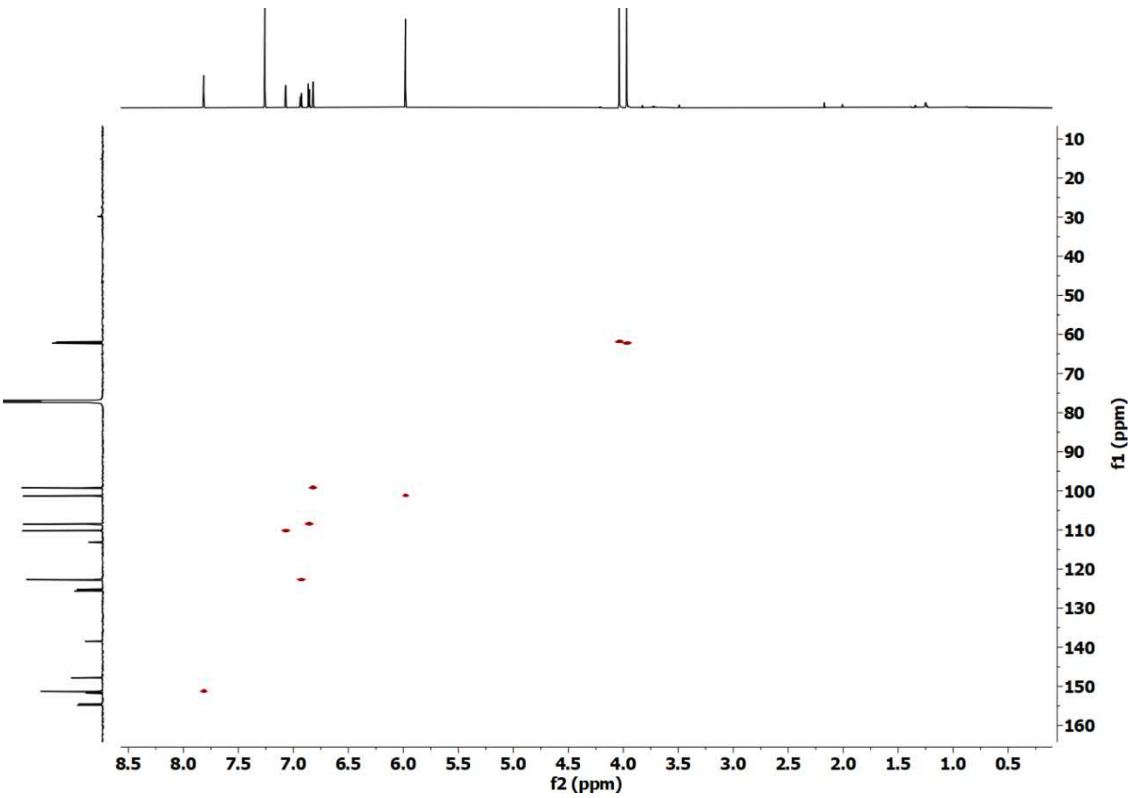

Figure S36. HSQC (800/200 MHz, CD3OD 25 ºC) spectrum of 6-hydroxy-7,8-dimethoxy-3',4'-methylenedioxyisoflavone (**4a**)

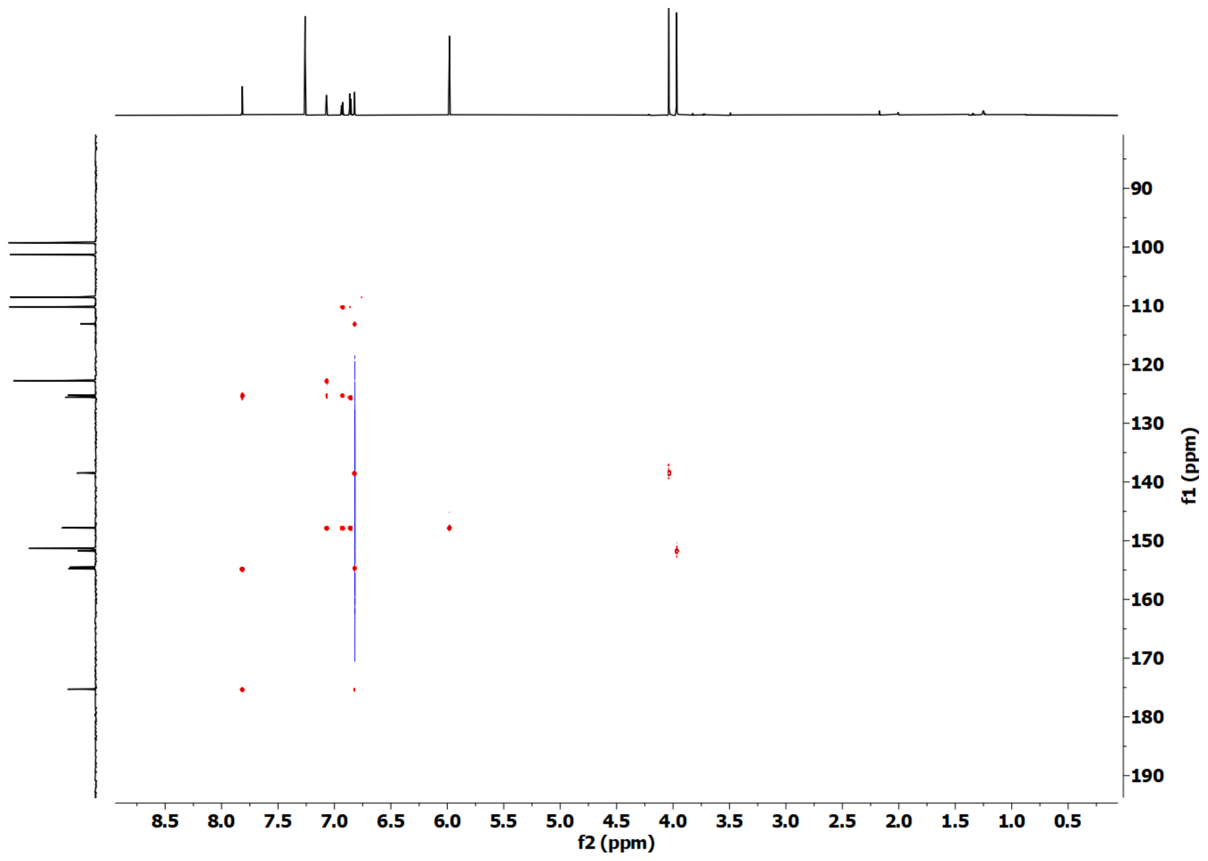

Figure S37. HMBC (800/200 MHz, CD3OD, 25 ºC) spectrum of 6-hydroxy-7,8-dimethoxy-3',4'-methylenedioxyisoflavone (**4a**)

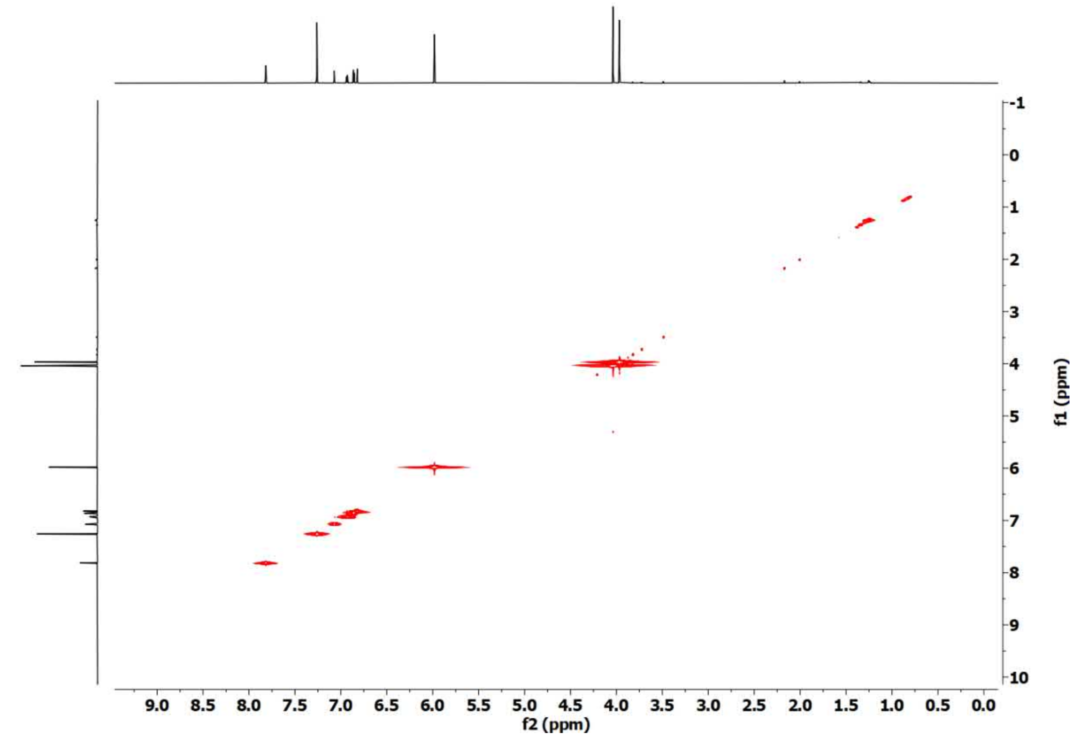

Figure S38. NOESY (800 MHz, CD_3OD, 25 ºC) spectrum of 6-hydroxy-7,8-dimethoxy-3',4'-methylenedioxyisoflavone (**4a**)

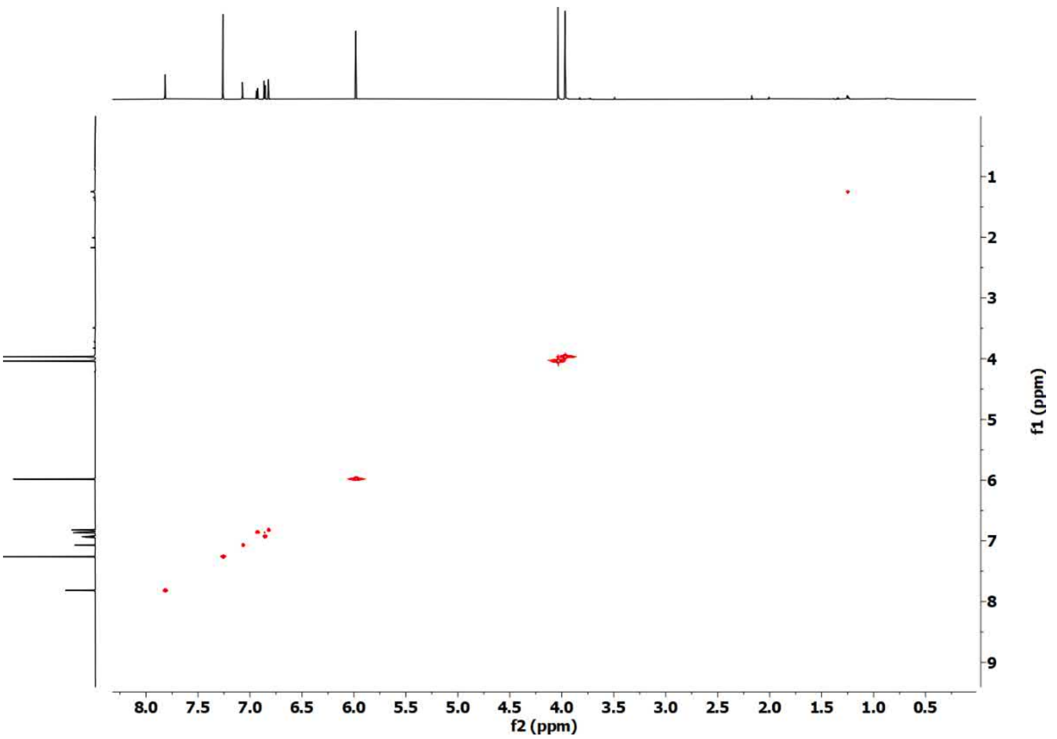

Figure S39. TOCSY (800 MHz, CD_3OD, 25 ºC) spectrum of 6-hydroxy-7,8-dimethoxy-3',4'-methylenedioxyisoflavone (**4a**)

Spectroscopic Data of Oblarotenoid C (5)

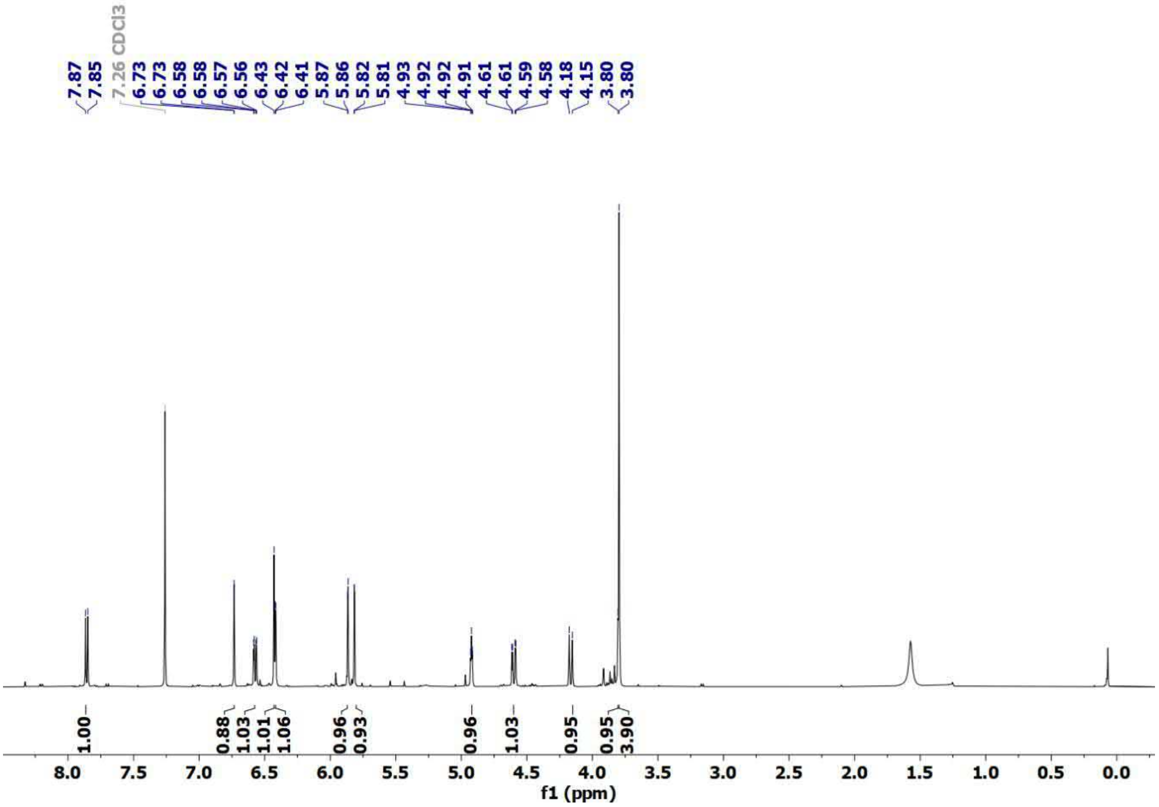

Figure S40. ^{1}H NMR (500 MHz, $CDCl_3$, 25 ºC) spectrum oblarotenoid C (**5**)

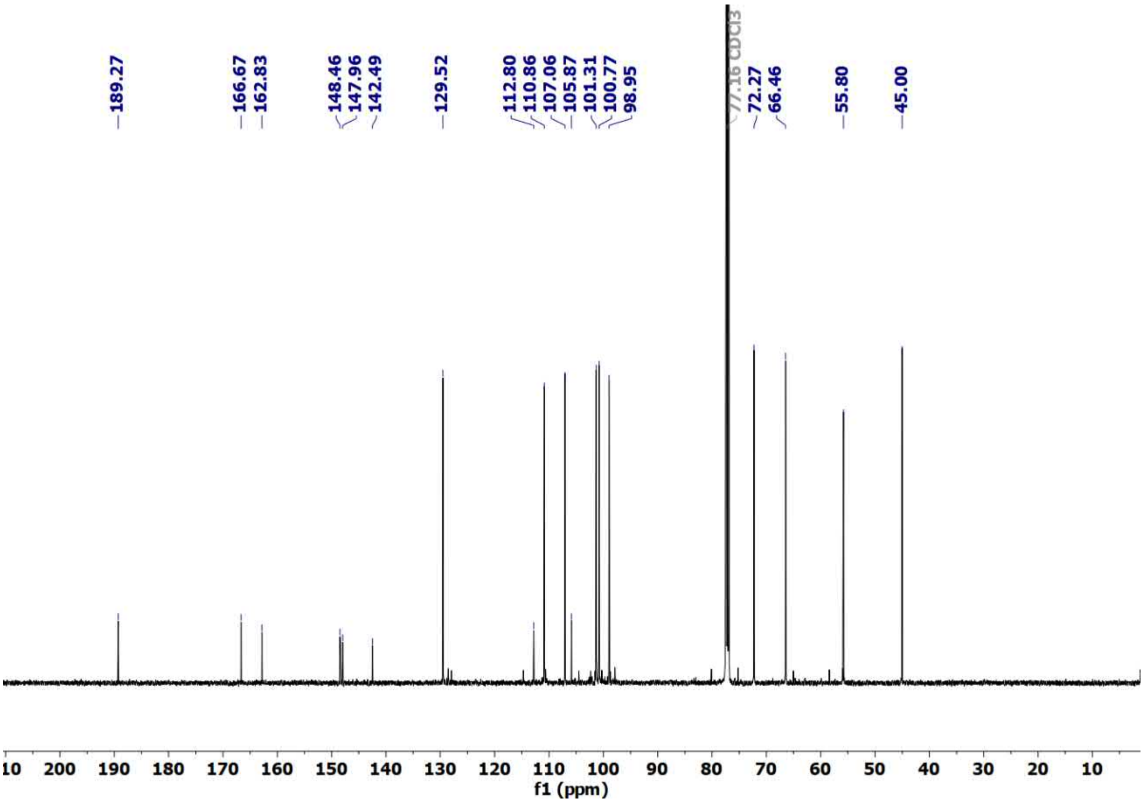

Figure S41. ^{13}C NMR (125 MHz, $CDCl_3$, 25 ºC) spectrum of oblarotenoid C (**5**).

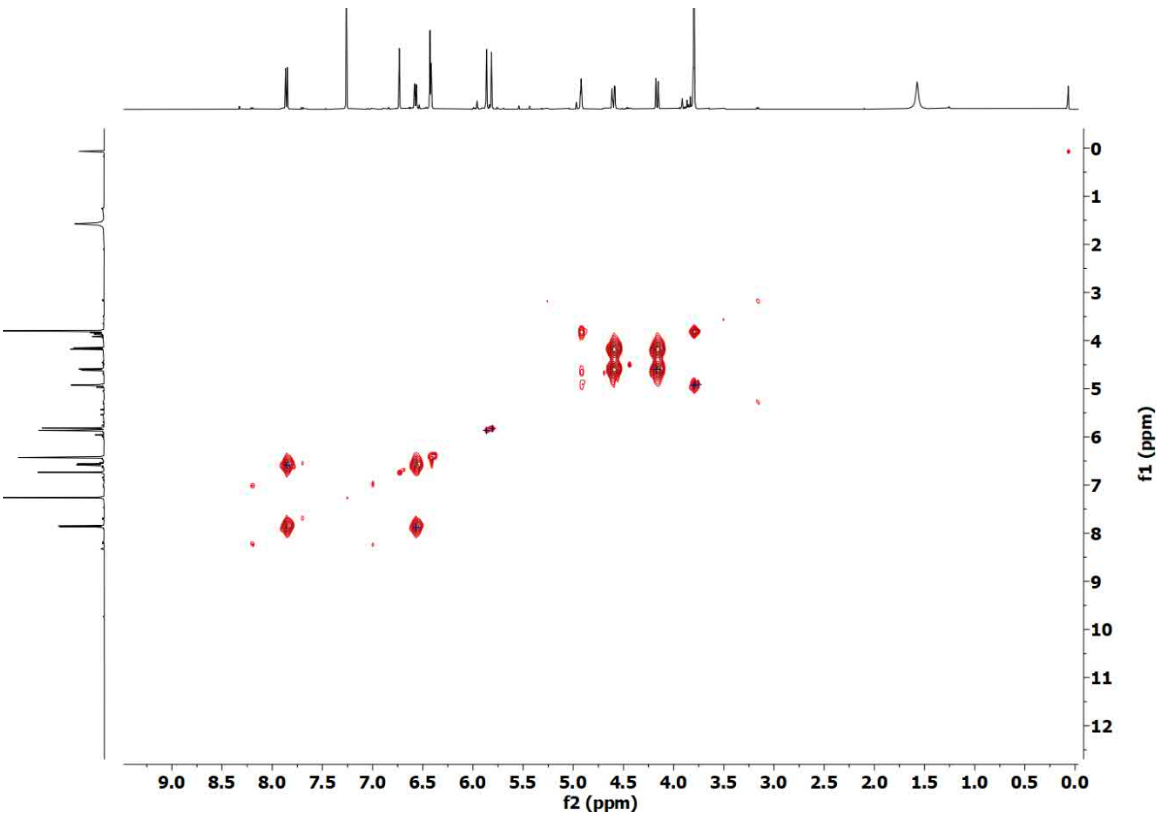

Figure S42. COSY (500 MHz, $CDCl_3$, 25 ºC) spectrum of oblarotenoid C (**5**).

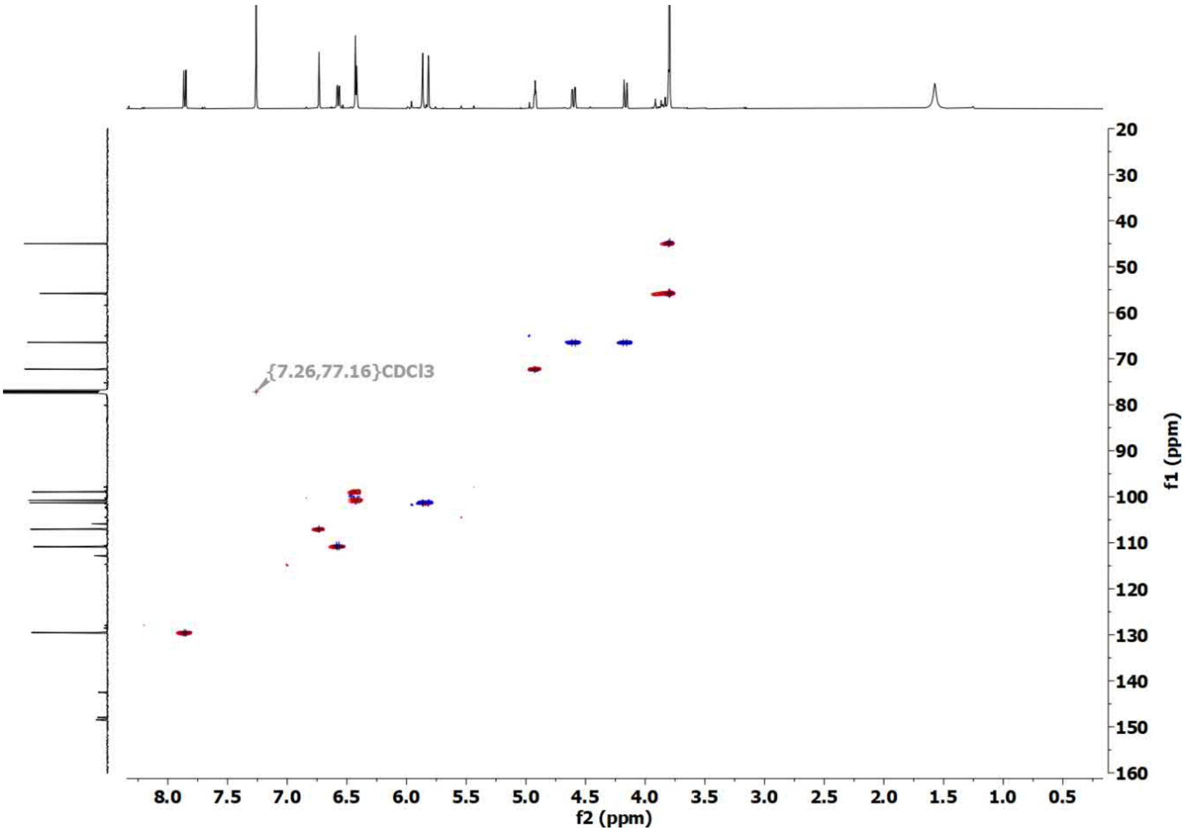

Figure S43. HSQC (500/125 MHz, $CDCl_3$, 25 ºC) spectrum of oblarotenoid C (**5**).

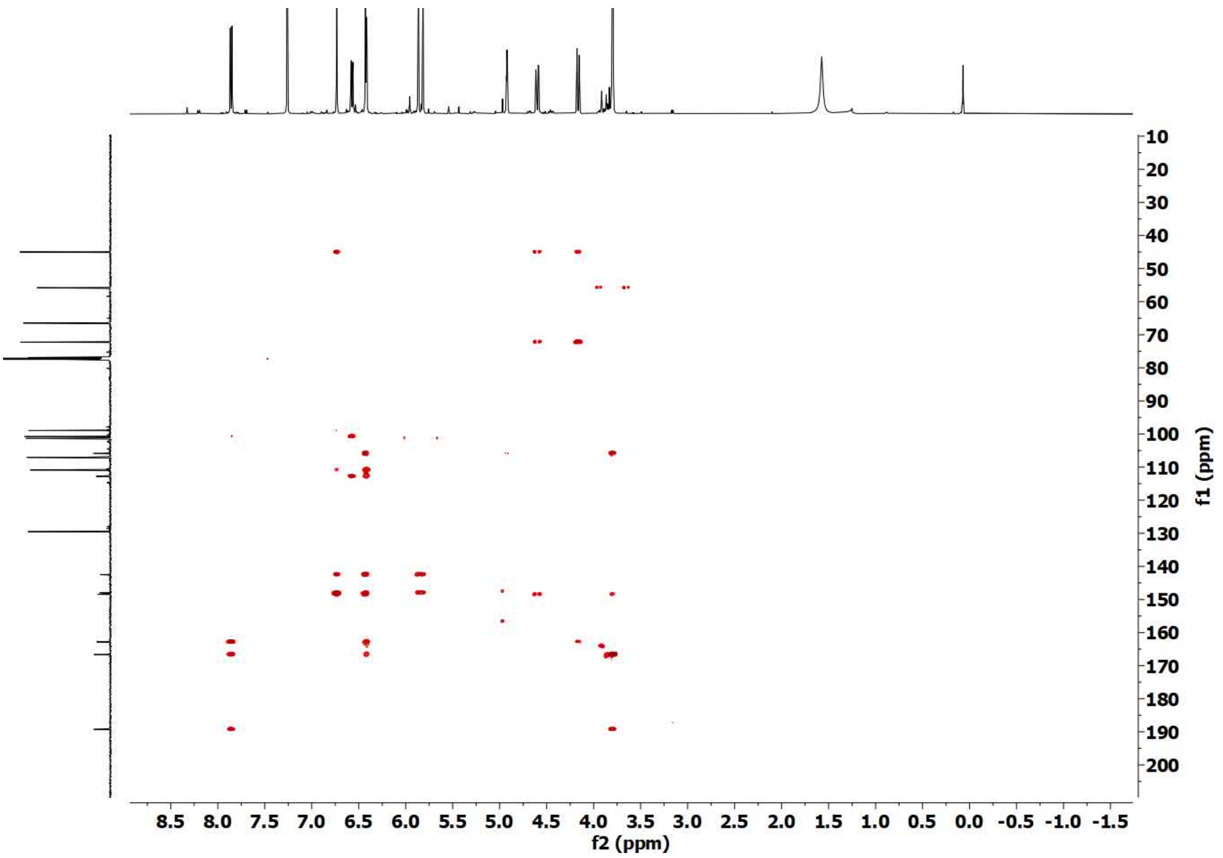

Figure S44. HMBC (500/125 MHz, $CDCl_3$, 25 ºC) spectrum of oblarotenoid C (**5**).

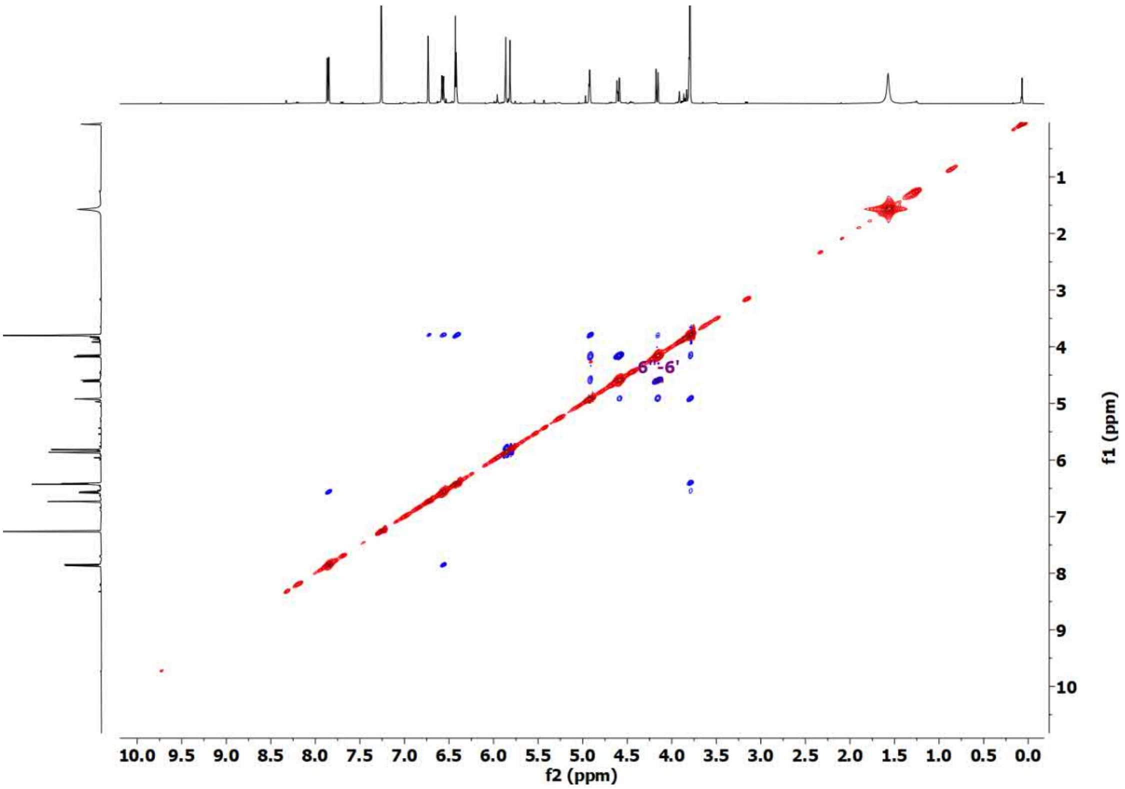

Figure S45. NOESY (500 MHz, $CDCl_3$, 25 ºC) spectrum of oblarotenoid C (**5**).

Spectroscopic Data of Oblarotenoid A (6)

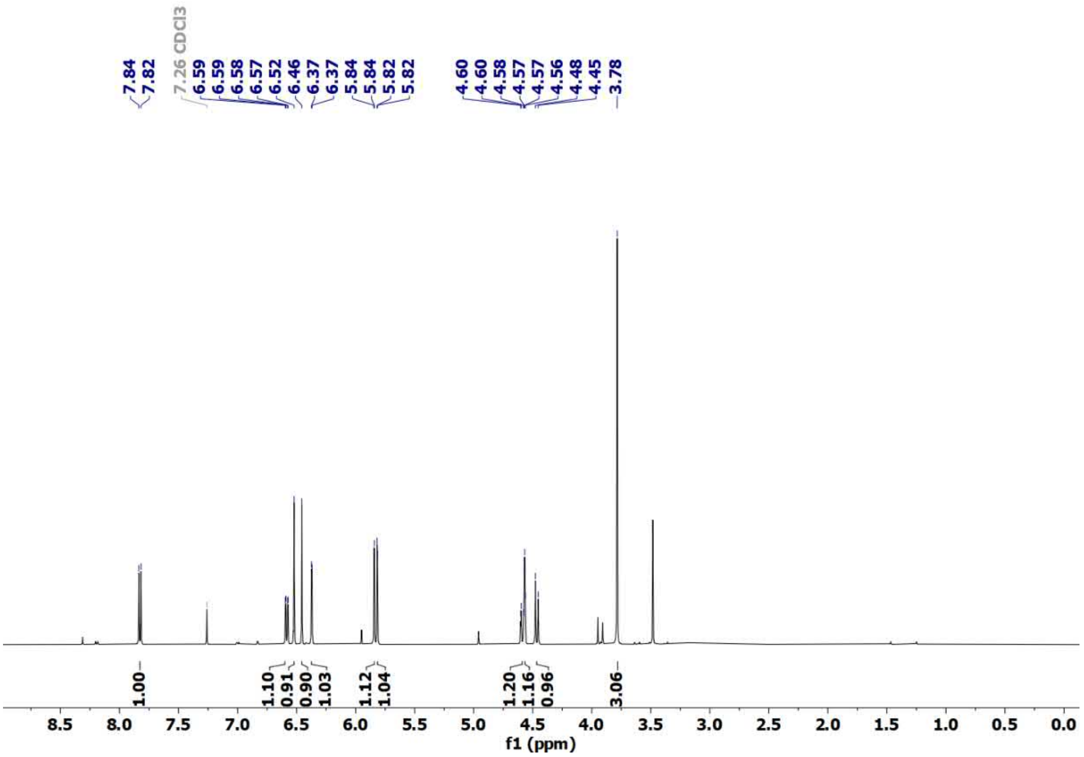

Figure S46. ^{1}H NMR (500 MHz, $CDCl_3$, 25 ºC) spectrum of oblarotenoid A (**6**)

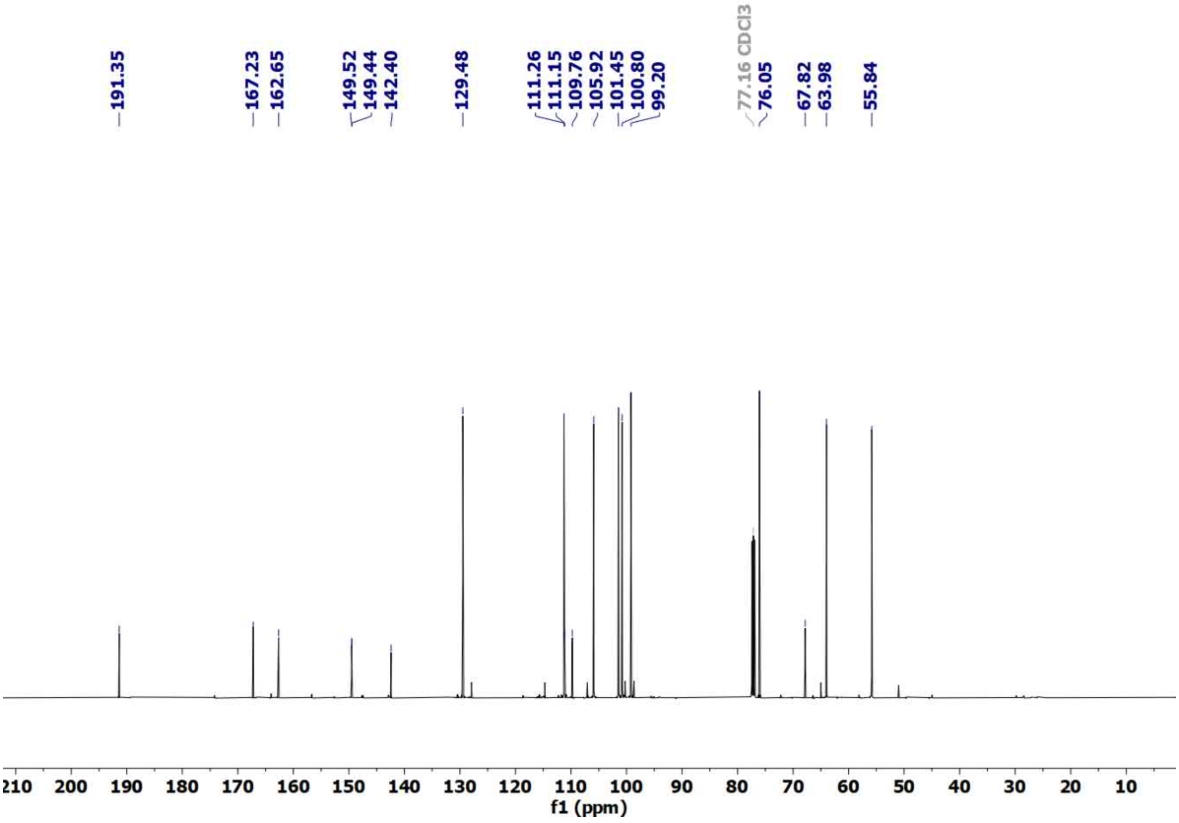

Figure S47. ^{13}C NMR (125 MHz, $CDCl_3$, 25 ºC) spectrum of oblarotenoid A (**6**)

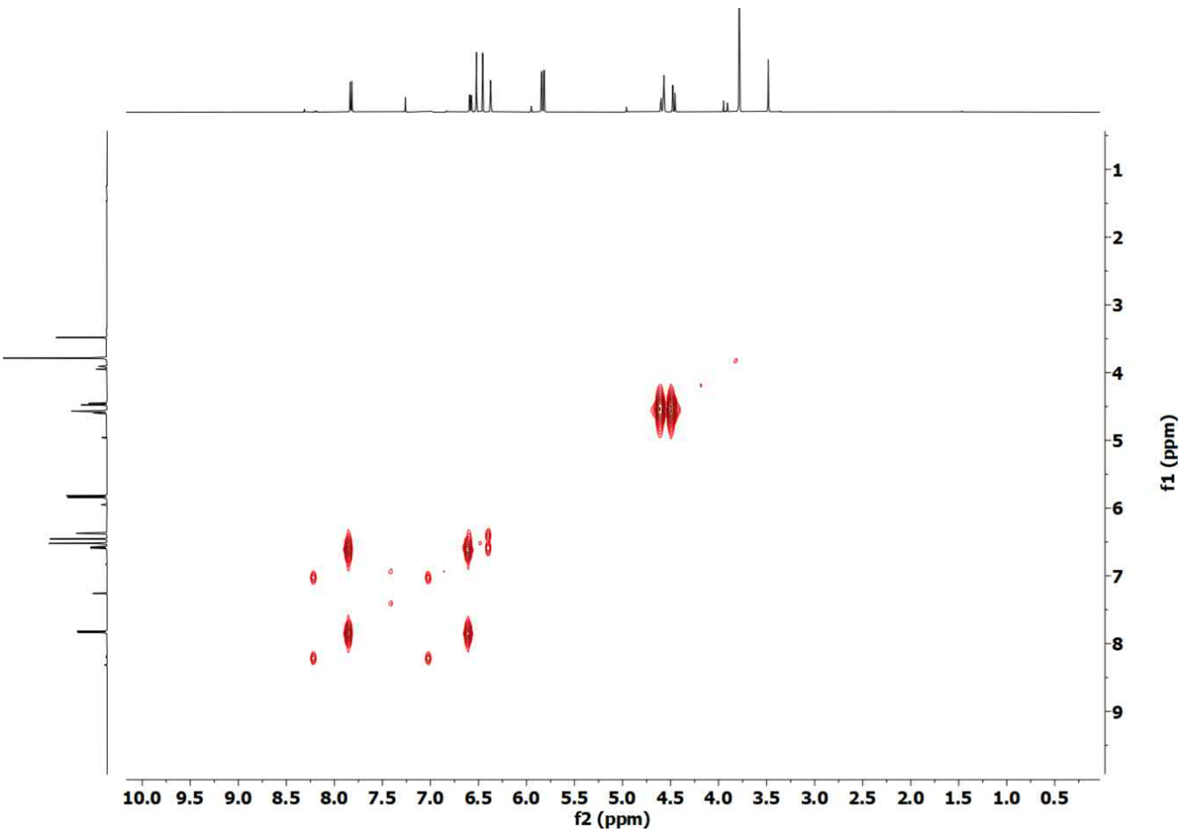

Figure S48. COSY (500 MHz, $CDCl_3$, 25 ºC) spectrum of oblarotenoid A (**6**).

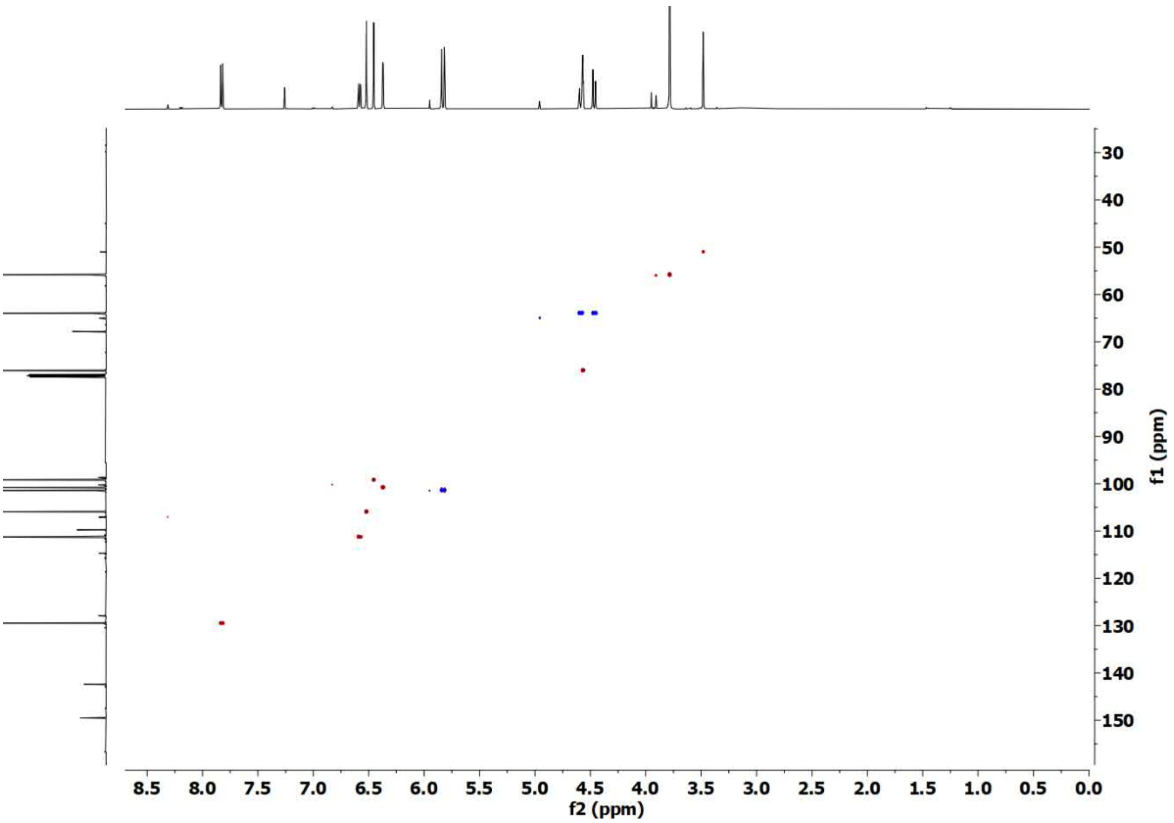

Figure S49. HSQC (500/125 MHz, $CDCl_3$, 25 ºC) spectrum of oblarotenoid A (**6**).

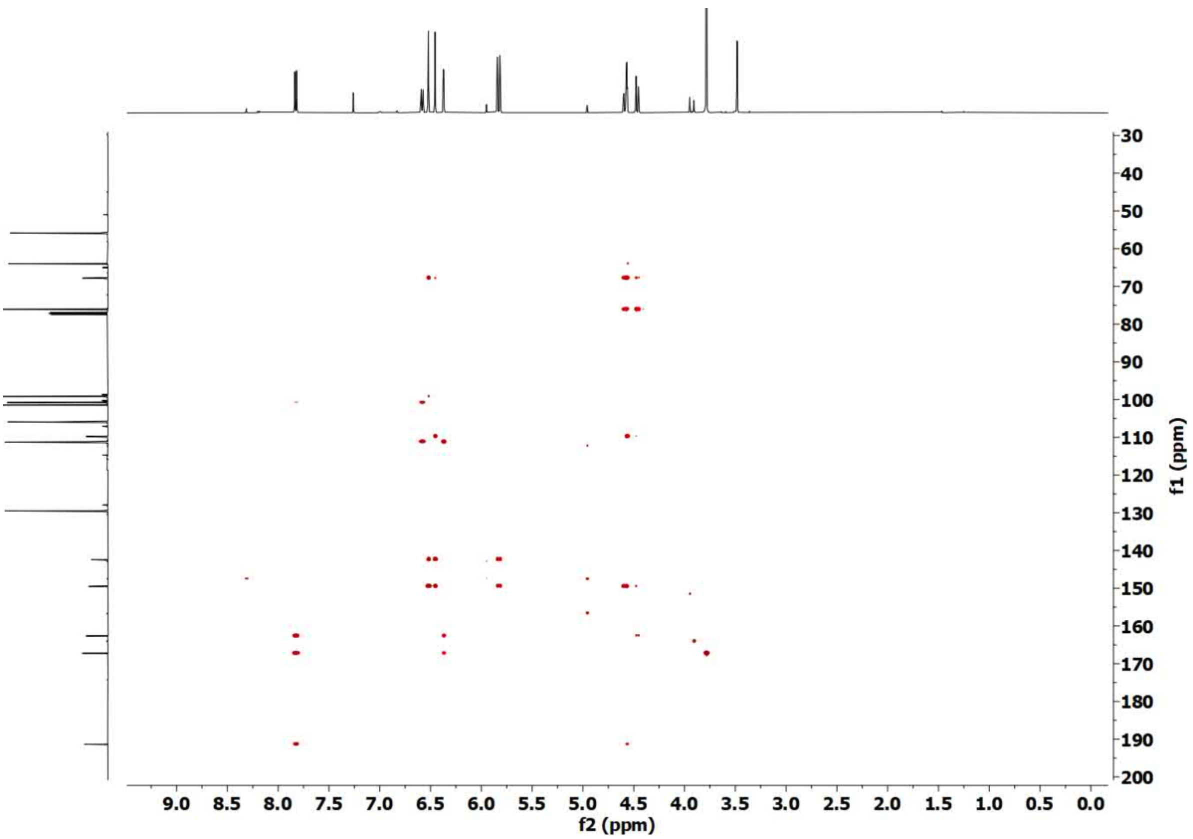

Figure S50. HMBC (500/125 MHz, $CDCl_3$, 25 ºC) spectrum of oblarotenoid A (**6**).

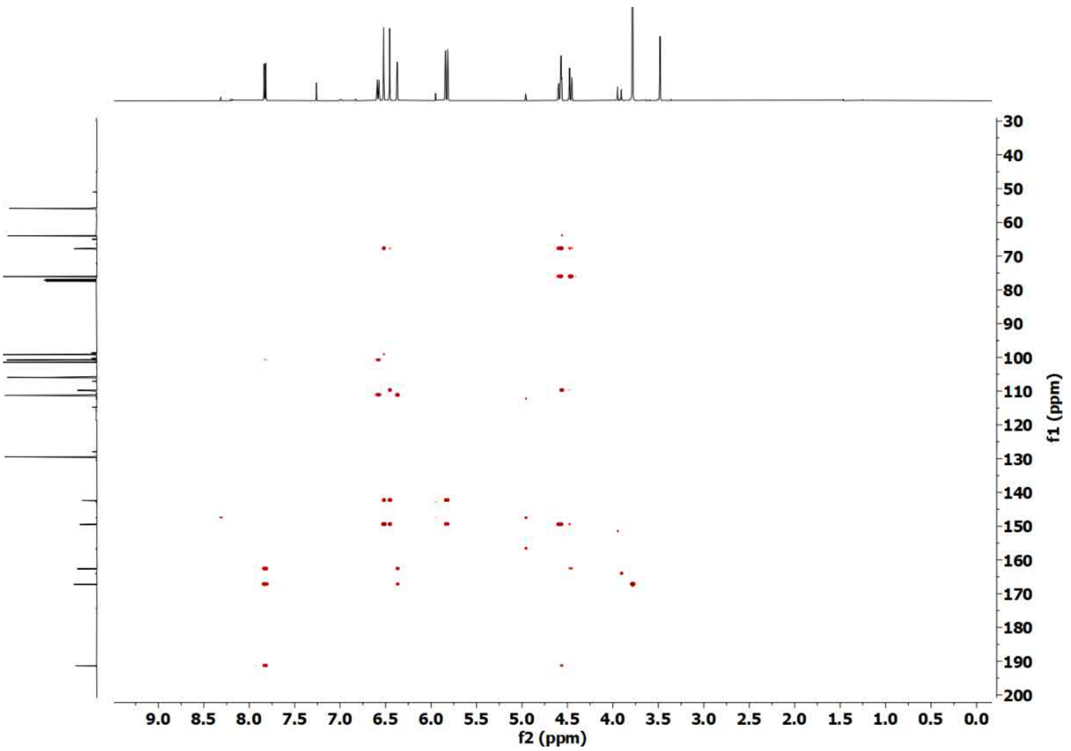

Figure S51. NOESY (500 MHz, $CDCl_3$, 25 ºC) spectrum of oblarotenoid A (**6**)

Spectroscopic Data of Oblarotenoid D (7)

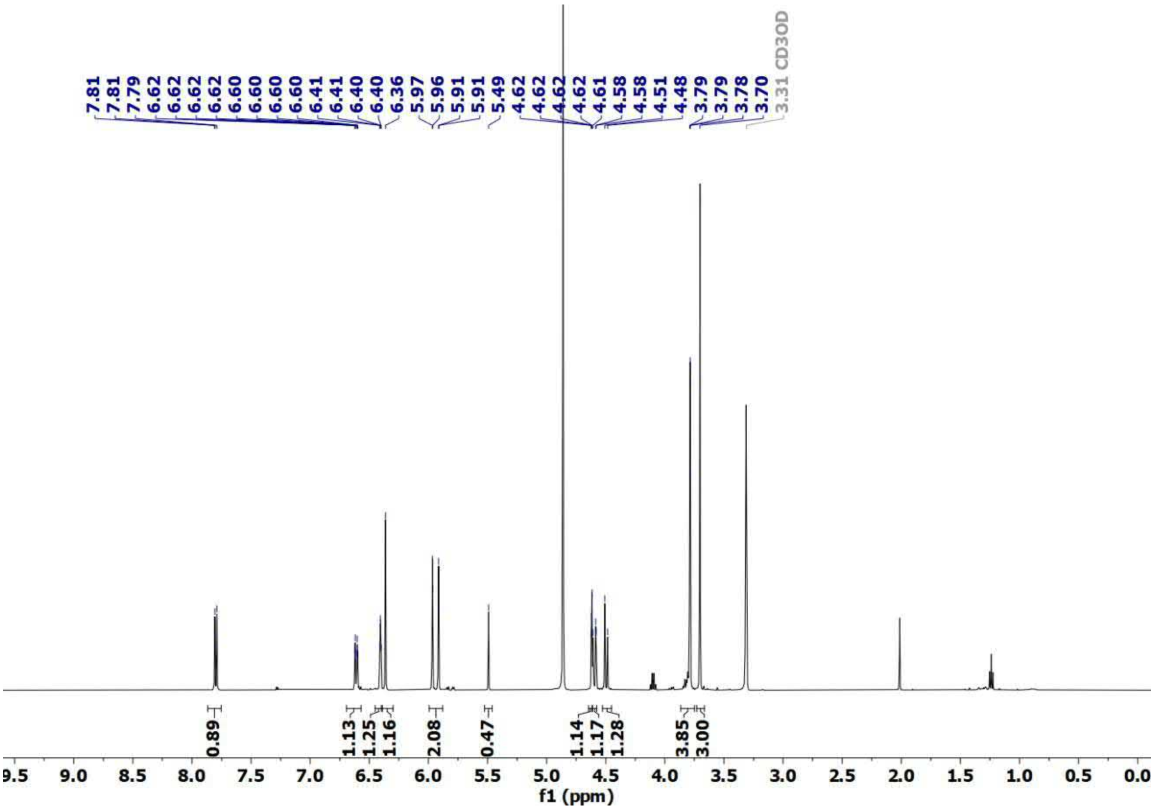

Figure S52. ^{1}H NMR (500 MHz, MeOD-d_4, 25 ºC) spectrum of oblarotenoid D (**7**)

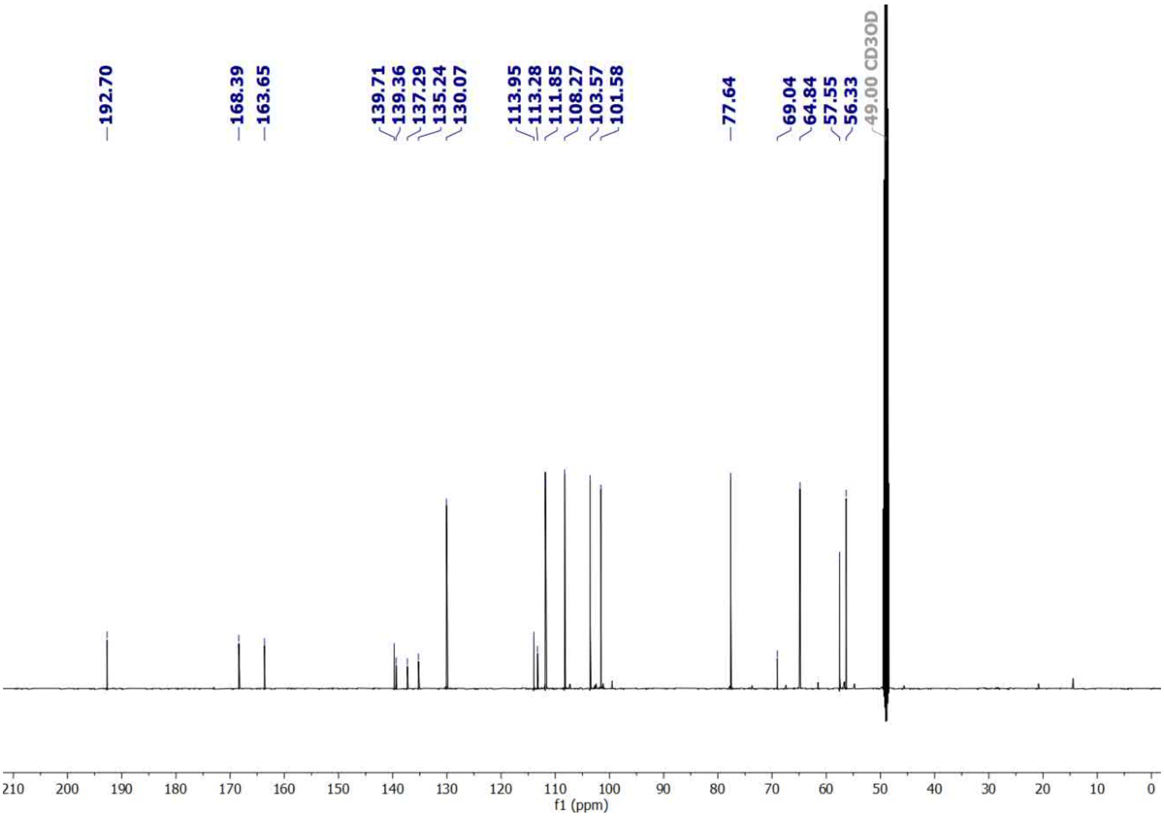

Figure S53. ^{13}C NMR (125 MHz, MeOD-d_4, 25 ºC) spectrum oblarotenoid D (**7**)

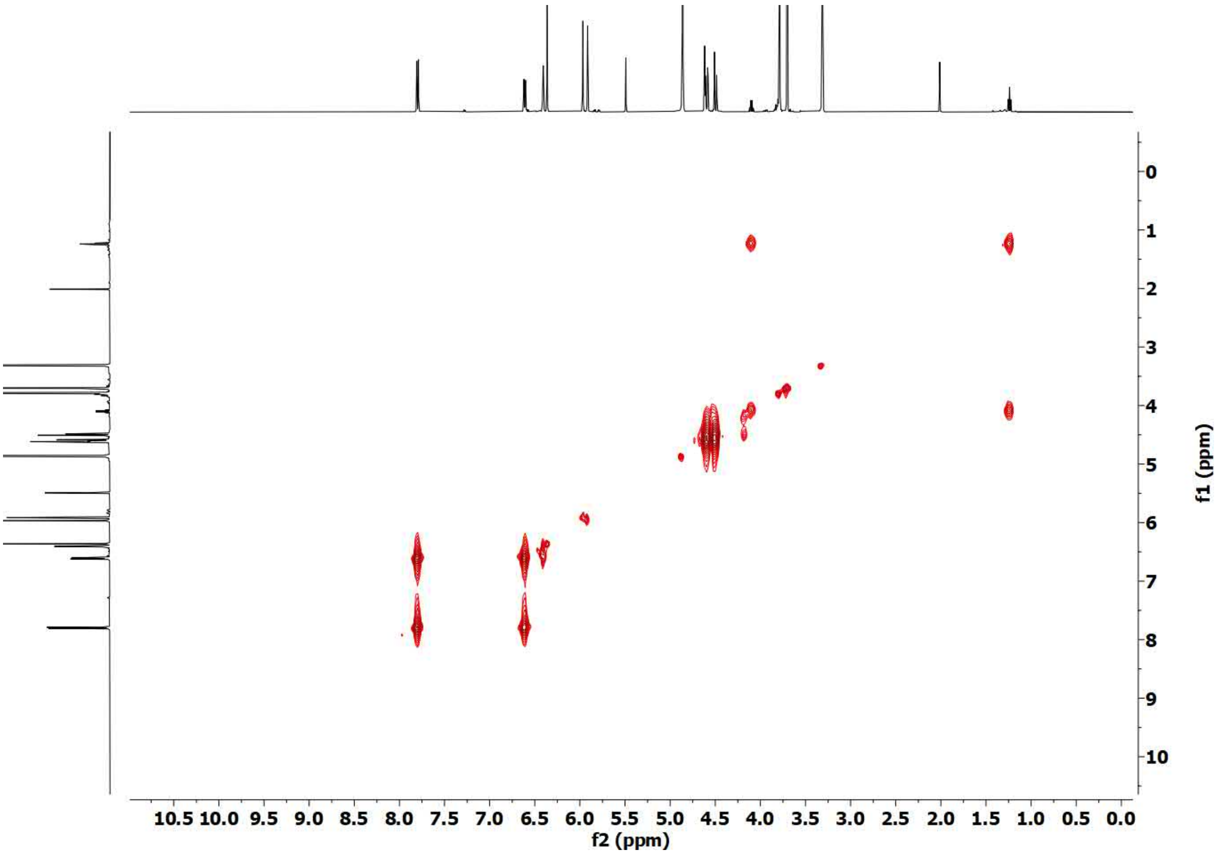

Figure S54. COSY (500 MHz, MeOD-d$_4$, 25 ºC) spectrum oblarotenoid D (7)

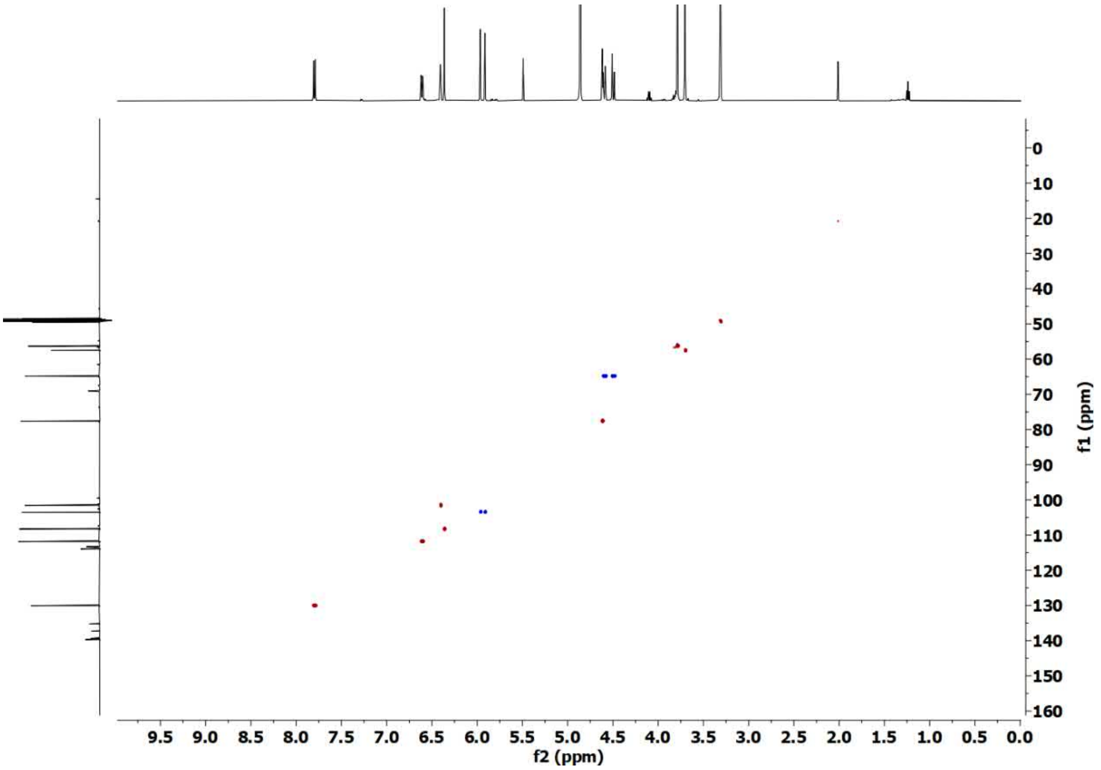

Figure S55. HSQC (500/125 MHz, MeOD-d$_4$, 25 ºC) spectrum oblarotenoid D (**7**)

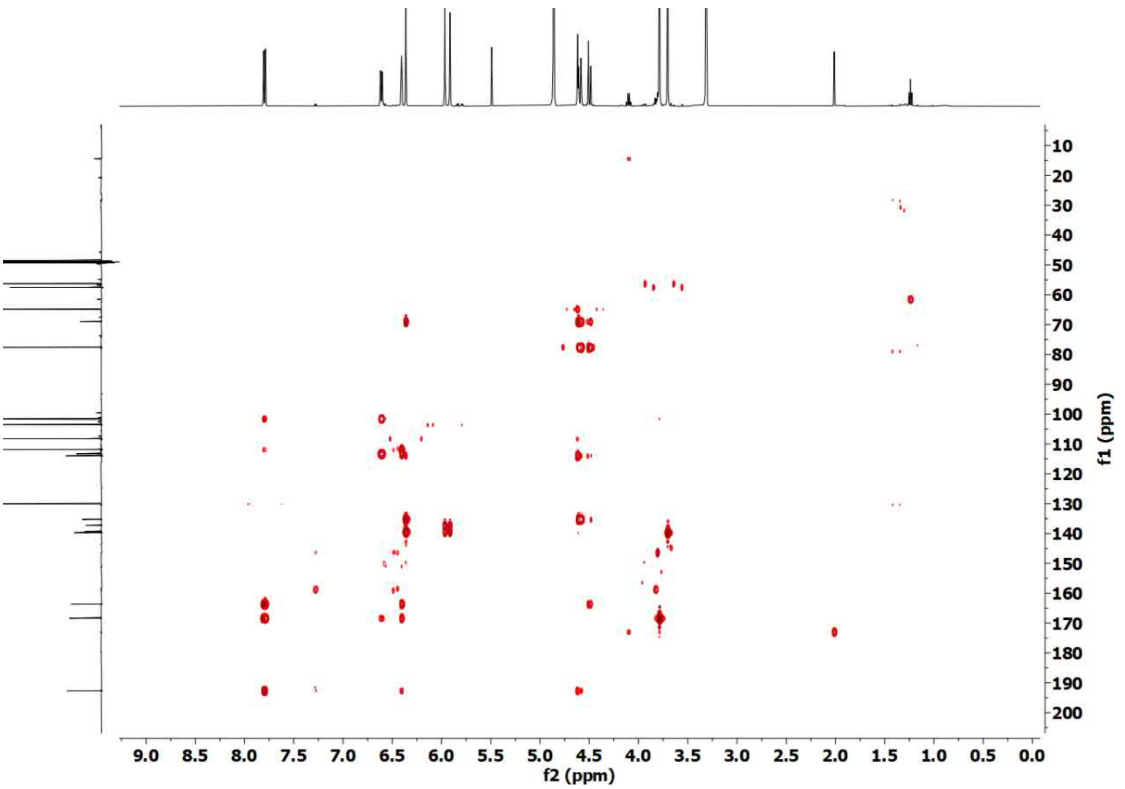

Figure S56. HMBC (500/125 MHz, MeOD-d_4, 25 ºC) spectrum oblarotenoid D (**7**)

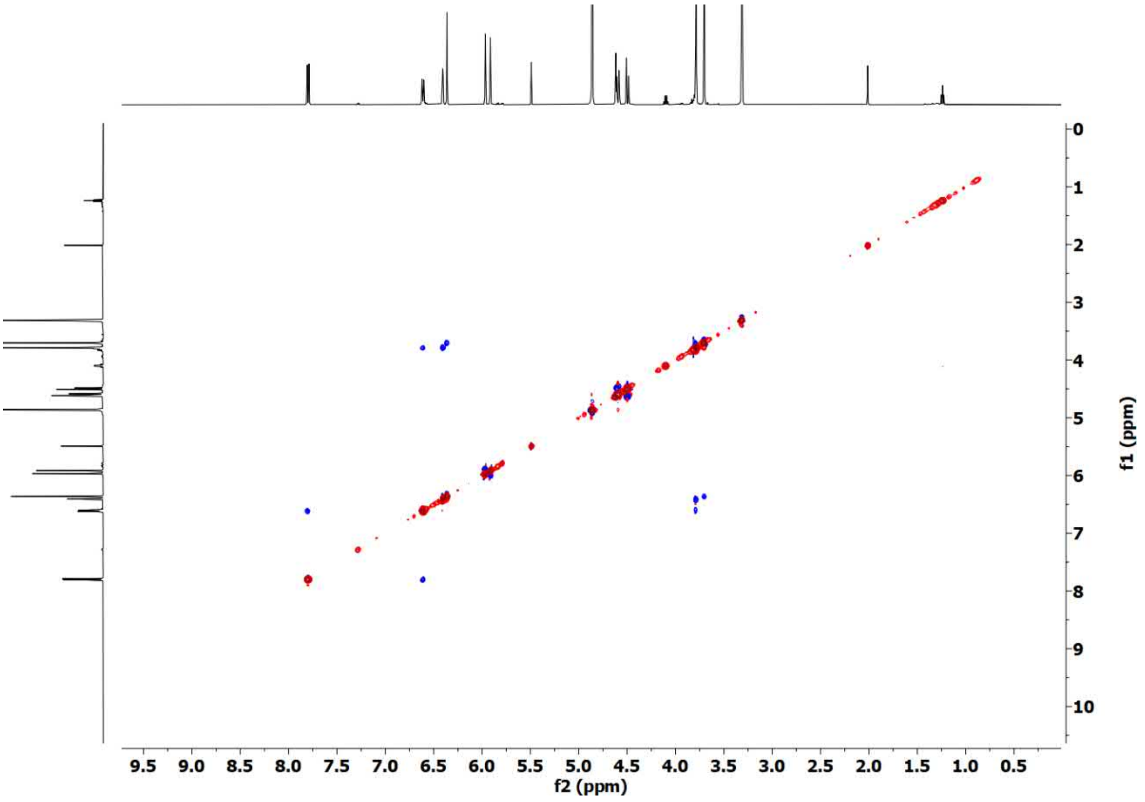

Figure S57. NOESY (500 MHz, MeOD-d_4, 25 ºC) spectrum oblarotenoid D (**7**)

Spectroscopic Data of 12a-Hydroxymunduserone (8)

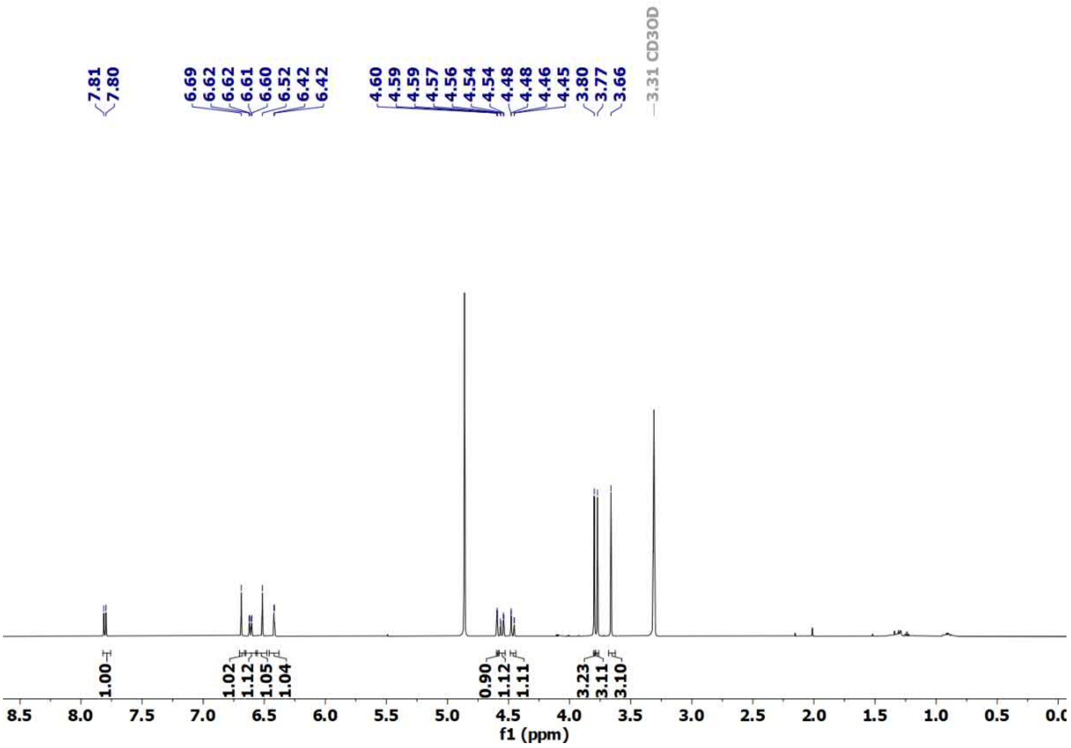

Figure S58. ^{1}H NMR (500 MHz, MeOD-d4, 25 ºC) spectrum of 12a-hydroxymunduserone (**8**)

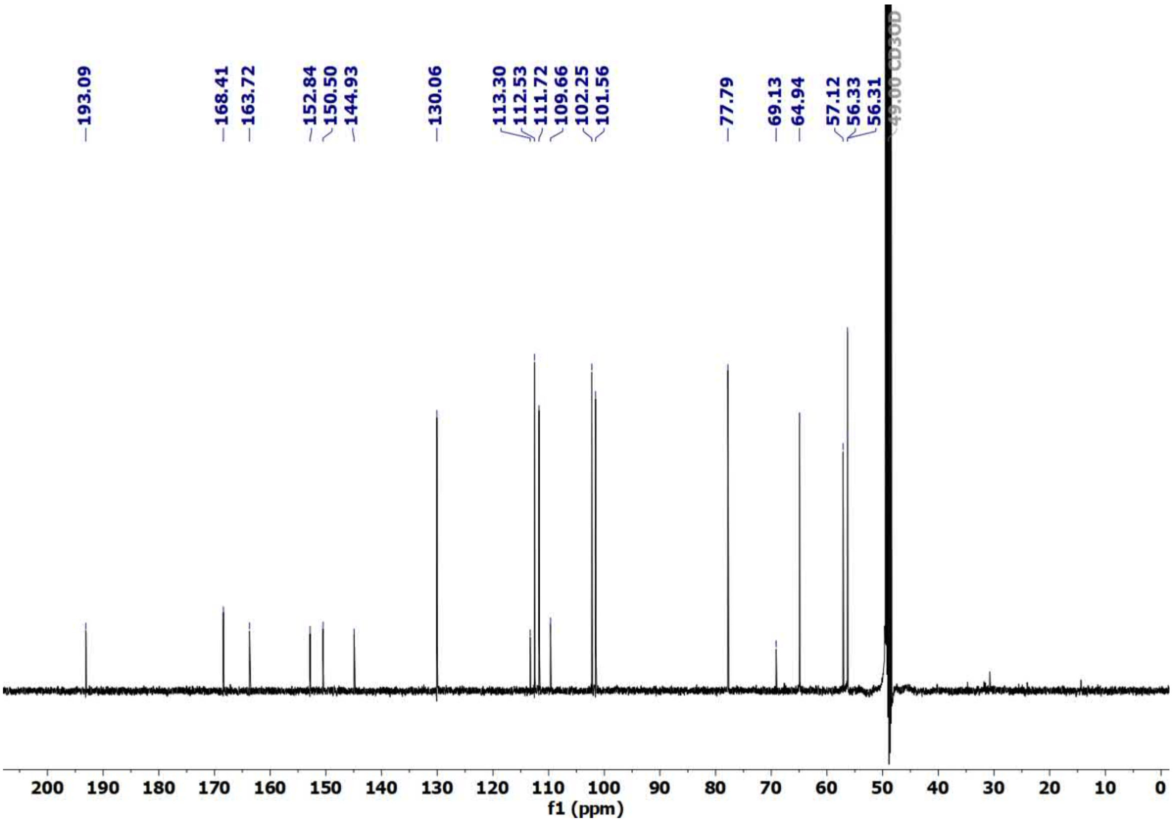

Figure S59. ^{13}C NMR (125 MHz, MeOD-d_4, 25 ºC) spectrum of 12a-hydroxymunduserone (**8**)

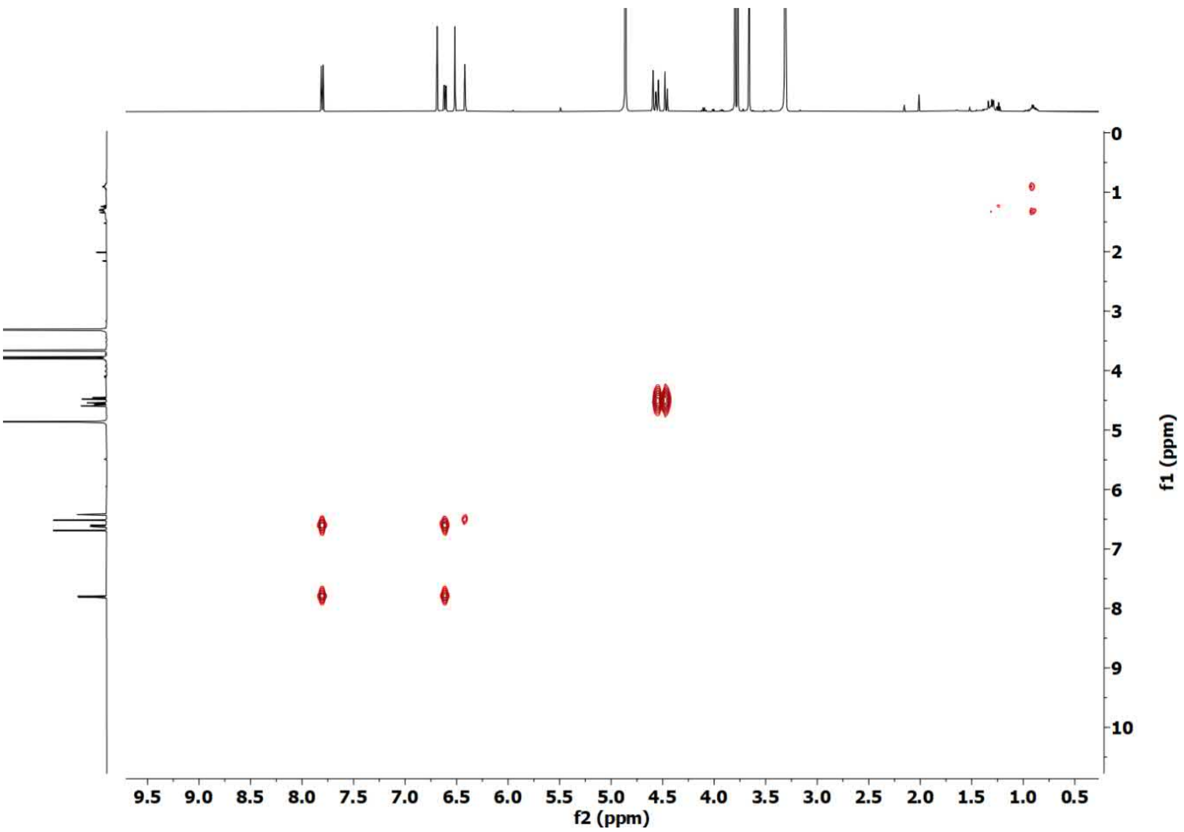

Figure S60. COSY (500 MHz, MeOD-d$_4$, 25 ºC) spectrum of 12a-hydroxymunduserone (**8**)

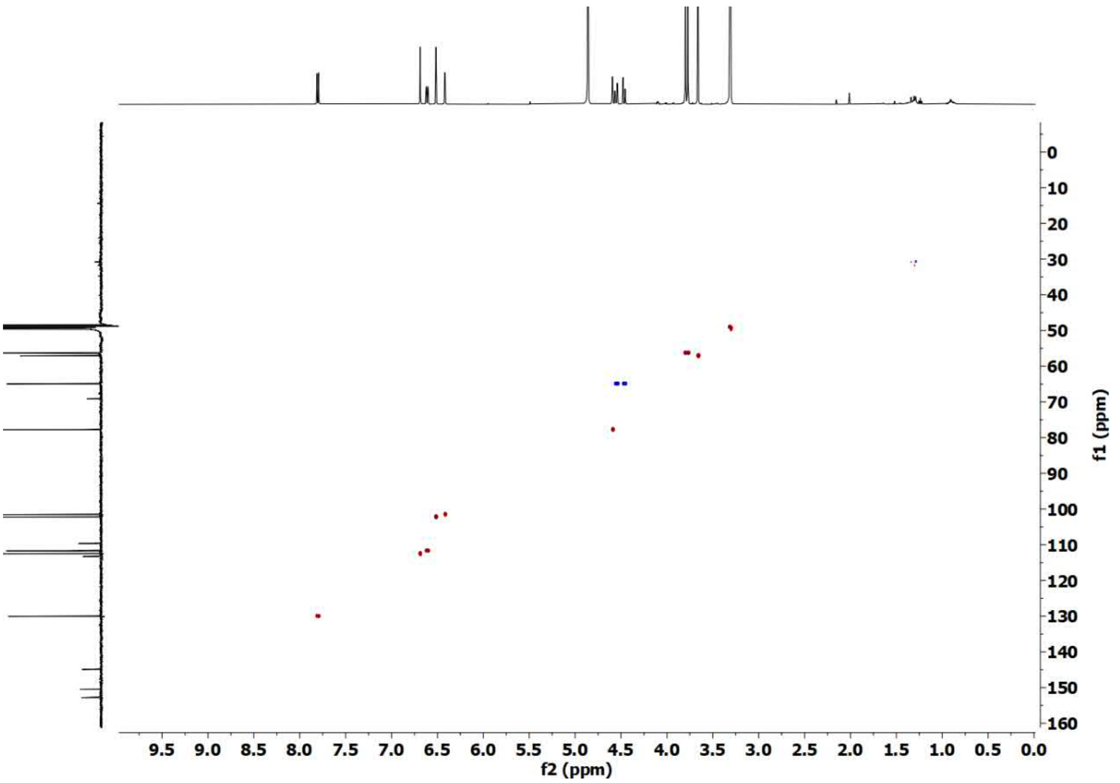

Figure S61. HSQC (500/125 MHz, MeOD-d$_4$, 25 ºC) spectrum of 12a-hydroxymunduserone (**8**)

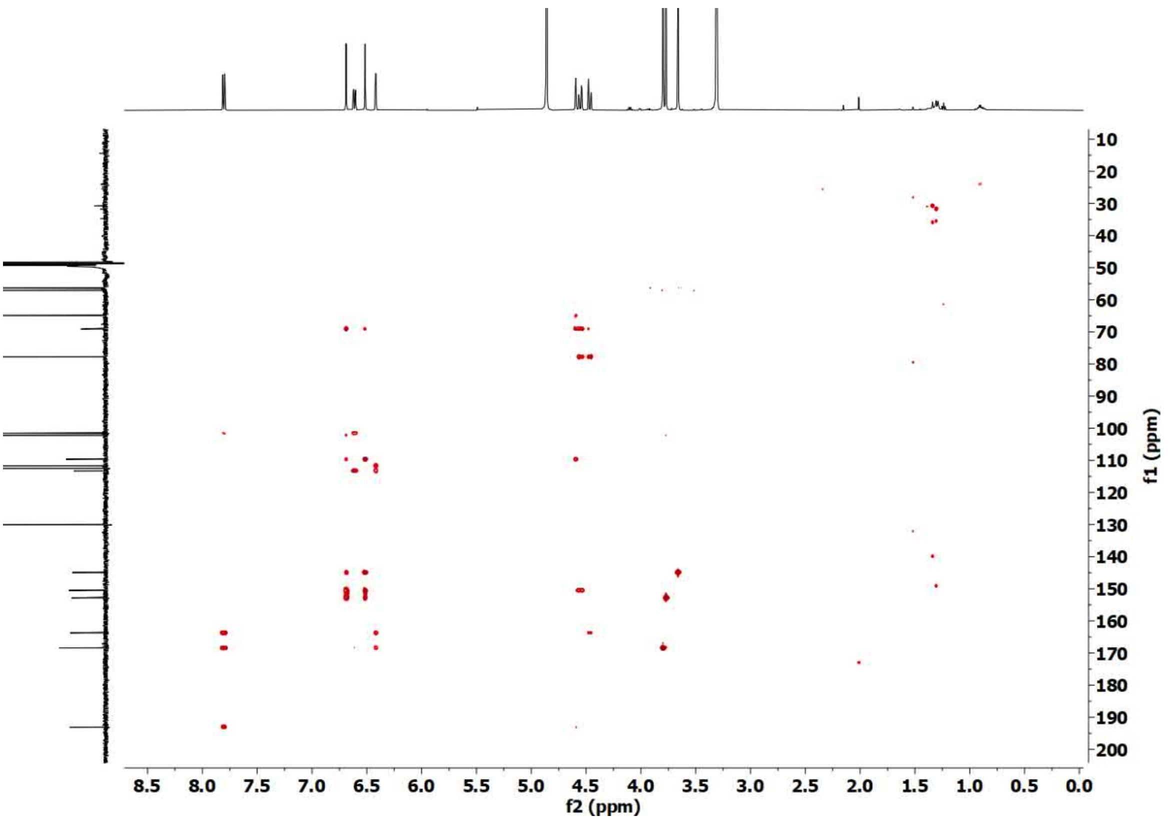

Figure S62. HMBC (500/125 MHz, MeOD-d4, 25 ºC) spectrum of 12a-hydroxymunduserone (**8**)

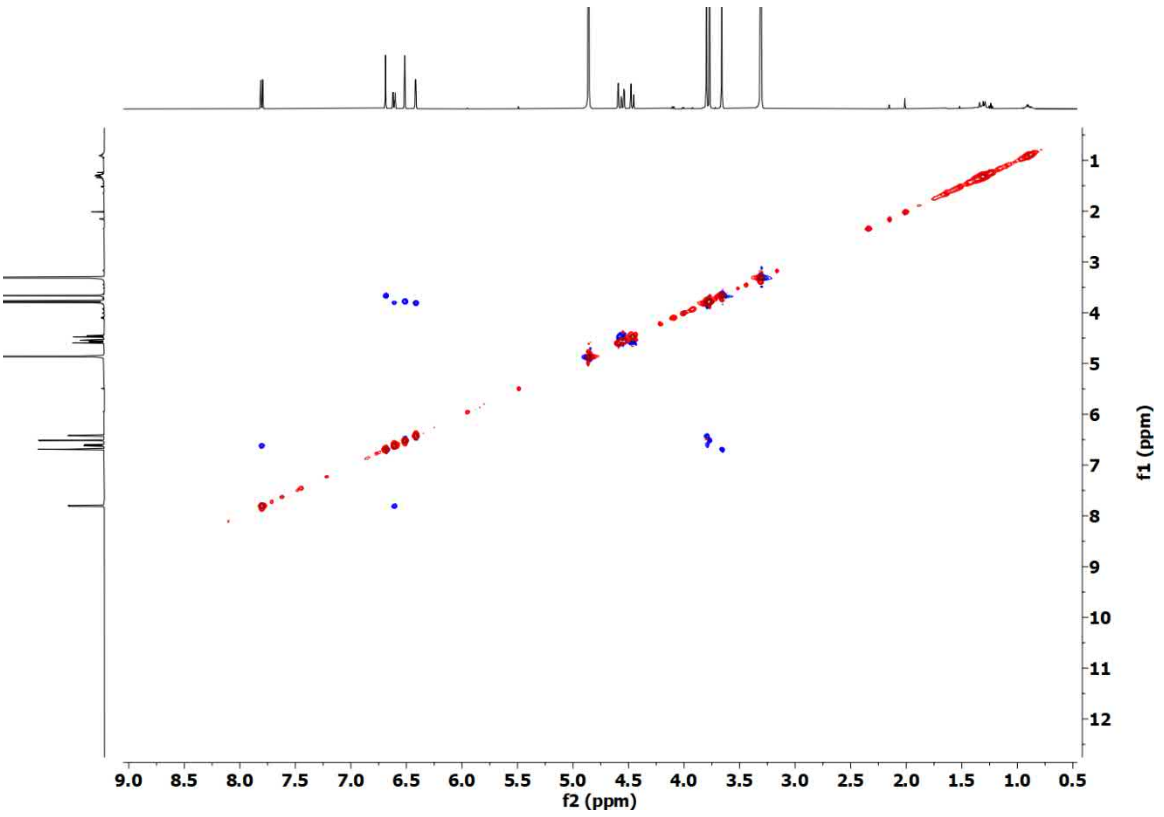

Figure S63. NOESY (500 MHz, MeOD-d4, 25 ºC) spectrum of 12a-hydroxymunduserone (**8**)

Spectroscopic Data of Tephrosin (9)

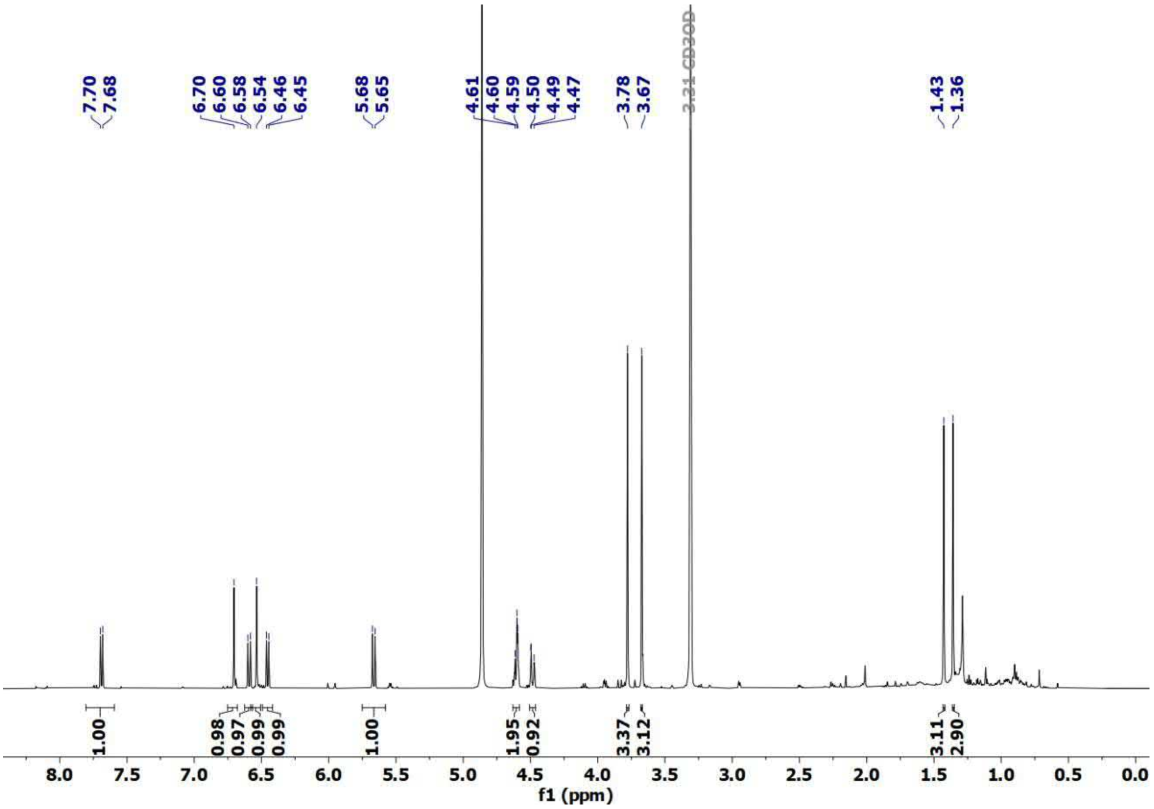

Figure S64. ^{1}H NMR (500 MHz, MeOD-d4, 25 ºC) spectrum of tephrosin (**9**)

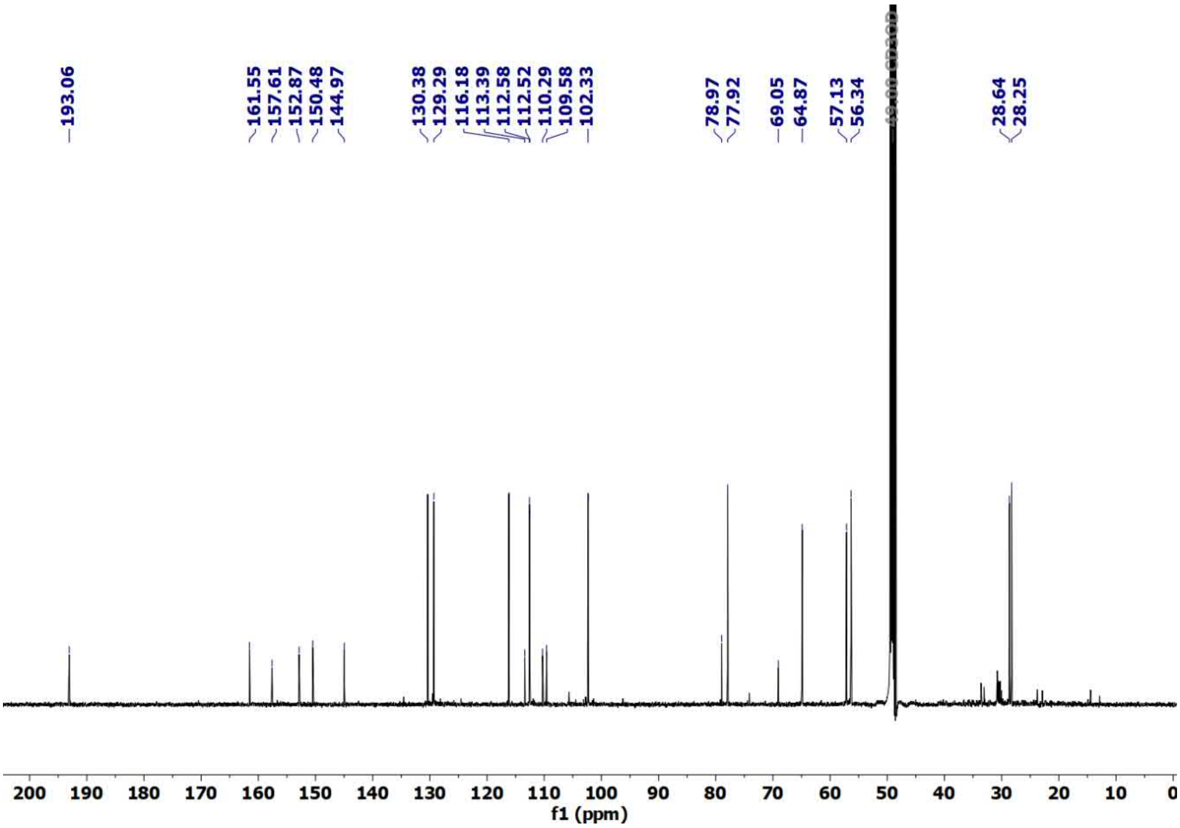

Figure S65. ^{13}C NMR (125 MHz, MeOD-d4, 25 ºC) spectrum of tephrosin (**9**)

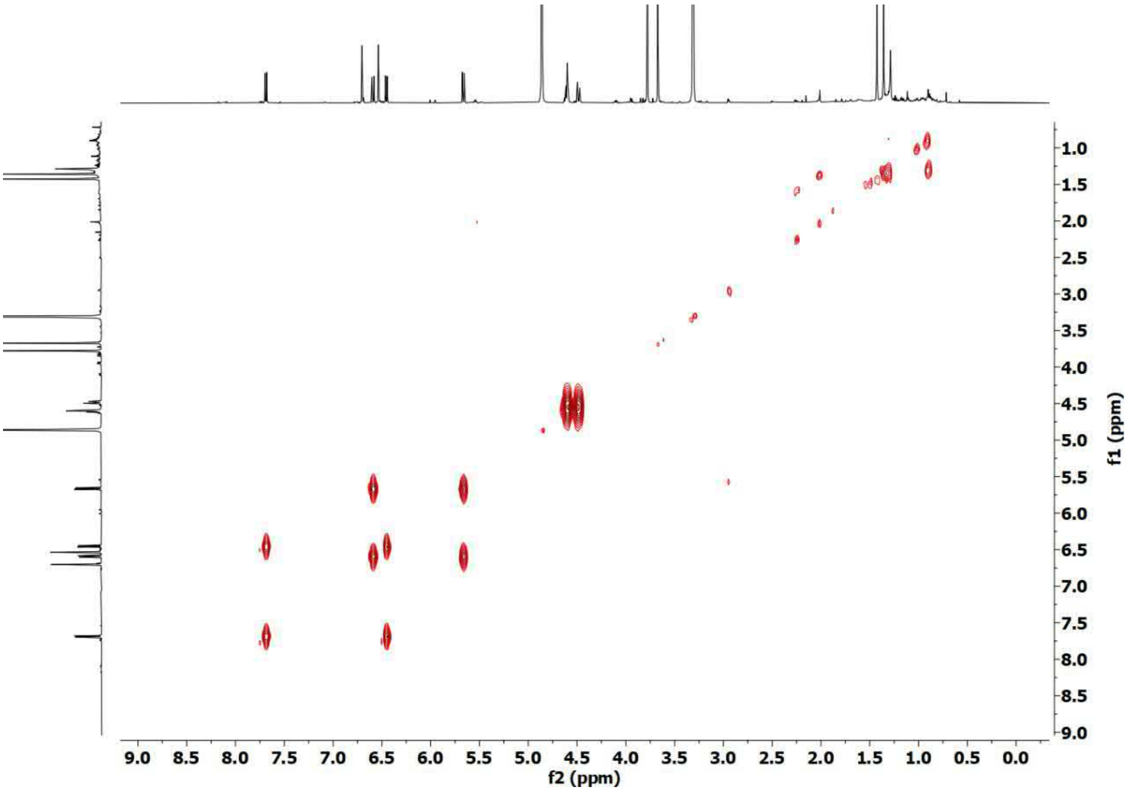

Figure S66. COSY (500 MHz, MeOD-d4, 25 ºC) spectrum of tephrosin (**9**).

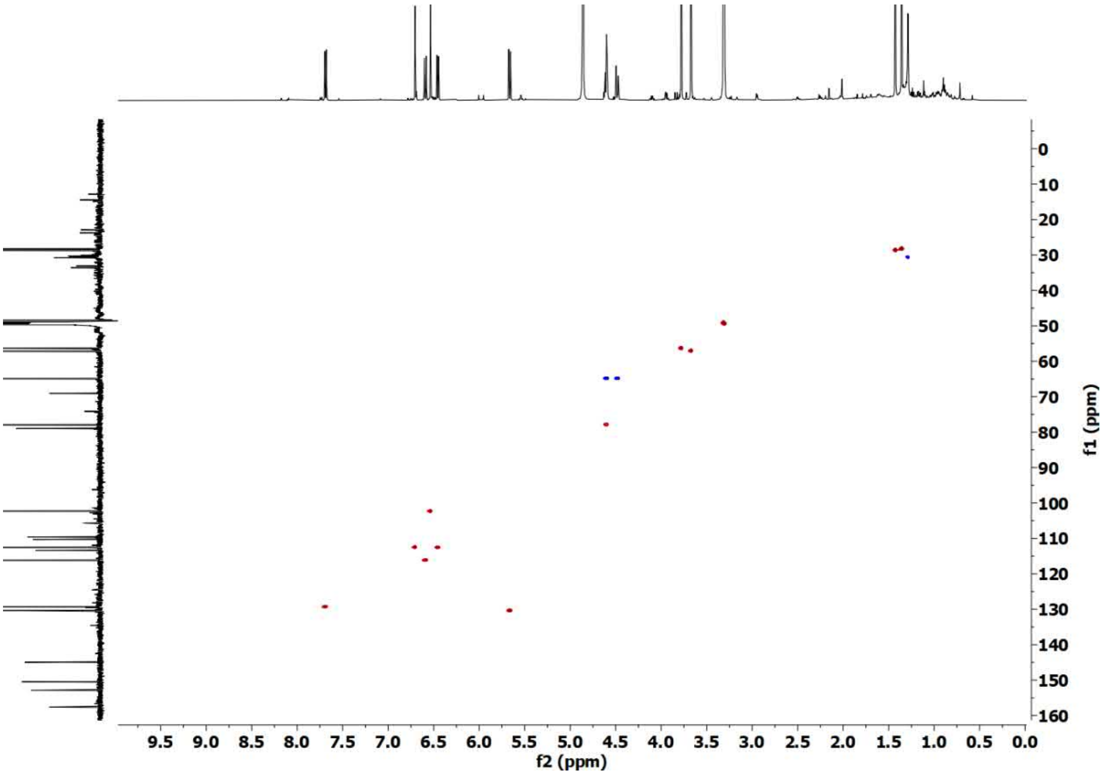

Figure S67. HSQC (500/125 MHz, MeOD-d4, 25 ºC) spectrum of tephrosin (**9**).

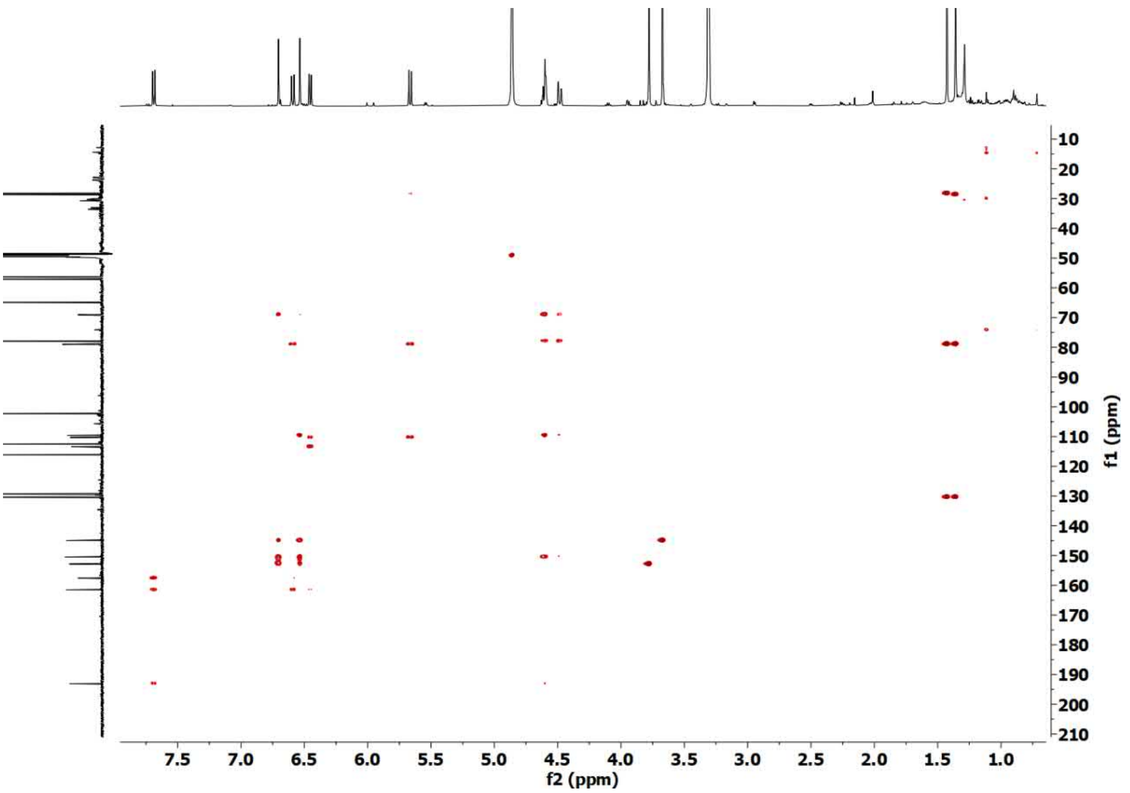

Figure S68. HMBC (500/125 MHz, MeOD-d4, 25 ºC) spectrum of tephrosin (**9**).

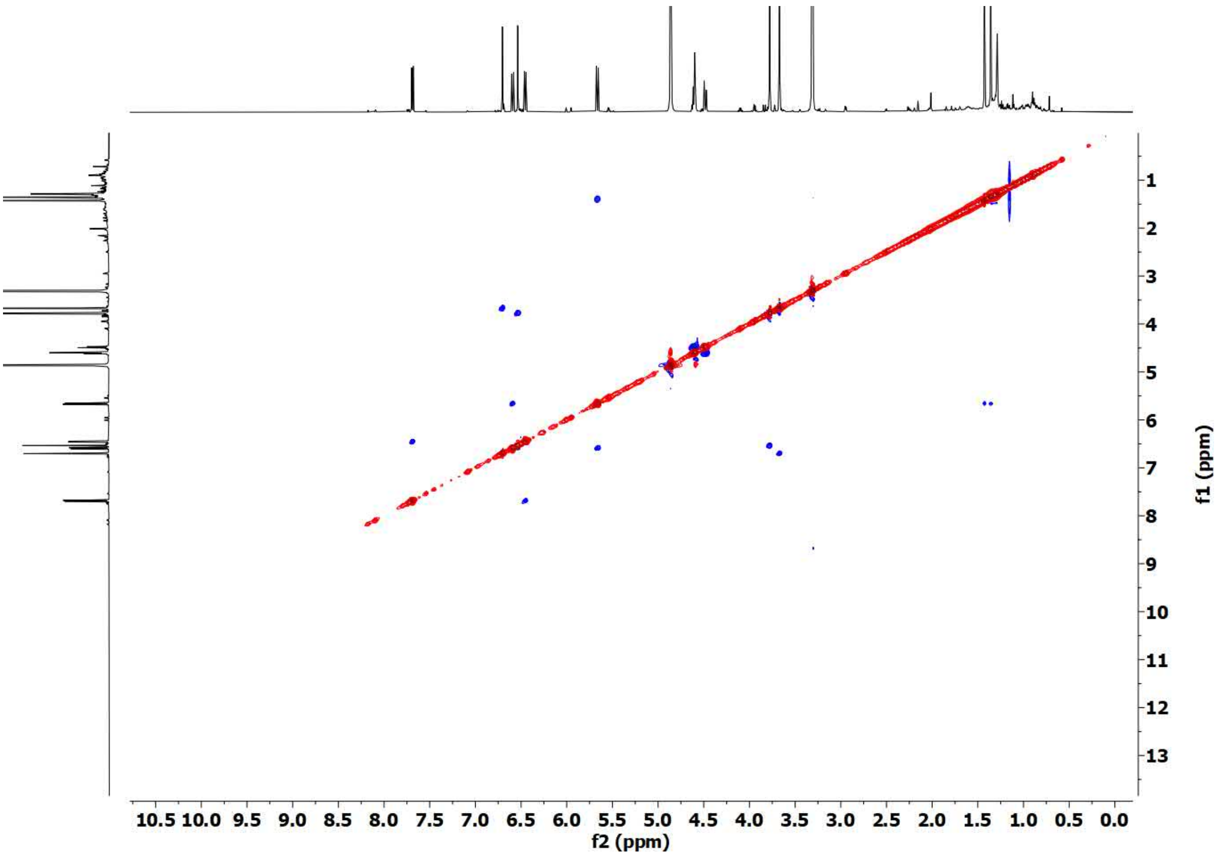

Figure S69. NOESY (500 MHz, MeOD-d4, 25 ºC) spectrum of tephrosin (**9**).

Spectroscopic Data of Deguelin (10)

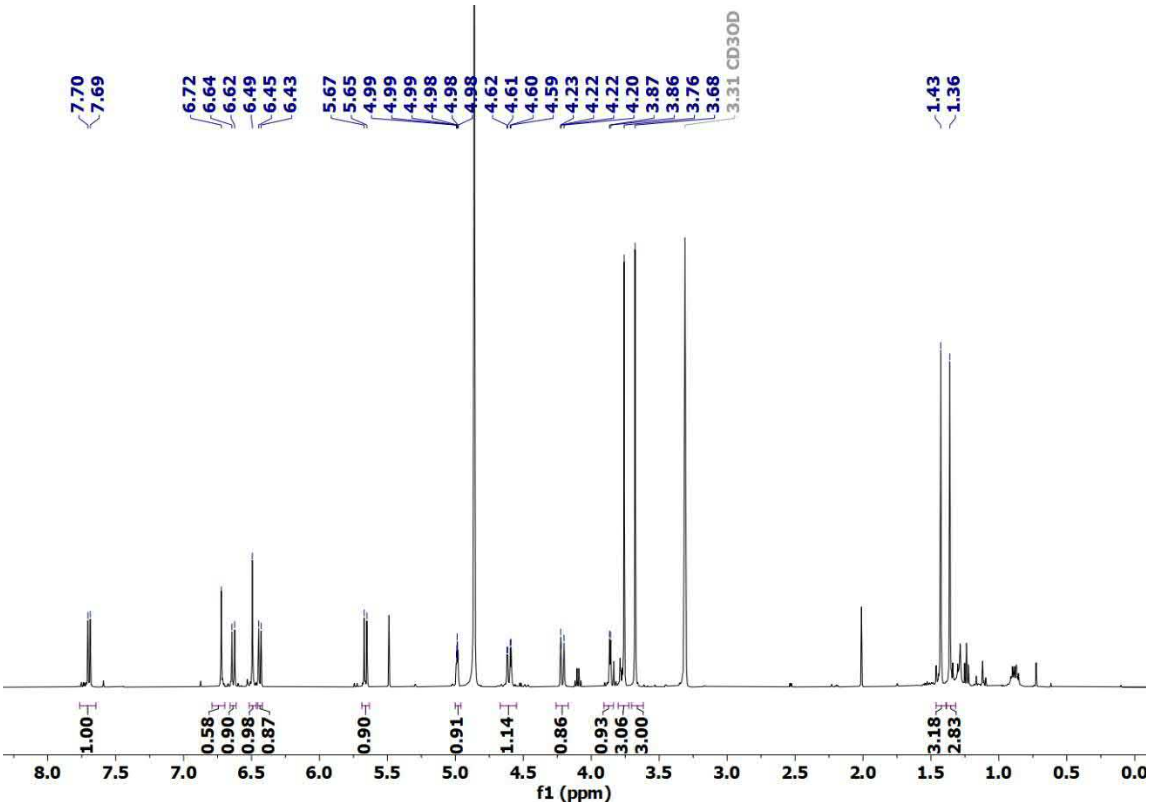

Figure S70. ^{1}H NMR (500 MHz, MeOD-d4, 25 ºC) spectrum of deguelin (**10**)

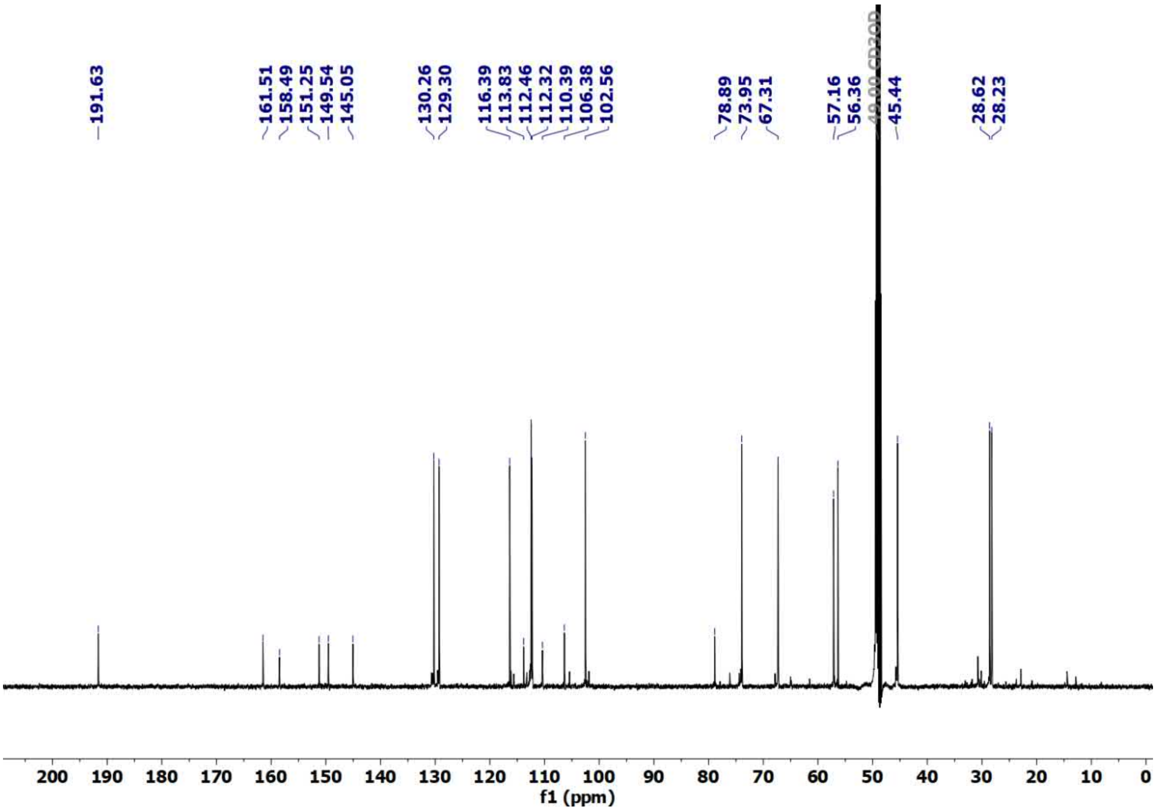

Figure S71. ^{13}C NMR (125 MHz, MeOD-d_4, 25 ºC) spectrum of deguelin (**10**)

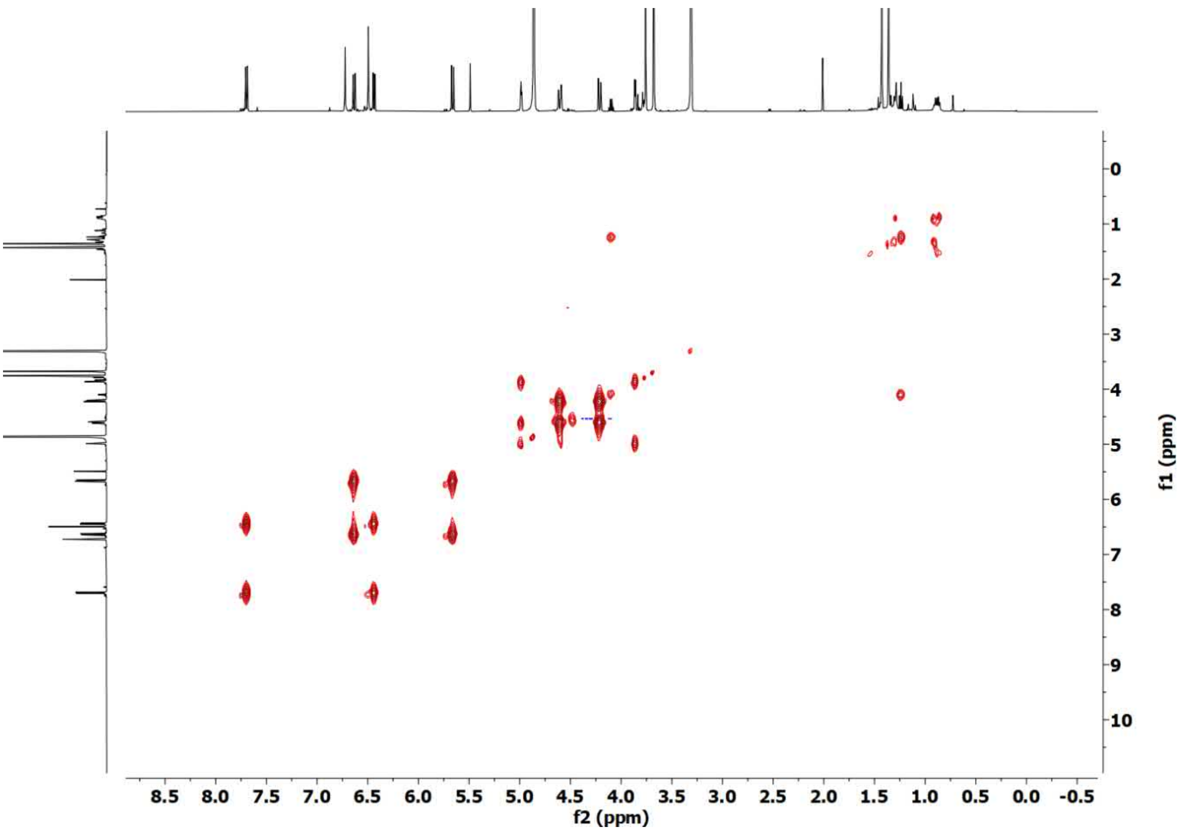

Figure S72. COSY (500 MHz, MeOD-d4, 25 ºC) spectrum of deguelin (**10**)

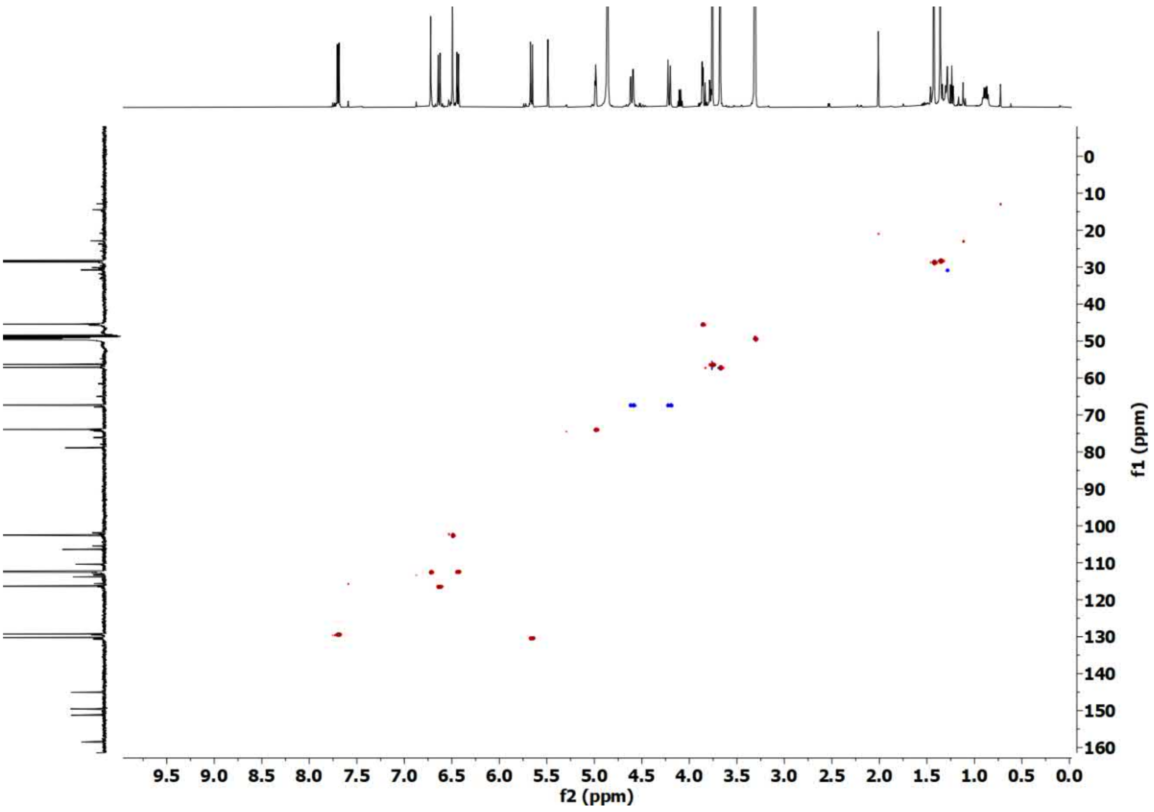

Figure S73. HSQC (500/125 MHz, MeOD-d_4, 25 ºC) spectrum of deguelin (**10**)

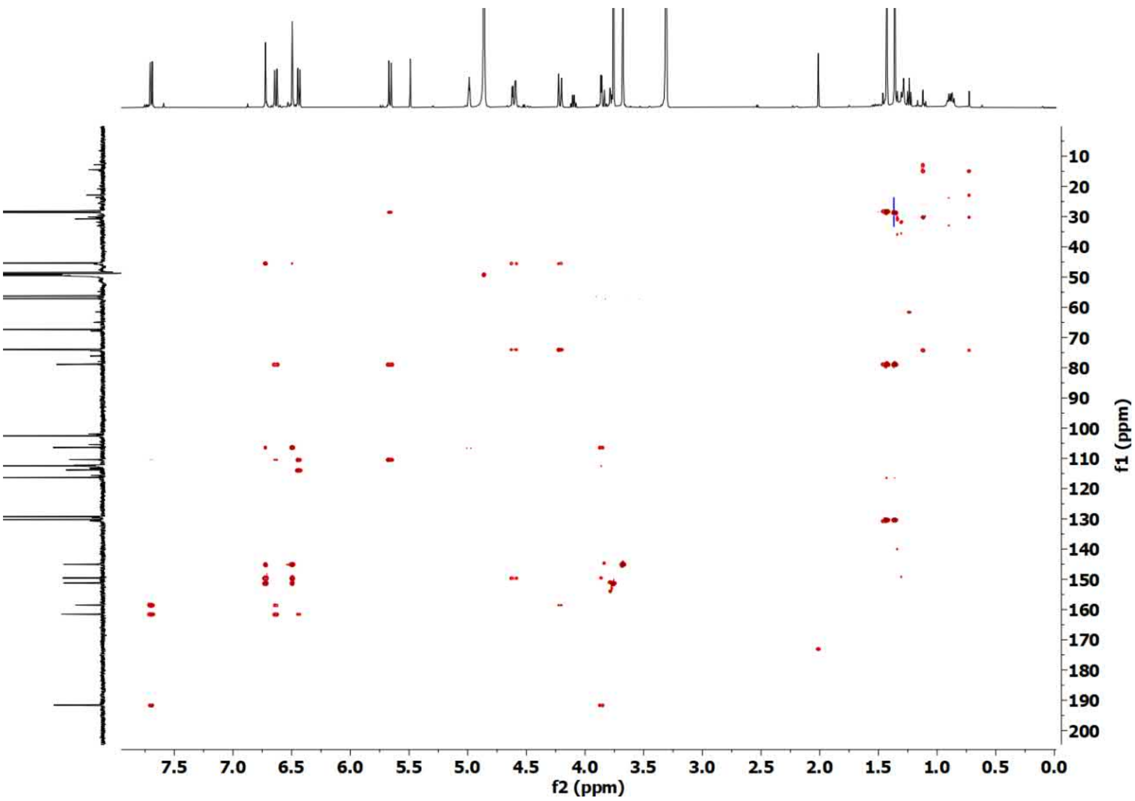

Figure S74. HMBC (500/125 MHz, MeOD-d_4, 25 ºC) spectrum of deguelin (**10**).

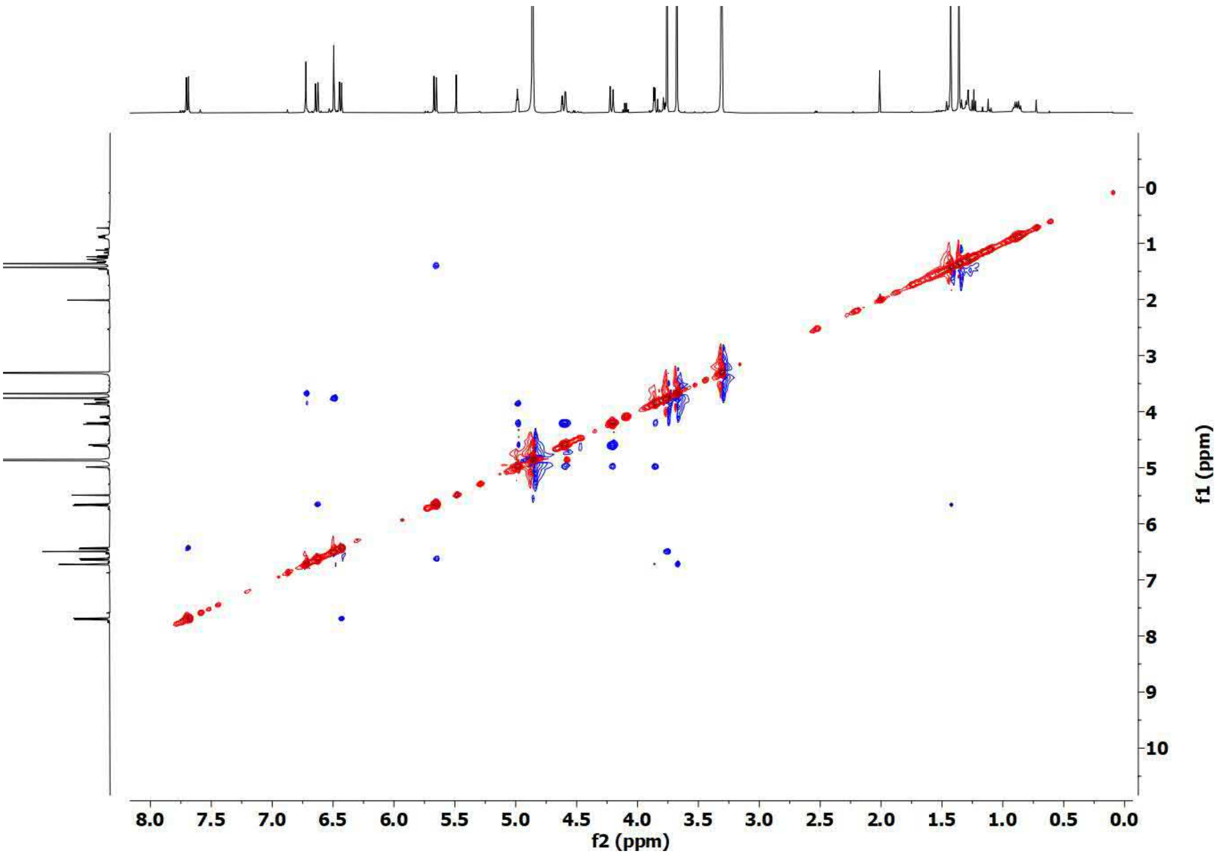

Figure S75. NOESY (500 MHz, MeOD-d_4, 25 ºC) spectrum of deguelin (**10**).

Spectroscopic Data of Ichthynone (11)

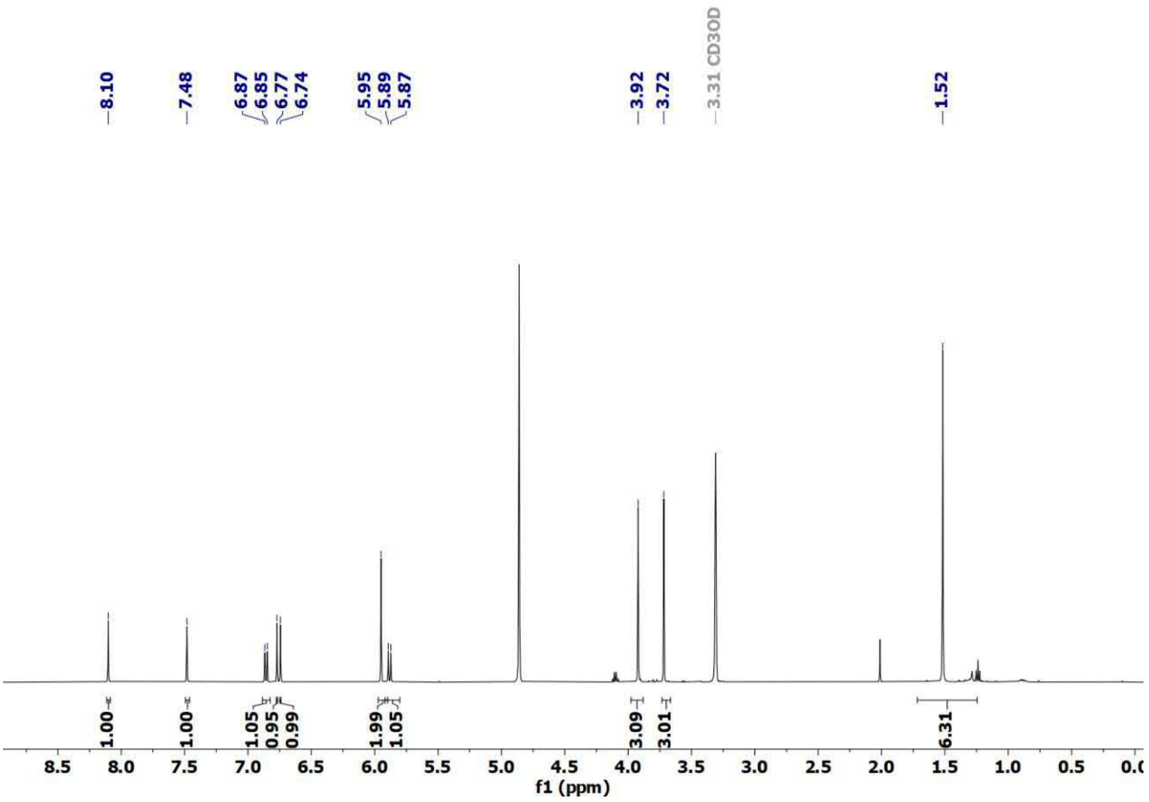

Figure S76. ^{1}H NMR (500 MHz, MeOD-d4, 25 ºC) spectrum of ichthynone (**11**).

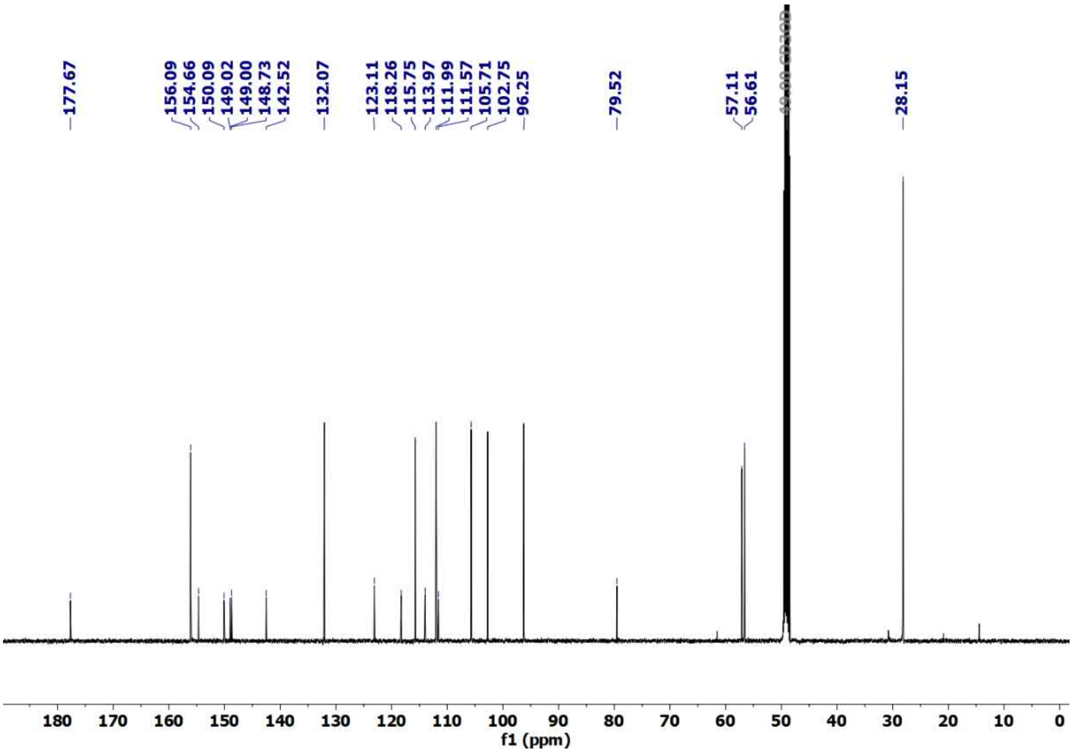

Figure S77. ^{13}C NMR (125 MHz, MeOD-d4, 25 ºC) spectrum of ichthynone (**11**).

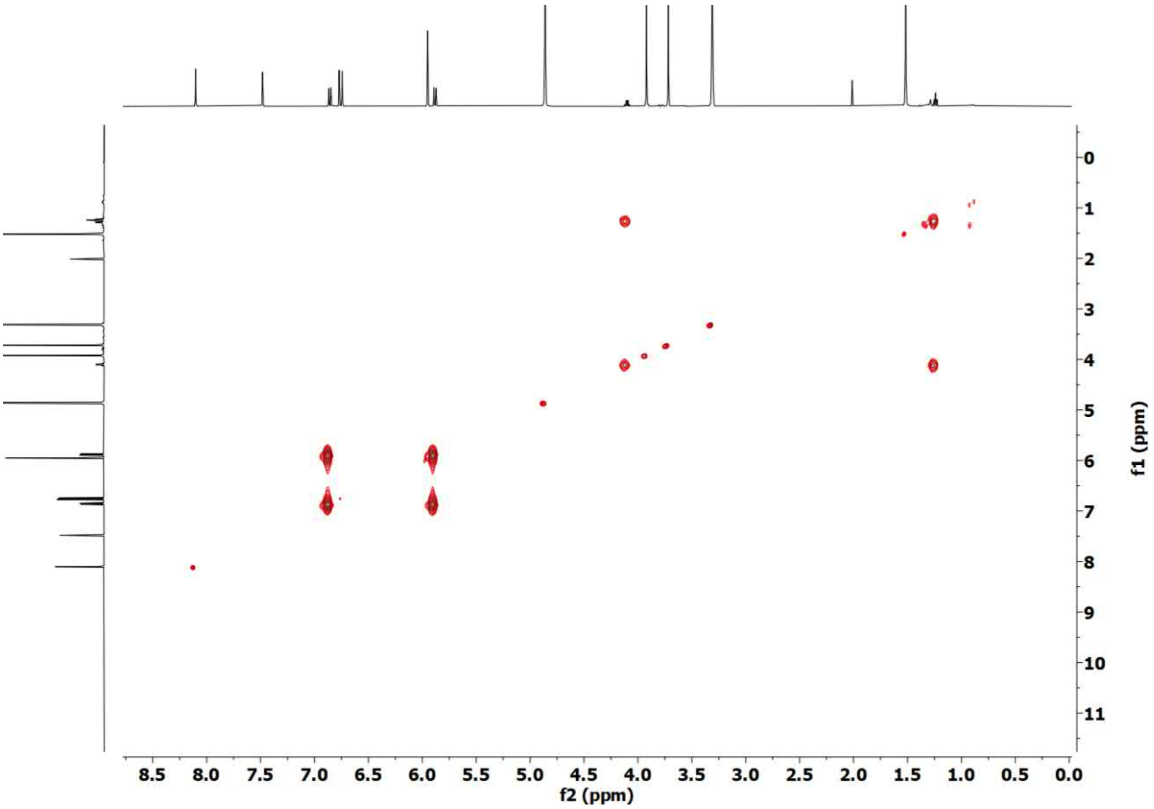

Figure S78. COSY (500 MHz, MeOD-d4, 25 ºC) spectrum of ichthynone (**11**).

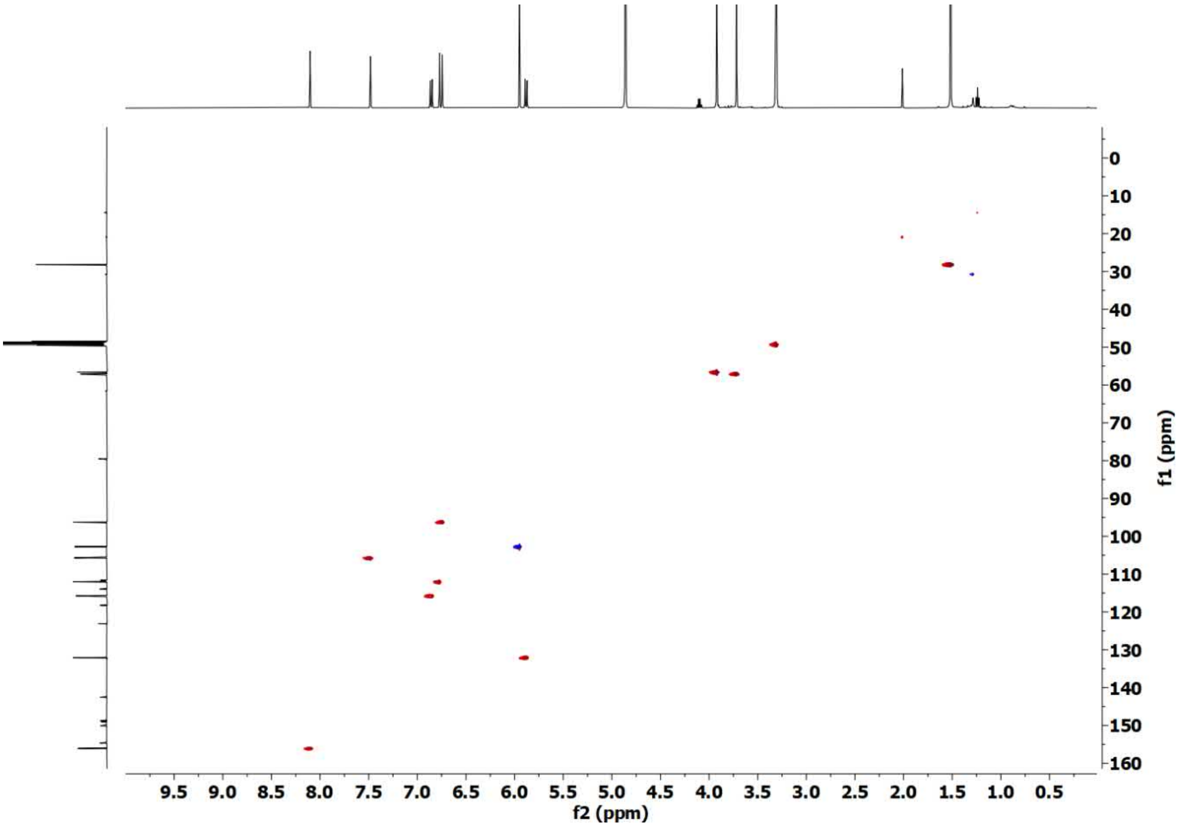

Figure S79. HSQC (500/125 MHz, MeOD-d4, 25 ºC) spectrum of ichthynone (**11**).

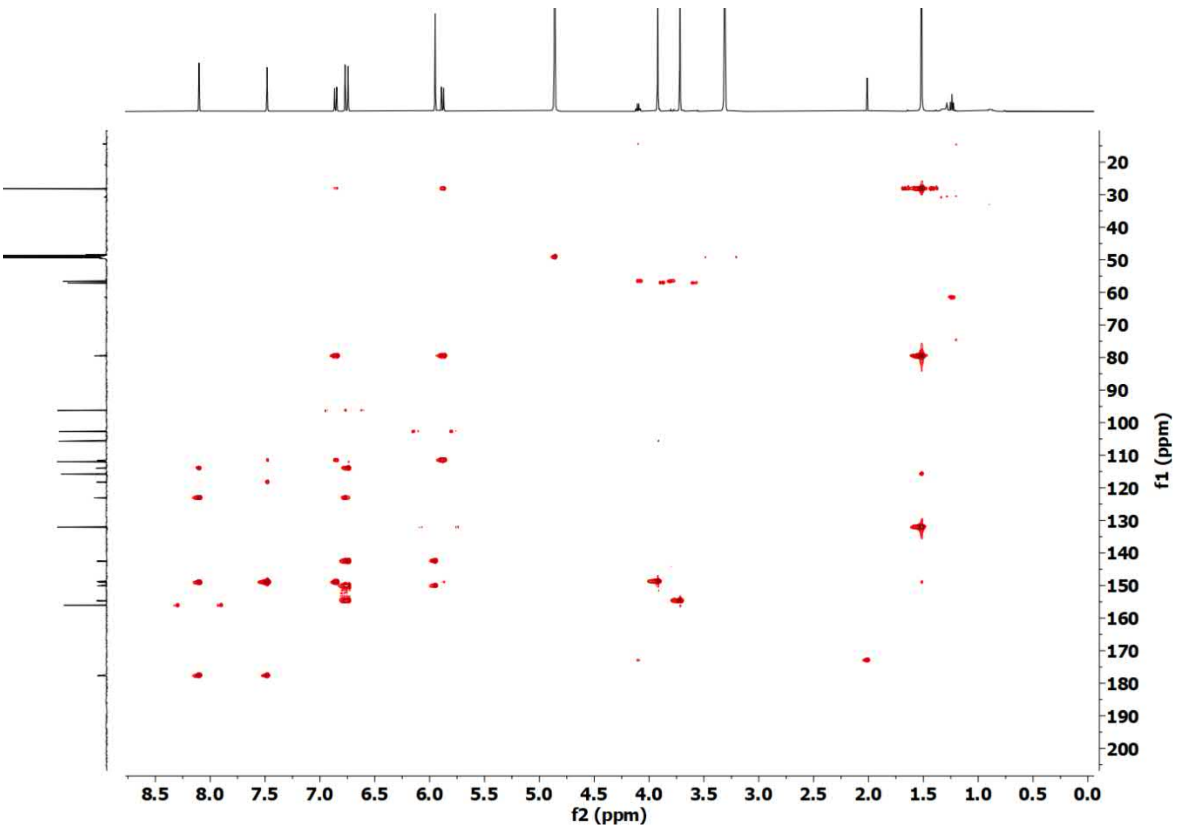

Figure S80. HMBC (500/125 MHz, MeOD-d4, 25 ºC) spectrum of ichthynone (**11**).

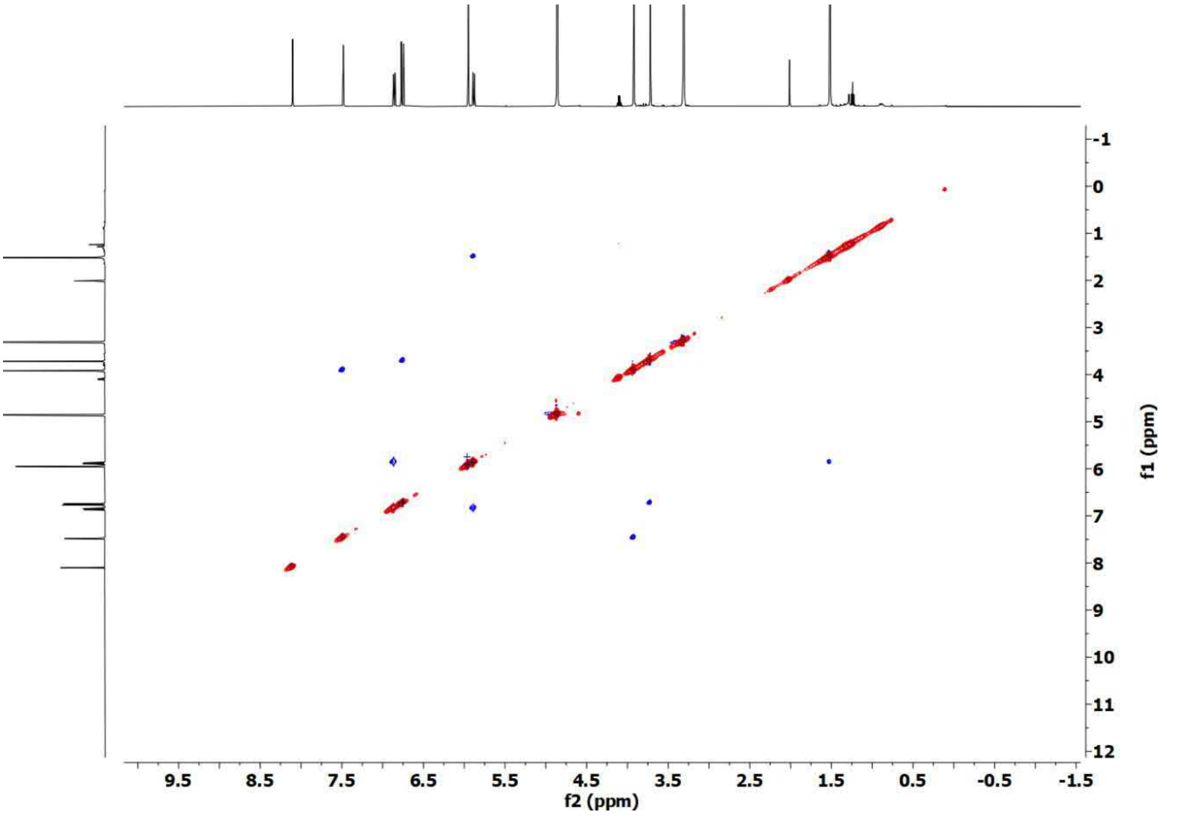

Figure S81. NOESY (500 MHz, MeOD-d4, 25 ºC) spectrum of ichthynone (**11**).

Spectroscopic Data of 7,2',3'-Trimethoxyl-3',4'-Methoxylenedioxyisoflavone (12)

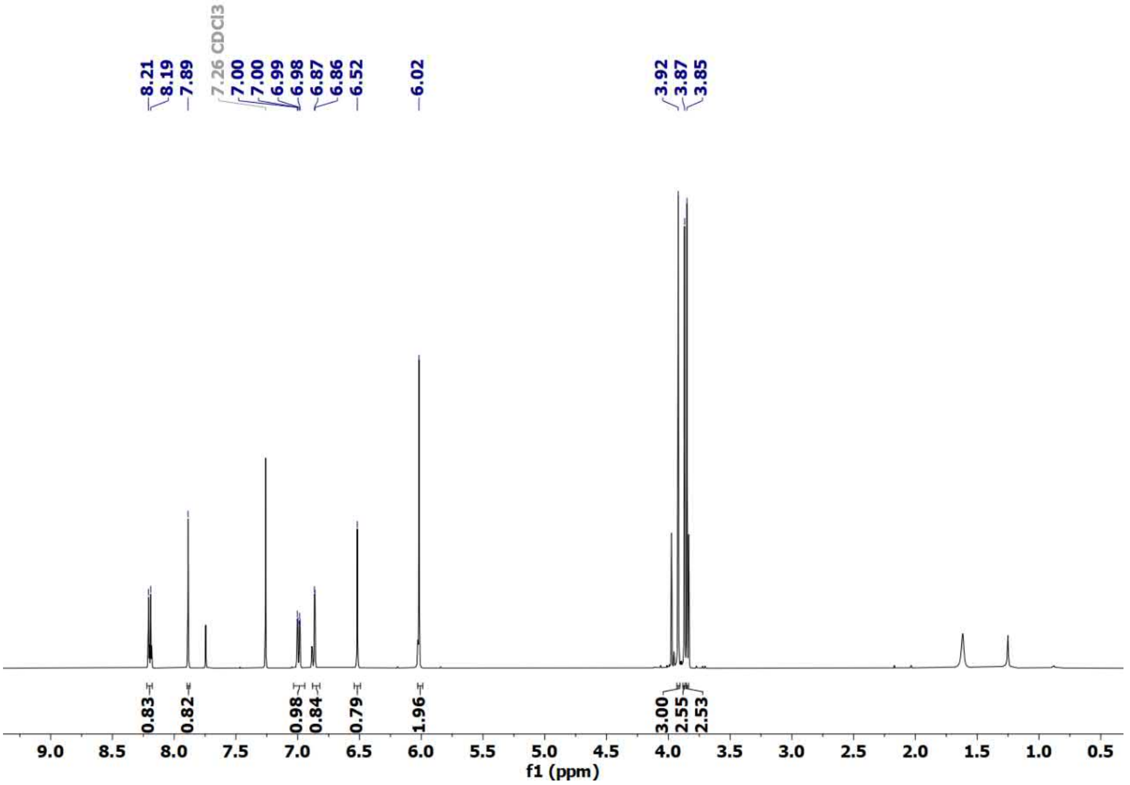

Figure S82. ^{1}H NMR (500 MHz, $CDCl_3$, 25 ºC) spectrum of 7,2',3'-trimethoxy-3',4'-methylenedioxyisoflavone (**12**)

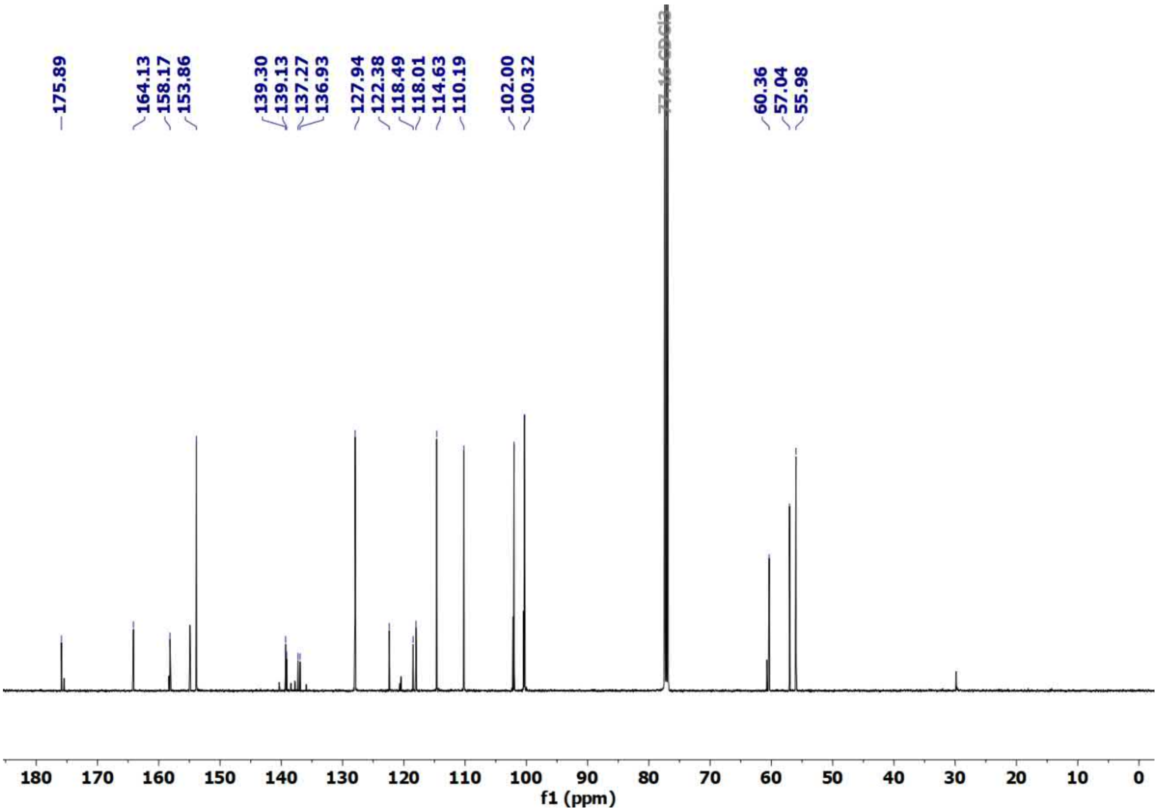

Figure S83. ^{13}C NMR (125 MHz, $CDCl_3$, 25 ºC) spectrum of 7,2',3'-trimethoxy-3',4'-methylenedioxyisoflavone (**12**)

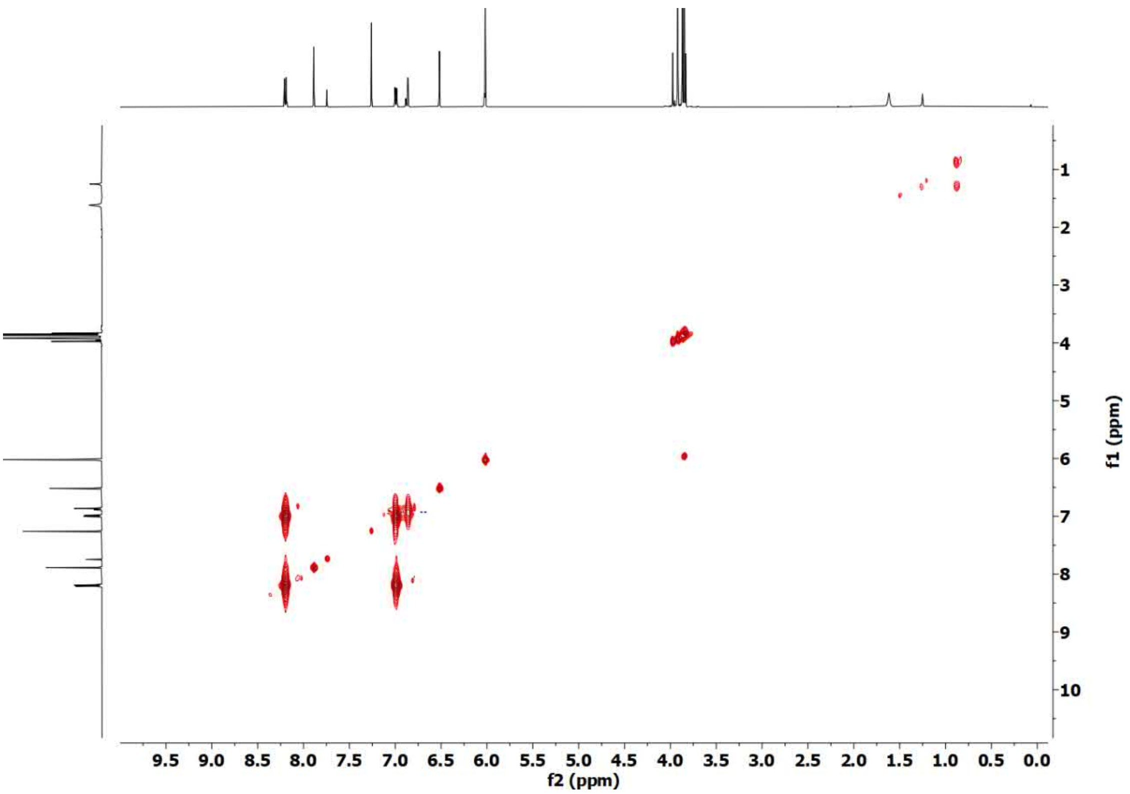

Figure S84. COSY (500 MHz, $CDCl_3$, 25 ºC) spectrum of 7,2',3'-trimethoxy-3',4'-methylenedioxyisoflavone (**12**)

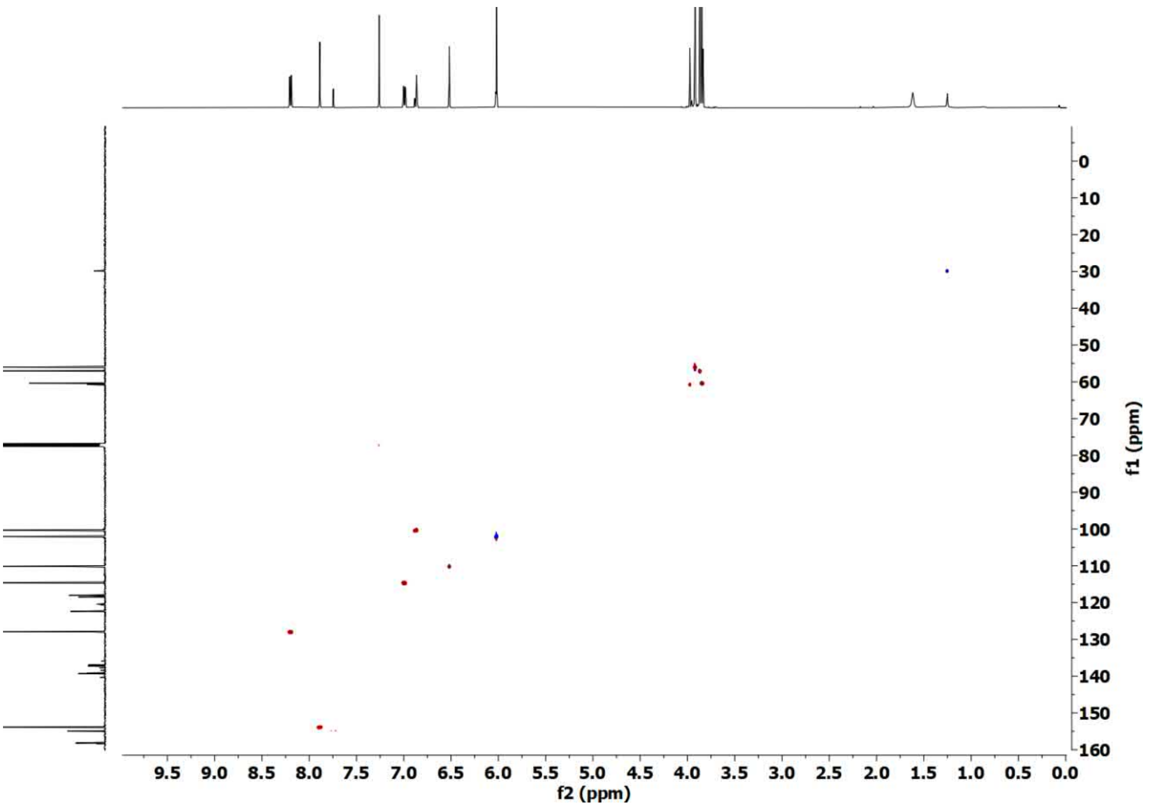

Figure S85. HSQC (500/125 MHz, $CDCl_3$, 25 ºC) spectrum of 7,2',3'-trimethoxy-3',4'-methylenedioxyisoflavone (**12**)

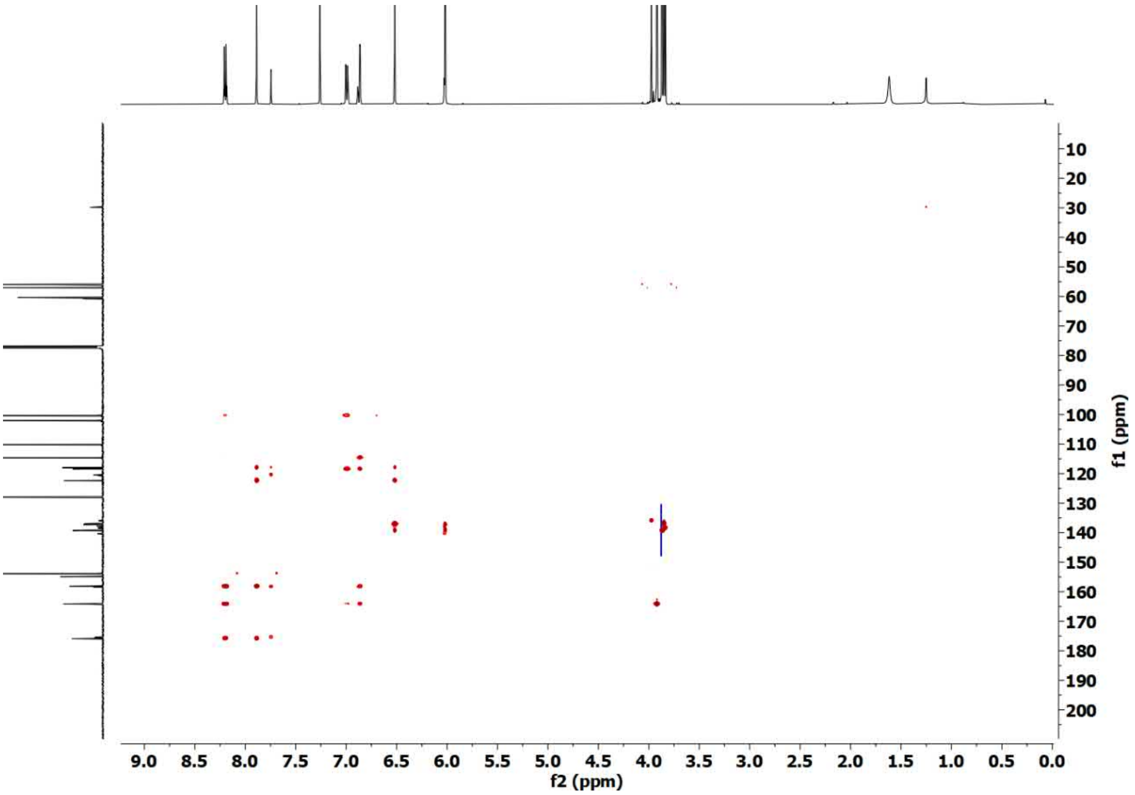

Figure S86. HMBC (500/125 MHz, $CDCl_3$, 25 ºC) spectrum of 7,2',3'-trimethoxy-3',4'-methylenedioxyisoflavone (**12**).

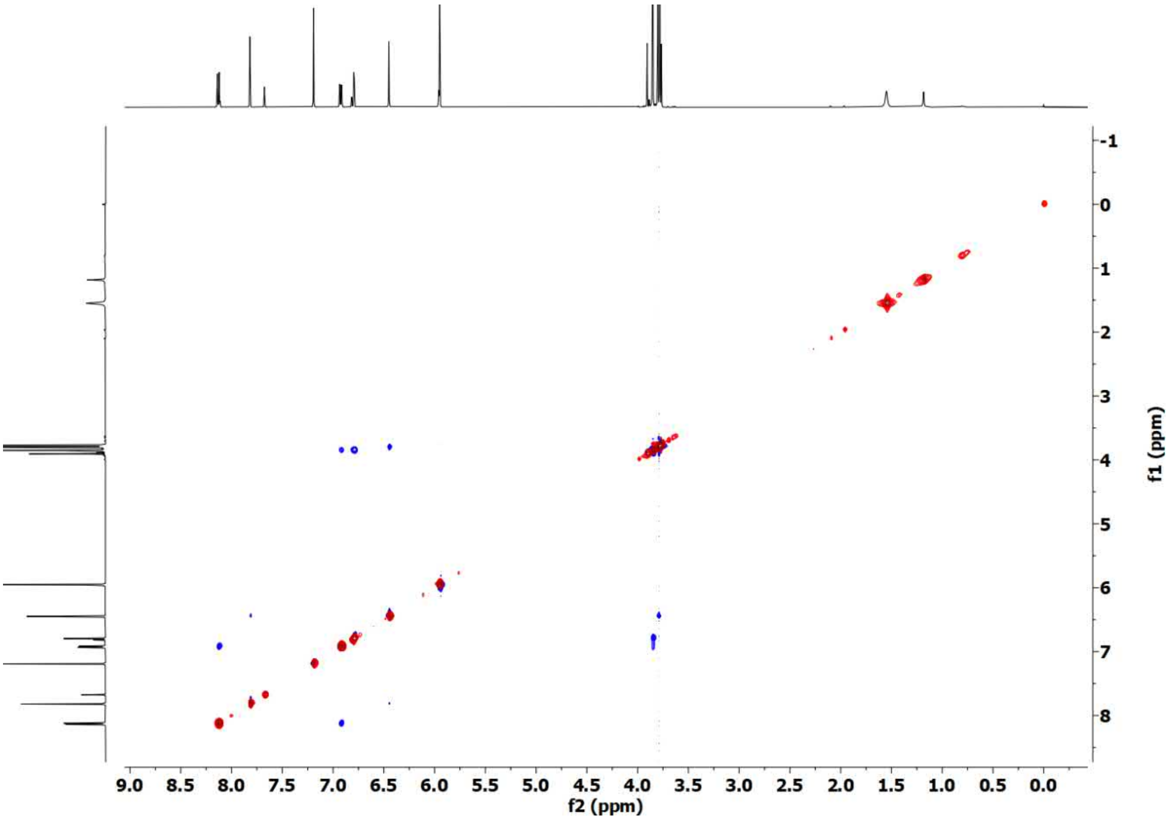

Figure S87. NOESY (500 MHz, $CDCl_3$, 25 ºC) spectrum of 7,2',3'-trimethoxy-3',4'-methylenedioxyisoflavone (**12**)

Spectroscopic Data of Isoerythrin-A-4'-prenylether (13)

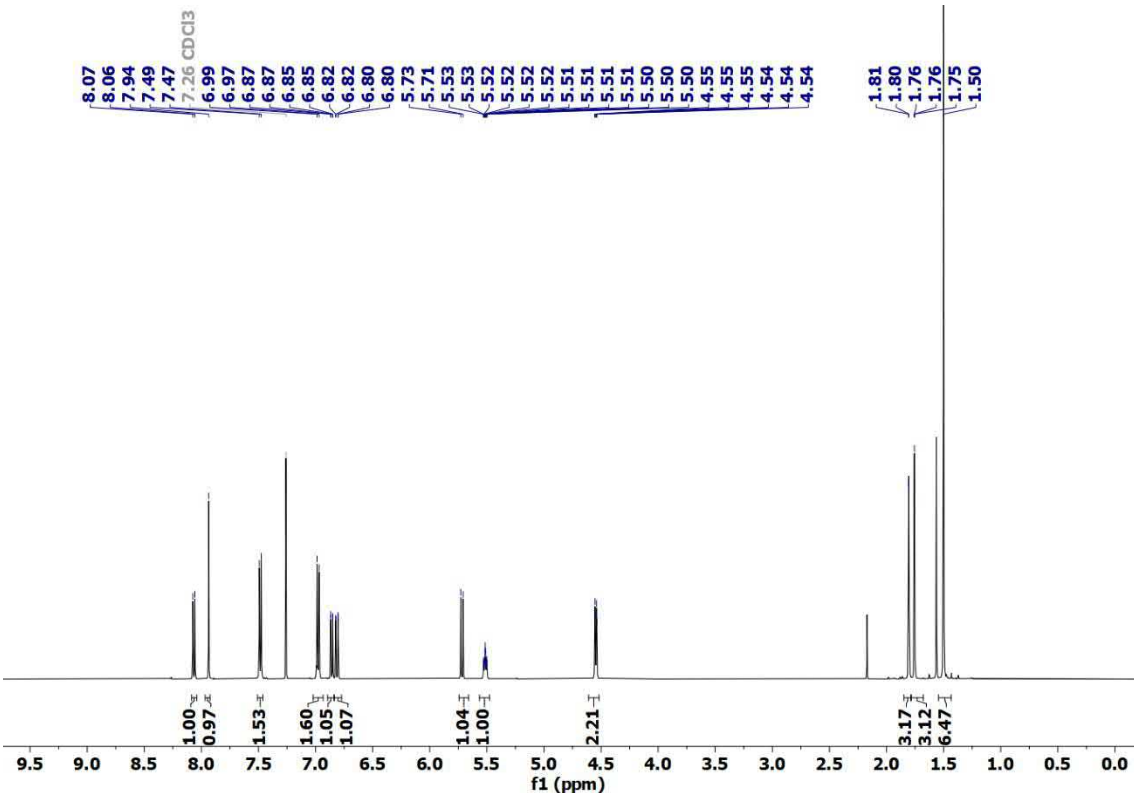

Figure S88. ^{1}H NMR (500 MHz, $CDCl_3$, 25 ºC) spectrum of isoerythrin-A-4'-prenylether (**13**)

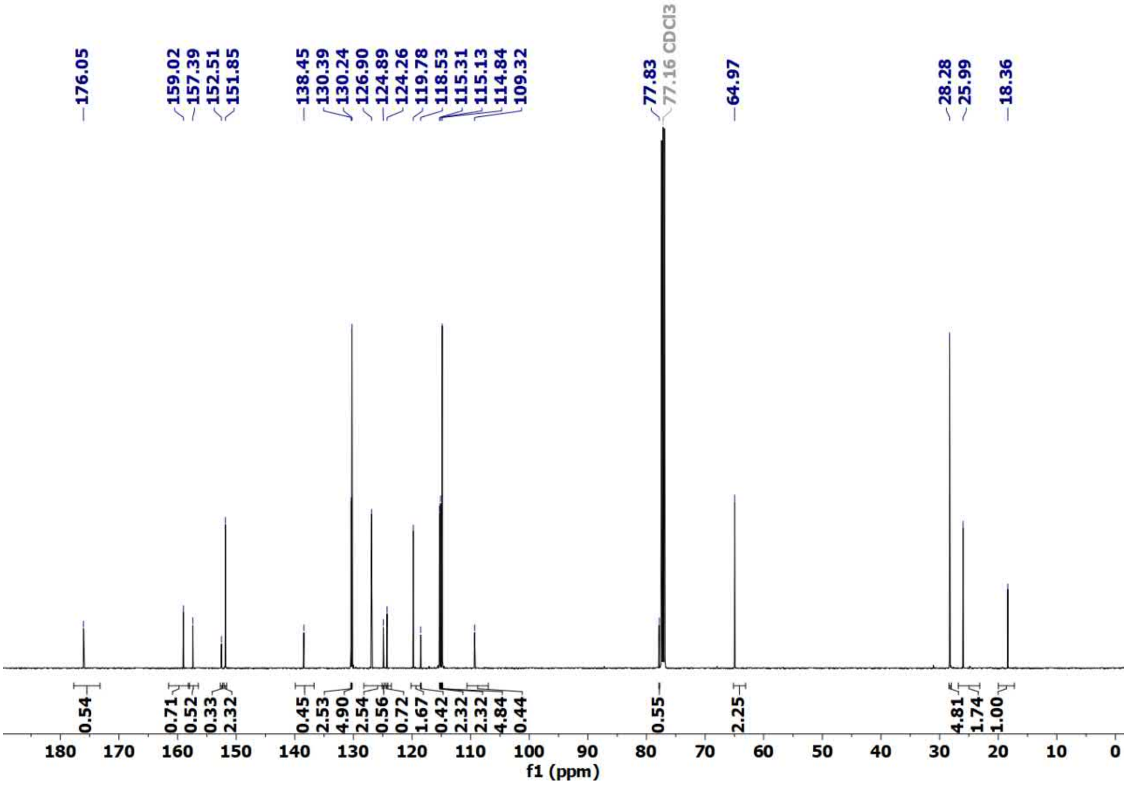

Figure S89. ^{13}C NMR (125 MHz, $CDCl_3$, 25 ºC) spectrum of isoerythrin-A-4'-prenylether (**13**)

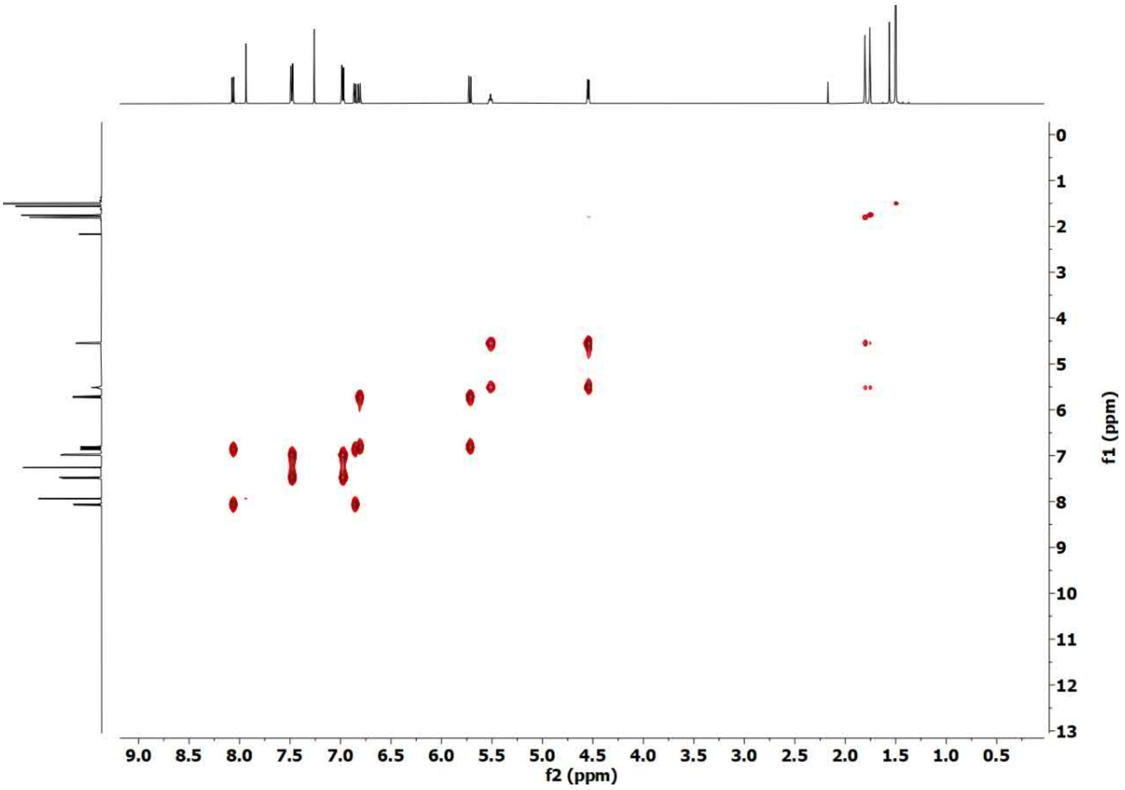

Figure S90. COSY (500 MHz, $CDCl_3$, 25 ºC) spectrum of isoerythrin-A-4'-prenylether (**13**)

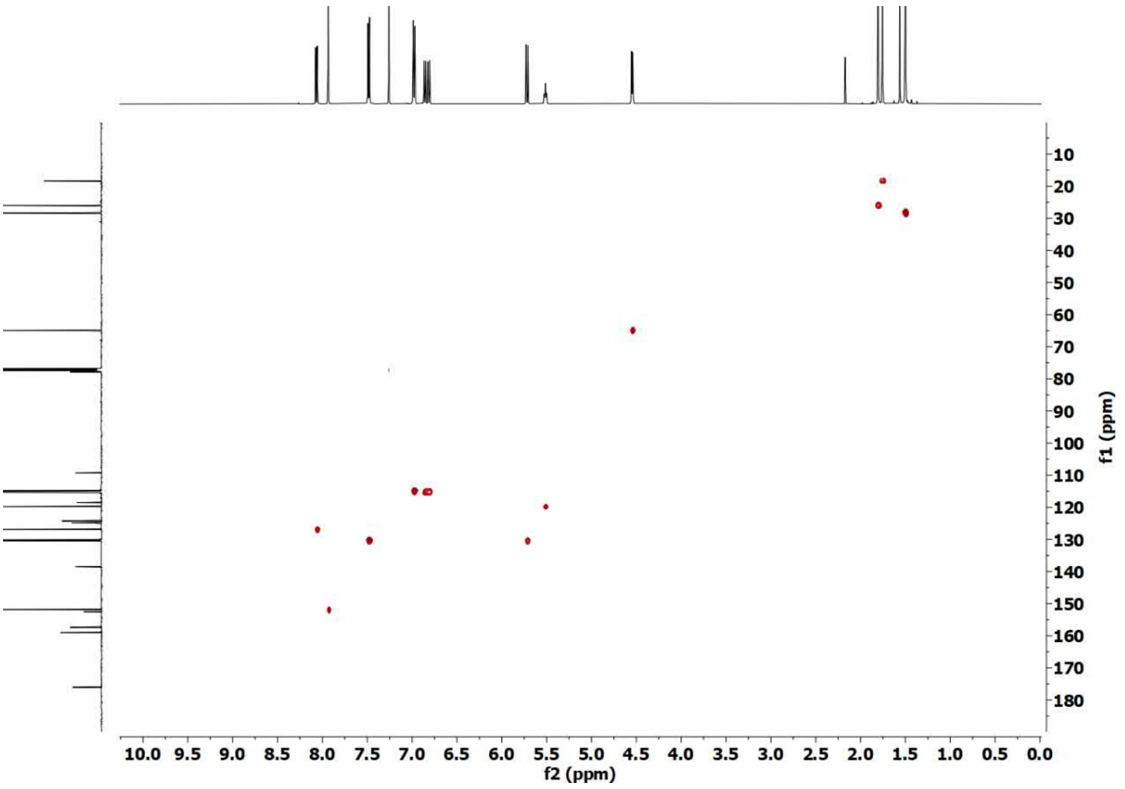

Figure S91. HSQC (500/125 MHz, $CDCl_3$, 25 ºC) spectrum of isoerythrin-A-4'-prenylether (**13**)

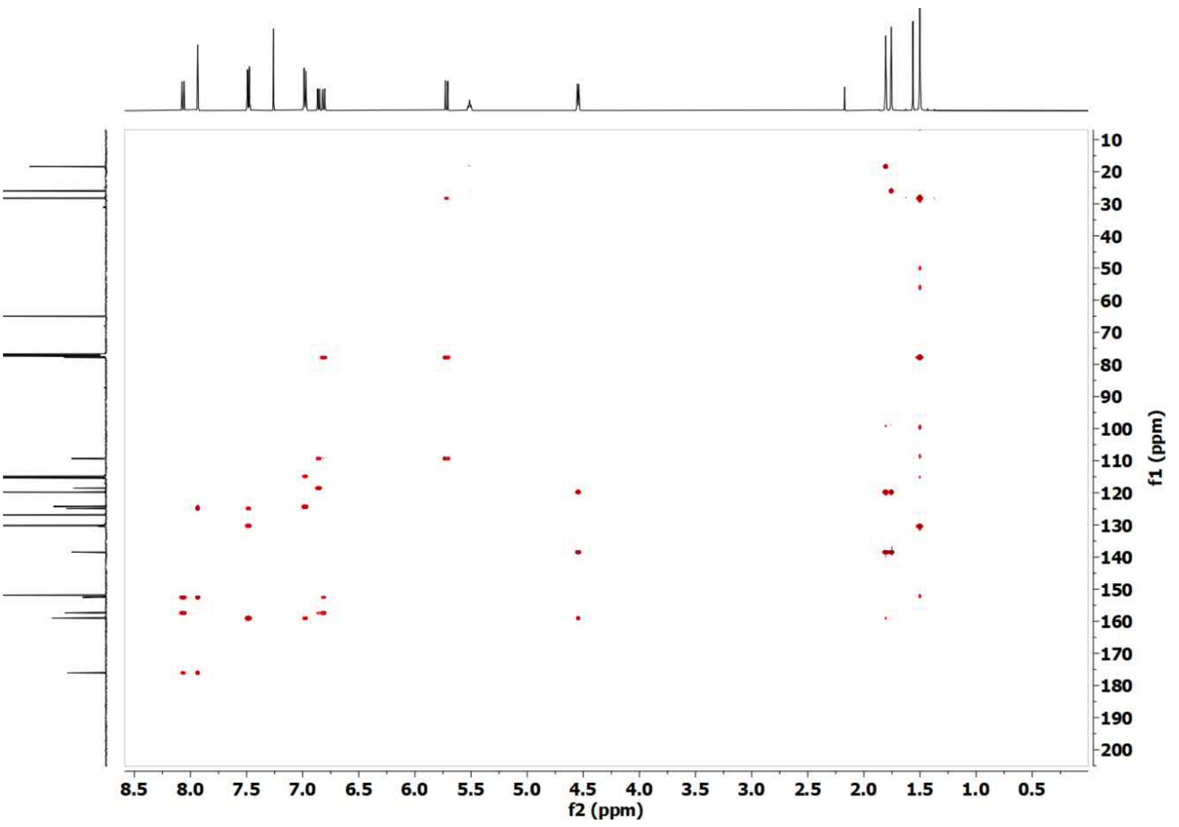

Figure S92. HMBC (500/125 MHz, $CDCl_3$, 25 ºC) spectrum of isoerythrin-A-4'-prenylether (**13**)

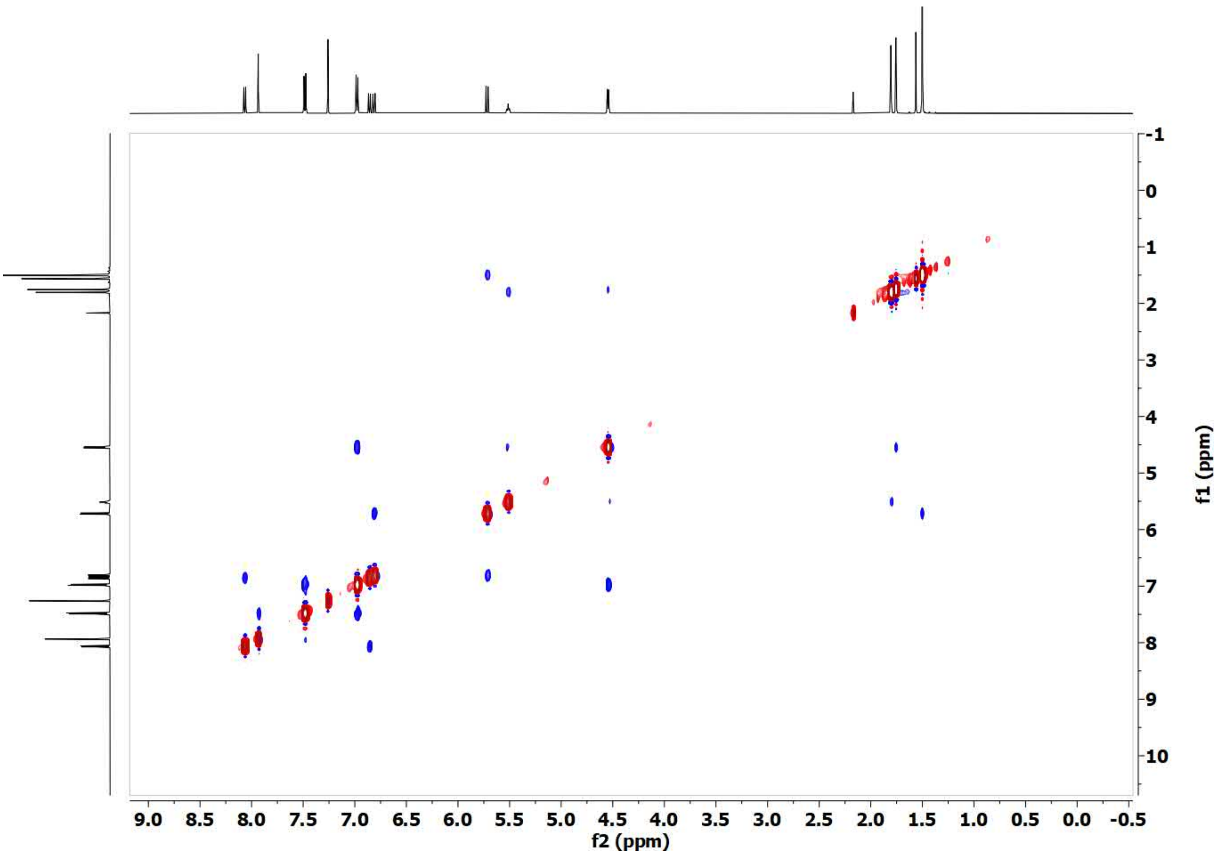

Figure S93. NOESY (500 MHz, $CDCl_3$, 25 ºC) spectrum of isoerythrin-A-4'-prenylether (**13**).

Spectroscopic Data of 4'-Prenyloxyderone (14)

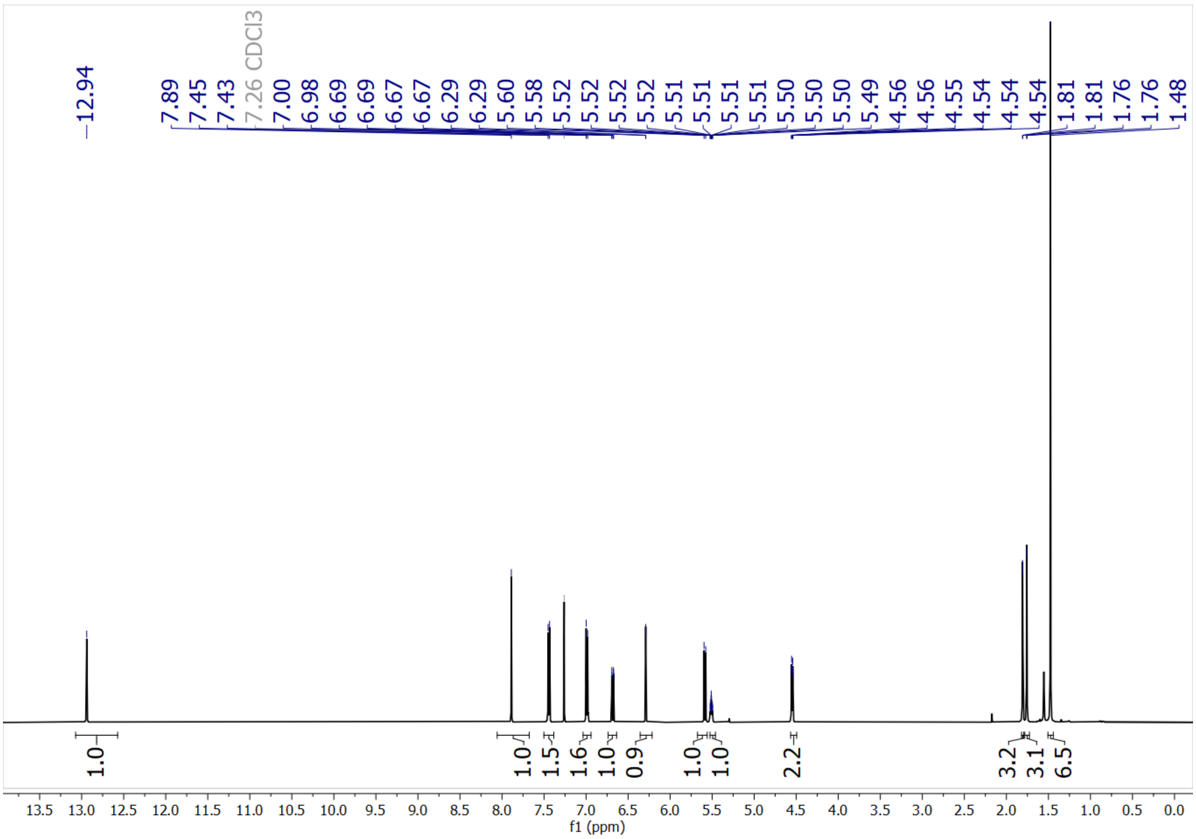

Figure S94. ^{1}H NMR (500 MHz, $CDCl_3$, 25 ºC) spectrum of 4'-prenyloxyderone (**14**)

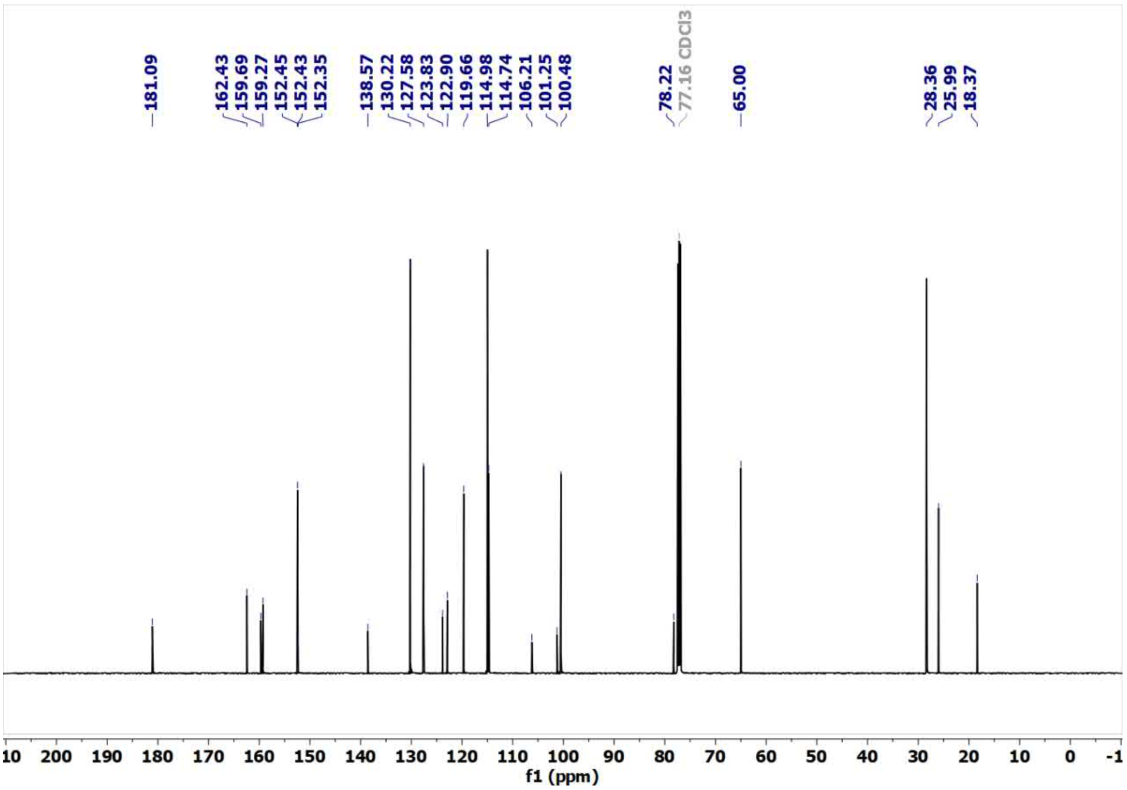

Figure S95. ^{13}C NMR (125 MHz, $CDCl_3$, 25 ºC) spectrum of 4'-prenyloxyderone (**14**)

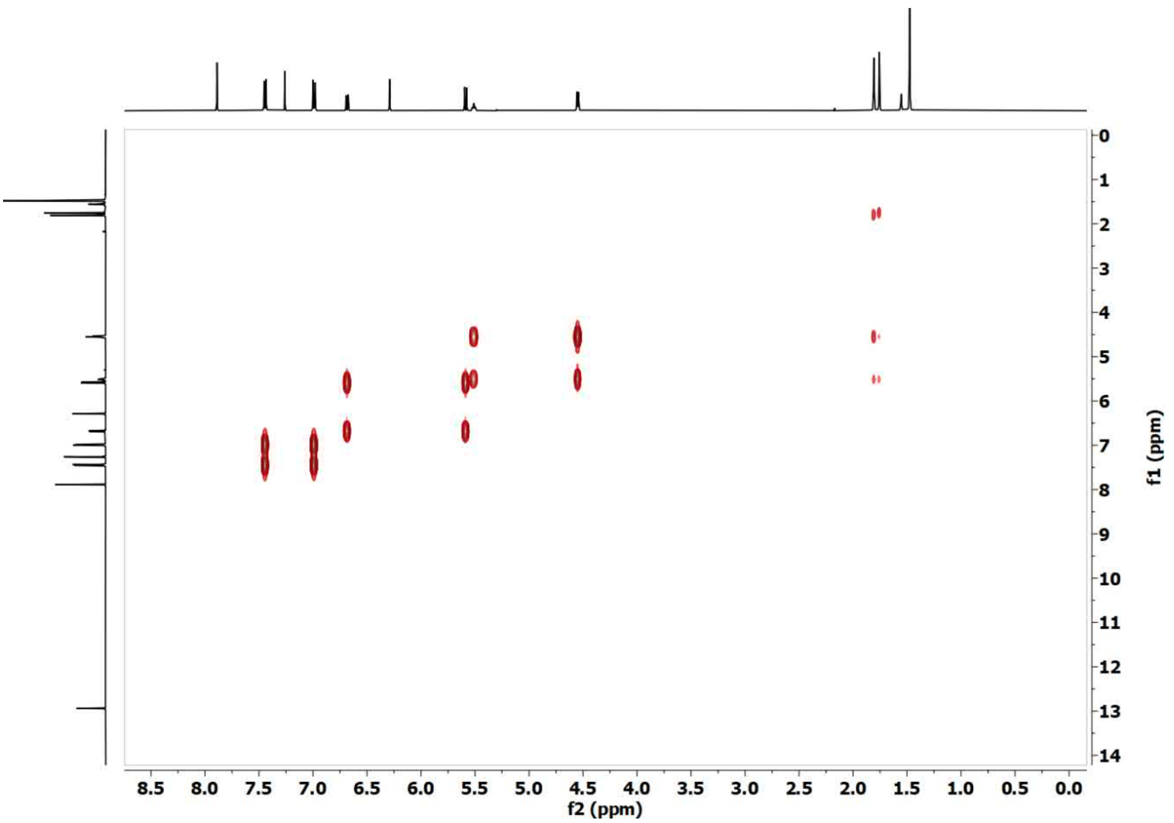

Figure S96. COSY (500 MHz, $CDCl_3$, 25 ºC) spectrum of 4'-prenyloxyderone (**14**)

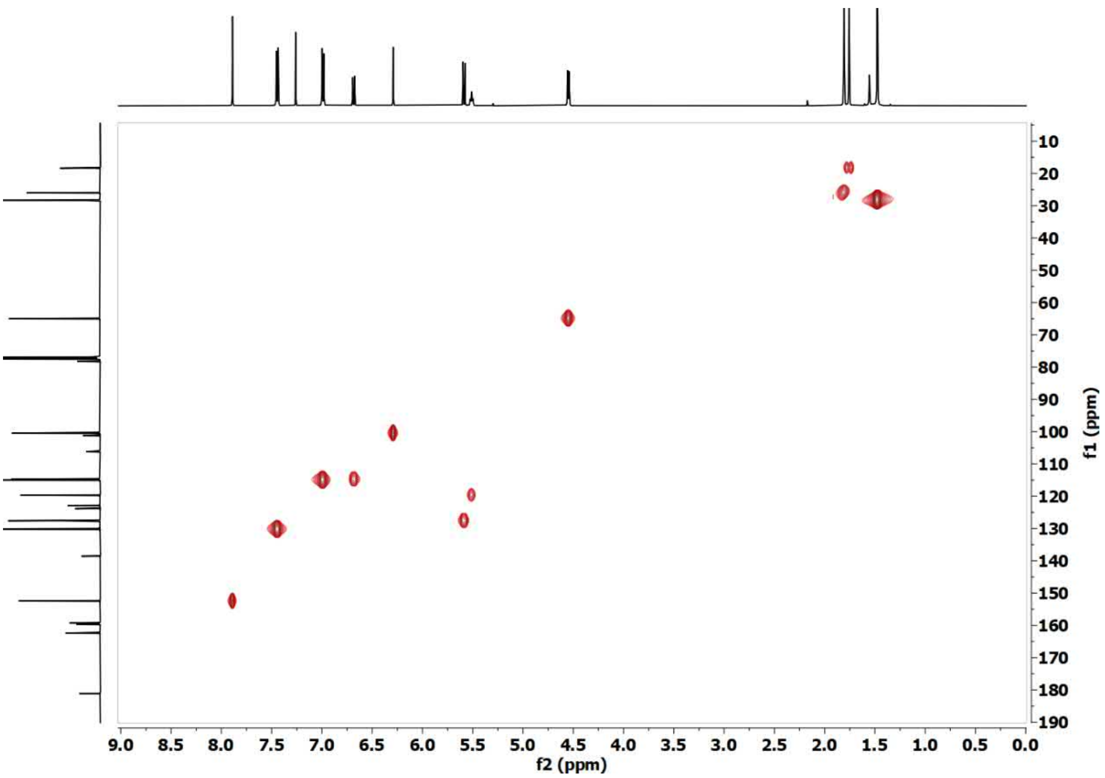

Figure S97. HSQC (500/125 MHz, $CDCl_3$, 25 ºC) spectrum of 4'-prenyloxyderone (**14**)

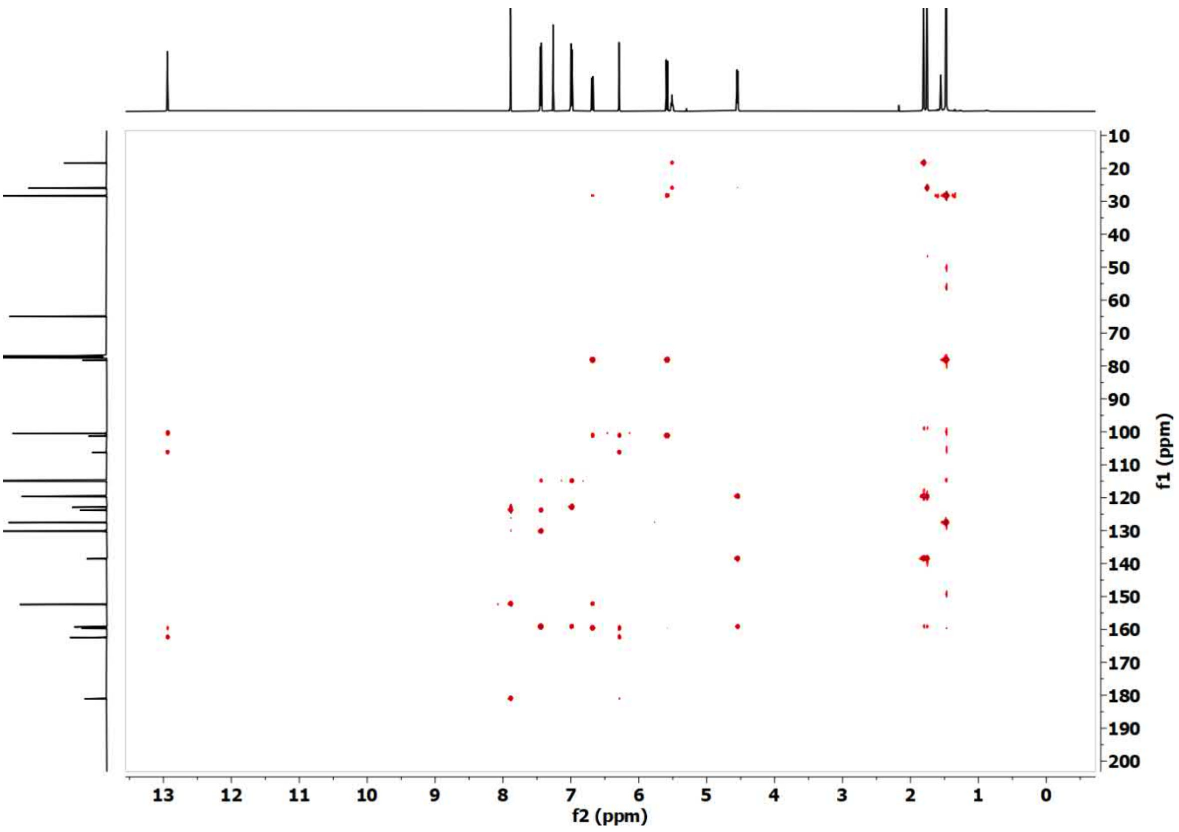

Figure S98. HMBC (500/125 MHz, $CDCl_3$, 25 ºC) spectrum of 4'-prenyloxyderone (**14**)

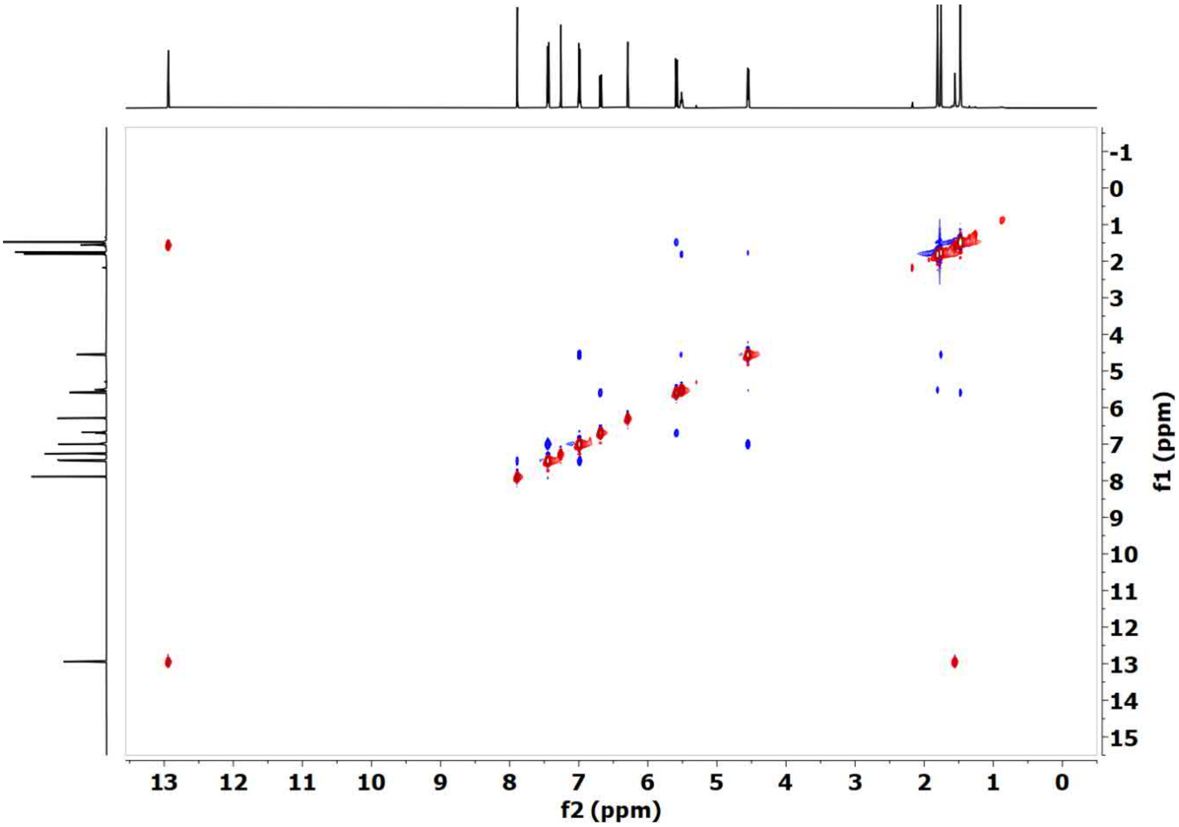

Figure S99. NOESY (500 MHz, $CDCl_3$, 25 ºC) spectrum of 4'-prenyloxyderone (**14**)

Spectroscopic Data of Cuneatin Methyl Ether (15)

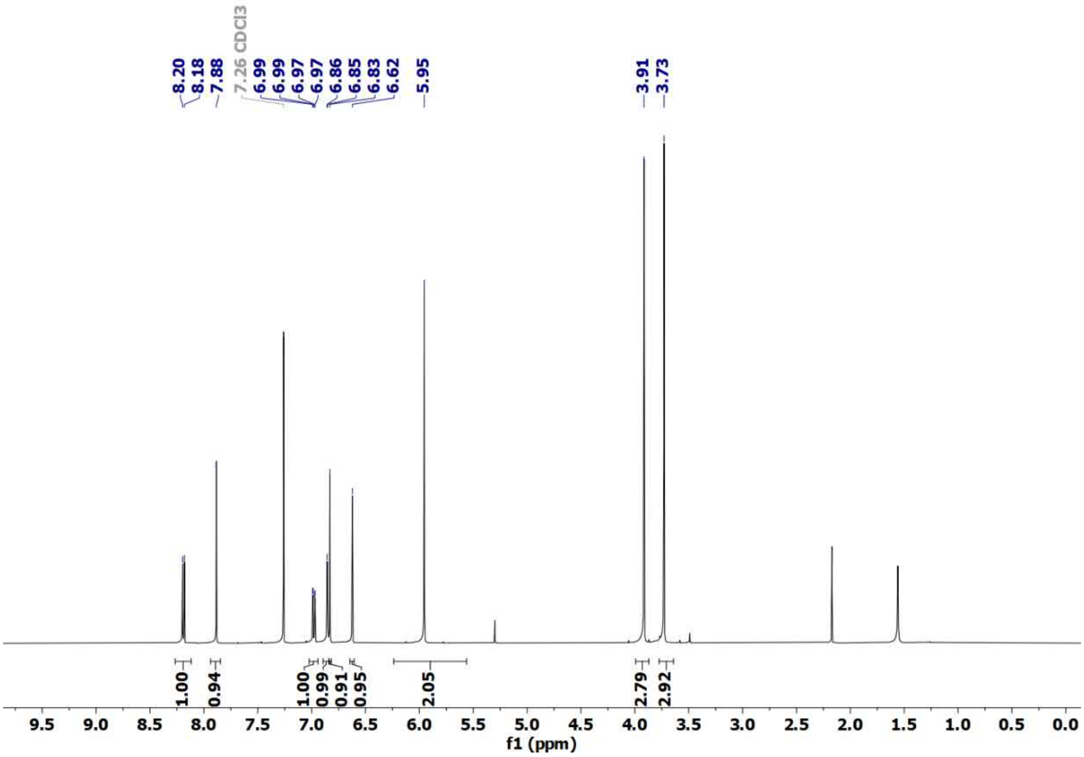

Figure S100. ^{1}H NMR (500 MHz, $CDCl_3$, 25 ºC) spectrum of cuneatin methyl ether (**15**)

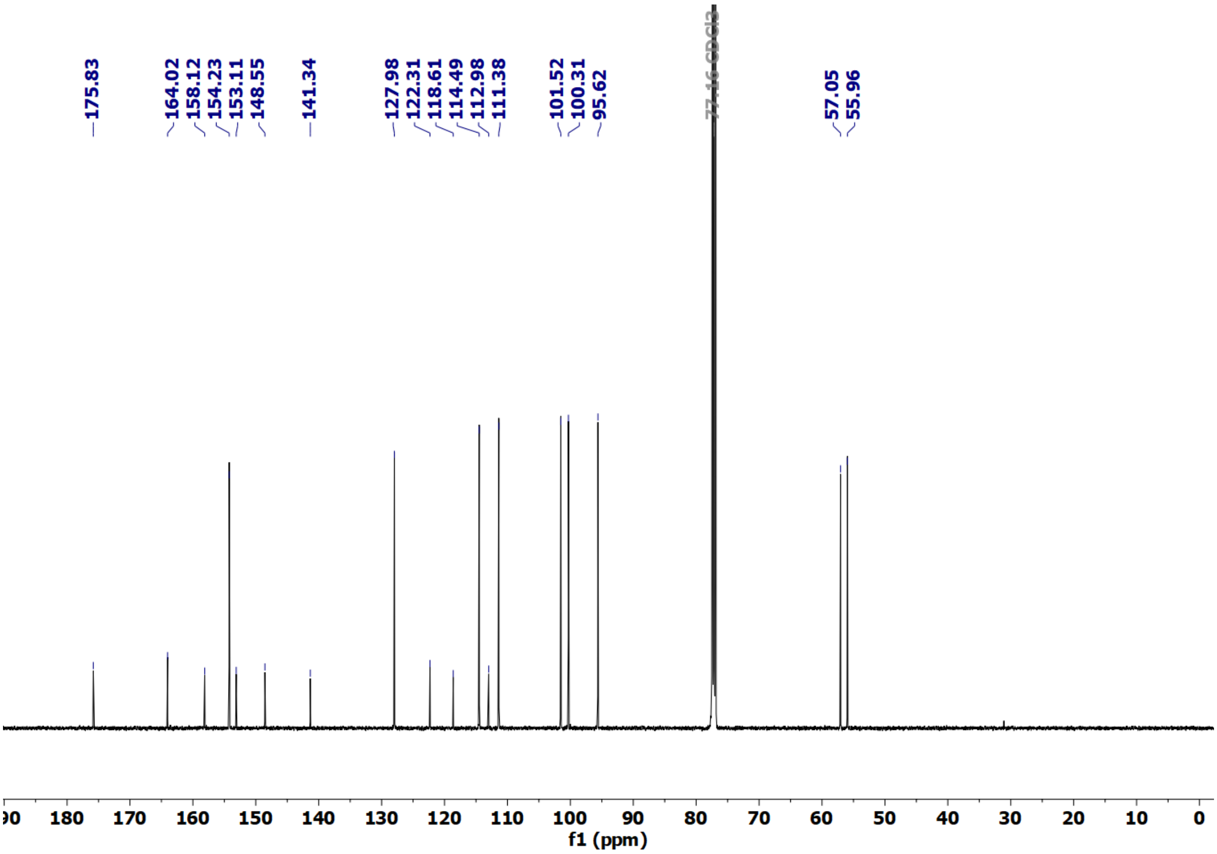

Figure S101. ^{13}C NMR (125 MHz, $CDCl_3$, 25 ºC) spectrum of cuneatin methyl ether (**15**)

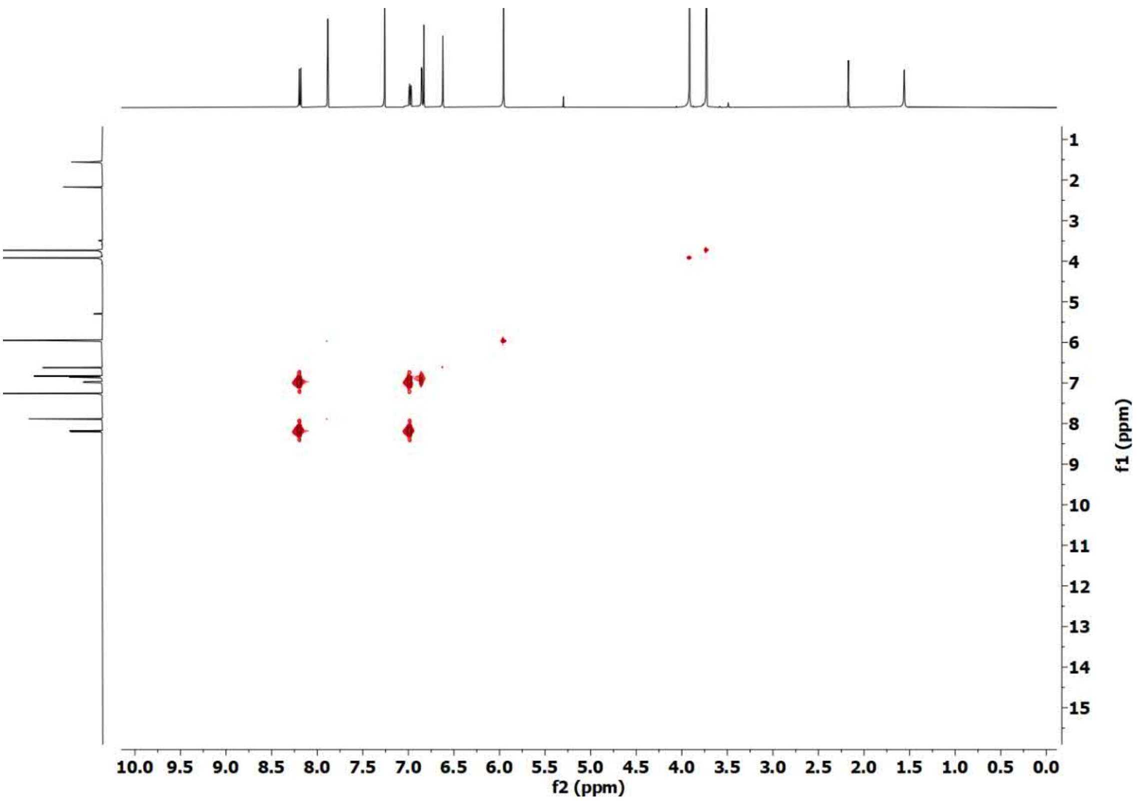

Figure S102. COSY (500 MHz, $CDCl_3$, 25 ºC) spectrum of cuneatin methyl ether (**15**)

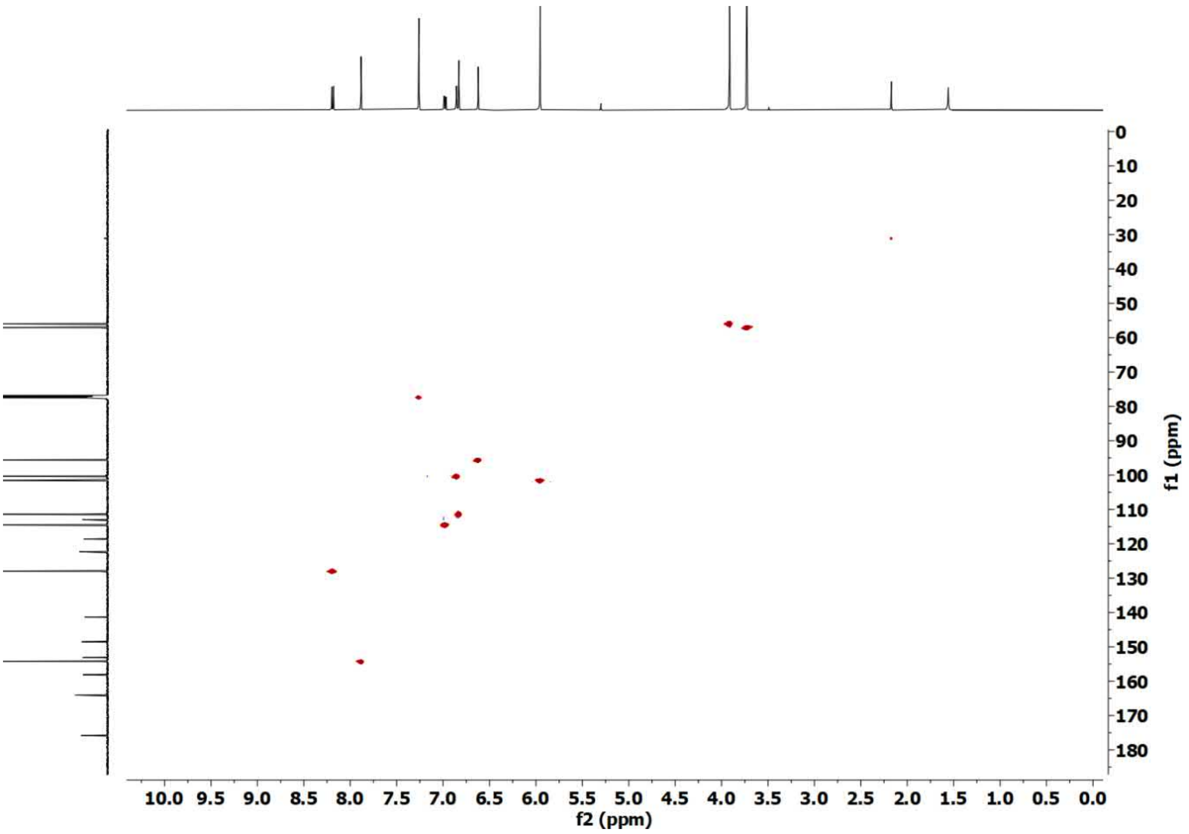

Figure S103. HSQC (500/125 MHz, $CDCl_3$, 25 ºC) spectrum of cuneatin methyl ether (**15**)

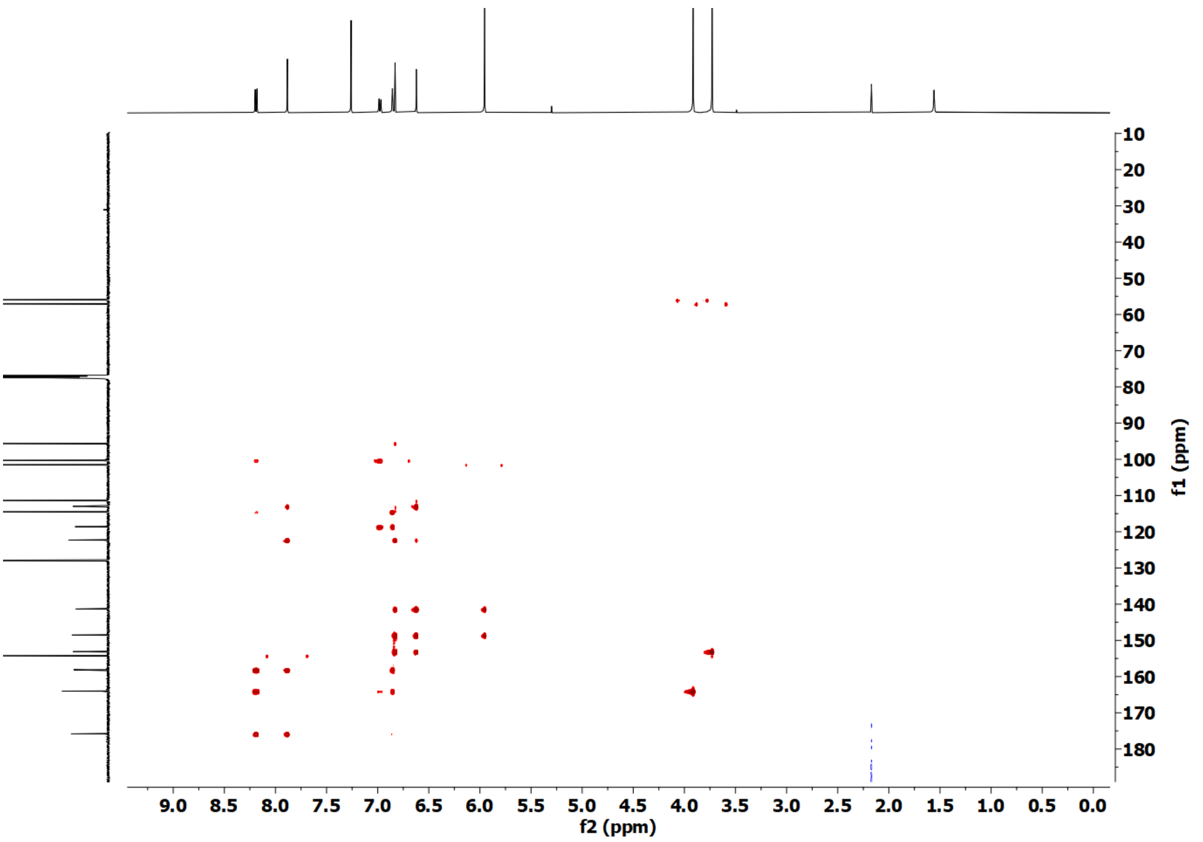

Figure S104. HMBC (500/125 MHz, $CDCl_3$, 25 ºC) spectrum of cuneatin methyl ether (**15**)

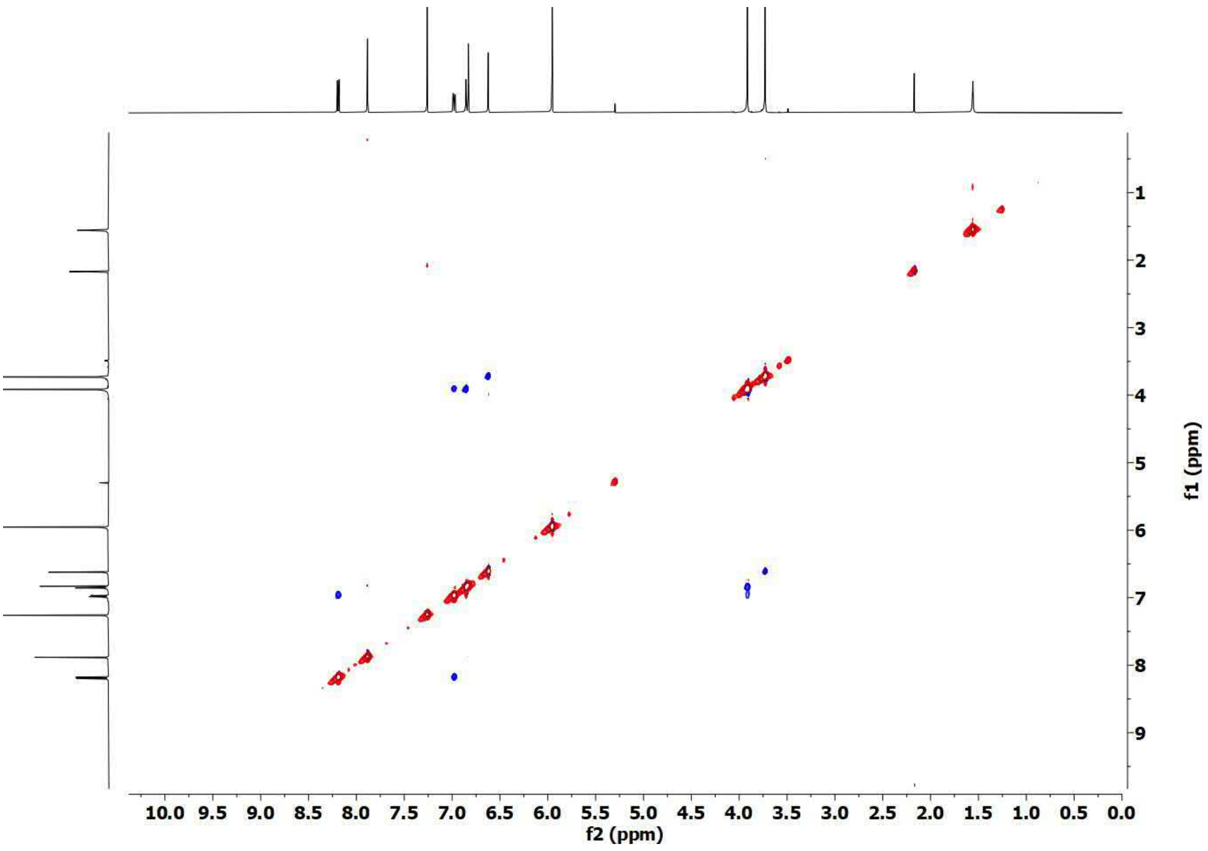

Figure S105. NOESY (500 MHz, $CDCl_3$, 25 ºC) spectrum of cuneatin methyl ether (**15**)

Spectroscopic Data of Calopogonium Isoflavone B (16)

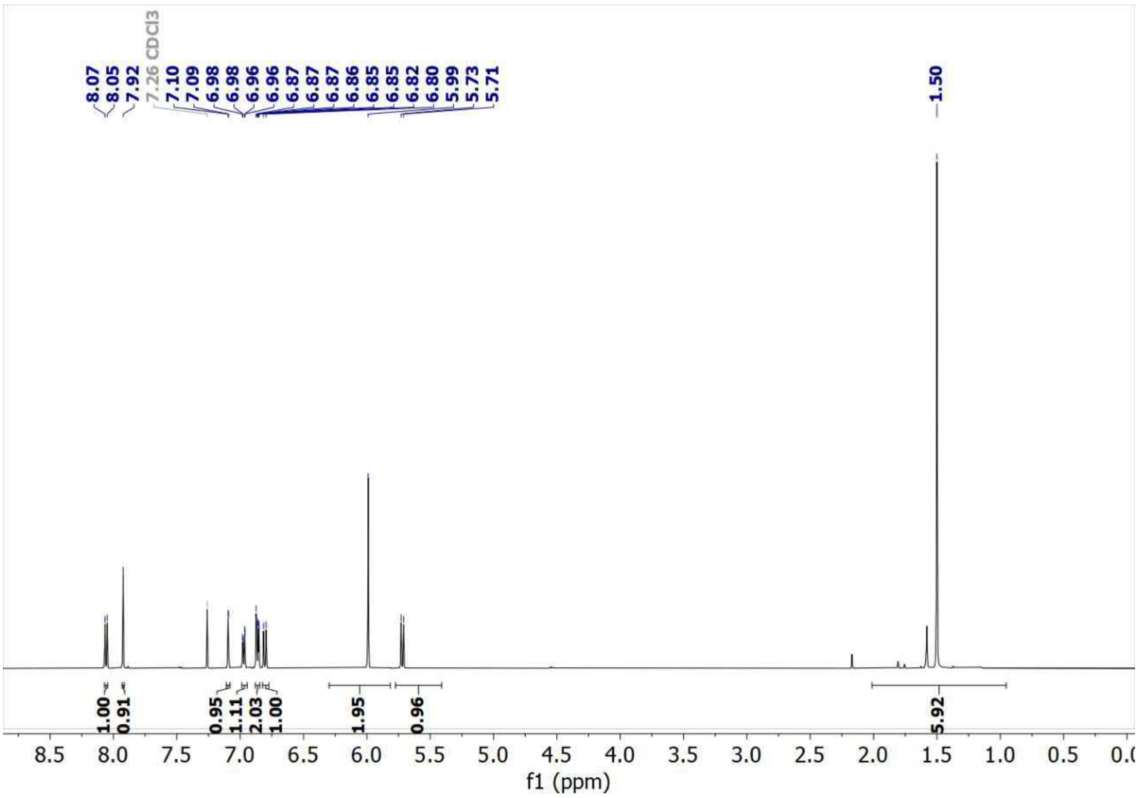

Figure S106. ^{1}H NMR (500 MHz, $CDCl_3$, 25 ºC) spectrum of calopogonium isoflavone B (**16**)

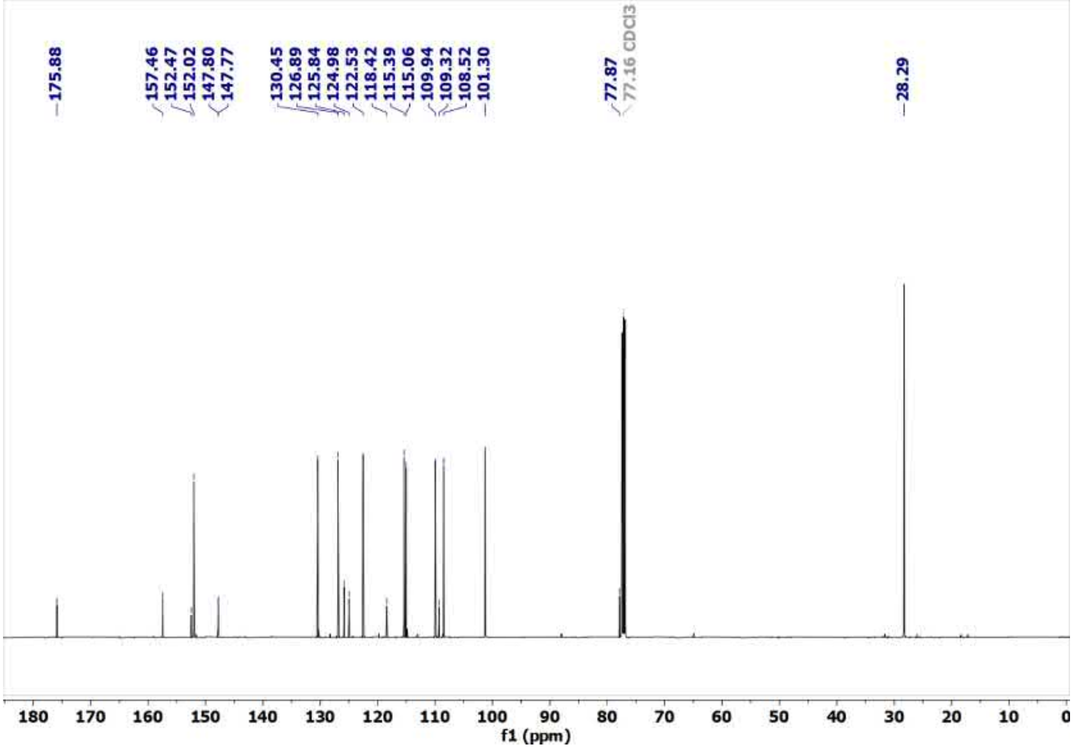

Figure S107. ^{13}C NMR (125 MHz, $CDCl_3$, 25 ºC) spectrum of calopogonium isoflavone B (**16**)

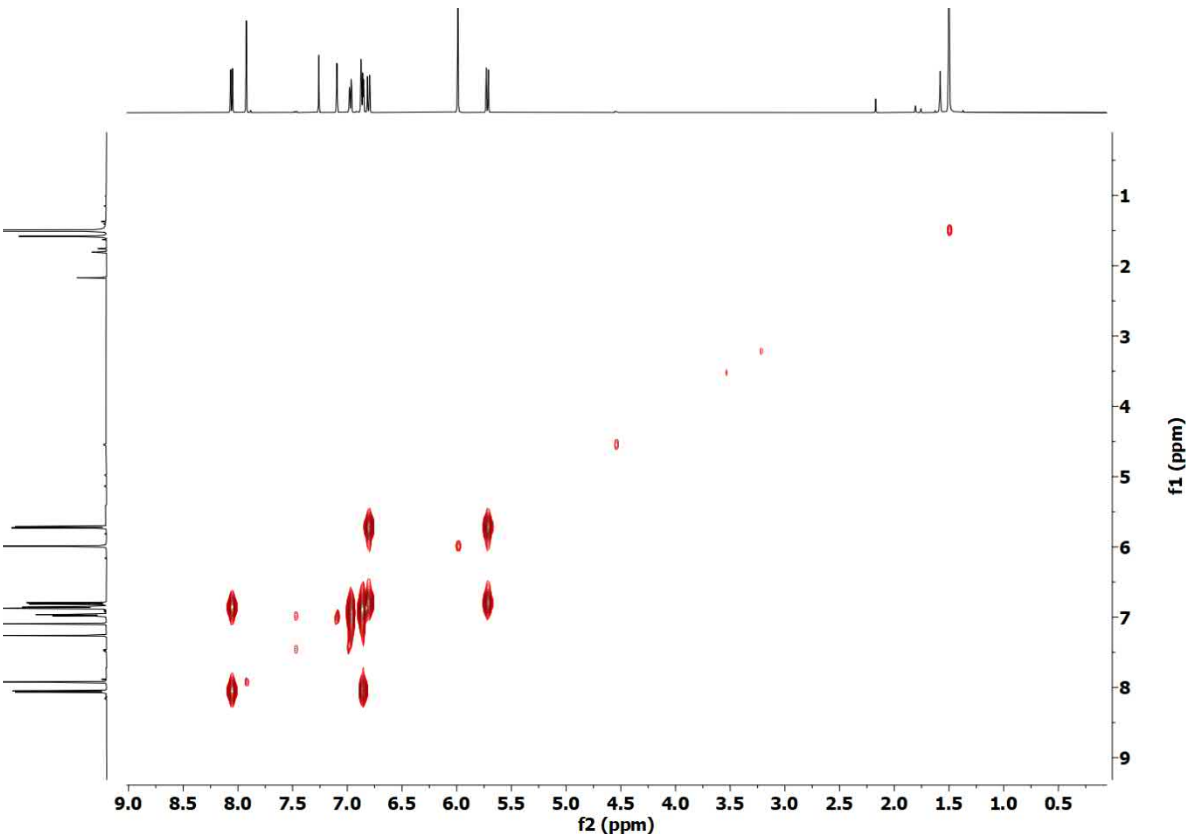

Figure S108. COSY (500 MHz, $CDCl_3$, 25 ºC) spectrum of calopogonium isoflavone B (**16**)

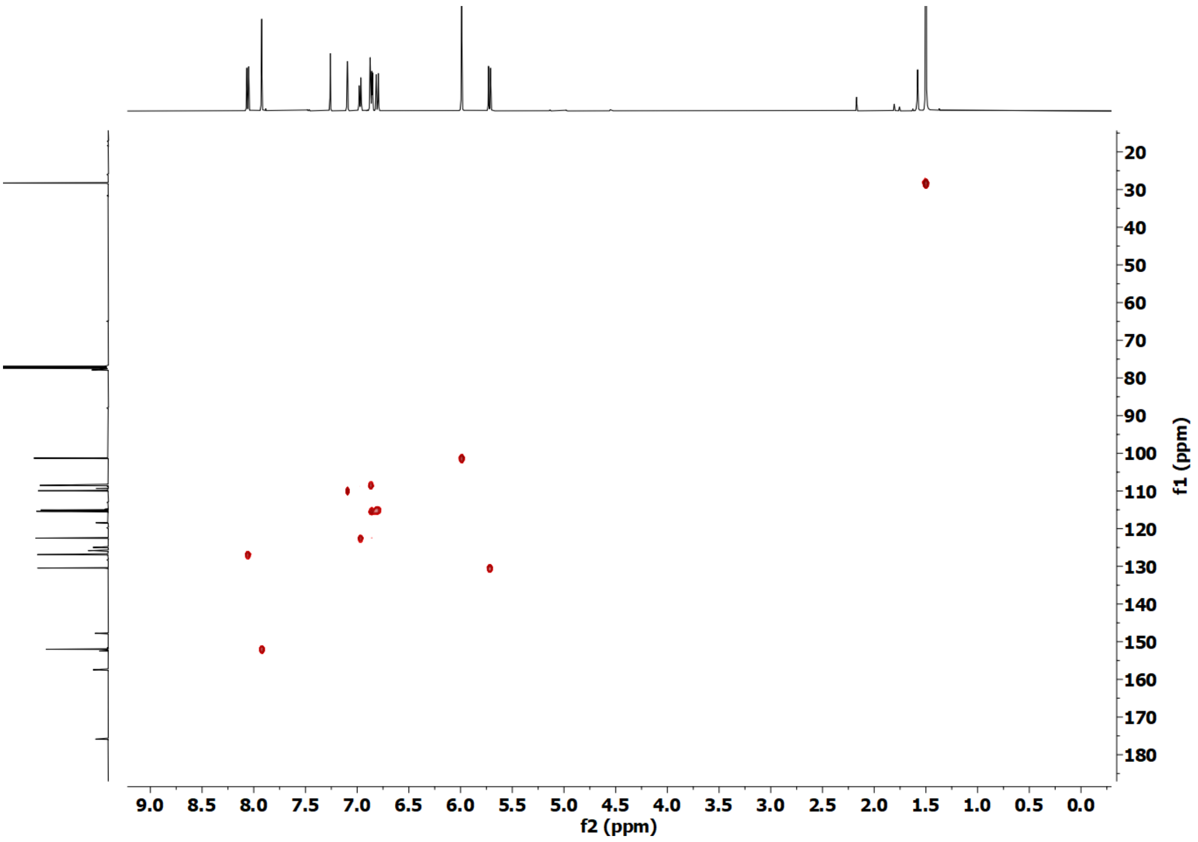

Figure S109. HSQC (500/125 MHz, $CDCl_3$, 25 ºC) spectrum of calopogonium isoflavone B (**16**)

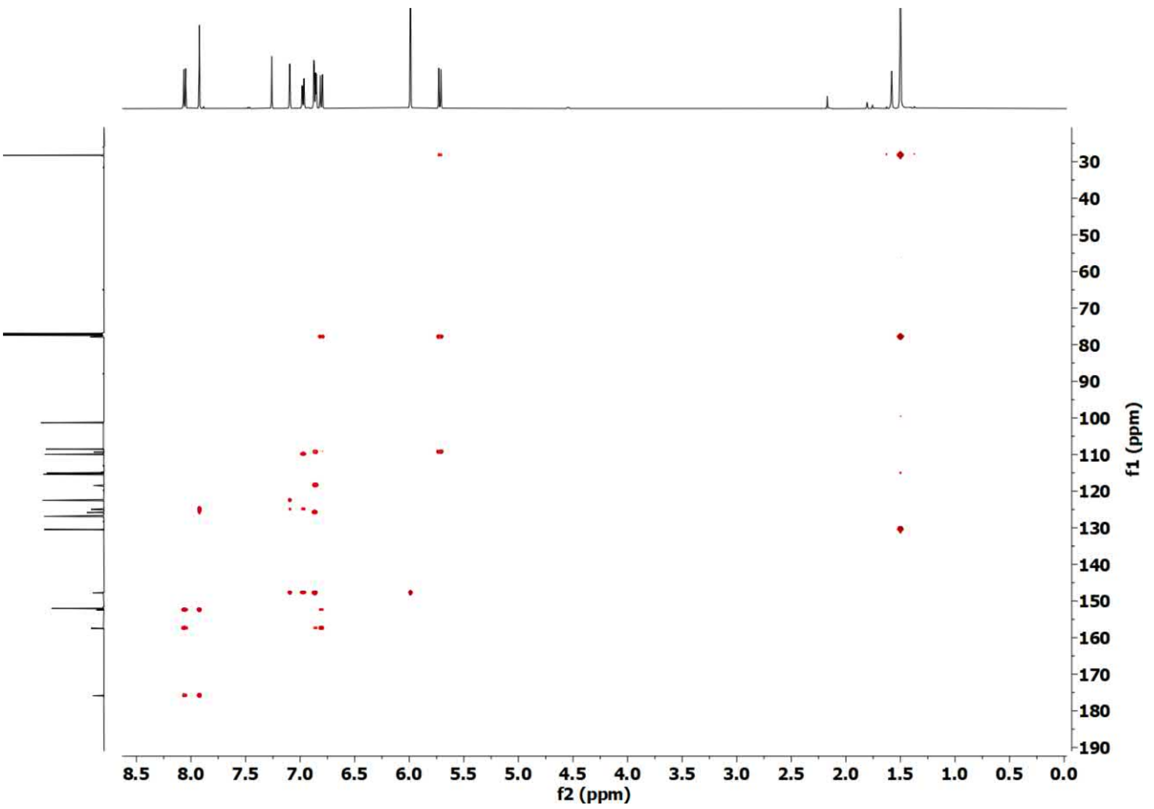

Figure S110. HMBC (500/125 MHz, $CDCl_3$, 25 ºC) spectrum of calopogonium isoflavone B (**16**)

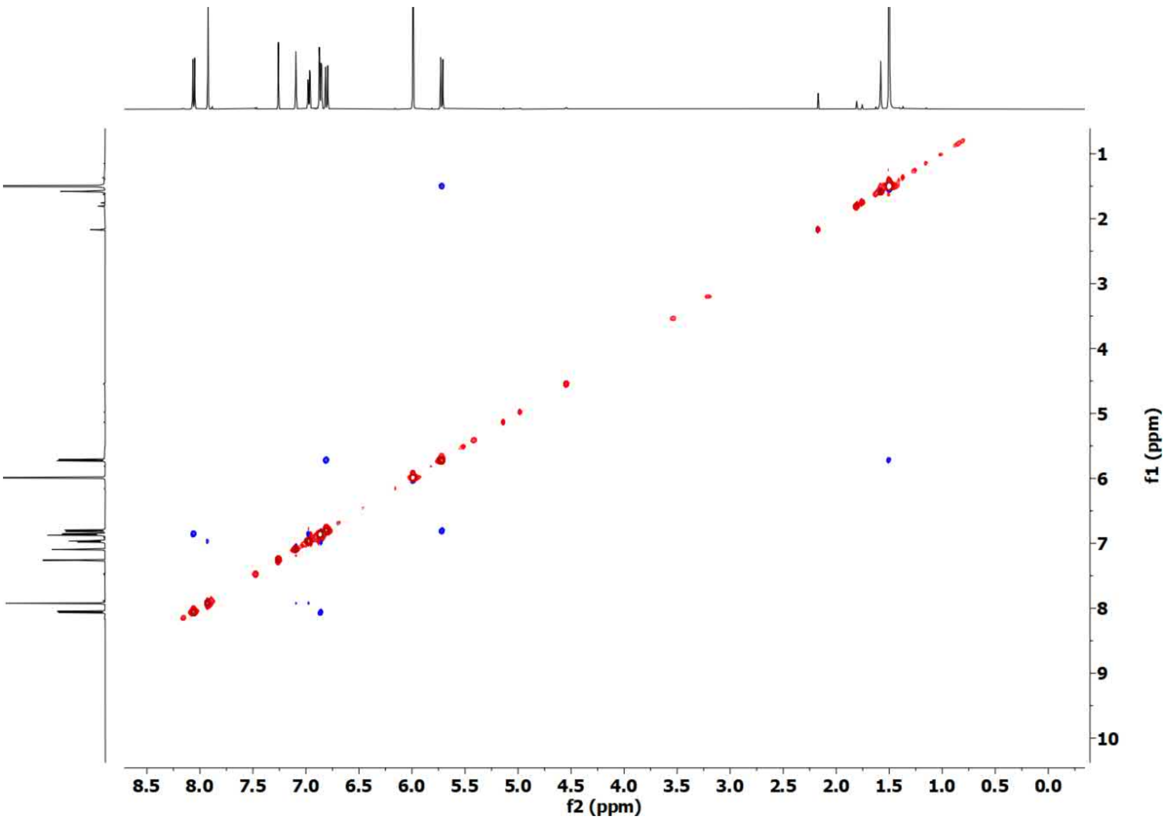

Figure S111. NOESY (500 MHz, $CDCl_3$, 25 ºC) Spectrum of calopogonium isoflavone B (**16**)

Spectroscopic Data of Maximaisoflavone G (17)

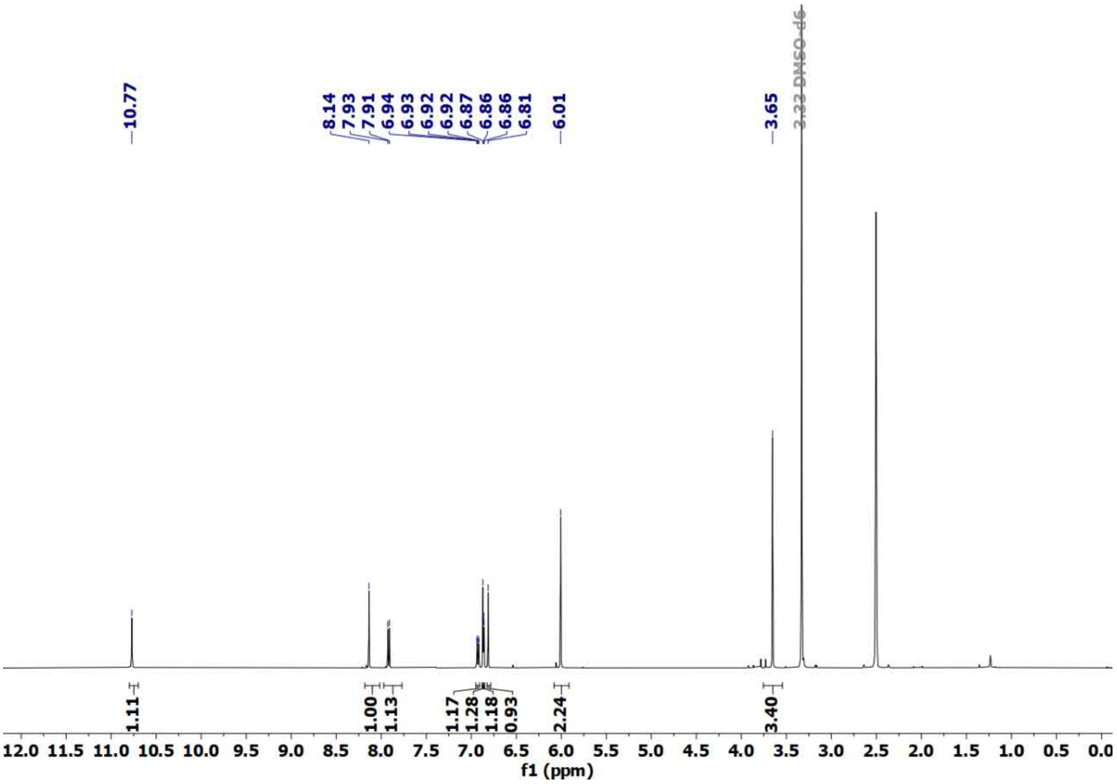

Figure S112. ^{1}H NMR (500 MHz, DMSO-d6, 25 ºC) spectrum of maximaisoflavone (**17**)

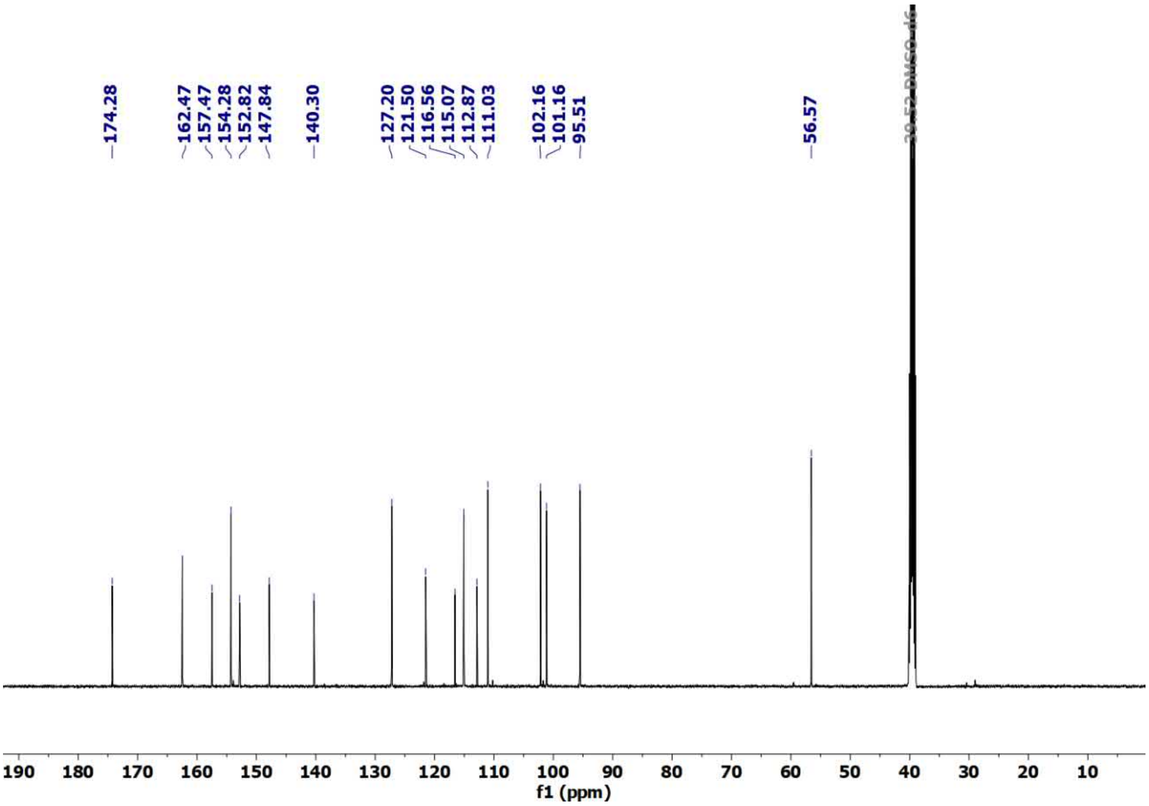

Figure S113. ^{13}C NMR (125 MHz, DMSO-d6, 25 ºC) spectrum of maximaisoflavone (**17**)

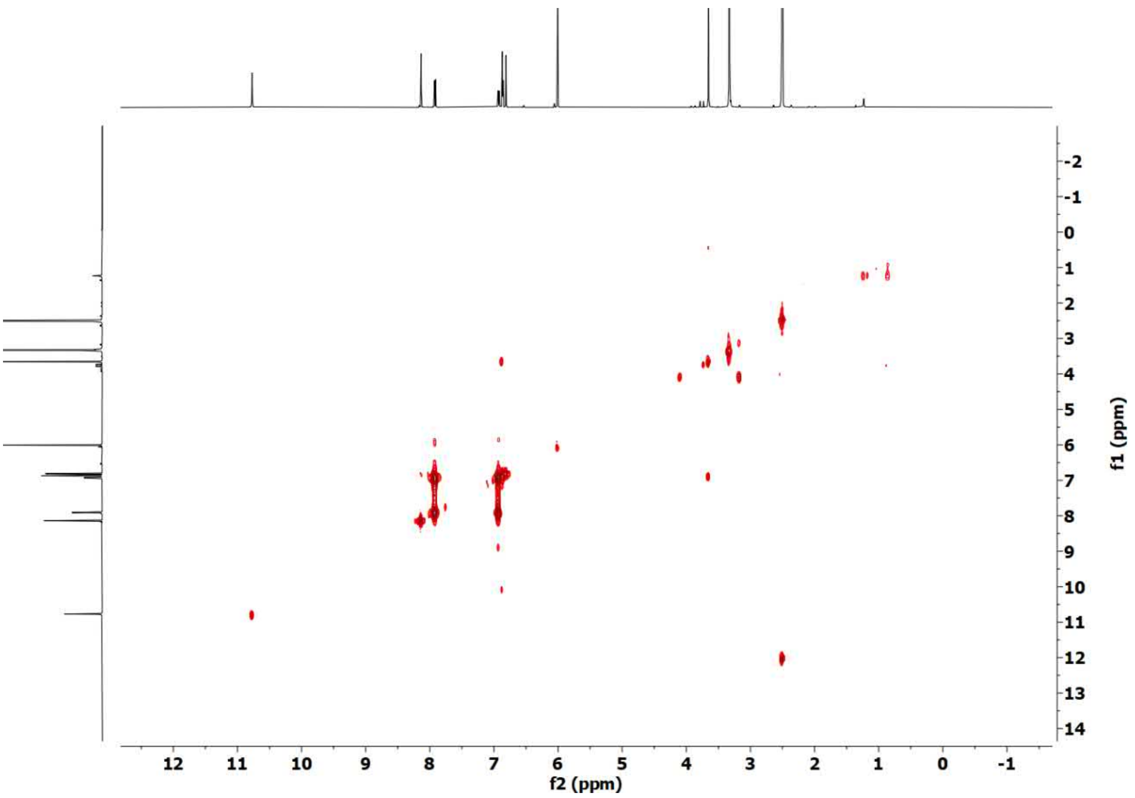

Figure S114. COSY (500 MHz, DMSO-d6, 25 ºC) spectrum of maximaisoflavone (**17**)

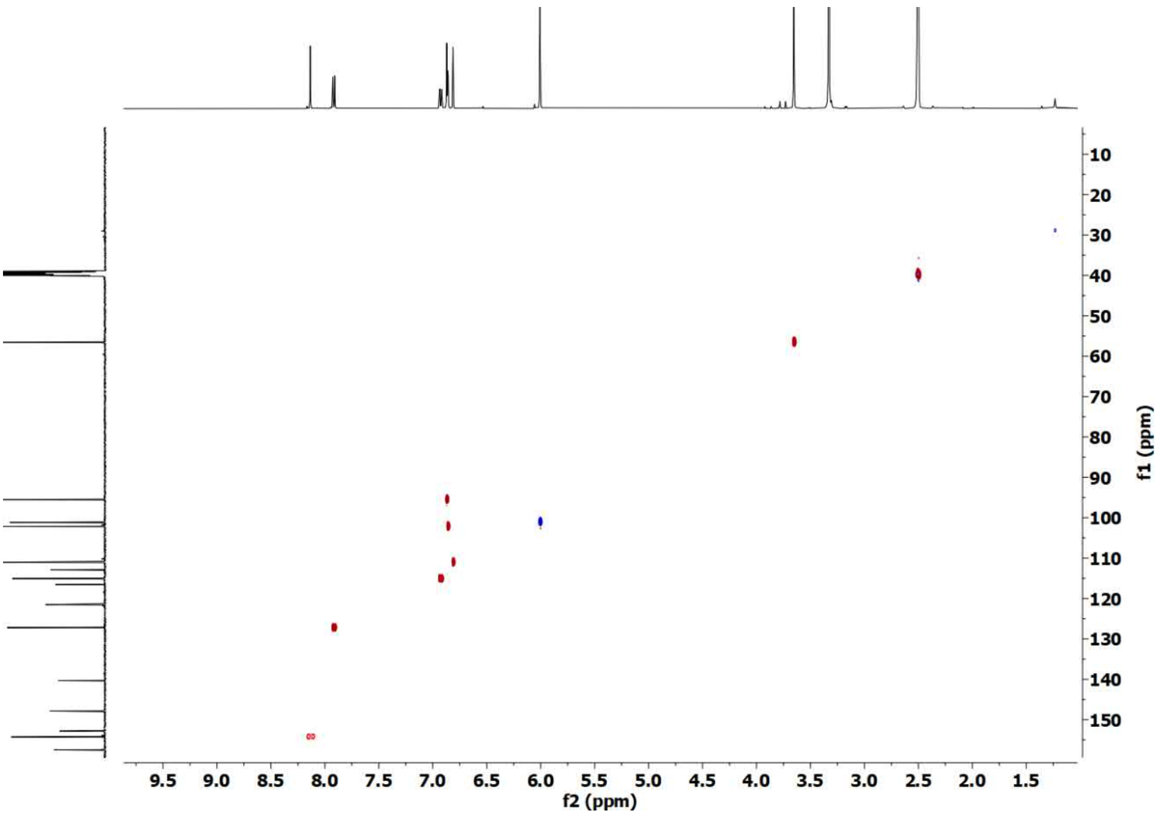

Figure S115. HSQC (500/125 MHz, DMSO-d6, 25 ºC) spectrum of maximaisoflavone (**17**)

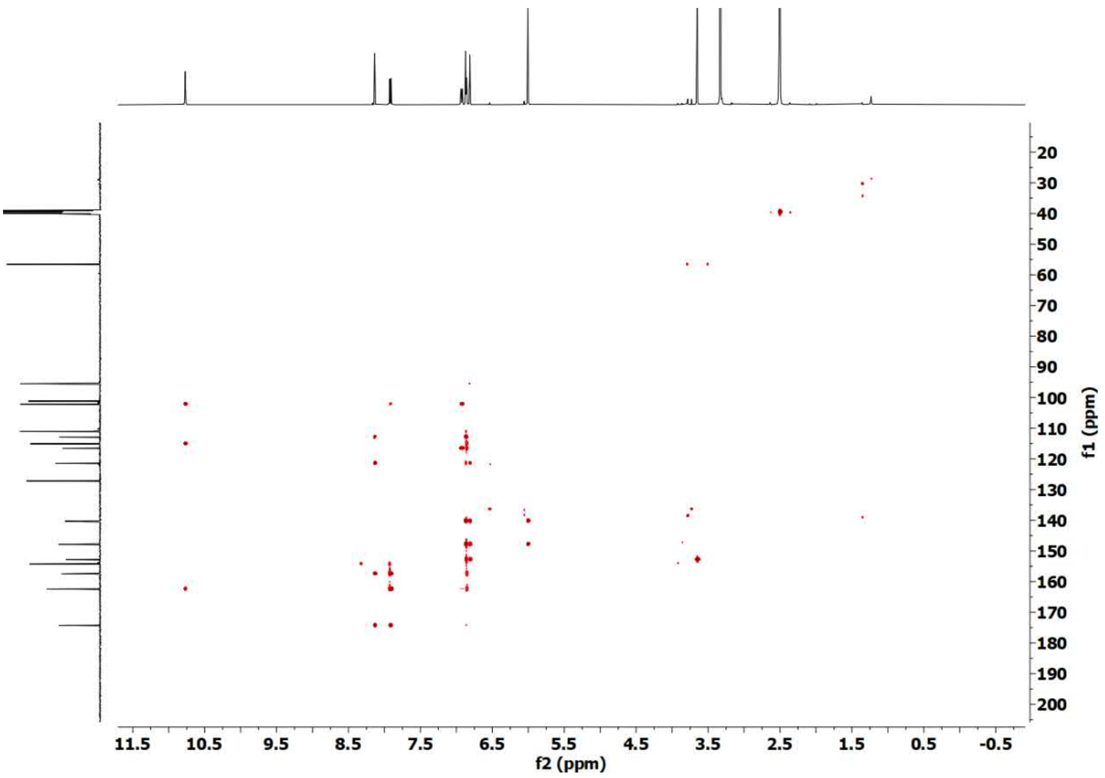

Figure S116. HMBC (500/125 MHz, DMSO-d6, 25 ºC) spectrum of maximaisoflavone (**17**)

Spectroscopic Data of Milldurone (18)

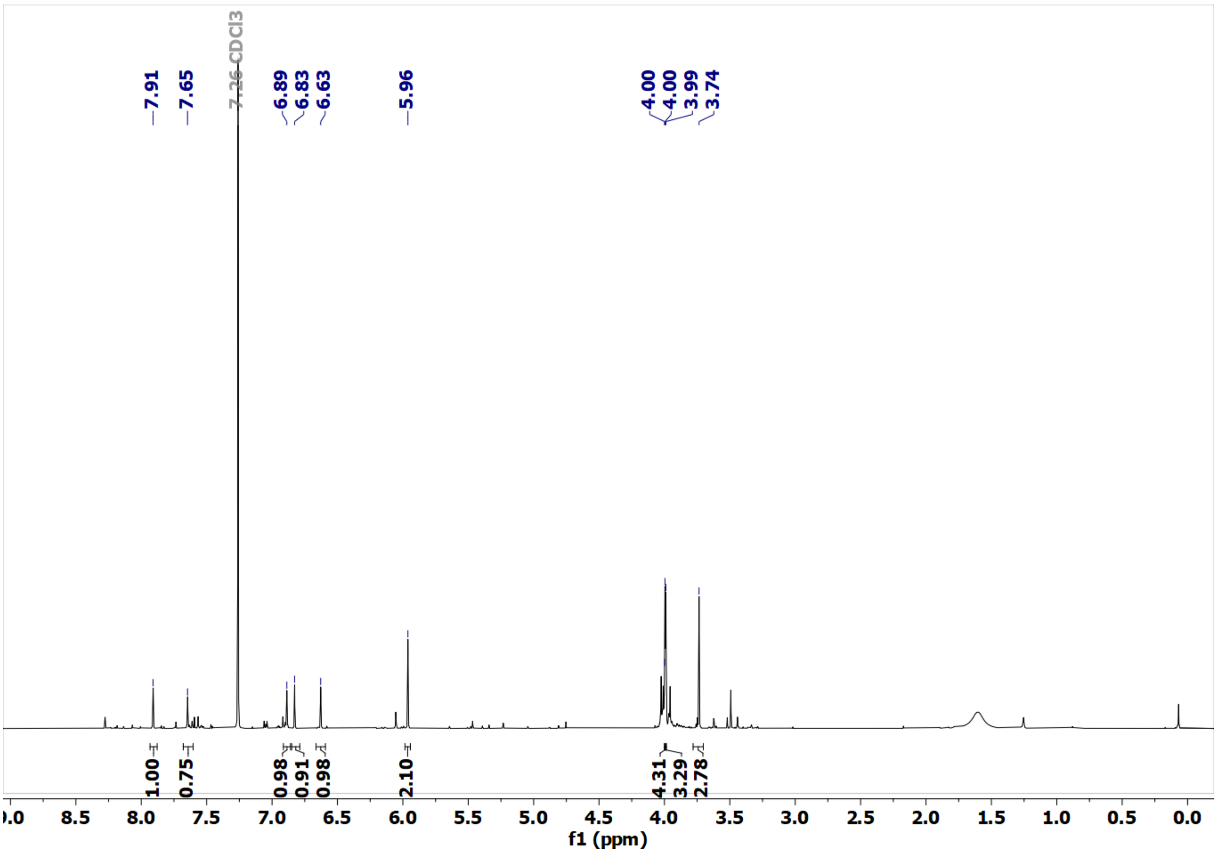

Figure S117. ^{1}H NMR (500 MHz, $CDCl_3$, 25 ºC) spectrum of milldurone (**18**)

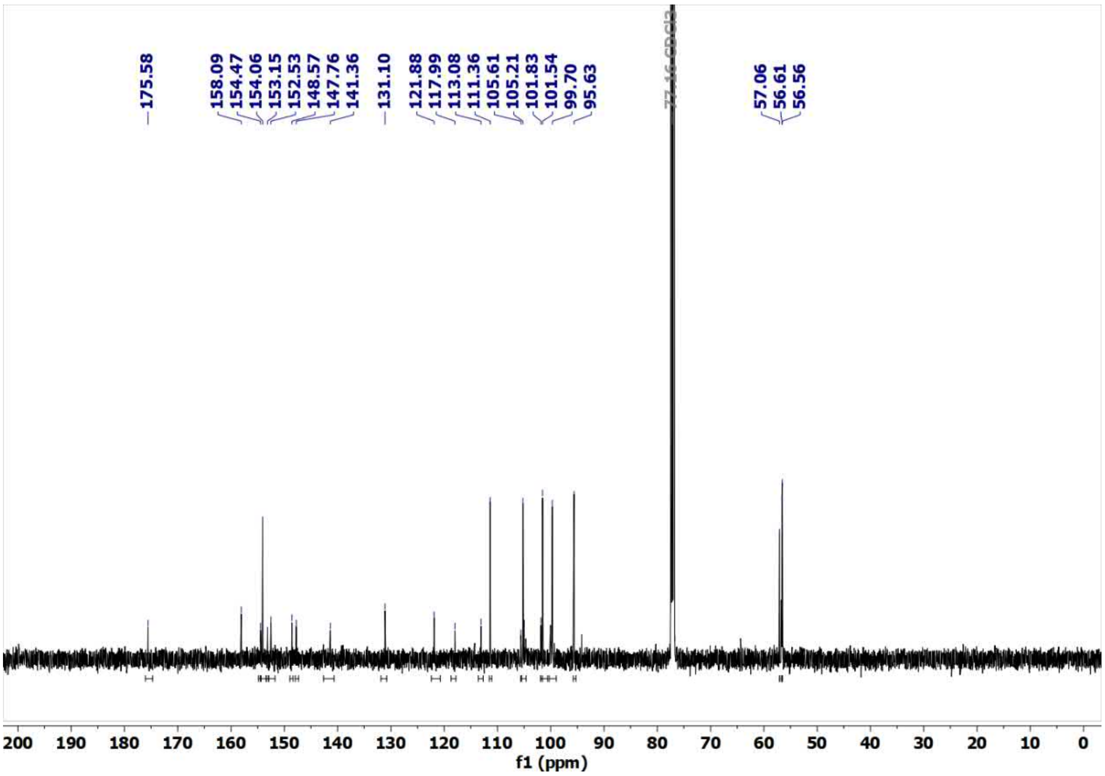

Figure S118. ^{13}C NMR (125 MHz, $CDCl_3$, 25 ºC) spectrum of milldurone (**18**)

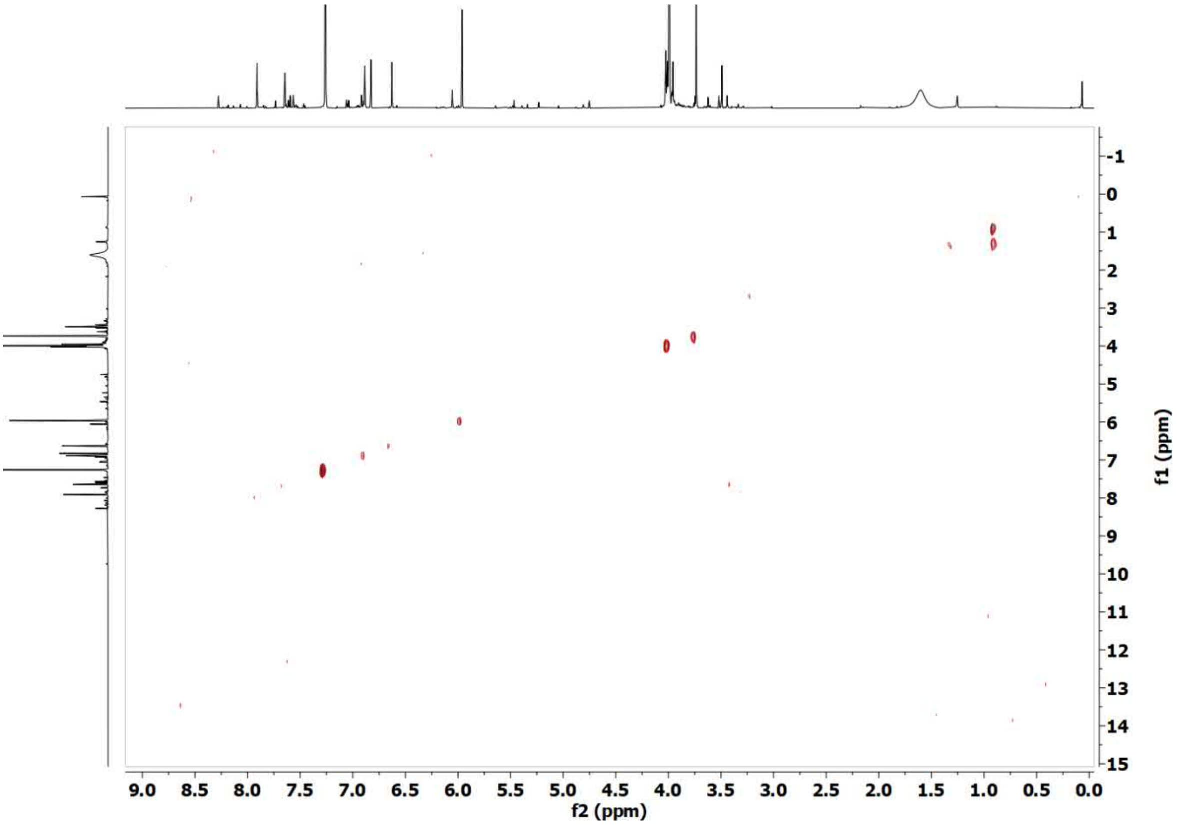

Figure S119. COSY (500 MHz, $CDCl_3$, 25 ºC) spectrum of milldurone (**18**)

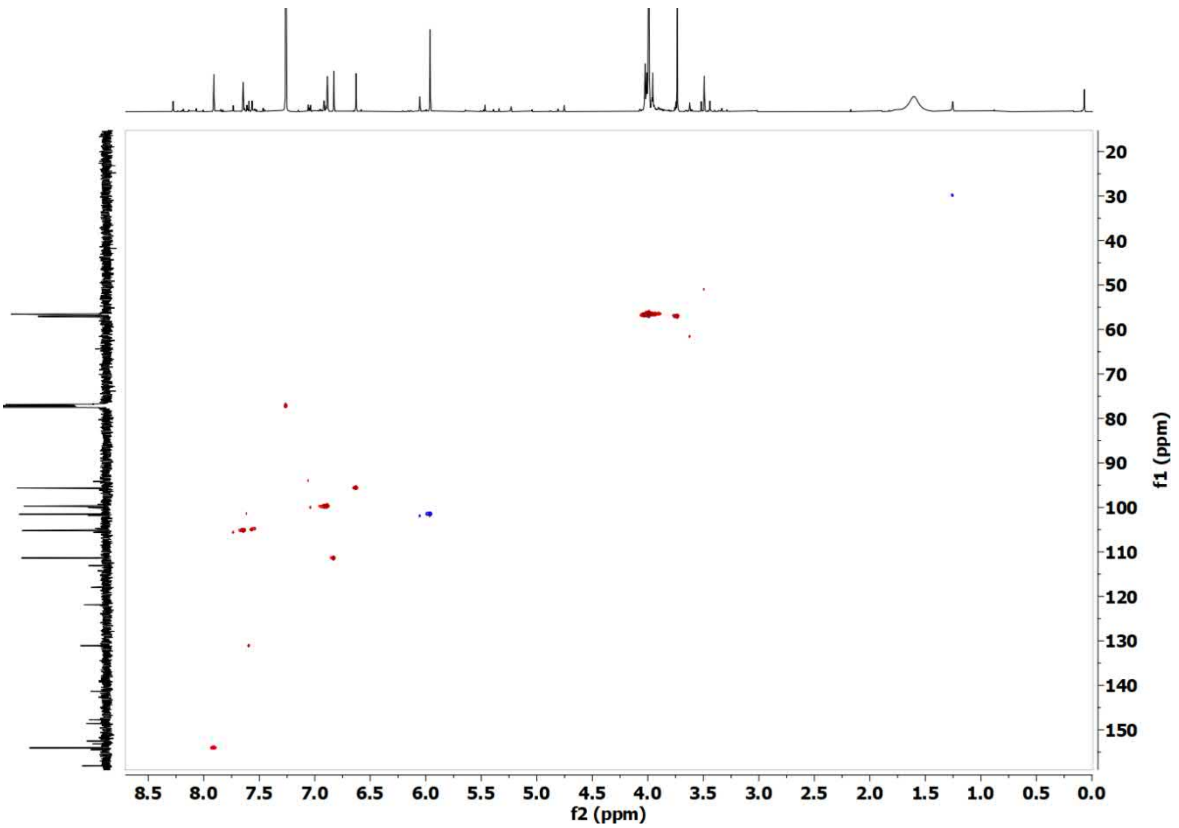

Figure S120. HSQC (500/125 MHz, $CDCl_3$, 25 ºC) spectrum of milldurone (**18**)

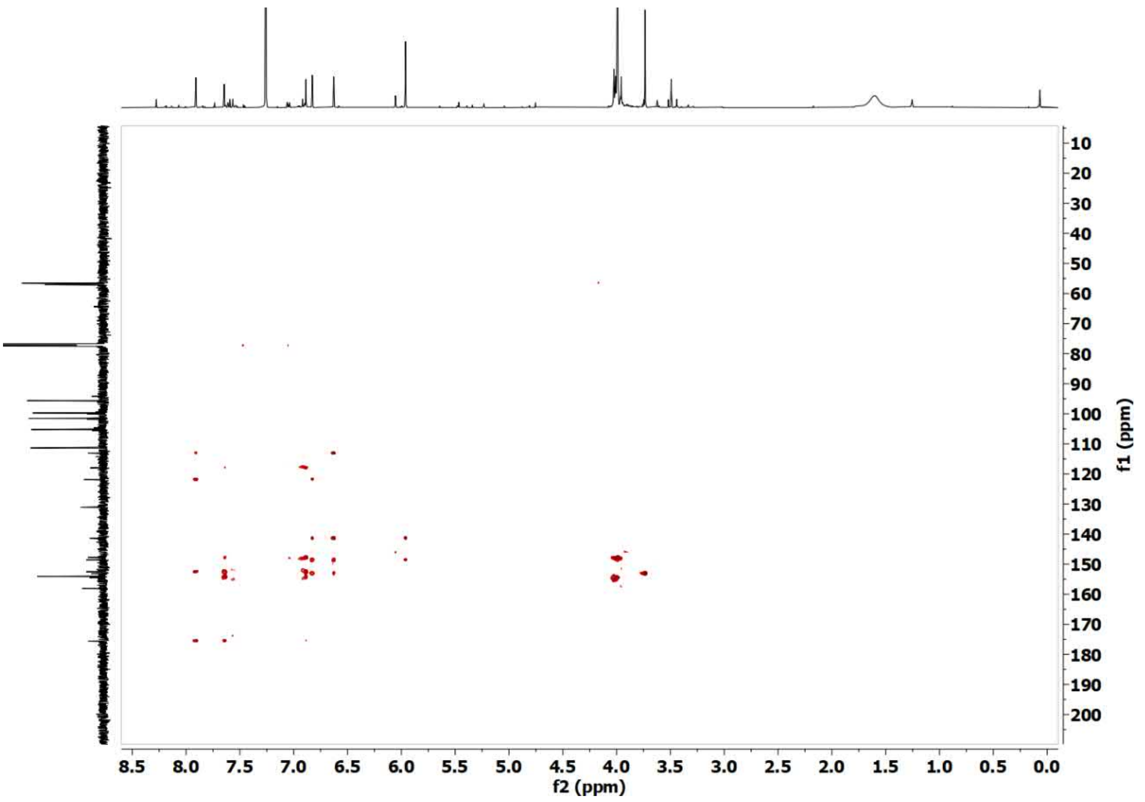

Figure S121. HMBC (500/125 MHz, $CDCl_3$, 25 ºC) spectrum of milldurone (**18**)

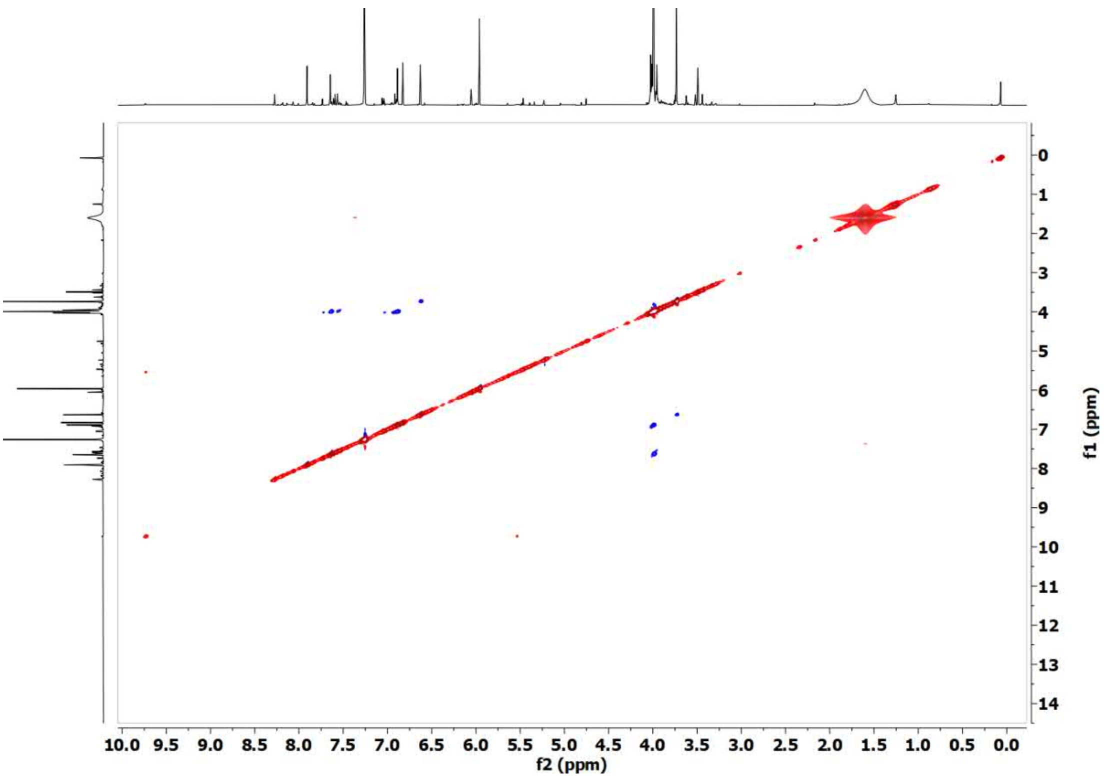

Figure S122. NOESY (500 MHz, $CDCl_3$, 25 ºC) spectrum of milldurone (**18**)

Spectroscopic Data of Isobava Chromene (19)

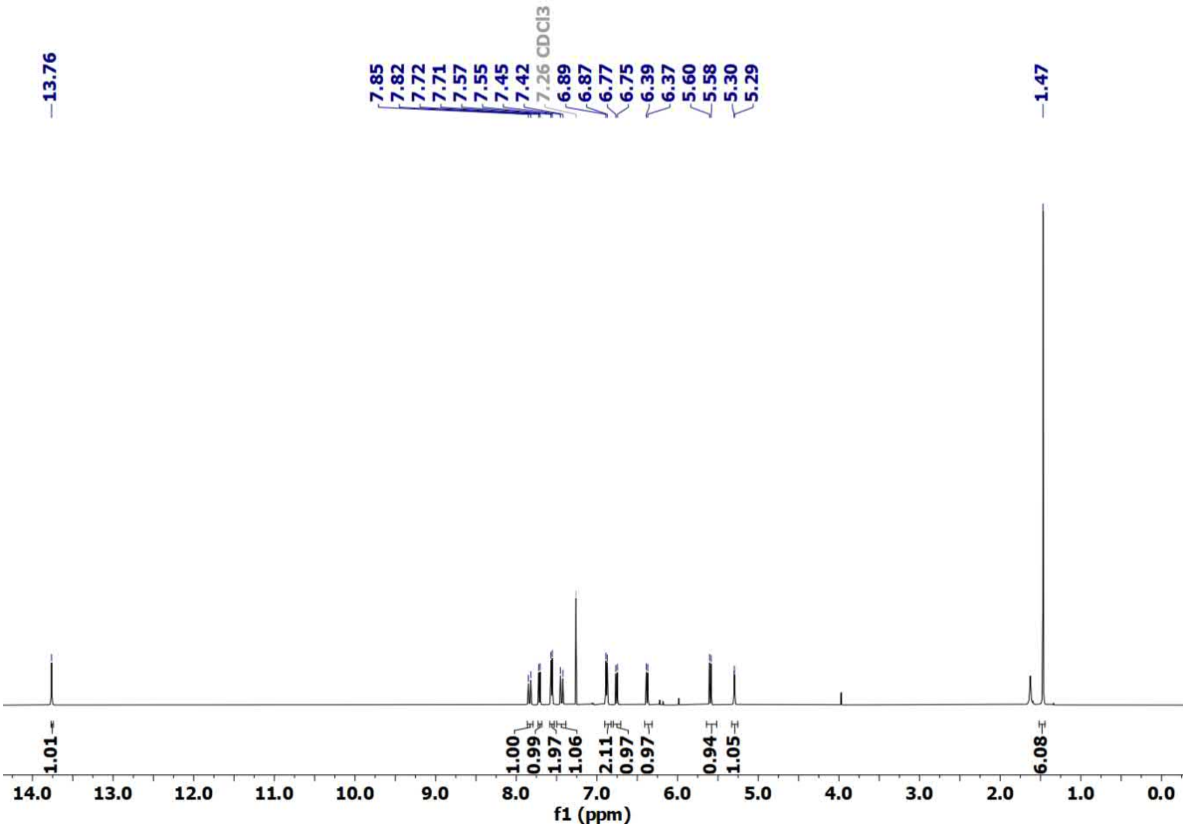

Figure S123. ^{1}H NMR (500 MHz, $CDCl_3$, 25 ºC) spectrum of isobava chromene (**19**)

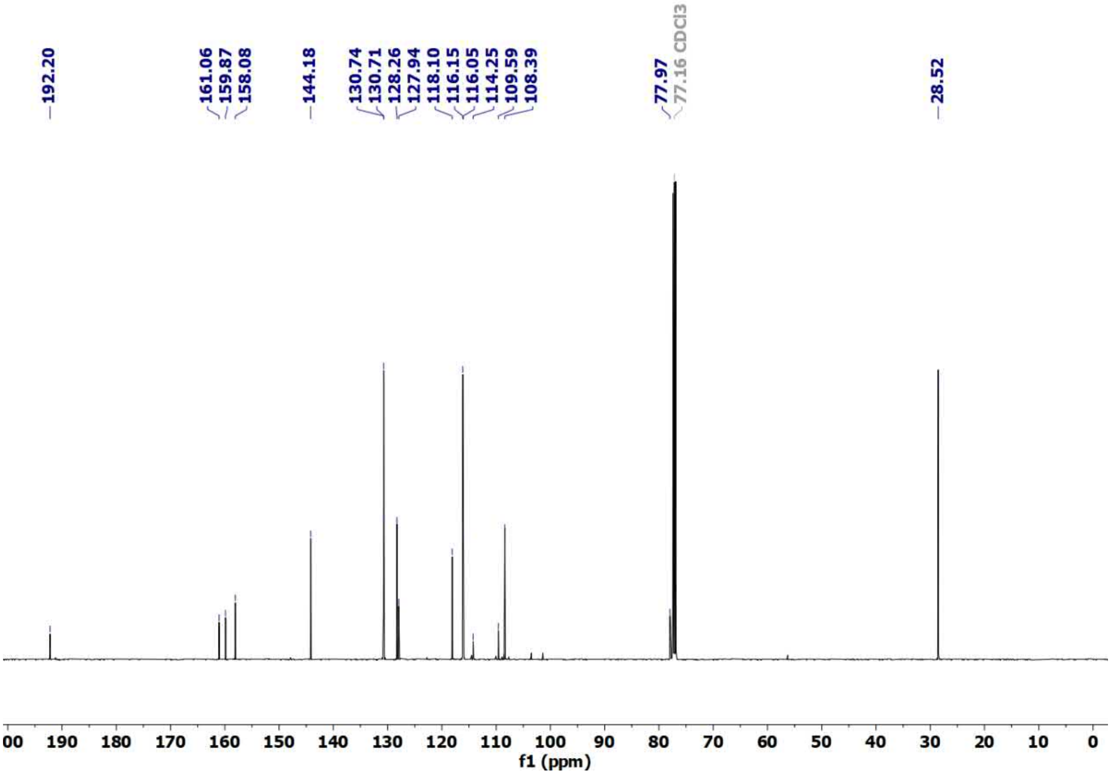

Figure S124. ^{13}C NMR (125 MHz, $CDCl_3$, 25 ºC) spectrum of isobava chromene (**19**)

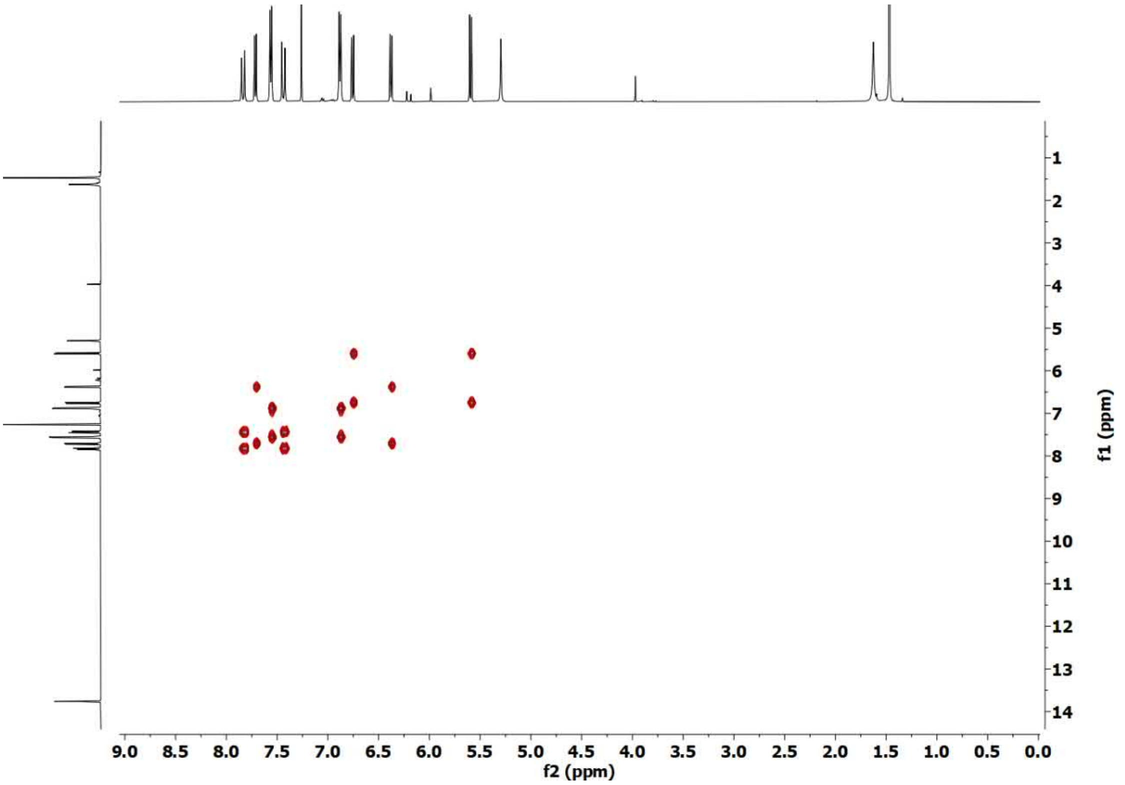

Figure S125. COSY (500 MHz, $CDCl_3$, 25 ºC) spectrum of isobava chromene (**19**).

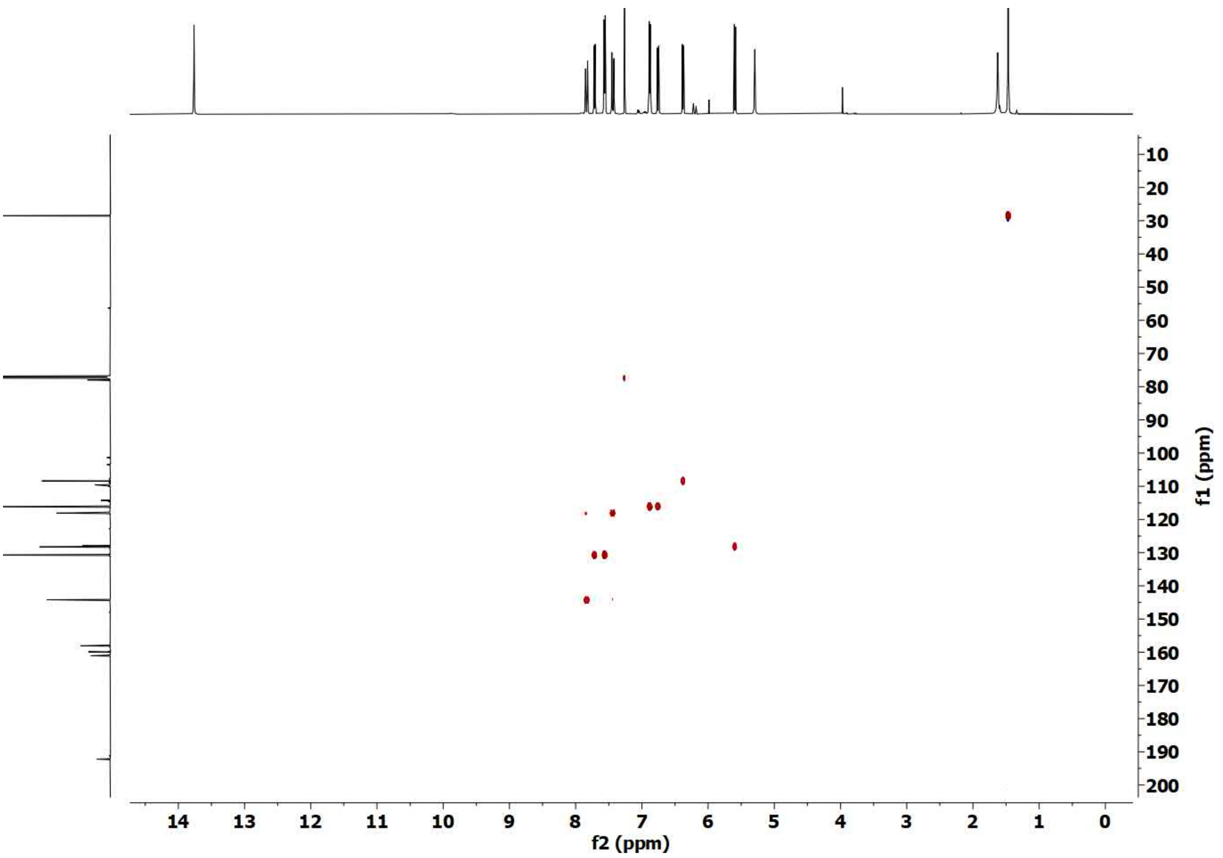

Figure S126. HSQC (500/125 MHz, $CDCl_3$, 25 ºC) spectrum of isobava chromene (**19**).

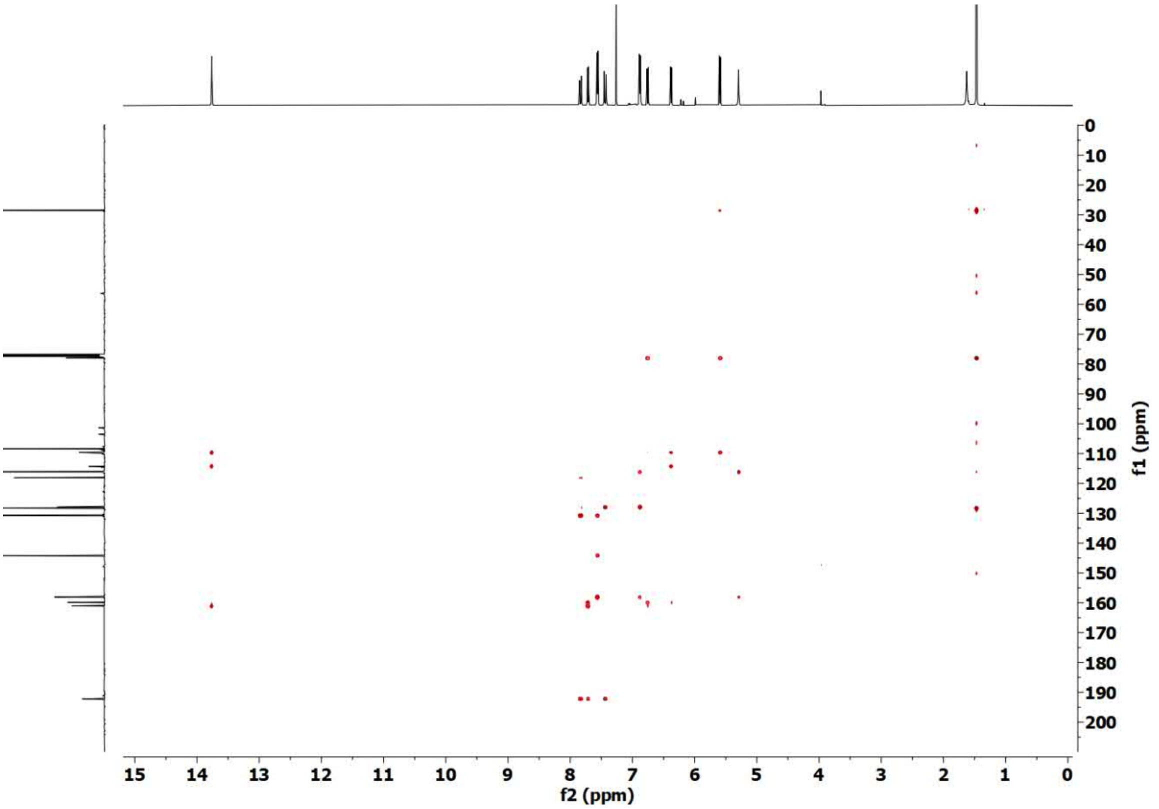

Figure S127. HMBC (500/125 MHz, $CDCl_3$, 25 ºC) spectrum of isobava chromene (**19**).

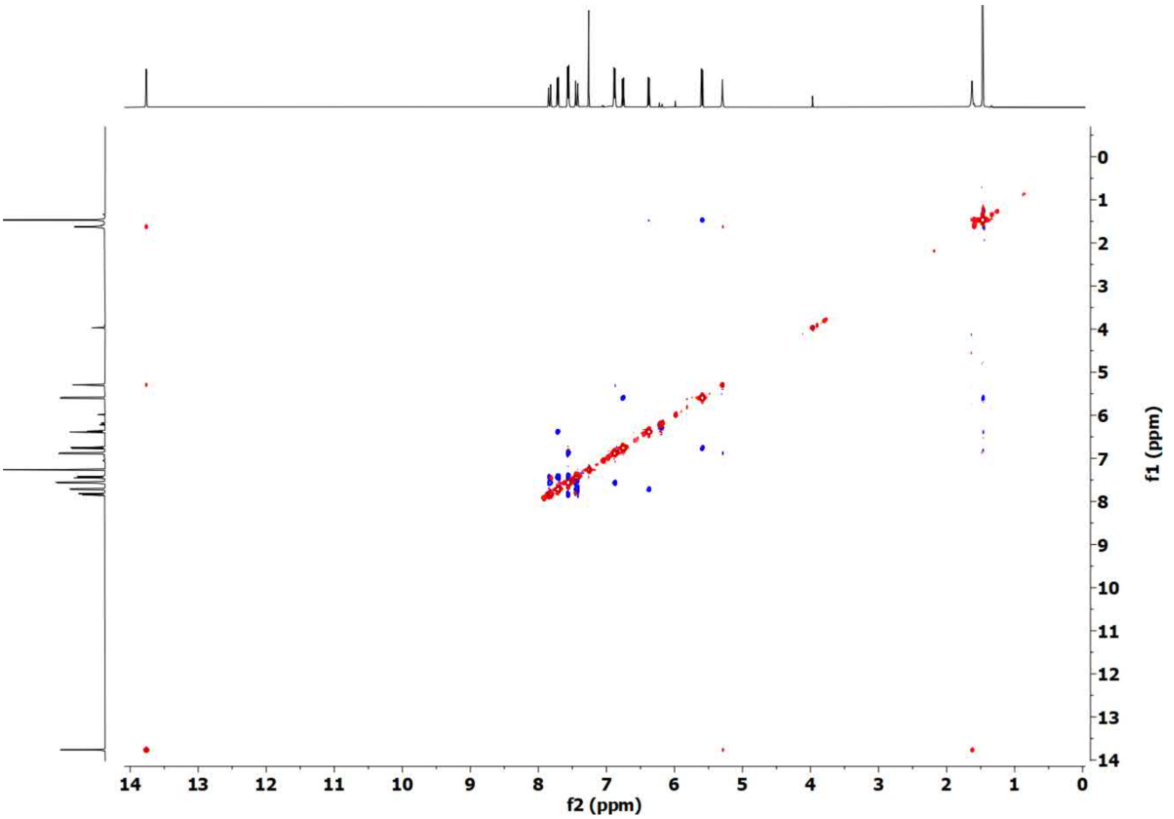

Figure S128. NOESY (500 MHz, $CDCl_3$, 25 °C) spectrum of isobava chromene (**19**).

Antiviral Activity and Cytotoxicity

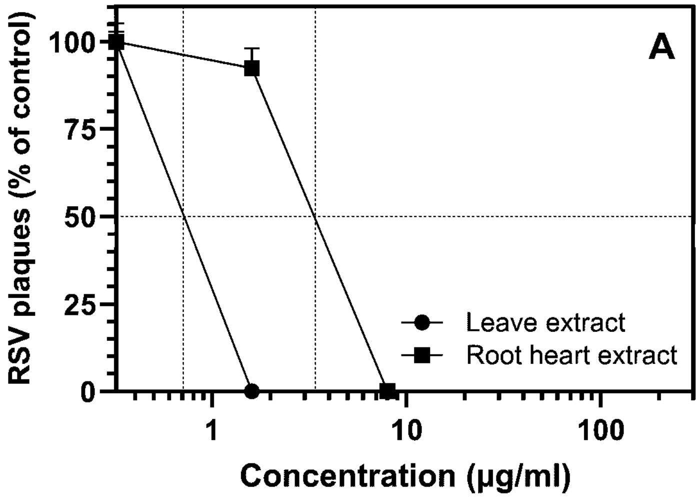

Figure S129. Anti-RSV (IC_{50}) of the crude extracts of leaves and root heart of *Milletia oblata ssp teitensis The results are expressed as a percentage of a number of viral plaques (PFU) developed in HEp-2 cells treated with different concentrations of test extract relative to DMSO treated controls. Three separate experiments each in duplicates were performed.*

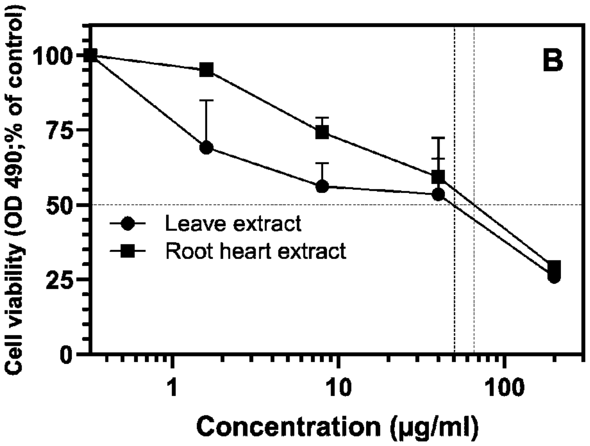

Figure S130. Cell viability in presence of the crude extracts of leaves and root heart of *Milletia oblata ssp teitensis*. The HEp-2 cells were incubated for 3 days at 37 °C with specific concentration of test extract. After addition of the tetrazolium (MTS)-based reagent the absorbance was recorded at 490 nm. The results are expressed as a percentage of absorbance developed in extract treated cells relative to DMSO treated controls. Three separate experiments each in duplicates were performed

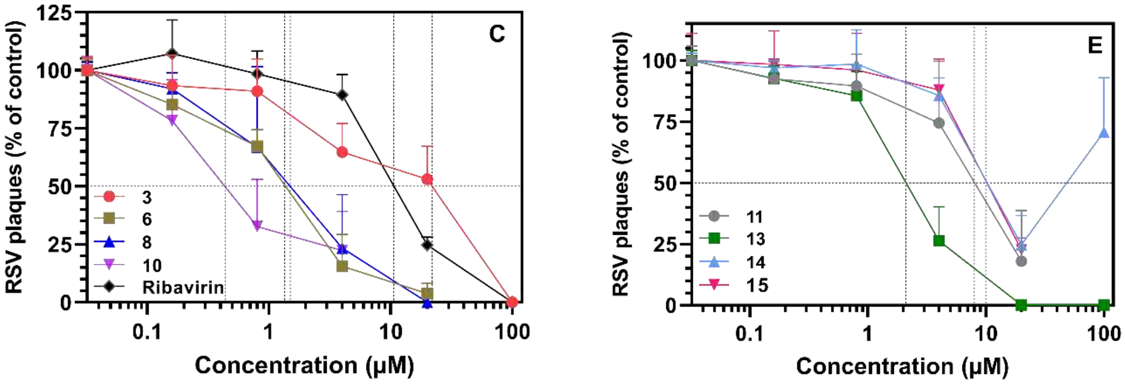

Figure S131. Anti-RSV (IC_{50}) of **3**, **6**, **8**, **10**, Ribavirin, **11**, **13**, **14**, **15** of *Milletia oblata ssp teitensis*. For explanations see legend to Figure S129.

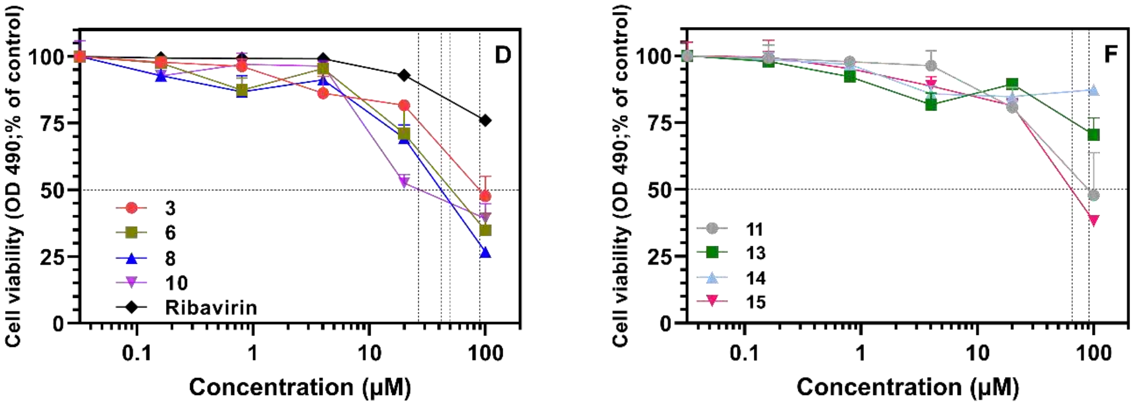

Figure S132. Cell viability in presence of **3**, **6**, **8**, **10**, Ribavirin, **11**, **13**, **14**, **15** of *Milletia oblata ssp teitensis*. For explanations see legend to Figure S130.

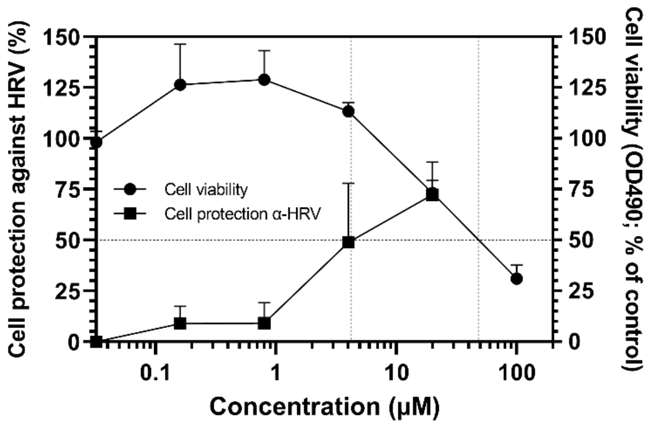

Figure S133. Anti-HRV-2 (IC_{50}) of **3** of *Milletia oblata ssp teitensis* in HeLa cells. In the cell protection against HRV assay, the cells were incubated in the presence of specific concentrations of test compound for 3 h, then inoculated with HRV and incubated for further 3 days at 34°C. Two separate experiments were performed, and the cell protection against HRV-2 was calculated according to formula described in the Method section. In the cell viability assay, the cells were incubated with test compound for 3 days at 34°C. For further explanations see legend to Figure S130.

Table S1. **Anti-RSV activity, cytotoxicity for HEp-2 cells, and selectivity indices (SI) of *Milletia oblata* extracts. For explanations see legend to Table 3.**

Compound/extract	Anti-RSV activity	Cytotoxicity		SI
	IC_{50} (μg/mL)	CC_{50} (μM)	Cytostatic activity[a]	(CC_{50}/IC_{50})
Leaf extract	0.7	50	PCS (1.6 μg/mL)	71.4
Root wood extract	3.4	66	PCS (8 μg/mL)	19.4

Optical Spectroscopy

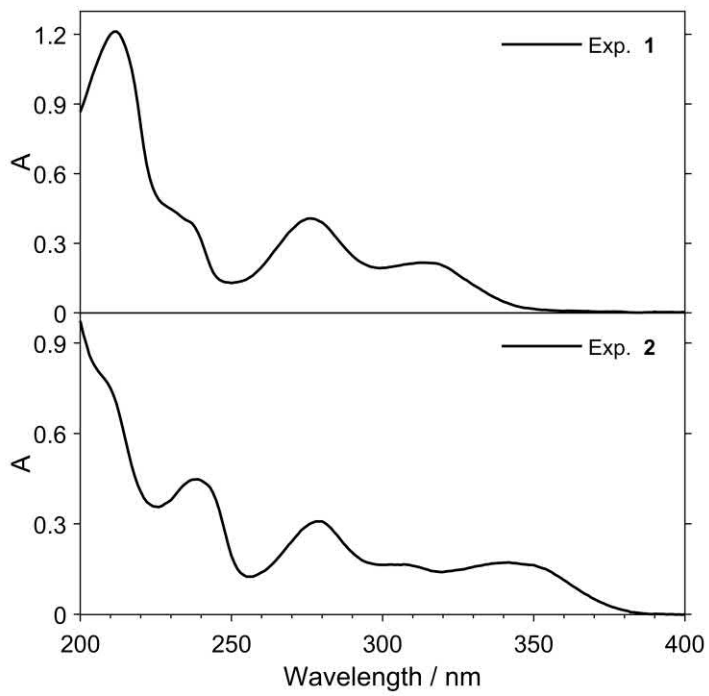

Figure S134. The absorbance spectra of oblarotenoids E (**1**) and F (**2**)

Experimental vs Calculated IR for Oblarotenoid E (1)

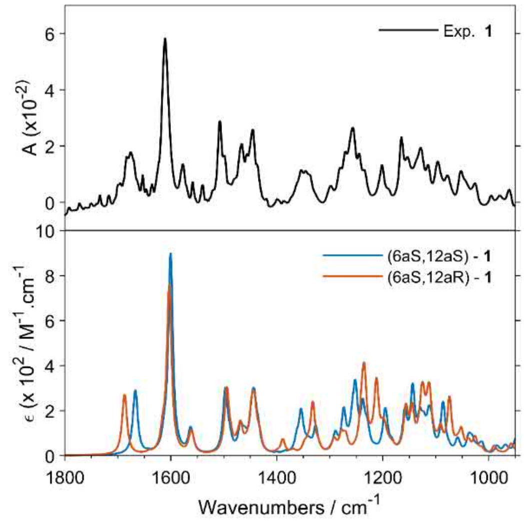

Figure S135. Experimental (black) vs calculated IR for (6a*S*, 12a*S*)-**1** (blue) and (6a*S*, 12a*R*)-**1** (red)

IR spectrum for Oblarotenoid F (2)

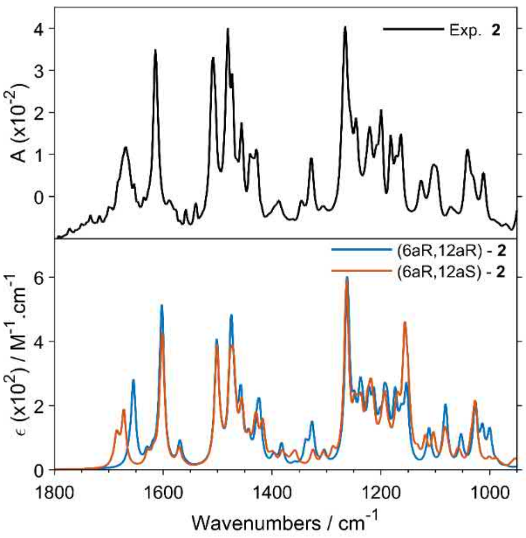

Figure S136. Experimental (black) vs calculated IR for (6a*R*, 12a*R*)-**2** (blue) and (6a*R*, 12a*S*)-**2** (red)
